# Applying Deep-Learning-Driven *De Novo* Design to Hit Identification: A Case Study on A_2A_ Adenosine Receptor Antagonists

**DOI:** 10.1021/acs.jmedchem.6c00231

**Published:** 2026-06-19

**Authors:** Margherita Persico, Alessandra Micoli, Veronica Salmaso, Agostino Cianciulli, Stefano Moro, Giampiero Spalluto, Michela Buccioni, Gabriella Marucci, Rosaria Volpini, Alfonso Pozzan, Fabrizio Micheli, Stephanie Federico

**Affiliations:** ¥ Department of Chemical and Pharmaceutical Sciences, Via Licio Giorgieri 1, Trieste 34127, Italy; § Aptuit, an Evotec Company, Via Alessandro Fleming 4, Verona 37135, Italy; † Molecular Modeling Section (MMS), Department of Pharmaceutical and Pharmacological Sciences, University of Padova, via Marzolo 5, Padova 35131, Italy; ∥ Medicinal Chemistry Unit, School of Pharmacy, University of Camerino, Camerino 62032, Italy

## Abstract

Artificial intelligence is increasingly applied in early drug discovery to accelerate hit identification and reduce costs. In this study, we implemented an AI-driven *de novo* design workflow using REINVENT to generate novel antagonists for the A_2A_ adenosine receptor, a validated target for neurodegenerative diseases. The approach combined ligand-based and structure-based components with pharmacokinetic considerations, including blood–brain barrier permeability, within a multiparameter optimization scoring function. Two generative runs were performed: the first, with balanced scoring weights, yielded inactive 1,2,4-triazole derivatives, while an alternative filtering pipeline identified a micromolar hit. A second run emphasizing structural constraints and key receptor interactions produced three active compounds with nanomolar affinity and predicted CNS permeability. These findings highlight the critical role of scoring function parametrization and filtering strategies in AI-driven drug design and demonstrate the potential of reinforcement learning to explore chemical space for CNS-targeted ligands.

## Graphical abstract

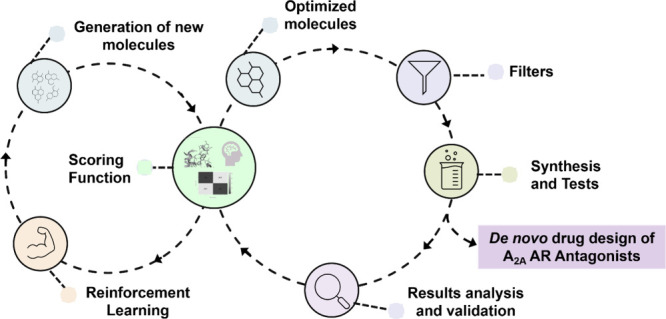

## Introduction

The early drug discovery (EDD) process includes the development of a huge number of compounds aimed to find the most effective one, requiring time, funds, and materials.^1,2^ In addition, the classical hit identification process relies on screening existing libraries, which makes patentability more difficult since many of the screened molecules are already disclosed in prior art. Most compounds developed in EDD fail in preclinical and clinical phases, mostly for issues related to pharmacokinetics, toxicity, and efficacy, being an environmentally harmful process.^3^ Therefore, the development of general approaches aimed at designing novel compounds optimized for both the pharmacokinetic and pharmacodynamic profiles from the EDD process is a major challenge.

In the contemporary context, a strategy that is gaining increasing attention is the implementation of artificial intelligence (AI) in *de novo* drug design. AI technology can automatically manage large quantities of data using algorithms to mimic human activity and solve complicated problems, and there are several examples of its use to accelerate the hit-to-lead phase, thereby reducing time, costs, and wastage.^4–7^ However, there are limits and challenges associated with this technology.^8^ One of the main issues is related to the necessity of large amounts of data on the selected target, together with its consistency, accuracy, and reliability, fundamental to build and train the machine learning (ML) models.^8–10^ Other major challenges are related to ethics, including automatization of processes resulting in job losses,^8^ and the lack of universality in current approaches, which are generally target-specific.^11^ Moreover, nowadays, results derived from AI must be validated and interpreted by researchers, thus suggesting the impossibility of these tools to substitute for human expertise at this stage.^8–10^

Most of the methods reported in the literature that use AI in the early drug discovery process are ligand-based approaches that use ultralarge libraries to virtual screen hit compounds.^12–16^ In recent years, AI-based *de novo* drug design approaches have increasingly emerged, with a growing—although still limited—adoption of structure-based components in the generative phase.^17–23^ Thus, exploitation of AI tools that include a structural component of the target in the generative phase is of high interest and represented the aim of this work. The A_2A_ adenosine receptor (A_2A_ AR) has been identified as a valid target to be used for this purpose. This is due to the high number and variety of published ligands for the target, which displayed high homogeneity of activity/affinity data, if compared to other targets, along with a huge number of deposited cocrystal structures with the receptor.^24–29^ In addition, due to the expertise of our research group and to their therapeutic potential, we decided to focus our project in the development of antagonists for the receptor.

Briefly, A_2A_ AR belongs to the G-protein-coupled receptor family of ARs, together with A_1_, A_2B_, and A_3_ ARs.^30–33^ A_2A_ AR has been demonstrated to play a key role in vasodilation,^34–36^ in cancer (e.g., for its role in suppressing the immune system),^37–39^ and especially in neuroinflammatory and/or neurodegenerative diseases.^40^ Concerning Parkinson’s disease,^41–43^ the A_2A_ AR antagonist istradefylline has been approved and is currently in clinical use.^44–47^ Moreover, A_2A_ AR has also been implicated in Alzheimer’s disease, amyotrophic lateral sclerosis, and Huntington’s disease, further strengthening the interest in A_2A_ AR ligands targeting the central nervous system for the treatment of these conditions.^40^

In this work, an *in silico* workflow was established, encompassing the entire process from data collection to the identification of novel targets for synthesis, with the aim of developing new A_2A_ AR antagonists able to cross the blood–brain barrier (BBB). To design these novel molecules, we employed REINVENT, an AI-based platform developed by AstraZeneca that applies multiparameter optimization (MPO) for *de novo* compound generation.^7,48–50^ This tool leverages reinforcement learning to refine molecular design within a defined chemical space, guided by a custom scoring function.^49,50^ The generated compounds were subsequently filtered and prioritized using a combination of AI/ML methodologies and traditional computational techniques, including molecular docking.

The primary objective of this study was to gain an in-depth understanding of the generative tool by evaluating both its strengths and its limitations. To achieve this, the scoring function was designed to include a target-based component (i.e., docking at the 3D structure of the receptor and relative score) as well as two ligand-based components: one concerning activity (e.g., machine learning activity model) and the other related to BBB permeation (e.g., central nervous system multiparameter optimization, CNS-MPO).^51^ Moreover, on a second iteration following the initial results, we included interaction-specific constraints to interact with a specific protein residue (Asn253) as a pure docking-driven score could miss this important interaction. These are the main differences from the seminal study by Thomas et al.,^17^ in which structure-based docking serves as the primary reinforcement-learning objective, while drug-like properties or specific residues interactions are considered secondarily. Then, in our work, particular attention was devoted to examining how the parametrization of the scoring function and the application of postprocessing filters affect the generative process and influence the prioritization of distinct chemotypes.

## Results and Discussion

New molecules were generated using a custom Evotec version of REINVENT 3.2 (https://github.com/MolecularAI/Reinvent), a *de nov*o design tool based on a recurrent neural network coupled with a reinforcement learning loop. This system performs multiparameter optimization (MPO) while generating novel compounds. To focus the generation of new molecules on a specific chemical space, the process was primed using an “inception” strategy, where three known antagonists were used as initial seeds to guide the model’s early exploration (Figure S1).^52–54^ Additionally, a diversity filter was applied to ensure broad coverage of the chemical space and promote structural variety among the generated molecules. As already mentioned, the core element of the generative model is the scoring function.

### Scoring Function

The scoring function was constructed from three primary components designed to identify orally bioavailable and CNS-permeable A_2A_ AR antagonists: (1) a ligand-based component, (2) a structure-based component, and (3) a pharmacokinetic component. This composite function assigns each molecule a multiparameter optimization (MPO) score ranging from 0 to 1. Molecules with scores exceeding 0.6 were considered to satisfy the design criteria and were retained as model outputs.

The ligand-based component consists of a quantitative structure–activity relationship (QSAR) model. A data set of 9237 A_2A_ AR ligands comprising antagonists, partial agonists, and inverse agonists (see the SI) was compiled from ChEMBL (https://www.ebi.ac.uk/chembl/) and SciFinder (https://www.ebi.ac.uk/chembl/) sources. Data curation, including structure preparation, duplicate removal, and SMILES canonicalization, was performed using KNIME (https://www.knime.com) workflows to ensure that the data set was appropriately processed for QSAR model generation. Multiple QSAR models were developed using different molecular descriptors, such as RDKit physicochemical properties and MACCS fingerprints. Model performance was rigorously assessed via fivefold cross-validation and an external validation set of 74 compounds published in ChEMBL after the initial data collection (Table S1).

The optimal model resulted in a binary classification system based on binding data with a threshold for active fixed at pKi ≥ 7. As shown in Figure 1A, this model achieved an accuracy exceeding 0.8 and a Cohen’s Kappa above 0.7, indicating high predictive reliability.^55,56^ After validation, the external data set was merged into the training set to generate the final production models (Tables S2 and S3). This classification model was integrated into the REINVENT scoring function as a discrete filter: molecules predicted as “active” receive a score of 1, while those predicted as “inactive” are assigned a score of 0.

**Figure 1:**
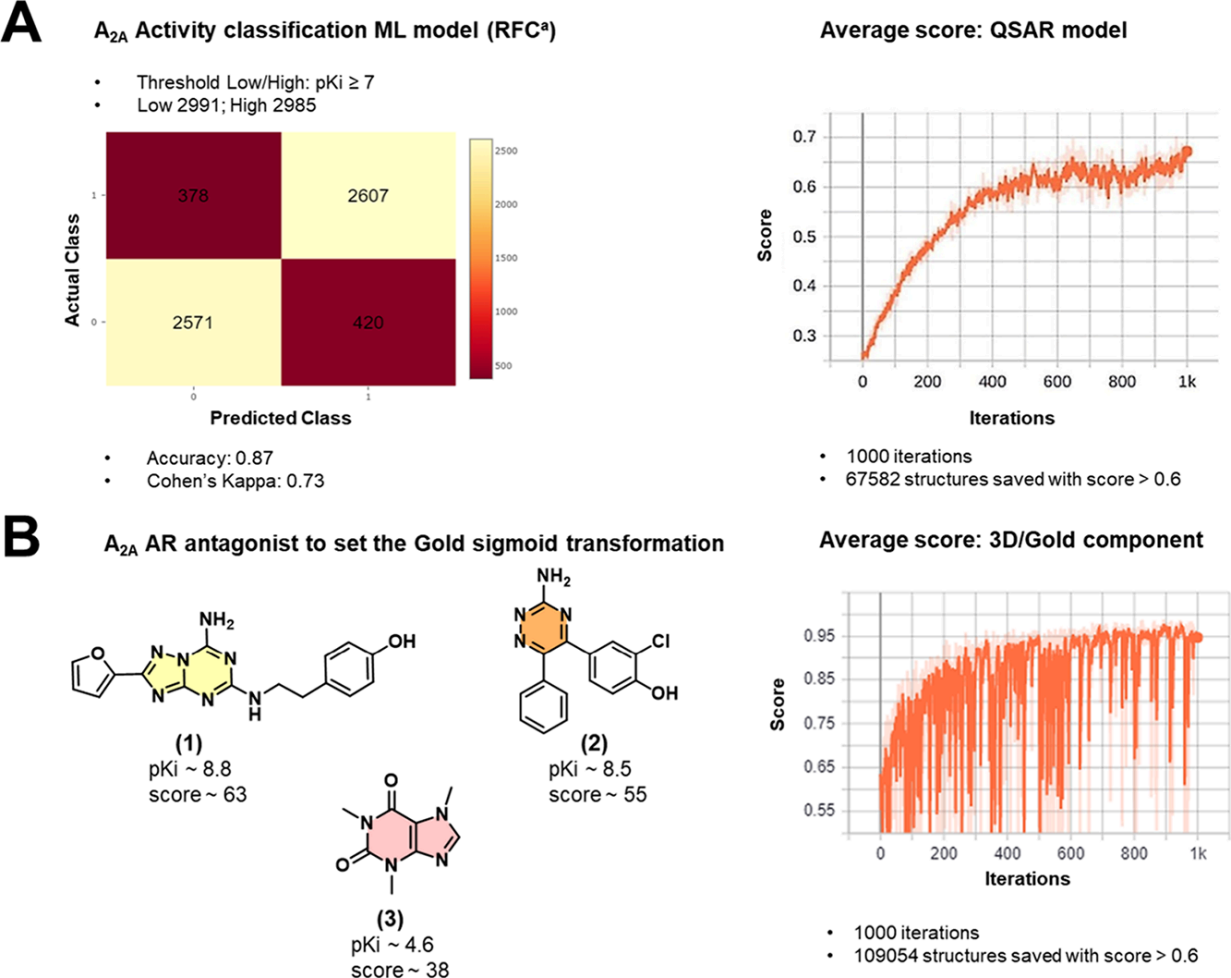
(A) QSAR activity model selected for the scoring function (*K*_i_, binding assays, low/high threshold pKi ≥ 7). Left: confusion matrix and performance metrics of the classification model based on the selected activity threshold. Right: graph generated with TensorBoard showing the average score achieved by REINVENT at each iteration using only the activity model component. (B) Left: sigmoid transformation with the chosen three A_2A_ AR antagonists ZM-241385 (**1**), ligand reported as a cocrystal structure with A_2A_ AR with PDB code5OLZ (**2**), and caffeine (**3**). Right: graph generated with TensorBoard showing the average score achieved by REINVENT at each iteration using only the 3D Gold component. ^a^Random forest classification.

The structure-based component utilizes molecular docking performed with the GOLD program,^57^ employing a crystal structure of the A_2A_ AR (e.g., PDB: 4EIY). To integrate the raw docking results into the final composite score, the values were mapped to a 0–1 range using a sigmoid transformation.

This transformation was calibrated based on three reference antagonists with diverse affinities for A_2A_ AR (Figure 1B): the high-affinity ZM-241385 (**1**) (docking score ca. 63, pKi ca. 8.8),^22^ compound (**2**) (PDB: 5OLZ, docking score ca. 55, pKi ca. 8.5),^21,58^ and the low-affinity caffeine (**3**) (docking score ≈ 38, pKi ca. 4.6).^20^ Consequently, docking scores below 50 are assigned a score of 0, values between 50 and 65 are transformed into a continuous score between 0 and 1, and scores exceeding 65 are assigned a maximum value of 1.

Finally, the pharmacokinetic component of the scoring function consisted of the CNS-MPO parameter, first described by Wager and colleagues, which gives an idea of the ability of each molecule to cross the BBB, by evaluating the adequacy of six specific physicochemical properties.^51^ The optimal range of CNS-MPO was identified between 0 and 6.^51^ Molecules with a score higher than 4 were considered as best candidates for passing the BBB. Therefore, an ascending sigmoid transformation was used to convert the CNS-MPO score (originally on a 0–6 scale) to a 0–1 range for the final composite score calculation. In this case, the transformation was applied as follows: a CNS-MPO score below 3 was assigned a value of 0 in the calculation of the average scoring function; scores between 3 and 4 were mapped to values between 0 and 1; and scores above 4 were assigned a value of 1.

Four MPO scoring functions have been explored to verify the ability of the generative model to optimize the single components, e.g., activity model and 3D/Gold component (Figure 1) and subsequently a combination of these two and of all three components (Figure S2). When combined, the components were uniformly weighted to set the total average score value (0–1).

Molecules from each of the 1000 cycles were collected if their average score was greater than 0.6 and resulted in 67,582 molecules when using the QSAR model alone and 109,054 when using the 3D/Gold component (Figure 1). When both scoring functions (QSAR model and 3D/Gold Score) were combined with equal weighting over the 1000 generative iterations, 97,826 molecules were retained (Figure S2A). Finally, combining all three components (QSAR model, 3D/Gold Score, and CNS-MPO) with an equal weight resulted in 91,115 molecules (Figure S2B).

The large set of molecules generated across the combined runs (365,577 molecules) was refined using KNIME (https://www.knime.com/). As part of the refinement, Tanimoto similarities were calculated with respect to molecules collected in the initial data set to remove identical molecules. Physicochemical properties (e.g., MW, cLogD, tPSA, CNS-MPO, and BBB score) were calculated, and clustering was also performed using structural fingerprints. The total number of molecules after the postprocessing was 365,321.

### Filtering

As the previous process yielded a set of molecules of a scale that required further prioritization, it was necessary to apply more filters to identify a smaller number of promising molecules for synthesis. The filter pipeline is illustrated in Figure 2.

**Figure 2:**
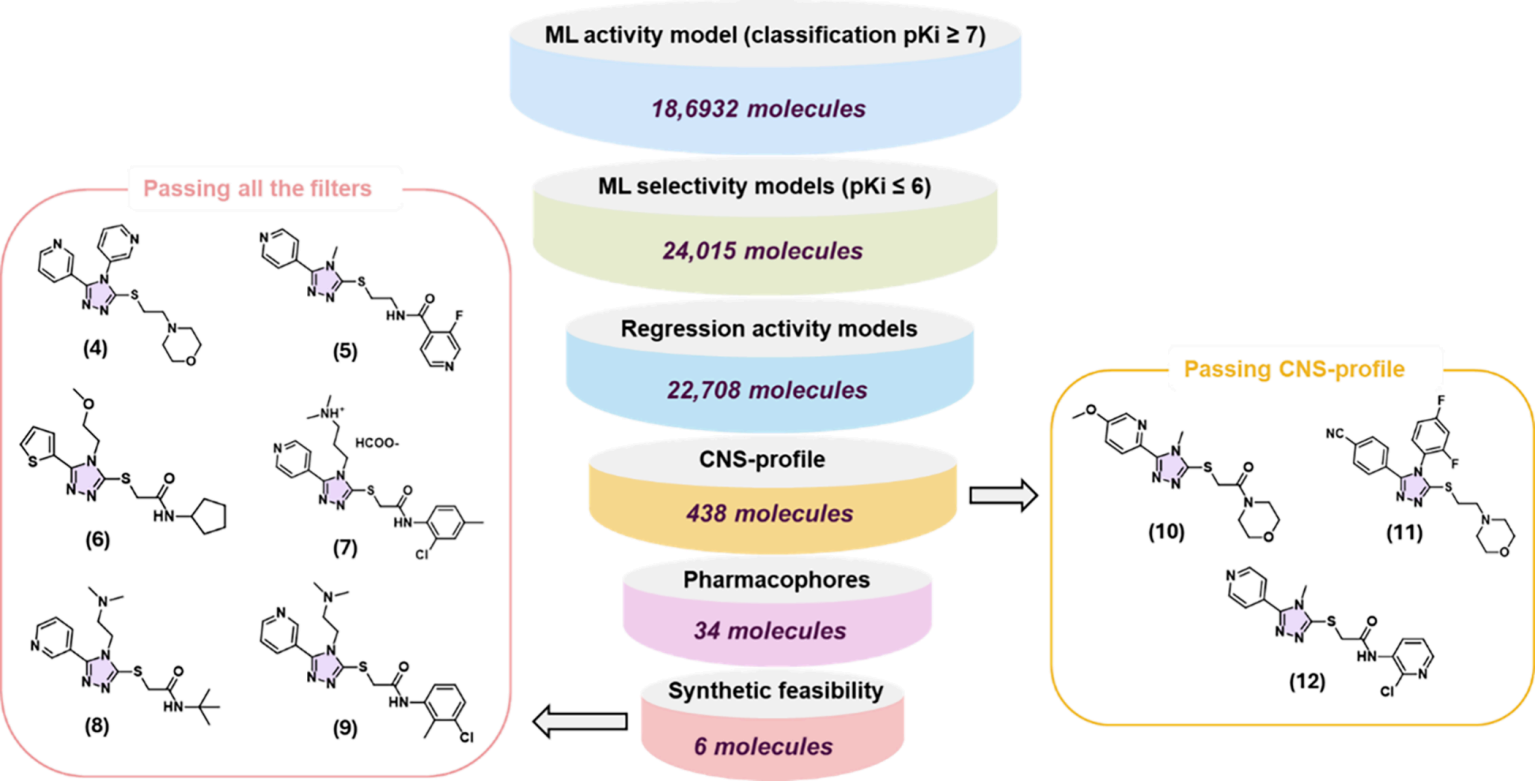
Filters applied to the generated molecules with highlights on the 1,2,4-triazoles selected for the synthesis (**4**–**12**).

The first filter required a predicted affinity greater than 7 toward the A_2A_ AR, as determined by the same binary classification model used in the scoring function. The second filter addressed a critical aspect of adenosine receptor antagonists: selectivity for the target subtype. To this end, additional QSAR classification models were developed for the remaining three adenosine receptor subtypes (A_1_, A_2B_, and A_3_ ARs). To build these models, *ad hoc* data sets for all the three receptors were built by collecting data published in CHEMBL (https://www.ebi.ac.uk/chembl/) for each receptor (see SI). Data included in the data sets were divided into four different files according to the type of assay (e.g., functional or binding assays) and kind of value determined (i.e., *K*_i_ and IC_50_) to have sets ready to be used for the generation of the models. For A_1_, A_2B_, and A_3_ ARs, as well as for A_2A_ AR, classification models have been built by using a graphical interface tool developed by Evotec and a five-fold stratified cross-validation and as descriptors RDKit Phys-Chem + MACCS keys, both using random forest classification (RFC) or support vector classification (SVC). For all the receptors, a pKi threshold ≥6 was set, allowing to filter only compounds with a micromolar *K*_i_ value on the other ARs. For A_1_ and A_3_ ARs, the best models in terms of accuracy and Cohen’s Kappa parameters^55,56^ were noticed to be the one using *K*_i_ + binding data (Table S2), whereas for A_2B_ AR, the best model included IC_50_ + functional data (Table S2), as the latter seems to be more realistic for defining the affinity of the molecules to the receptor. In fact, most of the articles reported functional assays, and only recently, several other reliable assays, including binding assays, have been developed and used in A_2B_ AR ligand discovery.^59–65^ The application of this second filter concerning the selectivity profile allowed the identification of 24,015 molecules to be further filtered.

The third filter evaluated the consistency between two regression models that were created for A_2A_ AR (Table S3): one built with *K*_i_ and binding data and the other with IC_50_ and binding data. Molecules with predicted pKi or pIC_50_ ≥ 7 in both regression models were considered for the next step, with a total of 22,708 surviving molecules. Because the project aims to develop A_2A_ AR antagonists for the treatment of neurodegenerative diseases, molecules were successively evaluated and filtered on the predicted permeability through the BBB using two different parameters: the CNS-MPO and the BBB scores (considering score values higher than 4 for both).^51,66^ The BBB score considers different physicochemical properties with respect to CNS-MPO, which are referred to as fundamental for the permeation through BBB. These parameters are the number of aromatic rings, number of heavy atoms, MWHBN (i.e., a descriptor composed of MW, the number of hydrogen bond donor (HBD) atoms, and the number of hydrogen bond acceptor (HBA) atoms), tPSA, and p*K*_a_ at physiological pH.^66^ According to these two parameters, only 438 molecules were retained. As evident from the data and specifically by the reduction from the initial 22,708 molecules, it is clear that the CNS profile was the most critical factor in narrowing down the selection. This significant decrease can be attributed to the stringent criteria applied in this filtering step. The 438 molecules were evaluated for the original different sources to which they belonged, i.e., by which of the four MPO scoring functions they were optimized. It was identified that most of the molecules that were selected at the end of the application of all the filters were derived from the combination of the QSAR activity model, 3D/Gold component, and CNS-MPO (185 of 438).

At this point, molecules selected from the previous steps were submitted to structural analysis by means of molecular docking. The 438 molecules were prepared for docking through the use of the OpenEye Scientific software. Initial molecules were processed to generate their most probable tautomers and protonation states and assign an initial 3D structure. This process resulted in an ensemble of 505 molecules for the docking campaign. The molecules were docked at the A_2A_ AR structure (PDB ID: 4EIY) using Gold–Goldscore, and 5050 docking poses were collected. Pose selection and molecule prioritization were conducted through a series of filters based on a pharmacophore hypothesis and pattern of interactions with the receptor.

A first pharmacophore filter (P1) was built using the 58 antagonist-bound A_2A_ AR X-ray structures available at the time of the present work in the PDB (https://www.rcsb.org/). The receptor structures were aligned, and the ligands were used to build a consensus pharmacophore hypothesis, matching most of the X-ray structures: this included at least one between an H-bond donor or acceptor, aimed at forming an H-bond with the key residue Asn253, together with an aromatic central core, aimed at forming a π–π interaction with the other key residue Phe168. These two residues were shown to be essential for ligand binding to the receptor.^67–69^ A total of 1263 poses survived the first pharmacophoric filter (Figure S3A), and these were further reduced by selecting poses presenting at least an H-bond (after computing) with Asn253 and with negative electrostatic and van der Waals interaction potential with the receptor, resulting in 364 poses (of 112 unique molecules).

Afterward, the presence of a bidentate hydrogen bond with Asn253, well-known to be a feature of several potent A_2A_ AR antagonists,^21,23,67–70^ was assessed, but this resulted just in three compounds, whose poses showed poor occupation of the binding pocket or tautomeric concerns. For this reason, none of them was selected for the synthesis, and additional pharmacophoric filters were added to find molecules with a better occupation of the binding pocket. Therefore, the 364 poses were filtered according to a second pharmacophore (P2, Figure S3B). In particular, since many of the X-ray antagonists also possess a hydrophobic or aromatic group that extends deep into the binding pocket toward the residue His250, which is also an essential residue for ligand binding,^68,69^ a second hydrophobic/aromatic pharmacophore feature resembling these groups was added to the pharmacophore filter. The resulting filtered poses were in total 271, corresponding to 83 unique molecules.

Finally, pursuing the aim of prioritizing a few molecules for synthesis and pharmacological evaluation, a third pharmacophoric filter (P3) was built and applied. Observing a general absence of the classic bidentate hydrogen bond with Asn253 in the generated poses, we investigated specific X-ray structures lacking this interaction and then analyzed their ligands (PDB IDs: 3REY, 3RFM, 5MZP, 5OLV, 6ZDR, 6ZDV, and 8GNG). Among these structures, chromone ligands of structures 6ZDR and 6ZDV position a further hydrogen-bond acceptor in proximity of the side-chain carbonyl of Asn253, speculatively suggesting possible water-mediated interactions. Furthermore, a water molecule occupies a similar position in structure 5OLV, mediating an interaction between the amide NH group of LUAA47070 and Asn253. Therefore, poses resembling the behavior of the chromone ligands (i.e., sharing a group able to act as an H-bond acceptor centered on the chromone pyranic oxygen) were selected (Figure S3C), resulting in 95 poses corresponding to 34 unique molecules.

The final prioritization was applied to the remaining 34 molecules, focusing on the evaluation of their central cores. To identify redundant nuclei and assess synthetic feasibility, a comprehensive search in SciFinder was performed ensuring that the selected molecules could be efficiently synthesized and are relatively novel. It was noticed that 23 out of 34 selected molecules are 1,2,4-triazoles with substitutions at the 3, 4, and 5 positions (Figure S4). Initially, six of them (**4**–**9**, Figure 2) were selected for the synthesis. As this nucleus was highly represented within the molecules generated by REINVENT, a retrospective analysis of the 1,2,4-triazoles discarded during the later stages of the filtration pipeline was performed by analyzing the synthetic feasibility of the compounds that passed the CNS-profile filter. By this way, we identified three other compounds (**10**–**12**, Figure 2) for the synthesis.

### Synthesis and Binding Affinity Evaluation on the Target for Compounds **4**–**12**

The nine compounds (**4**–**12**) were synthesized as reported in Scheme S1. For the compound (**11**), the derivative containing the −COOH (**11b**) moiety instead of −CN was obtained from the synthetic pathway. All these compounds were evaluated for the affinity to the target by a classical radioligand binding assay and proved to be inactive (*K*_i_ hA_2A_ > 30,000 nM). By checking the origin of these molecules, we noticed that four of them were derived from a scoring function combining the QSAR activity model, the 3D/Gold Score, and the CNS-profile (**4**, **6**, **10**, and **11**), while the others were derived from the combination of the activity model and the 3D/Gold Score (**5**, **7**–**9**, **12**). This distribution suggests that the lack of activity cannot be ascribed to a specific configuration of the scoring function as the inactive compounds originate from diverse component combinations.

It is evident that our initial confidence in this nucleus was driven by the high prevalence of 1,2,4-triazoles within the molecules generated by REINVENT. However, such molecules lack some fundamental characteristics shared by the majority of A_2A_ ARs reported in the literature, i.e., a group able to form a bidentate H bond with Asn253.^19–22,24,27,66–69^ The hypothetical binding modes of the designed triazoles docked at the A_2A_ AR orthosteric pocket (PDB ID: 4EIY, https://www.rcsb.org/) were further visually inspected. The pose of compound **4** is reported in Figure 3 as a prototypical example: the triazole aromatic ring is engaged in a π–π interaction with Phe168, the nitrogen atom at position 2 interacts through an H-bond with Asn253 on transmembrane helix (TM) 6, the 5-aryl substituent is directed at the bottom of the binding pocket making a T-shaped π–π interaction with His250 (TM6), the 4-aryl substituent (here aryl) points toward TM1–2, and the 3-substituent extends toward the extracellular opening of the receptor. This binding mode supports the hypothesis that the inactivity of these molecules might be due to the absence of a moiety capable of forming the bidentate hydrogen bond with Asn253, suggesting that the bidentate H-bond Asn253 should be integrated as a structural constraint in the scoring function during the generative phase and not as an *a posteriori* pharmacophoric filter.

**Figure 3:**
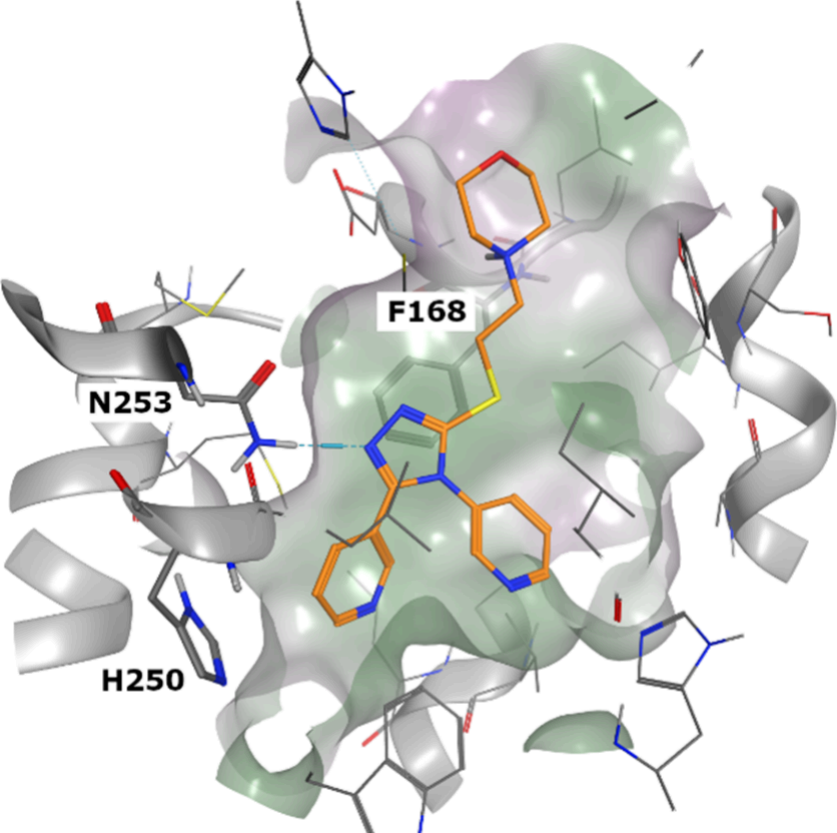
Docking pose of compound **4** (orange) at A_2A_ AR (PDB ID: 4EIY). The protein is represented as a gray ribbon with key residues displayed as sticks. Hydrogen bond interactions are highlighted in light blue. The surface of the receptor is colored according to lipophilicity (from green (lipophilic) to pink (hydrophilic)).

### Alternative Filtering of Generated Molecules

To determine whether the failure was due to the filtering process rather than an inadequate generation phase, an alternative sequence and type of filters were applied to the initial 365,321 molecules obtained after KNIME refinement (Figure 4).

**Figure 4:**
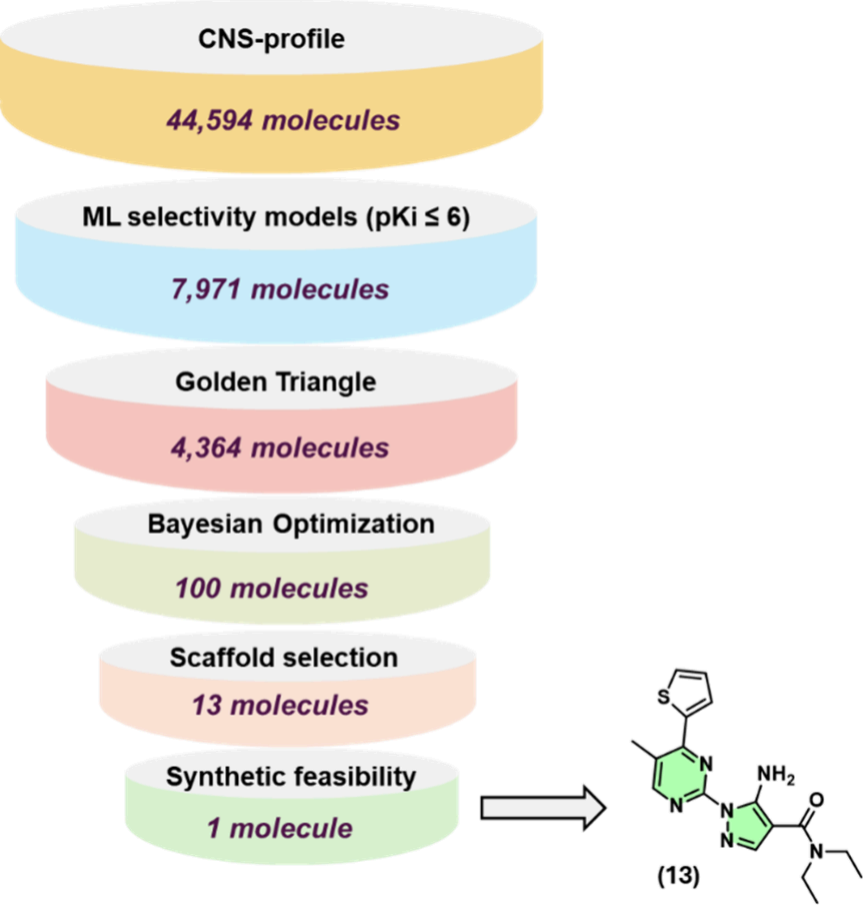
Alternative filtering of generated molecules.

In particular, the filter based on the CNS-MPO score was applied first since it allows one to drastically reduce the number of selected molecules to 44,594. It was followed by an assessment of selectivity against the other three adenosine receptor subtypes, which further restricted the selection to 7971 molecules. At this stage, 215 molecules were discarded due to the presence of the “inactive” 1,2,4-triazole based on the earlier findings. The remaining ones were filtered according to the Golden Triangle criteria.^71^ By plotting molecular weight versus calculated cLogD at a pH value of 7.4, only 4364 compounds that fell within the optimal physicochemical space for metabolic clearance and oral absorption survived for the next filter. At this stage, to prioritize the synthesis of the most promising molecules in terms of A_2A_ antagonistic activity, a Bayesian optimization (BO) analysis was performed.^72–76^ BO is a supervised learning approach in which data–label pairs are used to train a surrogate model, typically a Gaussian process model. Specifically, a command-line interface tool developed by Evotec was used to rank the 4364 filtered compounds. The ranking was based on Continuous and Data-Driven Descriptors (CDDDs)^77^ and a reference data set of 5934 A_2A_ AR antagonists with known pKi values. The top 100 ranked compounds were then selected for further evaluation (Table S6).

In particular, these top-ranking molecules were compared against the 438 ones passing the filters for activity, selectivity, and CNS profile and obtained from the first filtering process (Figure 4) and excluding also in this case structures bearing a 1,2,4-triazole. By the analysis of the dominant cores shared between the two subsets using the automatic SAR analysis function within DataWarrior (DW), we identified the highly represented central ring system.^78^ This systematic comparison facilitated the selection of 13 high-priority molecules for chemical synthesis (Figure S5). Among these molecules, the pyrazolo-pyrimidine (**13**) containing an exocyclic free amino group, potentially suitable for the formation of a bidentate H-bond with Asn253, was selected for the synthesis even considering its synthetic feasibility. The compound was not selected from the molecular modeling filtering pipeline because in its predicted docking poses, it lacks both of the H-bond with Asn253.

This molecule (Scheme S2), together with the two intermediates of the synthesis containing an ethyl ester (**14**, Table 1) or a carboxylic acid (**15**, Table 1) in the lateral chain instead of diethyl amide, was evaluated for its affinity toward hA_2A_ AR.

**Table 1:**
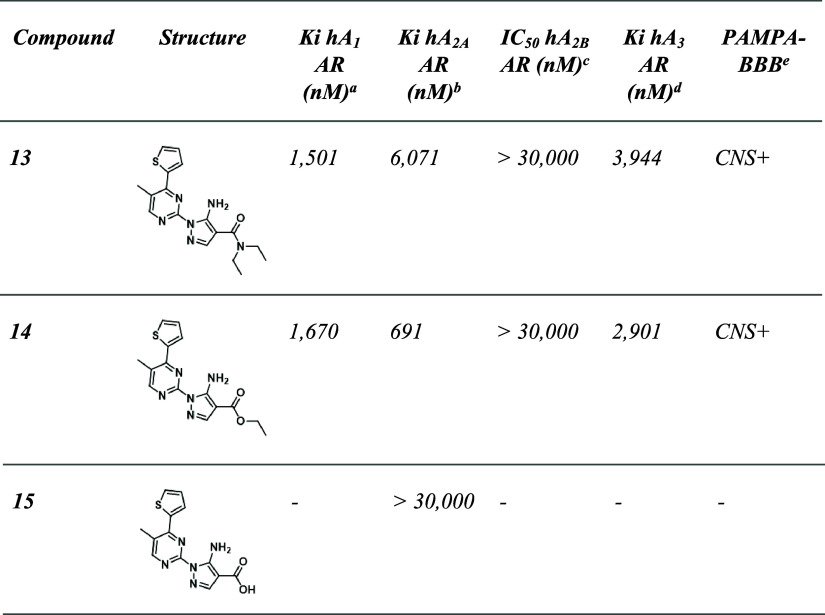
Binding Affinities at A_1_, A_2A_, and A_3_ ARs and Activity toward A_2B_ AR for Compounds **13**–**15** and Evaluation of Apparent Passive Permeation through BBB

a Displacement of specific [^3^H]­CCPA binding at human A_1_ AR expressed in Chinese hamster ovary cells (CHO).

b Displacement of specific [^3^H]­NECA binding at human A_2A_ AR expressed in CHO cells.

c IC_50_ values of the inhibition of NECA-stimulated adenylyl cyclase activity in CHO cells expressing hA_2B_AR.

d Displacement of specific [^3^H]­HEMADO binding at human A_3_ AR expressed in CHO cells.

e Parallel artificial membrane permeability assay: CNS+ means that the compound has been predicted to pass the BBB (details are reported in the SI).

The REINVENT generated compound (**13**) proved to be effectively active against hA_2A_ AR, showing a *K*_i_ value of 6071 nM (*K*_i_ hA_1_ AR 1501 nM, IC_50_ hA_2B_ AR > 30,000 nM, *K*_i_ hA_3_ AR 3944 nM). Interestingly, ethyl ester is more potent at the target than **13**, displaying a *K*_i_ of 691 nM, while carboxylic acid is inactive at a concentration of 30 μM. To exploit if the CNS parameter in the scoring function and/or in the filters worked properly, compounds **13** and **14** were evaluated for their ability to pass the BBB through a parallel artificial membrane permeability assay (PAMPA)^79–81^ using a porcine brain lipid solution to generate the membrane. From the obtained apparent permeability value (Figure S6 and Tables S7 and S8), compound **13** was predicted to permeate the BBB, confirming the strength of the CNS components in our workflow.

The second pipeline led to promising results, represented by the identification of the noteworthy hit compound **13**. Despite its affinity for the target remaining in the micromolar range, these findings indicate that further refinement, particularly during the generative phase, may be required to enhance the target binding.

### Second Scoring Function

To better understand the role of the scoring function, a second run of REINVENT was performed. The scoring logic was modified to prioritize structure-based insights and address the failures of the first run. Assuming the importance of forming a bidentate H-bond with Asn253, in this second run, the absence of this interaction was introduced as a penalty in the score attributed to the molecule. Additionally, the structure-based reward was tightened by applying an ascending sigmoidal transformation to the GOLD Scores with a more stringent range of 55–65. The weights of the three components of the scoring function were also modified by increasing the weight of the 3D structural component, to 5 or 10, while maintaining a weight of 1 for the CNS profile and QSAR models. Noteworthily, an alternative strategy recently emerging applies Pareto-based multiobjective optimization as an alternative to the weighted mean scoring functions used in *de novo* design frameworks such as REINVENT, primarily to better manage trade-offs among multiple objectives.^82–84^ In this work, we adopt a different perspective by employing REINVENT largely as is, with the aim of critically assessing both the strengths and limitations of its generative stage and postgenerative filtering through a comprehensive validation pipeline, extending to synthesis and *in vitro* evaluation. Furthermore, the QSAR activity models were updated and retrained with the incorporation of the new data coming from the inactive 1,2,4-triazoles previously synthesized to steer the agent away from unproductive chemical space (Tables S4 and S5). Two independent cycles of 1000 iterations were performed (using the two different weights for the 3D structural component); the first (weight 5) yielded 57,847 molecules, and the second (weight 10) produced 59,562 molecules, all maintaining a total average score >0.6. The resulting library of 117,409 compounds was processed through the identical KNIME refinement protocol used previously, with a minimal further reduction of molecules, 117,386 in total.

### Filtering of the Second Run

For the identification of the most promising molecules in this second round, we implemented a streamlined strategy focused on the QSAR activity model, CNS profile, key pharmacophoric interactions, and synthetic feasibility (Figure 5).

**Figure 5:**
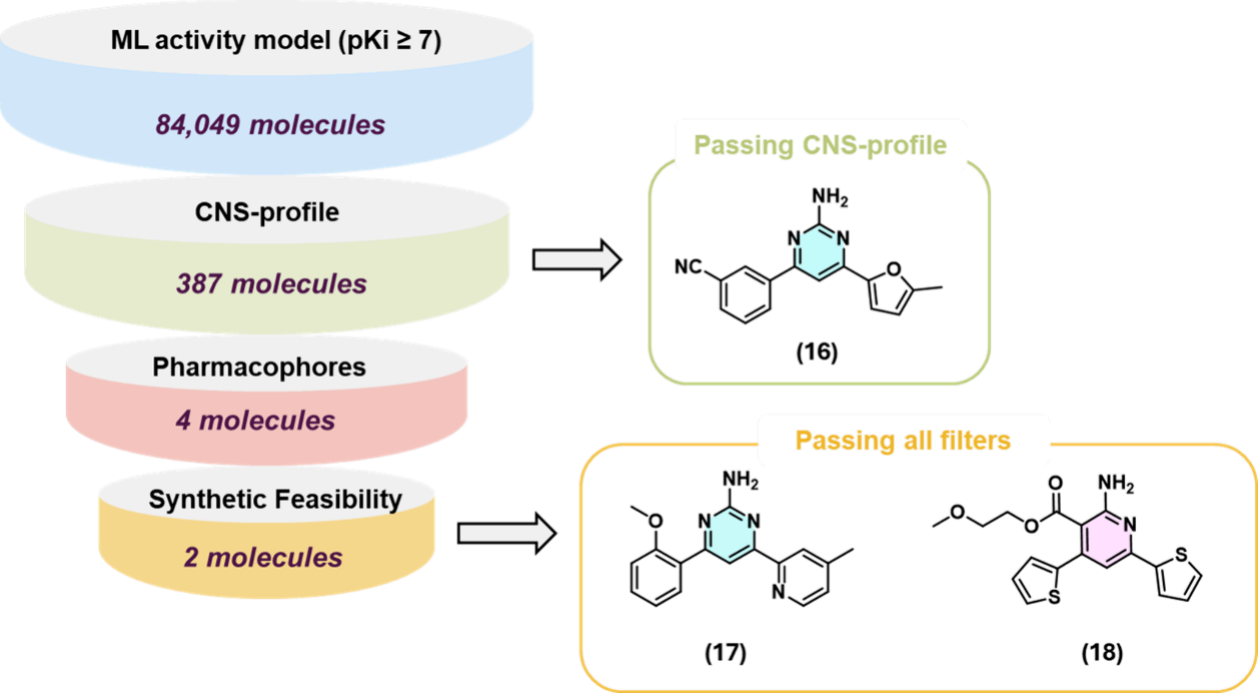
Filtering of generated molecules and selected molecules **16**–**18** from the second run of REINVENT.

The initial 117,386 molecules were evaluated for the predicted activity at the target by using the same QSAR model present in the scoring function and keeping only molecules with a predicted pKi > 7. The 84,049 remaining molecules were evaluated for CNS-like properties using the CNS-MPO and BBB score^51,66^ and the same cutoff values used for the first run. As expected, a strong reduction in the total number of molecules was obtained with 387 molecules surviving such criteria, which were docked at the A_2A_ AR (PDB: 4EIY, https://www.rcsb.org/) using the Gold software, resulting in 4530 poses. These poses were subjected to a multistep structure-based filtering protocol based on the previously defined pharmacophores P1 and P2 and the essential Asn253 interaction: (1) application of the first pharmacophoric filter (P1) narrowed the selection to 1370 poses; (2) poses with at least one H-bond with Asn253 were selected, resulting in 562 poses; (3) poses with unfavorable (positive) electrostatic and/or van der Waals interaction energies were discarded, leaving 486 poses; (4) the presence of a bidentate hydrogen bond with Asn253 was assessed, and this further reduced the number to 22 poses; and (5) application of the second pharmacophoric filter (P2) resulted in 11 poses, corresponding to five unique molecules. Following the exclusion of one molecule due to tautomeric and stereochemistry considerations, four molecules (**17**–**20**), reported in Figure 6, were prioritized for further investigation.

**Figure 6:**
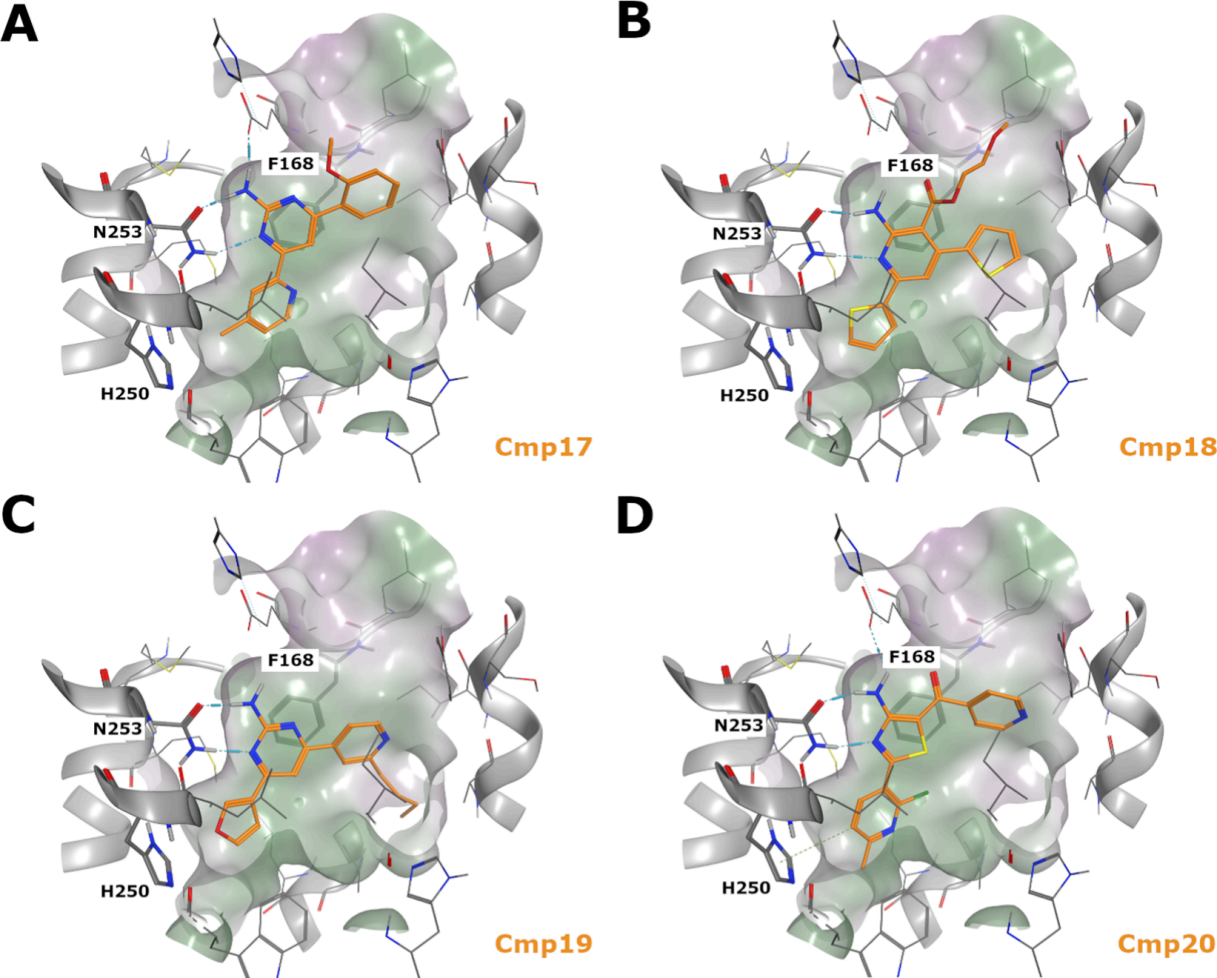
Binding modes of representative compounds docked into the active site of the target protein A_2A_ AR (PDB ID: 4EIY). (A) Compound **17**, (B) compound **18**, (C) compound **19**, and (D) compound **20**. Ligands are shown in orange, with hydrogen bond interactions highlighted in light blue. The protein is represented as a gray ribbon with key residues displayed as sticks. The surface of the receptor is colored according to lipophilicity (from green (lipophilic) to pink (hydrophilic)).

The docking poses of these molecules (**17–20**, Figure 6) revealed their ability to form a bidentate hydrogen bond with Asn253. This interaction is facilitated by the close proximity of the free amino group and the endocyclic nitrogen in amino-pyrimidine, pyridine, and thiazole rings. Moreover, the central aromatic core is engaged in a π–π interaction with Phe168, and an aryl substituent is placed in proximity to His250. Based on synthetic feasibility, we prioritize the 2-aminopyrimidine **17** and 2-aminopyridine **18**. Compound **16** that did not pass all the pharmacophoric filters was also retained and considered for the synthesis, as a structural analog of **17** and, for this reason, a valuable point of comparison for the SAR analysis.

### Synthesis and Pharmacological Characterization of Selected Compounds of the Second Run (**16**–**18**)

The synthesis of compounds **16**–**18** is reported in Schemes S3–S5. Affinities for these compounds are reported in Table 2, together with the apparent passive permeation of BBB through the *in vitro* PAMPA assay.

**Table 2:**
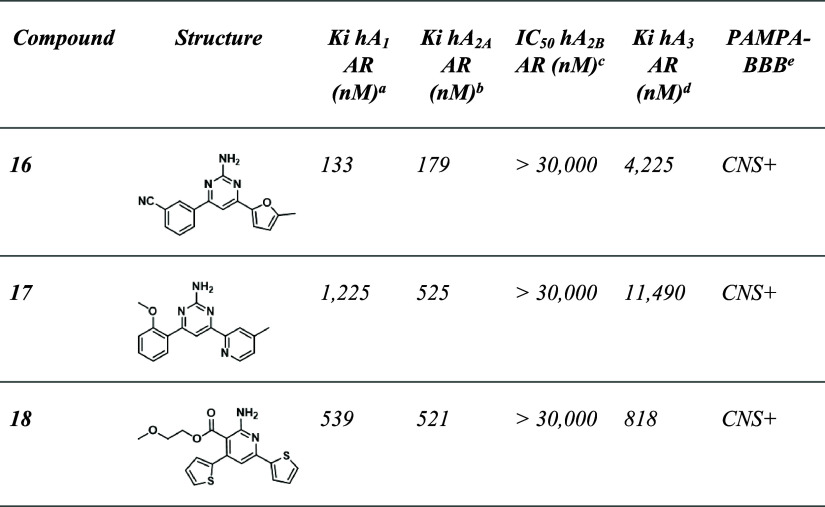
Binding Affinities at A_1_, A_2A_, and A_3_ ARs and Activity toward A_2B_ AR for Compounds **16**–**18** and Evaluation of Apparent Passive Permeation through BBB

a Displacement of specific [^3^H]­CCPA binding at human A_1_ AR expressed in CHO cells;

b Displacement of specific [^3^H]­NECA binding at human A_2A_ AR expressed in CHO cells.

c IC_50_ values were obtained inducing an increase of cAMP accumulation in CHO cells expressing hA_2B_ AR.

d Displacement of specific [^3^H]­HEMADO binding at human A_3_ AR.

e Parallel artificial membrane permeability assay: CNS+ means that the compound has been predicted to pass the BBB (details are reported in the SI).

The second run was entirely successful, with the selected compounds demonstrating binding affinity to the hA_2A_ AR in the high nanomolar to low micromolar range (179–525 nM).

Furthermore, the selectivity profile against the other AR subtypes showed that these compounds were generally nonselective toward hA_1_ AR and hA_3_ AR, particularly against hA_1_ AR. In contrast, they exhibited a favorable selectivity profile against hA_2B_ AR. Notably, in this second run, selectivity filters were not applied; therefore, the observed lack of selectivity was unsurprising. All compounds (**16–18**) were predicted to cross the BBB in the PAMPA-BBB assay (Table 2 and Table S8), confirming again the strength of considering the CNS profile both in the scoring function and in the filtering process.

To have a clear idea of the exploration of the 2-amino pyrimidine scaffold for the target receptor, an evaluation of the IP space for the nucleus both on the starting data set and in the literature was performed. The analysis on the starting data set was made by using DataWarrior (DW) and led to the identification of 106 molecules sharing the 2-amino pyrimidines core; however, most of them were tetrasubstituted and not trisubstituted as in our compounds (**16**–**17**, **19**). Among the 106 molecules, only two were found to be very similar to the generated compounds and were also reported by van Veldhoven and colleagues as A_1_ AR antagonists.^85^ In addition, other studies reported in the literature on biaryl derivatives seem to suggest a nonselective antagonism for the target.^20,22,86–92^ It is significant also that etrumadenant is a biaryl 2-amino pyrimidine and dual A_2A_ AR/A_2B_ AR antagonist currently in phase Ib/II for metastatic colorectal cancer.^86–89^ Such molecule was not present in the training data set, but it should be highlighted how a generative approach like REINVENT in our study was able to generate molecules very close to a clinical candidate even if only two similar molecules were present in the database. Consequently, compounds **16**–**18** were very important to us to be synthesized to determine their AR affinity and selectivity profiles.

Thus, among the experimentally validated hits obtained from both generative runs, only compound **13** represents a novel chemotype. Although additional distinct and potentially novel chemotypes were identified at the final filtering stage in both runs (Figures S4 and S5 and Figure 6), these were not pursued for synthesis due to the limited literature support for feasible synthetic routes and are therefore left for future investigation.

Table 3 summarizes the progression in success rate and potency of the synthesized compounds throughout our experimentation. Run 1 (standard) represents the baseline generative model (QSAR, CNS-MPO, and 3D/Gold) where compounds were selected using standard filtering criteria. All nine synthesized compounds were inactive due to the lack of critical bidentate hydrogen bonds with Asn253. Utilizing the same raw data from the baseline generative run, an alternative postprocessing strategy (including BO) was applied. This allowed for the identification of micromolar compound **13**. In this case, compounds **14** and **15** were not considered because they are intermediate compounds not directly generated by the model; thus, in this case, the success rate is 100%. Finally, in run 2, the generative model was improved by integrating a specific structural penalty to enforce the Asn253 interaction directly within the reinforcement learning loop. This “structural steering” significantly increased the quality of the generated chemical space, resulting in a 100% experimental success rate and an improvement in compounds’ affinity, with Ki values toward A_2A_ AR in the high nanomolar range.

**Table 3:** Quantitative Comparison of Generative Performance and Postprocessing Strategies

	**run 1** (standard)	**run 1** (alt. filtering)	**run 2** (optimized model)
Asn253 H-bond	no	no	enforced
compounds synthesized	9 (**4–12**)	1 (**13**)	3 (**16–18**)
active compounds	0	1	3
success rate (per run)	0%	100%	100%
best potency	inactive	6.07 μM	179 nM

## Conclusions

In summary, we implemented a *de novo* design strategy using the tool REINVENT to discover new A_2A_ AR antagonists targeting the CNS, ensuring that both the pharmacodynamic potency and pharmacokinetic viability were prioritized as part of the generative process. To achieve this, a multiparameter scoring function was developed combining three components: structure insights of the target, ligand-based data from published actives, and pharmacokinetic considerations concerning BBB permeability. Initially, all scoring components were weighed equally, leading to the identification of 1,2,4-triazole derivatives, which unfortunately resulted in being inactive at the target. To address this outcome, an alternative filtering pipeline was explored using Bayesian optimization as a machine learning approach to identify hA_2A_ AR hits, leading to a micromolar compound (**13**). On the other hand, the inactivity of the 1,2,4-triazole derivatives was attributed to the absence of a bidentate hydrogen bond donor capable of interacting with Asn253, a key residue for antagonism. To address this, a revised scoring function was implemented where the 3D component was emphasized by increasing its weight and also including, as a pharmacophoric constraint, the Asn253 interaction. Interestingly, this approach successfully led to the identification of three active compounds: **16**, **17**, and **18**.

Such results highlight the importance of both scoring functions and selection filtering pipelines. Indeed, applying different filtering strategies to the same pool of generated molecules resulted in completely different outcomes: the initial strategy failed to identify A_2A_ AR antagonists (i.e., inactive 1,2,4-triazoles, **4–12**), while the alternative strategy successfully identified a hit compound (**13**). The comparison between the first and second runs highlights the importance of the pharmacophoric 3D constraints to focus on molecules that can form specific interactions with the target. Finally, this study demonstrates that strategic weighting of REINVENT’s scoring components and fine-tuning postprocessing through the filtering pipeline are essential for prioritizing diverse chemotypes and introduce novel scaffolds. Tanimoto similarity (Table S9) analysis confirmed these trends: compounds from the first run showed similarity values below 0.61 across all reference databases. In contrast, compounds from the second run showed higher similarity scores, suggesting that, as a sort of trade-off, the introduction of specific binding constraints focused the generative process on more established chemical motifs. A_2A_ AR is a well-studied target; consequently, several antagonists for this receptor and multiple crystal structures of the protein are available. Based on our findings, successful generative *de novo* design still requires the incorporation of target-specific structural constraints. A major limitation, however, is that while these methodologies are very effective for data-rich targets, they are limited for less characterized or “orphan” targets. Such targets are precisely those for which computational *de novo* approaches would be most valuable. Ultimately, this work adds valuable knowledge to a rapidly evolving field of AI-driven drug discovery. It is anticipated that as these methodologies mature, new strategies will emerge to mitigate current limitations and expand the utility toward less characterized chemical and biological spaces.

## Experimental Section

### Computational Studies

#### Data Curation

Data curation was carried out using the KNIME workflow system (version 4.7.2), a platform built on a graphical user interface and composed of interconnected processing nodes that exchange data. The workflow consists of three main stages: compound preparation and standardization using ChemAxon and RDKit, which include cleaning structures, neutralizing molecules, and generating the most probable tautomer;^93^ data set refinement by removing incomplete or unsuitable entries, aggregating data by assay type and potency measure, converting values to a logarithmic scale, and eliminating duplicates while retaining the most active compound; and file generation in SDF or CSV format, ready for machine learning applications.

The starting data points available for each target were 9237 for the A_2A_ AR antagonist, 5313 for the A_1_ AR antagonist, 5136 for the A_3_ AR antagonist, and 4037 for the A_2B_ AR antagonist, respectively.

#### Machine Learning (ML) Model Generation

First run: Classification and regression models for predicting binding to four different adenosine antagonists were trained on the curated data sets using an automated ML model building pipeline developed at Evotec. Multiple model configurations were evaluated by varying (i) activity thresholds (pKi/pIC_50_ = 6, 7, and 8) in classification models; (ii) two- versus three-class labeling schemes, with the latter introducing an intermediate “medium-activity” uncertainty zone; and (iii) combinations of activity types (binding vs functional, Ki vs IC_50_) for both regression and classification models. Compounds were represented using MACCS keys along with 18 molecular properties (TPSA, ALogP, Molecular Weight, FSP3, Heavy Atom Count, Atom Count, HBD, Aromatic Heterocycles Count, Heteroatom Count, Rotable Bond Count, Aromatic Ring Count, Aromatic Carbocycles Count, Aliphatic Heterocycles Count, Aliphatic Ring Count, Saturated Heterocycles Count, Saturated Ring Count, Aliphatic Carbocycles Count, and Saturated Carbocycles Count), all calculated using RDKit v. 2021.3.5 (https://www.rdkit.org). For every data set, both random forest (RF) and support vector machine (SVM) models were trained using the scikit-learn v. 0.21.3 library in Python,^94^ and the model achieving the highest performance in fivefold cross-validation (CV) was retained. Hyperparameters for each model were optimized using grid search on the training folds. Models were further validated against an external test set comprising 74 molecules extracted from ChEMBL (Table S1). Tables S2 and S3 summarize the constructed ML models and the corresponding CV performance metrics.

Second run: After the synthesis and biological evaluation of 11 compounds from the first run of REINVENT, additional ML models were built using an alternative workflow developed at Evotec^11^ based on RDKit v. 2021.03.1 and pycaret v. 2.3.10 (an open-source, low-code machine learning library in Python. 2020. https://www.pycaret.org), a python library for automated ML. The workflow comprised descriptor calculation, train-test splitting (an 85:15 random split for regression tasks and a stratified random split for classification tasks), feature selection, model selection and hyperparameter optimization using 10-fold CV on the training set, model evaluation on the held-out test set, and final retraining on the full set. In this second modeling round, compounds were represented using a concatenation of molecular fingerprints and descriptors, namely, Morgan fingerprints (2048 bits), Atom Pair fingerprints (1024 bits), Topological RDKit fingerprints (1024 bits), MACCS keys (167 bits), and RDKit molecular descriptors (208 molecular, structural, physicochemical, fragment-based, and topological properties). Modeled end points and performance metrics are reported in Tables S4 and S5.

#### *De Novo* Design

REINVENT is a generative AI tool for *de novo* drug design, a generative model based on a recurrent neural network coupled with a reinforcement learning loop.^49,50,95^ The main feature of REINVENT is the ability to generate new molecules with optimal values for a set of desired properties: it generates new molecules while performing multiparameter optimization (MPO). To force the tool to explore different areas in the chemical space, REINVENT includes diversity filters ensuring that the model explores a wide range of chemical space rather than getting confined to a narrow region. It is also possible to use known good molecules as inception to help the model to begin its exploration from a proven, effective chemical space. A custom Evotec version of the public tool, based on version 3.2 of REINVENT (https://github.com/MolecularAI/Reinvent), has been used starting from the Python Notebook coming from AstraZeneca. Additionally Augmented Hill Climb,^96^ an alternative algorithm for faster MPO not present in the original REINVENT, has been implemented as the default reinforcement learning strategy in the Evotec version and has been exploited in the second generative run. For each run, the three literature-reported molecules shown in Figure S1 have been used as inception, and a diversity filter based on identical Murcko scaffolds with a minimum similarity of 0.4 has been applied. Details on the setup are given in Table S10.

#### Idea Postprocessing

All the ideas generated from the two runs of REINVENT, 365,577 with the first scoring function and 117,409 for the second one, have been submitted, separately, to a KNIME workflow (KNIME 4.7.2)^93^ for the postprocessing step. The workflow included the preparation and standardization of structures using ChemAxon and RDKit nodes; removal of salts, fragments, and solvents; neutralization; stereochemistry clearing; and tautomer generation. Refinement steps merged duplicates and excluded compounds that were already present in collected databases. Physicochemical properties (e.g., MW, cLogD, and tPSA) and Tanimoto similarity were then calculated followed by clustering based on fingerprint similarity. Finally, output files in SDF or CSV format were generated for machine learning predictions.

At the end of this process, 365,321 new ideas from the first run and 117,386 new ideas from the second one have been obtained. The antagonist target activity for various adenosine receptors (A_2A_, A_2B_, A_3_, and A_1_ ARs) has been predicted for all the newly generated ideas using the ML models described above and a command-line interface (CLI).

#### Ligand Library Preparation for Docking

The 438 molecules filtered by predicted A_2A_ AR activity by the QSAR model, predicted selectivity profile, consistency between regression models (predicted A_2A_ AR activity thresholds), and CNS profile and the 387 molecules filtered from the second run for predicted A_2A_ AR activity and CNS profile were prepared for the following molecular docking studies. The OpenEye Suite (http://www.eyesopen.com) was employed, in particular, the tool “Tautomers” was used for the generation of the most probable tautomeric states of the molecules, the tool “Omega” was used to generate a tridimensional structure for the molecule, generating multiple stereoisomers when not univocally assigned, and the tool “Fixpka” was used for the assignment of the most favorable ionization state at pH ∼7.4. This process was repeated for both the runs by identifying 505 structures starting from the 438 molecules of the first run and 453 molecules starting from the 387 molecules of the second run.

#### Protein Preparation for Docking

The A_2A_ AR structure employed for molecular docking was the X-ray antagonist-bound structure labeled 4EIY according to the RCSB PDB. The protein structure was prepared using the MOE2022 suite.^97^ The fusion protein apocytochrome b(562)­RIL, replacing intracellular loop 3 (ICL3), was removed, and then ICL3 (residues Lys209–Gly218) was reconstructed with the Loop Modeler tool of the MOE suite using a knowledge-based approach. The Structure Preparation tool was used for the reconstruction of missing atoms, capping of N-/C-termini with acetyl and *N*-methyl amide groups, and management of alternate states (with retention of the one with higher occupancy). Hydrogens were added using the Protonate3D tool, with assignment of most probable protonation states at pH ∼7.4. The added hydrogens were minimized using the Amber14:EHT force field. The crystallographic sodium present as a negative allosteric modulator in the structure was retained, together with three water molecules of its sphere of solvation.

#### Molecular Docking

The prepared ligand databases were subjected to a molecular-docking-based virtual screening. The GOLD docking algorithm was employed^57^ using Gold Score as the scoring function. Ten poses per compound were generated, and the conformational exploration was confined to an 11 Å radius sphere, centered on the center of mass of key residues Asn253 and Phe168.

#### Pharmacophore Model

A pharmacophore model was built using the antagonist-bound A_2A_ AR X-ray structures available in the PDB database (accessed on May 2023), consisting of structures with PDB ID: 3EML, 3PWH, 3REY, 3RFM, 3UZA, 3UZC, 3VG9, 3VGA, 4EIY, 5IU4, 5IU7, 5IU8, 5IUA, 5IUB, 5JTB, 5K2A, 5K2B, 5K2C, 5K2D, 5MZJ, 5MZP, 5N2R, 5NLX, 5NM2, 5NM4, 5OLG, 5OLH, 5OLO, 5OLV, 5OLZ, 5OM1, 5OM4, 5UIG, 5UVI, 5VRA, 6AQF, 6GT3, 6JZH, 6LPJ, 6LPK, 6LPL, 6MH8, 6PS7, 6S0L, 6S0Q, 6WQA, 6ZDR, 6ZDV, 7PX4, 7PYR, 7RM5, 7T32, 8CU6, 8CU7, 8DU3, 8FYN, 8GNE, and 8GNG. A consensus pharmacophore model was built with MOE2022 Pharmacophore Editor functionalities, adjusting the selection of pharmacophoric features and dimensions of the pharmacophoric spheres in a way to include most of the crystallographic antagonists. Thus, three different pharmacophore hypotheses were built, taking into account the following features: (a) The first pharmacophore was prepared including an essential aromatic/hydrophobic feature of 1.6 Å (overlapped to the aromatic scaffolds involved in a π–π interaction with Phe168 in most of the antagonists) and at least one hydrogen bond acceptor feature (1.4 Å radius) or one hydrogen bond donor/acceptor feature (1.54 Å radius) in proximity of Asn253 (Figure S3A). (b) The second pharmacophore was prepared including an additional feature to those mentioned above, that is, an additional 2 Å aromatic/hydrophobic feature in proximity of His250 (Figure S3B). (c) The third pharmacophore was prepared including a hydrogen-bond acceptor sphere of radius 1.7 Å centered on the pyranic oxygen of chromone 4d (PDB ID: 6ZDR). Basing on the listed pharmacophoric filters, compounds contained in the sets derived from the two runs of REINVENT were filtered to identify only the most promising ones in terms of positioning in the binding pocket (Figure S3C).

#### Ligand–Receptor Interaction Analysis in Docking

The PLIF tool of the MOE2022 suite was employed for the calculation of ligand–receptor fingerprint interactions by focusing just on interactions satisfying the default “strong” energetic thresholds. The ligand–protein electrostatic and van der Waals interactions were computed using an “svl” script within the MOE2022 platform; in particular, PM3 partial charges were assigned to the docking poses, and Amber14:EHT partial charges were assigned to the protein atoms.

#### Bayesian Optimization (BO)

BO is based on a supervised learning approach where data–label pairs are available to train a central ML model (surrogate model). The trained surrogate model, generally a Gaussian process model, provides as prediction a mean and a standard deviation. Both predictions are used by an acquisition function that is either minimized to generate a new point to test or used to score and rank existing points with unknown values. These points are the most informative for the model and should be tested to provide the maximum information for the project in the next round of optimization.^77^ A CL interface tool developed by Evotec has been used for the ranking of the 4364 filtered compounds by using the following setting parameters: BO as model, CDDD as descriptors, a data set of 5934 compounds with known pKi A_2A_AR antagonist activity as known input file, EI (Expected Improvement) as acquisition function, an acquisition jitter of 0.01, A_2A_ antagonist pKi as objective, and botorch as Gaussian Process Engine. The 100 molecules with the best ranking are represented in Table S6. The 13 molecules selected for visual inspection of ranking value for BO analysis are depicted in Figure S5.

### Chemistry

#### General Chemistry Experiment and Information

Reagents used in the synthesis were purchased from commercial suppliers and directly used for the synthesis without any further purification. The completion of the reaction was monitored by UPLC–MS (Analytical UPLC–MS (ES+/ES−) 2.0 min method Acquity UPLC coupled with a QDA or SQD2 mass Spectrometer with a range 210–350 nm and an acquisition rate of 40 Hz) or/and thin layer chromatography (TLC) by using precoated silica gel plates on aluminum sheets (ALUGRAM, Macherey-Nagel, 60F UV254). When expressed, TLC was revealed with an acid solution of KMnO_4_ (1 g KMnO_4_/100 mL H_2_O + 10 mL H_2_SO_4_ 10%). The UPLC–MS was registered with two different instruments with proper columns: Kintex EVO C18 (1.7 μm, 2.1 × 50 mm) at 40 °C or Acquity UPLC CSH C18 (1.7 μm, 2.1 × 50 mm, 130 Å) at 40 °C, with basic or acid methods (2 min, flow = 0.9 mL/min). LC–MS basic method: solvents = 10 mM ammonium bicarbonate aqueous solution adjusted to pH 10 with ammonia (A)/ACN (B); gradient = 0.0 min 97% (A), 3% (B); 1.4 min 0.1% (A), 99.9% (B), 1.9 min 0.1% (A), 99.9% (B); 2.0 min 97% (A), 3% (B). LC–MS acid method: solvents = H_2_O + 0.1% HCOOH (A)/ACN + 0.1% HCOOH (B); gradient = 0.0 min 97% (A), 3% (B); 1.4 min 0.1% (A), 99.9% (B), 1.9 min 0.1% (A), 99.9% (B); 2.0 min 97% (A), 3% (B). Final compounds and intermediates were purified in two different ways, i.e., by flash chromatography (FC) using a chromatographic column and a stationary phase silica gel (Macherey-Nagel, silica 60, 240–400 mesh) or by Isolera One (Biotage) on Sfär Silica Cartridges Duo 60 μm or Sfär C18 Cartridges Duo 100 Å 30 μm containing different quantities of the stationary phase (from 5 to 60 g). The time used for elution is given as CV = column volumes of the more polar solvent and 1 CV = 0.26 s = 15 mL of solvent (with the proper ratio). Unless otherwise stated, purification was performed by column chromatography rather than using the Isolera One system. Some of the compounds were purified with HyperSep SCX cartridges (chemical: benzenesulfonic acid, silica irregular particles 40–63 μm, 60 Å), by eluting three times with MeOH and three times with NH_3_ 1 M in MeOH. When used, light petroleum ether (PetEt) refers to the fractions boiling at 40–60 °C and NH_4_OH to a solution 28–30% in water. The ^1^H NMR and ^13^C NMR were recorded in DMSO-*d*_6_ or CD_3_OD by using Varian 400 MHz or Varian 500 MHz spectrometers (101 MHz, 126 MHz for ^13^C NMR, respectively) or Bruker 400 or 500 MHz. Bidimensional spectra, HSQC and HMBC, were registered with Bruker 400 or 500 MHz and, when the ^13^C NMR was not registered, were used to derive the carbon signals of each molecule. Chemical shifts (δ scale) are reported in parts per million (ppm) by setting as a reference the solvent peak. The multiplicity of signals is given as s (singlet), d (doublet), dd (doublet of doublets), ddd (doublet of doublets of doublets), dt (doublet of triplets), t (triplet), and m (multiplet), and coupling constants (*J*) are given in hertz. ESI-MS analysis was performed by using an LTQ-XL spectrometer, or UPLC–MS (explicated in the previous rows) and HRMS were registered with Exploris 240. Compounds analyzed with ESI-MS or HRMS were prepared by solubilizing 1 mg of compound in 1 mL of MeOH. Purities were determined on the final derivatives by analytic HPLC on a SHIMADZU CBM-20A instrument by using as a Gemini 5 μm NX-C18 column (Phenomenex) or by UPLC–MS with the methods indicated (acid/basic). The detector was set at 254 nm. Solutions used for the evaluation of the purity were prepared by solubilizing 1 mg of compounds in 1 mL of MeOH/H_2_O 1:1. Three different methods were used (HPLC): method A: H_2_O/MeOH in 20 min, from 20% to 45% of MeOH (1 mL/min); method B: H_2_O/MeOH in 20 min, from 30% to 45% of MeOH (1 mL/min); and method C: H_2_O/MeOH in 20 min, from 70% to 95% of MeOH (1 mL/min). The purity of all final compounds is >95%. Melting points for solid compounds were determined with a Stuart SMP10 melting point apparatus.

#### General Synthesis of 2,5-Biaryl-4*H*-1,2,4-triazole-3-thiol (**21**–**22**)

A solution of the proper isothiocyanate (**23**–**24**) (7.30 mmol, 1.0 equiv) and proper hydrazide (**25**–**26**) (7.30 mmol, 1.0 equiv) in 12 mL of EtOH was refluxed for 30 min. After this time, the reaction was checked with UPLC–MS analysis (data not reported) and TLC (DCM/MeOH 9:1). Then the solvent was removed under reduced pressure, and the solid was diluted with 12 mL of an aqueous solution of 2 M NaOH. The mixture was then refluxed for 3 h. The reaction was checked using UPLC–MS analysis (data not shown) and TLC (DCM/MeOH 9:1). The reaction was cooled to rt, and HCl 37% (conc.) was added until pH ∼5, at which point the product started to precipitate. The precipitate was collected through filtration, and the solid was dried under a vacuum, affording the desired compounds (**21**–**22**).

##### 4,5-Di­(pyridin-3-yl)-4*H*-1,2,4-triazole-3-thiol (**21**)

890 mg, yield = 95%. Pale yellow solid. ^1^H NMR (Bruker 400 MHz, DMSO-*d*_6_) δ 14.38 (s, 1H), 8.66 (dd, *J* = 4.8, 1.4 Hz, 1H), 8.63–8.59 (m, 2H), 8.55 (d, *J* = 1.7 Hz, 1H), 7.95–7.89 (m, 1H), 7.70 (dt, *J* = 8.1, 1.7 Hz, 1H), 7.57 (dd, *J* = 8.1, 4.8 Hz, 1H), 7.45–7.41 (m, 1H). Mass calculated for C_12_H_9_N_5_S = 255.0579 g/mol. UPLC–MS (MeOH): *m*/*z* 256.24 [M + H]^+^, 254.24 [M – H]^−^. Rt. = 0.15 min. Analysis type: LC–MS basic.

##### 4-(4-(2,4-Difluorophenyl)-5-mercapto-4*H*-1,2,4-triazol-3-yl)­benzoic acid (**22**)

1970 mg, yield = 95%. Pale yellow solid. Purity = 100% calculated by LC–MS (acid method) a/a by UV. ^1^H NMR (Bruker 400 MHz, DMSO-*d*_6_) δ 8.46 (s, 1H), 7.81–7.74 (m, 2H), 7.59–7.51 (m, 1H), 7.45–7.38 (m, 1H), 7.23–7.21 (m, 2H). Two protons not visible. Mass calculated for C_15_H_9_F_2_N_3_O_2_S = 333.0384 g/mol. UPLC–MS (MeOH): *m*/*z* 334.17 [M + H]^+^. Rt. = 0.85 min. Analysis type: LC–MS acid method.

#### General Synthesis of the 4-Methyl-5-aryl-4*H*-1,2,4-triazole-3-thiols (**27**–**28**)

To a solution of the proper carboxylic acid (**29**–**30**) (4.06 mmol, 1.0 equiv) and 4-methyl-3- thiosemicarbazide (**31**) (470 mg, 4.47 mmol, 1.1 equiv) in 2.0 mL of DMF, DIPEA (1.27 mL, 7.31 mmol, 1.8 equiv) was added. The reaction was cooled to 0 °C and stirred for 10 min. Then a 50% solution of T3P in EtOAc (3.63 mL, 6.09 mmol, 1.5 equiv) was added dropwise, and the reaction was stirred at rt for 3 h. The reaction was monitored with UPLC–MS analysis (data not reported). Then 6.0 mL of an aqueous solution of 4M NaOH was added until pH = 8. The aqueous phase was washed with EtOAc (1 × 3 mL), and a further 4 M solution of NaOH (4 mL) was added until pH ∼11. The resulting solution was stirred at 70 °C for 40 min. After this time, the reaction was cooled to RT, and HCl 37% (conc.) was added until pH ∼5, at which point the product started to precipitate. The day after, the precipitate was collected through filtration. The solid was dried under a vacuum, affording the desired compounds (**27**–**28**).

##### 4-Methyl-5-(pyridin-4-yl)-4*H*-1,2,4-triazole-3-thiol (**27**)

665 mg, yield = 85%. White solid. ^1^H NMR (Bruker 400 MHz, DMSO-*d*_6_) δ 14.12 (s, 1H), 8.78 (d, *J* = 6.0 Hz, 2H), 7.76 (d, *J* = 6.0 Hz, 2H), 3.60 (s, 3H). Mass calculated for C_8_H_8_N_4_S = 192.0470 g/mol. UPLC–MS (MeOH): *m*/*z* 193.23 [M + H]^+^, 191.13 [M – H]^−^. Rt. = 0.15 min. Analysis type: LC–MS basic.

##### 5-(5-Methoxypyridin-2-yl)-4-methyl-4*H*-1,2,4-triazole-3-thiol (**28**)

829 mg, yield = 71%. Yellow solid. Purity = 100% calculated by LC–MS (acid method) a/a by UV. ^1^H NMR (Bruker 400 MHz, DMSO-*d*_6_) δ 13.95 (s, 1H), 8.44 (d, *J* = 3.0 Hz, 1H), 7.94 (d, *J* = 8.8 Hz, 1H), 7.59 (dd, *J* = 8.8, 3.0 Hz, 1H), 3.91 (s, 3H), 3.80 (s, 3H). Mass calculated for C_9_H_10_N_4_OS = 222.0575 g/mol. UPLC–MS (MeOH): *m*/*z* 223.09 [M + H]^+^. Rt. = 0.71 min. Analysis type: LC–MS acid method.

#### General Synthesis of the 4-Alkyl-5-aryl-4*H*-1,2,4-triazole-3-thiols (**32**–**34**)

A solution of 1,1-thiocarbonyldiimidazole (**35**) (417 mg, 2.34 mmol, 1.3 equiv) and the proper amine chain (**36**–**38**) (1.98 mmol, 1.1 equiv) in 8.0 mL of 2-MeTHF was placed in a sealed microwave vial and stirred for 10 min at rt. After this time, UPLC–MS analysis (data not reported) and TLC (DCM/MeOH 9:1) were performed to see the formation of the intermediates (**39**–**41**). Then, the proper hydrazide (**42**–**44**) (1.8 mmol, 1.0 equiv) was added, and the reaction was irradiated under MW conditions at 130 °C, performing two cycles of 60 min and continuing until the consumption of starting materials revealed with TLC (DCM/MeOH 9:1). After these cycles, the reaction was concentrated under reduced pressure, and the residue was purified by direct phase FC by using specific conditions for each product. The fractions containing the pure product were collected together, and the solvent was removed under reduced pressure to afford the desired compounds (**32**–**34**).

##### 4-(2-(Dimethylamino)­ethyl)-5-(pyridin-3-yl)-4*H*-1,2,4-triazole-3-thiol (**32**)

412 mg, yield = 92%. Yellow solid. Purification on 25 g silica cartridge by Isolera One, using as eluents DCM and a mixture of DCM/MeOH (9:1), by eluting from 5% to 60% of DCM/MeOH (9:1) in 65 CV. ^1^H NMR (Bruker 400 MHz, DMSO-*d*_6_) δ 12.38 (s, 1H), 8.85 (d, *J* = 1.6 Hz, 1H), 8.77 (dd, *J* = 4.9, 1.9 Hz, 1H), 8.15 (dt, *J* = 4.9, 1.6 Hz, 1H), 7.62–7.58 (m, 1H), 4.10 (t, *J* = 6.4 Hz, 2H), 2.39 (t, *J* = 6.4 Hz, 2H), 1.90 (s, 6H). Mass calculated for C_11_H_15_N_5_S = 249.1048 g/mol. UPLC–MS (MeOH): *m*/*z* 250.16 [M + H]^+^. Rt. = 0.18 min. Analysis type: LC–MS acid method.

##### 4-(3-(Dimethylamino)­propyl)-5-(pyridin-4-yl)-4*H*-1,2,4-triazole-3-thiol (**33**)

687 mg, yield = 36%. Yellow solid. Purity = 100% calculated by LC–MS (basic method) a/a by UV. Purification on 60 g silica cartridge by Isolera One, by eluting with DCM and DCM/MeOH (9:1), from 5% to 60% of DCM/MeOH (9:1) in 65 CV. ^1^H NMR (Bruker 400 MHz, DMSO-*d*_6_) δ 8.79 (dd, *J* = 4.4, 1.6 Hz, 2H), 7.73 (dd, *J* = 4.4, 1.7 Hz, 2H), 4.17–4.07 (m, 2H), 2.19 (t, *J* = 6.6 Hz, 2H), 2.03 (s, 6H), 1.78–1.65 (m, 2H). One proton not visible. Mass calculated for C_12_H_17_N_5_S = 263.1205 g/mol. UPLC–MS (MeOH): *m*/*z* 264.25 [M + H]^+^, 262.25 [M – H]^−^. Rt. = 0.34 min. Analysis type: LC–MS basic method.

##### 4-(2-Methoxyethyl)-5-(thiophen-2-yl)-4*H*-1,2,4-triazole-3-thiol (**34**)

440 mg, yield = 52%. Yellow solid. Purity = 100% calculated by LC–MS (acid method) a/a by UV. Purification on 11 g silica cartridge by Isolera One, by eluting with cyclohexane/EtOAc/EtOH (3:1), going from 10% to 80% EtOAc/EtOH (3:1) in 50 CV. ^1^H NMR (Bruker 400 MHz, DMSO-*d*_6_) δ 7.83 (dd, *J* = 5.1, 1.1 Hz, 1H), 7.77 (dd, *J* = 3.7, 1.1 Hz, 1H), 7.25 (dd, *J* = 5.1, 3.7 Hz, 1H), 4.28 (t, *J* = 5.6 Hz, 2H), 3.68 (t, *J* = 5.6 Hz, 2H), 3.17 (s, 3H). One proton not visible. Mass calculated for C_9_H_11_N_3_OS_2_ = 241.0344 g/mol. UPLC–MS (MeOH): *m*/*z* 242.14 [M + H]^+^. Rt. = 0.78 min. Analysis type: LC–MS acid method.

#### General Synthesis for 4-(2-((4,5-Biaryl-4*H*-1,2,4-triazol-3-yl)­thio)­ethyl)­morpholines (**4**, **11b**)

To a solution of the proper thio-1,2,4-triazole intermediate (**21**–**22**) (0.80 mmol, 1.0 equiv) in 8.0 mL of EtOH, 0.22 mL of an aqueous solution of KOH 4 M was added, and the mixture was stirred for 30 min at rt. After this time, 2-chloroethylmorpholine (HCl) (**45**) (1.0–2.0 equiv) was added, and the mixture was refluxed for 3 h. The reaction was checked with UPLC–MS (data not reported). The solvent was removed under reduced pressure, and the solid was purified by FC, with specific conditions for each compound. The solvent was removed under reduced pressure to afford the desired compounds (**4**, **11b**).

##### 4-(2-((4,5-Di­(pyridin-3-yl)-4*H*-1,2,4-triazol-3-yl)­thio)­ethyl)­morpholine (**4**)

65.8 mg, yield = 23%. White solid. Purity = 98% calculated by LC–MS (basic method) a/a by UV. mp = 130 °C. 2-Chloroethylmorpholine (HCl) (149 mg, 0.80 mmol, 1.0 equiv). Purification on 25 g silica cartridge by Isolera One, using as eluents DCM and a mixture of DCM/MeOH (9:1), with a gradient from 5% to 60% of DCM/MeOH (9:1) in 60 CV. ^1^H NMR (Bruker 500 MHz, DMSO-*d*_6_) δ 8.75 (dd, *J* = 4.8, 1.5 Hz, 1H), 8.70 (dd, *J* = 2.5, 0.6 Hz, 1H), 8.60 (dd, *J* = 4.9, 1.6 Hz, 1H), 8.57 (dd, *J* = 2.2, 0.8 Hz, 1H), 8.01 (ddd, *J* = 8.0, 2.5, 1.5 Hz, 1H), 7.74–7.69 (m, 1H), 7.62 (ddd, *J* = 8.1, 4.8, 0.6 Hz, 1H), 7.42 (ddd, *J* = 8.0, 4.9, 0.8 Hz, 1H), 3.55–3.50 (m, 4H), 3.38–3.35 (m, 2H), 2.63 (t, *J* = 6.9 Hz, 2H), 2.40–2.33 (m, 4H). HSQC, HMBC (Bruker 500 MHz, DMSO-*d*_6_) δ 152.87 (C), 152.47 (C), 151.19 (C), 150.93 (CH), 150.74 (C), 150.52 (CH), 148.46 (CH), 148.25 (CH), 135.64 (CH), 135.58 (CH), 124.46 (CH), 123.45 (CH), 65.84 (2 CH_2_), 56.79 (CH_2_), 52.67 (2 CH_2_), 29.64 (CH_2_). Mass calculated for C_18_H_20_N_6_OS = 368.1419 g/mol. UPLC–MS (MeOH): *m*/*z* 369.4 [M + H]^+^. Rt. = 0.56 min. Analysis type: LC–MS basic method. HRMS (ESI-TOF, Exploris 240): experimental *m*/*z* 369.1494 [M + H]^+^, theoretical *m*/*z* 369.1492 [M + H]^+^. Δ = 0.0002.

##### 4-(4-(2,4-Difluorophenyl)-5-((2-morpholinoethyl)­thio)-4*H*-1,2,4-triazol-3-yl)­benzoic Acid (**11b**)

15.0 mg, yield = 5%. Colorless oil. Purity = 100% calculated by LC–MS (acid method) a/a by UV. 2- Chloroethylmorpholine (HCl) (298 mg, 1.60 mmol, 2.0 equiv). Purification on 11 g C18 cartridge by Isolera One, by eluting with ACN + 0.1% formic acid and H_2_O + 0.1% formic acid, going from 5% to 60% ACN + 0.1% formic acid in 65 CV. ^1^H NMR (Bruker 500 MHz, CD_3_OD) δ 8.08–8.00 (m, 2H), 7.73–7.60 (m, 1H), 7.59–7.49 (m, 2H), 7.36–7.27 (m, 1H), 7.27–7.20 (m, 1H), 4.09–3.86 (m, 4H), 3.72–3.60 (m, 4H), 3.30 (dt, *J* = 3.3, 1.6 Hz, 4H). One proton not visible. HSQC, HMBC (Bruker 500 MHz, CD_3_OD) δ 166.68 (C), 163.39 (C), 155.17 (C), 152.94 (C), 132.50 (C), 131.21 (CH), 129.96 (2 CH), 129.02 (C), 127.82 (2 CH), 116. 88 (C), 113.31 (CH), 105.64 (CH), 104.65 (C), 63.87 (2 CH_2_), 56.71 (CH_2_), 48.08 (2 CH_2_), 25.53 (CH_2_). Mass calculated for C_21_H_20_F_2_N_4_O_3_S = 446.1224 g/mol. UPLC–MS (MeOH): 447.3 [M + H]^+^. Rt. = 0.57 min. Analysis type: LC–MS acid method.

#### Synthesis of *tert*-Butyl (2-((4-Methyl-5-(pyridin-4-yl)-4*H*-1,2,4-triazol-3-yl)­thio)­ethyl)­carbamate (**46**)

4-Methyl-5-(pyridin-4-yl)-4*H*-1,2,4-triazole-3-thiol (**27**) (100 mg, 0.52 mmol), 2-(Boc-amino)­ethyl bromide (**47**) (175 mg, 0.78 mmol, 1.5 equiv), and K_2_CO_3_ (147 mg, 1.04 mmol, 2.0 equiv) were placed in a sealed microwave vial and dissolved in 3.0 mL of a mixture of acetone/MeOH (1:1). The reaction was shaken overnight using a PLS (parallel reaction synthesizer) at rt. The following day, the reaction was checked with UPLC–MS (data not shown). Then, 15 mL of H_2_O was added to the mixture, and the compound was extracted with EtOAc (3 × 5 mL). The organic layer was washed with H_2_O (3 × 30 mL) and brine (1 × 10 mL). The organic phase was dried using a phase separator, and the solvent was removed under reduced pressure. The product was used for the next reaction without any further purification (**46,** 673 mg, yield = 77%) as a yellow solid. Purity = 100% calculated by LC–MS (acid method) a/a by UV. ^1^H NMR (Bruker 400 MHz, DMSO-*d*_6_) δ 8.77 (dd, *J* = 4.5, 1.6 Hz, 2H), 7.75 (dd, *J* = 4.5, 1.6 Hz, 2H), 3.67 (s, 3H), 3.30–3.20 (m, 4H), 1.37 (s, 9H). Mass calculated for C_15_H_21_N_5_O_2_S = 335.1416 g/mol. UPLC–MS (MeOH): *m*/*z* 336.25 [M + H]^+^. Rt. = 0.64 min. Analysis type: LC–MS acid method.

#### Synthesis of 2-((4-Methyl-5-(pyridin-4-yl)-4*H*-1,2,4-triazol-3-yl)­thio)­ethan-1-amine (**48**)

To (2-((4-methyl-5-(pyridin-4-yl)-4*H*-1,2,4-triazol-3-yl)­thio)­ethyl)­carbamate (**46**) (670 mg, 2.00 mmol, 1.0 equiv) in 10 mL of DCM, TFA (1.53 mL, 20.0 mmol, 10 equiv) was added. The reaction was stirred at rt for 2 h. The reaction was checked with UPLC–MS (data not reported). Then, TFA was removed under reduced pressure. The crude was dissolved in 5.0 mL of MeOH to perform an SCX purification using as eluent MeOH (3 × 5 mL) and methanolic ammonia 1 M in MeOH (3 × 5 mL). Both the two phases were checked with UPLC–MS. The solvent was removed under reduced pressure to afford the compound **48** (466 mg, yield = 99%) as a white solid. Purity = 100% calculated by LC–MS (basic method) a/a by UV. ^1^H NMR (Bruker 400 MHz, DMSO-*d*_6_) δ 8.77 (dd, *J* = 4.5, 1.6 Hz, 2H), 7.75 (dd, *J* = 4.5, 1.6 Hz, 2H), 3.68 (s, 3H), 3.27 (t, *J* = 6.8 Hz, 2H), 2.97 (t, *J* = 6.8 Hz, 2H). Two protons not visible. Mass calculated for C_10_H_13_N_5_S = 235.0892 g/mol. UPLC–MS (MeOH): *m*/*z* 236.24 [M + H]^+^. Rt. = 0.46 min. Analysis type: LC–MS basic method.

#### Synthesis of 3-Fluoro-*N*-(2-((4-methyl-5-(pyridin-4-yl)-4*H*-1,2,4-triazol-3-yl)­thio)­ethyl)­isonicotinamide (**5**)

A first solution made of 2-((4-methyl-5-(pyridin-4-yl)-4*H*-1,2,4-triazol-3-yl)­thio)­ethan-1-amine (**48**) (100 mg, 0.42 mmol, 1.0 equiv) and DIPEA (0.13 mL, 0.76 mmol, 1.8 equiv) in 5.0 mL of DMF was prepared and stirred at 0 °C. At the same time, another solution containing 3-fluoroisonicotinoyl chloride (**49**) (0.64 mmol, 1.5 equiv) in 3.0 mL of DMF was prepared and after 10 min added dropwise to the first solution at 0 °C. The reaction was left at rt overnight. The day after, the reaction was checked with UPLC–MS (data not reported). Then, DMF was partially removed under reduced pressure, and the product was purified by inverse FC using 30 g C18 cartridge by Isolera One, eluting with ACN + 0.1% formic acid and H_2_O + 0.1% formic acid, going from 5% to 60% ACN + 0.1% formic acid in 65 CV. The desired final product (**5**) was dried under reduced pressure. 30.5 mg, yield = 13%. White solid. Purity = 100% calculated by LC–MS (acid method) a/a by UV. mp = 148 °C. ^1^H NMR (Bruker 500 MHz, DMSO-*d*_6_) δ 8.91 (t, *J* = 5.3 Hz, 1H), 8.77 (dd, *J* = 4.5, 1.6 Hz, 2H), 8.69 (d, *J* = 1.0 Hz, 1H), 8.53 (dd, *J* = 4.8, 1.0 Hz, 1H), 7.74 (dd, *J* = 4.5, 1.6 Hz, 2H), 7.61–7.56 (m, 1H), 3.68 (s, 3H), 3.67–3.62 (m, 2H), 3.41 (t, *J* = 6.6 Hz, 2H). HSQC, HMBC (Bruker 500 MHz, DMSO-*d*_6_) δ 162.20 (C), 153.31 (C), 151.91 (C), 150.20 (2 CH), 146.29 (C), 146.11 (CH), 138.79 (CH), 130.67 (C), 123.11 (CH), 122.21 (C), 122.01 (2 CH), 38.78 (CH_2_), 31.77 (CH_2_), 31.62 (CH_3_). Mass calculated for C_16_H_15_FN_6_OS = 358.1012 g/mol. UPLC–MS (MeOH): *m*/*z* 359.4 [M + H]^+^. Rt. = 0.49 min. Analysis type: LC–MS acid method. HRMS (ESI-TOF, Exploris 240): experimental *m*/*z* 359.1084 [M + H]^+^, theoretical *m*/*z* 359.1085 [M + H]^+^. Δ = 0.0001.

#### Synthesis of Methyl 2-((4-Methyl-5-(pyridin-4-yl)-4*H*-1,2,4-triazol-3-yl)­thio)­acetate (**50**)

4-Methyl-5-(pyridin-4-yl)-4*H*-1,2,4-triazole-3-thiol (**27**) (100 mg, 0.52 mmol, 1.0 equiv), K_2_CO_3_ (144 mg, 0.94 mmol, 1.8 equiv), and ethyl 2-bromoacetate (**51**) (0.10 mL, 0.94 mmol, 1.8 equiv) were placed in a sealed microwave vial and dissolved in 3.0 mL of a mixture of acetone/MeOH (1:1). The reaction was shaken overnight using PLS for 3 h at rt. The reaction was checked using UPLC–MS (data not shown), which revealed the presence of the product of the trans-esterification with MeOH (reported in synthetical scheme). Then, 20 mL of H_2_O was added to the mixture, and the compound was extracted with EtOAc (3 × 8 mL). The organic layer was further washed with H_2_O (3 × 30 mL) and brine (10 mL). The organic phase was dried using a phase separator, and the solvent was removed under reduced pressure to afford the compound **50** (103 mg, yield = 75%) as a pale yellow oil. Purity = 96% calculated by LC–MS (acid method) a/a by UV. ^1^H NMR (Bruker 400 MHz, DMSO-*d*_6_) δ 8.77 (dd, *J* = 4.5, 1.6 Hz, 2H), 7.75 (dd, *J* = 4.5, 1.6 Hz, 2H), 4.15 (s, 2H), 3.70 (s, 3H), 3.67 (s, 3H). Mass calculated for C_11_H_12_N_4_O_2_S = 264.0681 g/mol. UPLC–MS (MeOH): *m*/*z* 265.25 [M + H]^+^. Rt. = 0.45 min. Analysis type: LC–MS acid method.

#### Synthesis of 2-((4-Methyl-5-(pyridin-4-yl)-4*H*-1,2,4-triazol-3-yl)­thio)­acetic Acid (**52**)

A solution of aqueous NaOH 4 M (0.25 mL) was added to methyl 2-((4-methyl-5-(pyridin-4-yl)-4*H*-1,2,4-triazol-3-yl)­thio)­acetate (**50**) (100 mg, 0.38 mmol, 1.0 equiv) while stirring. The reaction mixture was left at rt for 3 h. The reaction was checked by using UPLC–MS (data not shown). Then, 15 mL of H_2_O was added to the mixture, and an extraction was performed using EtOAc (3 × 5 mL). The compound remained in the aqueous phase. Water was removed under reduced pressure, and the crude was purified by FC on an 11 g C18 cartridge, using as eluent a mixture of H_2_O + formic acid 0.1% and ACN + formic acid 0.1%, with a gradient from 5% to 35% of ACN + formic acid 0.1% in 50 CV. The pure fractions were collected together, and the solvent was removed under reduced pressure to afford the compound **52** (39.0 mg, yield = 75%) as a pale yellow solid, which was used for the next reaction.

#### Synthesis of *N*-(2-Chloro-3-pyridinyl)-2-[(4-methyl-5-pyridin-4-yl-1,2,4-triazol-3-yl)­sulfanyl]­acetamide (**12**)

A solution of 2-((4-methyl-5-(pyridin-4-yl)-4*H*-1,2,4-triazol-3-yl)­thio)­acetic acid (**52**) (39.0 mg, 0.15 mmol, 1.0 equiv) and 2-chloropyridine-3-amine (**53**) (25.7 mg, 0.20 mmol, 1.3 equiv) in 5.0 mL of CPME was shaken in PLS for 10 min. After this time, *N*-methyl imidazole (0.04 mL, 0.55 mmol, 3.7 equiv) and TCFH (48.4 mg, 0.17 mmol, 1.15 equiv) were added, and the mixture was shaken at rt in PLS overnight. The day after, the reaction was checked with UPLC–MS (data not reported). The product was extracted with EtOAc (3 × 5 mL), and the organic phase was washed with H_2_O (3 × 15 mL) and brine (1 × 10 mL). The organic phase was dried with a phase separator, and the solvent was removed under reduced pressure. Then, the product was purified on 5 g silica cartridge by Isolera One, using as eluents cyclohexane and a mixture of EtOAc/EtOH (3:1), going from 5% to 70% of EtOAC/EtOH (3:1) in 70 CV. The product **12** (38.0 mg, yield = 70%) was recovered as a white solid. Purity = 100% calculated by LC–MS (acid method) a/a by UV. mp = 110 °C. ^1^H NMR (Bruker 500 MHz, DMSO-*d*_6_) δ 10.13 (s, 1H), 8.77 (dd, *J* = 4.5, 1.6 Hz, 2H), 8.28–8.18 (m, 2H), 7.75 (dd, *J* = 4.5, 1.6 Hz, 2H), 7.44 (dd, *J* = 8.0, 4.7 Hz, 1H), 4.26 (s, 2H), 3.72 (s, 3H). HSQC, HMBC (Bruker 500 MHz, DMSO-*d*_6_) δ 167.02 (C), 153.41 (C), 151.74 (C), 150.15 (2 CH), 145.37 (C), 145.19 (CH), 142.60 (C), 133.12 (CH), 123.27 (CH), 122.27 (C), 122.00 (2 CH), 36.71 (CH_2_), 31.90 (CH_3_). Mass calculated for C_15_H_13_ClN_6_OS = 360.0560 g/mol. UPLC–MS (MeOH): *m*/*z* 361.3 [M + H]^+^. Rt. = 0.55 min. Analysis type: LC–MS acid method. HRMS (ESI-TOF, Exploris 240): experimental *m*/*z* 361.0631 [M + H]^+^, theoretical *m*/*z* 361.0633 [M + H]^+^. Δ = 0.0002.

#### General Procedures of the Synthesis for 4,5-Disubstitued-3-thioacetamide Derivatives (**6**–**10**)

Procedure A: To a solution of the proper thio-1,2,4-triazole intermediate (**32**, **34**) (0.80 mmol, 1.0 equiv) in 8.0 mL of EtOH, 0.22 mL of a 4 M solution of KOH was added, and the mixture was stirred for 30 min at rt. After this time, the proper alkylating agent (**54**, **55**) (1.0–2.0 equiv) was added, and the mixture was refluxed for 3 h. The reaction was checked with UPLC–MS (data not reported). The solvent was removed under reduced pressure, and the solid was purified by FC, with specific conditions for each compound. The solvent was removed under reduced pressure to afford the desired compounds (**6**, **8**).

##### *N*-Cyclopentyl-2-((4-(2-methoxyethyl)-5-(thiophen-2-yl)-4*H*-1,2,4-triazol-3-yl)­thio)­acetamide (**6**)

46.7 mg, yield = 17%. White solid. Purity = 100% calculated by LC–MS (acid method) a/a by UV. mp = 150 °C. 2-Bromo-*N*-cyclopentylacetamide (**54**, 330 mg, 1.60 mmol, 2.0 equiv). Purification on a 25 g silica cartridge by Isolera One, using as eluents cyclohexane and a mixture of EtOAc/EtOH (3:1), going from 5% to 60% of EtOAc/EtOH (3:1) in 65 CV. ^1^H NMR (Bruker 500 MHz, DMSO-*d*_6_) δ 8.17 (d, *J* = 7.1 Hz, 1H), 7.79 (dd, *J* = 5.1, 1.1 Hz, 1H), 7.64 (dd, *J* = 3.7, 1.1 Hz, 1H), 7.24 (dd, *J* = 5.1, 3.7 Hz, 1H), 4.31 (t, *J* = 5.3 Hz, 2H), 3.95 (dd, *J* = 13.3, 7.1 Hz, 1H), 3.85 (s, 2H), 3.61 (t, *J* = 5.3 Hz, 2H), 3.18 (s, 3H), 1.81–1.55 (m, 4H), 1.52–1.28 (m, 4H). HSQC, HMBC (Bruker 500 MHz, DMSO-*d*_6_) δ 166.14 (C), 151.12 (C), 150.97 (C), 128.80 (CH), 128.05 (C), 127.98 (CH), 127.84 (CH), 69.49 (CH_2_), 58.16 (CH_3_), 50.44 (CH), 44.23 (CH_2_), 36.99 (CH_2_), 31.88 (2 CH_2_), 23.07 (2 CH_2_). Mass calculated for C_16_H_22_N_4_O_2_S_2_ = 366.1184 g/mol. UPLC–MS (MeOH): *m*/*z* 367.3 [M + H]^+^. Rt. = 0.82 min. Analysis type: LC–MS acid method. HRMS (ESI-TOF, Exploris 240): experimental *m*/*z* 367.1259 [M + H]^+^, theoretical *m*/*z* 367.1257 [M + H]^+^. Δ = 0.0002.

##### *N*-(*tert*-Butyl)-2-((4-(2-(dimethylamino)­ethyl)-5-(pyridin-3-yl)-4*H*-1,2,4-triazol-3-yl)­thio)­acetamide (**8**)

42.0 mg, yield = 14%. White solid. Purity = 99% calculated by LC–MS (basic method) a/a by UV. mp = 150 °C. *N*-(*tert*-Butyl)-2-chloroacetamide (**55**, 120 mg, 0.80 mmol, 1.0 equiv). Purification on 25 g silica cartridge by Isolera One, using as eluents DCM and a mixture of DCM/MeOH (9:1), with a gradient from 5% to 60% of DCM/MeOH (9:1) in 60 CV. ^1^H NMR (Bruker 500 MHz, DMSO-*d*_6_) δ 8.83 (dd, *J* = 2.2, 0.8 Hz, 1H), 8.74 (dd, *J* = 4.9, 1.6 Hz, 1H), 8.14–8.05 (m, 1H), 7.90 (s, 1H), 7.60 (ddd, *J* = 7.9, 4.9, 0.8 Hz, 1H), 4.08 (t, *J* = 6.4 Hz, 2H), 3.90 (s, 2H), 2.41 (t, *J* = 6.4 Hz, 2H), 1.97 (s, 6H), 1.24 (s, 9H). HSQC, HMBC (Bruker 500 MHz, DMSO-*d*_6_) δ 166.13 (C), 153.00 (C), 151.15 (C), 150.86 (C), 150.50 (CH), 148.65 (CH), 135.87 (CH), 123.55 (CH), 57.41 (CH_2_), 50.50 (C), 44.79 (2 CH_3_), 42.35 (CH_2_), 37.78 (CH_2_), 28.04 (3 CH_3_). Mass calculated for C_17_H_26_N_6_OS = 362.1889 g/mol. UPLC–MS (MeOH): *m*/*z* 363.5 [M + H]^+^. Rt. = 0.63 min. Analysis type: LC–MS basic method. HRMS (ESI-TOF, Exploris 240): experimental *m*/*z* 363.1962 [M + H]^+^, theoretical *m*/*z* 363.1962 [M + H]^+^. Δ = 0.0000.

Procedure B: The proper thio-1,2,4-triazole intermediate (**28**, **32**) (0.76 mmol, 1.0 equiv), K_2_CO_3_ (1.0–2.5 equiv), and the proper alkylating agent (**56**–**58**) (1.0–2.0 equiv) were placed in a screw-cap vial and dissolved in 6.0 mL of a mixture of acetone/MeOH (1:1). The reaction was shaken overnight using PLS or magnetic plate at 50 °C. The reaction was checked using UPLC–MS (data not shown) or TLC (DCM/MeOH 9:1). The solvent was removed under reduced pressure, and the crude product was purified by FC using specific conditions for each compound. The pure product was collected, and the solvent was removed under reduced pressure to afford the desired compounds (**7**, **9**–**10**).

##### 3-(3-((2-((2-Chloro-4-methylphenyl)­amino)-2-oxoethyl)­thio)-5-(pyridin-4-yl)-4*H*-1,2,4-triazol-4-yl)-*N*,*N*-dimethylpropan-1-aminium Formate (**7**)

31.6 mg, yield = 8%. Colorless oil. Purity = 100% calculated by LC–MS (acid method) a/a by UV. 2-Bromo-*N*-(2-chloro-4-methylphenyl)­acetamide (**56**, 399 mg, 1.52 mmol, 2.0 equiv), K_2_CO_3_ (116 mg, 0.84 mmol, 1.1 equiv). Double purification: (1) on a 11 g C18 cartridge by Isolera One, eluting with ACN and H_2_O + 0.1% NH_4_OH, with a gradient from 5% to 70% ACN in 55 CV; (2) on a 11 g C18 cartridge, using as eluent ACN + 0.1% formic acid and H_2_O + 0.1% formic acid, going from 5% to 60% ACN + 0.1% formic acid in 60 CV. ^1^H NMR (Bruker 500 MHz, DMSO-*d*_6_) δ 9.88 (s, 1H), 8.77 (dd, *J* = 4.5, 1.6 Hz, 2H), 8.19 (s, 1H), 7.72 (dd, *J* = 4.5, 1.6 Hz, 2H), 7.61 (d, *J* = 8.2 Hz, 1H), 7.32 (d, *J* = 0.9 Hz, 1H), 7.13 (dd, *J* = 8.2, 0.9 Hz, 1H), 4.25 (s, 2H), 4.14 (t, *J* = 6.5 Hz, 2H), 2.28 (s, 3H), 2.10 (t, *J* = 6.5 Hz, 2H), 1.98 (s, 6H), 1.76–1.64 (m, 2H). HSQC, HMBC (Bruker 500 MHz, DMSO-*d*_6_) δ 166.25 (C), 163.26 (CH), 153.05 (C), 151.58 (C), 150.29 (2 CH), 136.28 (C), 131.97 (C), 129.42 (CH), 127.85 (CH), 125.94 (C), 125.23 (CH), 122.39 (C), 122.18 (2 CH), 55.12 (CH_2_), 44.54 (2 CH_3_), 42.66 (CH_2_), 36.64 (CH_2_), 26.67 (CH_2_), 19.89 (CH_3_). Mass calculated for C_21_H_25_ClN_6_OS = 444.1499 g/mol. UPLC–MS (MeOH): *m*/*z* 445.3 [M + H]^+^ (not salified). Rt. = 0.52 min. Analysis type: LC–MS acid method. HRMS (ESI-TOF, Exploris 240): experimental *m*/*z* 445.1573 [M + H]^+^, theoretical *m*/*z* 445.1572 [M + H]^+^. Δ = 0.0001.

##### *N*-(3-Chloro-2-methylphenyl)-2-((4-(2-(dimethylamino)­ethyl)-5-(pyridin-3-yl)-4*H*-1,2,4-triazol-3-yl)­thio)­acetamide (**9**)

101 mg, yield = 59%. Pale yellow oil. Purity = 100% calculated by LC–MS (acid method) a/a by UV. 2-Bromo-*N*-(3-chloro-2-methylphenyl)­acetamide (**57**, 399 mg, 1.52 mmol, 2.0 equiv), K_2_CO_3_ (210 mg, 1.52 mmol, 2.0 equiv). Purification on 11 g C18 cartridge by Isolera One, eluting with ACN + 0.1% TFA and H_2_O + 0.1% TFA, going from 5% to 50% ACN + 0.1% TFA in 60 CV. ^1^H NMR (Bruker 400 MHz, DMSO-*d*_6_) δ 10.04 (s, 1H), 8.88 (d, *J* = 1.8 Hz, 1H), 8.80 (dd, *J* = 4.8, 1.6 Hz, 1H), 8.14 (dt, *J* = 7.9, 1.8 Hz, 1H), 7.63 (dd, *J* = 8.0, 4.8 Hz, 1H), 7.31 (dd, *J* = 7.9, 1.6 Hz, 2H), 7.20 (t, *J* = 8.0 Hz, 1H), 4.50–4.38 (m, 2H), 4.30 (s, 2H), 3.38–3.30 (m, 2H), 2.78 (s, 6H), 2.20 (s, 3H).^13^C NMR (Varian 101 MHz, DMSO-*d*_6_) δ 166.18 (C), 158.83 (C), 158.49 (C), 152.79 (C), 151.02 (CH), 148.79 (CH), 137.45 (C), 136.66 (CH), 133.88 (C), 130.58 (C), 126.95 (CH), 126.42 (CH), 124.41 (CH), 124.17 (CH), 53.99 (CH_2_), 42.54 (2 CH_3_), 38.89 (CH_2_), 37.59 (CH_2_), 15.03 (CH_3_). Mass calculated for C_20_H_23_ClN_6_OS = 430.1343 g/mol. UPLC–MS (MeOH): *m*/*z* 431.3 [M + H]^+^. Rt.= 0.52 min. Analysis type: LC–MS acid method. HRMS (ESI-TOF, Exploris 240): experimental *m*/*z* 431.1417 [M + H]^+^, theoretical *m*/*z* 431.1415 [M + H]^+^. Δ = 0.0002.

##### 2-((5-(5-Methoxypyridin-2-yl)-4-methyl-4*H*-1,2,4-triazol-3-yl)­thio)-1-morpholinoethan-1-one (**10**)

68.2 mg, yield = 59%. Pale yellow solid. Purity = 100% calculated by LC–MS (acid method) a/a by UV. mp = 198 °C. 2-Chloro-1-morpholinoethan-1-one (**58**, 124 mg, 0.76 mmol, 1.0 equiv), K_2_CO_3_ (210 mg, 1.52 mmol, 2.0 equiv). Purification on a 11 g C18 cartridge, using as eluents with ACN + 0.1% TFA and H_2_O + 0.1% TFA, going from 5% to 50% of ACN + 0.1% TFA in 60 CV. ^1^H NMR (Varian 400 MHz, DMSO-*d*_6_) δ 8.43 (d, *J* = 3.0 Hz, 1H), 8.06 (d, *J* = 8.8 Hz, 1H), 7.59 (dd, *J* = 8.8, 3.0 Hz, 1H), 4.25 (s, 2H), 3.92 (s, 3H), 3.91 (s, 3H), 3.65–3.53 (m, 4H), 3.52–3.40 (m, 4H).^13^C NMR (Varian 126 MHz, DMSO-*d*_6_) δ 165.65 (C), 155.87 (C), 152.97 (C), 151.06 (C), 139.65 (C), 136.72 (CH), 124.11 (CH), 121.88 (CH), 65.95 (2 CH_2_), 55.90 (CH_3_), 45.93 (CH_2_), 42.01 (CH_2_), 36.43 (CH_2_), 32.71 (CH_3_). Mass calculated for C_15_H_19_N_5_O_3_S = 349.1209 g/mol. UPLC–MS (MeOH): *m*/*z* 350.3 [M + H]^+^. Rt. = 0.66 min. Analysis type: LC–MS acid method. HRMS (ESI-TOF, Exploris 240): experimental *m*/*z* 350.1280 [M + H]^+^, theoretical *m*/*z* 350.1281 [M + H]^+^. Δ = 0.0001.

#### Synthesis of Ethyl 5-Amino-1-(5-methyl-4-(thiophen-2-yl)­pyrimidin-2-yl)-1*H*-pyrazole-4-carboxylate (**14**)

2-Hydrazineyl-5-methyl-4-(thiophen-2-yl)­pyrimidine (**59**) (500 mg, 2.42 mmol, 1.0 equiv) and ethyl (*E*)-2-cyano-3-ethoxyacrylate (**60**) (409 mg, 2.42 mmol, 1.0 equiv) were solubilized in 10 mL of EtOH, and the mixture was placed to reflux overnight. The reaction was checked with TLC (PetEt/EtOAc 6:4). The crude product was purified on a silica gel by eluting with PetEt and EtOAc with a gradient from 0% to 50% of EtOAc. The pure product was collected, and the solvent was removed under reduced pressure to afford **14** (293 mg, yield = 41%) as a white solid. Purity (method C) = 100%. mp = 185 °C. ^1^H NMR (Varian 400 MHz, DMSO-*d*_6_) δ 8.76 (s, 1H), 8.00–7.90 (m, 2H), 7.79 (s, 1H), 7.56 (s, 2H), 7.33 (dd, *J* = 5.1, 3.9 Hz, 1H), 4.23 (q, *J* = 7.1 Hz, 2H), 2.57 (s, 3H), 1.29 (t, *J* = 7.1 Hz, 3H). Mass calculated for C_15_H_15_N_5_O_2_S = 329.0946 g/mol. HRMS (ESI-TOF, Exploris 240): experimental *m*/*z* 352.0834 [M + H]^+^.

#### Synthesis of 5-Amino-1-(5-methyl-4-(thiophen-2-yl)­pyrimidin-2-yl)-1*H*-pyrazole-4-carboxylic Acid (**15**)

Ethyl 5-amino-1-(5-methyl-4-(thiophen-2-yl)­pyrimidin-2-yl)-1*H*-pyrazole-4-carboxylate (**14**) (72.0 mg, 0.27 mmol, 1.0 equiv) was solubilized in 3.0 mL of dioxane. Then, 12 mL of aqueous LiOH 3 M was added. The reaction was stirred for 3 days at rt. The reaction was checked with TLC (PetEt/EtOAc 6:4), and the product was revealed by KMnO_4_. The mixture was extracted with EtOAc (2 × 10 mL) and washed with H_2_O (2 × 20 mL). The aqueous phase was acidified with HCl 37% until pH ∼5, at which point the product started to precipitate. The solid was collected through filtration to afford **15** (60.0 mg, yield = 74%) as a white solid. ^1^H NMR (Varian 400 MHz, DMSO-*d*_6_) δ 12.15 (s, 1H), 8.75 (s, 1H), 8.01–7.89 (m, 2H), 7.76 (s, 1H), 7.49 (s, 2H), 7.33 (dd, *J* = 5.0, 3.9 Hz, 1H), 2.57 (s, 3H). Mass calculated for C_13_H_11_N_5_O_2_S = 301.0633 g/mol. HRMS (ESI-TOF, Exploris 240): experimental *m*/*z* 300.0557 [M – H]^−^.

#### Synthesis of 5-Amino-*N*,*N*-diethyl-1-(5-methyl-4-(thiophen-2-yl)­pyrimidin-2-yl)-1*H*-pyrazole-4-carboxamide (**13**)

5-Amino-1-(5-methyl-4-(thiophen-2-yl)­pyrimidin-2-yl)-1*H*-pyrazole-4-carboxylic acid (**15**) (50.0 mg, 0.16 mmol, 1.0 equiv) was solubilized in 4.0 mL of DMF. Then, diethylamine (**61**) (0.02 mL, 0.22 mmol, 1.4 equiv), DIPEA (0.14 mL, 0.80 mmol, 5.0 equiv), and HBTU (75.8 mg, 0.20 mmol, 1.3 equiv) were added in this exact order. The mixture was stirred at 50 °C overnight. The reaction was checked with TLC (DCM/MeOH 9:1). The mixture was extracted with EtOAc (2 × 5 mL), and the organic phase was washed with H_2_O (3 × 15 mL), brine (1 × 10 mL), and saturated aqueous solution of NaHCO_3_ (1 × 10 mL). The organic layer was dried over Na_2_SO_4_. The solid was filtered off, and the solvent was removed under reduced pressure. The crude was purified by direct FC on a silica gel by eluting with DCM and MeOH going from 0% to 15% of MeOH. The solvent was removed under reduced pressure to afford the desired compound **13** (23.0 mg, yield = 40%) as a white solid. Purity (method C) = 100%. mp = 185 °C. ^1^H NMR (Varian 400 MHz, DMSO-*d*_6_) δ 8.75 (s, 1H), 7.95–7.94 (m, 1H), 7.93 (s, 1H), 7.72 (s, 1H), 7.70 (s, 2H), 7.33 (dd, *J* = 4.9, 4.1 Hz, 1H), 3.47 (q, *J* = 7.0 Hz, 4H), 2.57 (s, 3H), 1.18 (t, *J* = 7.0 Hz, 6H). ^13^C NMR (Varian 101 MHz, DMSO-*d*_6_) δ 163.98 (C), 161.06 (CH), 157.48 (C), 155.01 (C), 152.93 (C), 141.60 (C), 140.24 (CH), 131.74 (CH), 131.67 (CH), 129.36 (CH), 122.76 (C), 95.69 (C), 41.12 (2 CH_2_), 17.50 (CH_3_), 13.80 (2 CH_3_). Mass calculated for C_17_H_20_N_6_OS = 356.1419 g/mol. HRMS (ESI-TOF, Exploris 240): experimental *m*/*z* 357.1492 [M + H]^+^, theoretical *m*/*z* 357.1491 [M + H]^+^. Δ = 0.0001.

#### Synthesis of 3-(2-Amino-6-bromopyrimidin-4-yl)­benzonitrile (**62**)

A three-neck flask was dried through vacuum-Ar. Then, 4,6-dibromopyrimidin-2-amine (**63**) (500 mg, 1.98 mmol, 1.3 equiv), 3-cyanophenylboronic acid (**64**) (232 mg, 1.58 mmol, 1.0 equiv), Pd­(PPh_3_)_4_ (115 mg, 0.08 mmol, 0.05 equiv), and 4.0 mL of an aqueous solution of Na_2_CO_3_ 2 M were solubilized in 10 mL of a mixture of dry toluene and EtOH (7:3). The reaction mixture was stirred at reflux at 110 °C for 5 h. The reaction was checked by TLC (PetEt/EtOAc 7:3). The reaction mixture was extracted with DCM (3 × 5 mL), and the organic phase was washed with H_2_O (3 × 15 mL) and brine (1 × 10 mL). The organic phase was dried over Na_2_SO_4_. Then the solid was filtered off, and the solvent was removed under reduced pressure. The resulting residue was purified by direct phase FC on a silica gel by eluting with PetEt and EtOAc going from 0% to 30% of EtOAc. The fractions containing the pure product were collected together, and the solvent was removed under reduced pressure to afford the desired compound **62** (111 mg, yield = 25%) as a white solid. ^1^H NMR (Varian 400 MHz, DMSO-*d*_6_) δ 8.54 (t, *J* = 1.5 Hz, 1H), 8.41 (dt, *J* = 7.7, 1.5 Hz, 1H), 8.00 (dt, *J* = 7.7, 1.5 Hz, 1H), 7.72 (t, *J* = 7.7 Hz, 1H), 7.55 (s, 1H), 7.30 (s, 2H). Mass calculated for C_11_H_7_BrN_4_ = 273.9854 g/mol. ESI-MS (LTQ-XL, MeOH) *m*/*z* 275.00 ^79^Br­[M + H]^+^, *m*/*z* 277.00 ^81^Br­[M + H]^+^.

#### Synthesis of 3-(2-Amino-6-(5-methylfuran-2-yl)­pyrimidin-4-yl)­benzonitrile (**16**)

A three-neck flask was anhydrified through vacuum-Ar. Then, 3-(2-amino-6-bromopyrimidin-4-yl)­benzonitrile (**62**) (200 mg, 0.73 mmol, 1.0 equiv), 4,4,5,5-tetramethyl-2-(5-methylfuran-2-yl)-1,3,2-dioxaborolane (**65**) (0.95 mmol, 1.3 equiv), Pd­(PPh_3_)_4_ (59.5 mg, 0.05 mmol, 0.07 equiv), and Na_2_CO_3_ (232 mg, 2.19 mmol, 3.0 equiv) were suspended in 8.0 mL of a solution of dry dioxane and H_2_O (3:1). The reaction mixture was stirred at reflux overnight. The reaction was checked by TLC (PetEt/EtOAc 7:3). Then, 20 mL of H_2_O was added to the reaction mixture, and the product was extracted with DCM (3 × 6 mL). The organic layer was washed with brine (1 × 15 mL) and then dried over Na_2_SO_4_. Then the solid was filtered off, and the solvent was removed under reduced pressure. The resulting residue was purified by direct phase FC on a silica gel by eluting with PetEt and EtOAc, going from 0% to 50% of EtOAc. Pure fractions were collected, and the solvent was removed under reduced pressure to afford the desired compound (**16**):114 mg, yield = 57%. White solid. Purity (method C) = 97%, mp = 225 °C. ^1^H NMR (Varian 500 MHz, DMSO-*d*_6_) δ 8.58 (s, 1H), 8.46 (dt, *J* = 8.0, 1.3 Hz, 1H), 7.99–7.96 (m, 1H), 7.73 (t, *J* = 8.0 Hz, 1H), 7.53 (s, 1H), 7.26 (d, *J* = 3.3 Hz, 1H), 6.83 (s, 2H), 6.34 (dd, *J* = 3.3, 1.0 Hz, 1H), 2.39 (s, 3H). ^13^C NMR (Varian 126 MHz, DMSO-*d*_6_) δ 163.86 (C), 162.06 (C), 156.99 (C), 154.86 (C), 150.25 (C), 138.31 (C), 133.80 (CH), 131.32 (CH), 130.41 (CH), 130.03 (CH), 118.64 (C), 113.66 (CH), 111.92 (C), 108.96 (CH), 99.78 (CH), 13.65 (CH_3_). Mass calculated for C_16_H_12_N_4_O = 276.1011 g/mol. HRMS (ESI-TOF, Exploris 240): experimental *m*/*z* 277.1083 [M + H]^+^, theoretical *m*/*z* 277.1084 [M + H]^+^. Δ = 0.0001.

#### Synthesis of 1-(2-Methoxyphenyl)-3-(4-methylpyridin-2-yl)­prop-2-en-1-one (**66**)

A solution (4.2 mL) of KOH 85% in a mixture of H_2_O/MeOH (1:6) was cooled to 0 °C for 10 min. Then 2-methoxybenzaldehyde (**67**) (8.25 mmol, 1.0 equiv) was added while stirring, keeping the temperature to 0 °C. Then, 1-(4-methylpyridin-2-yl)­ethan-1-one (**68**)­(8.25 mmol, 1.0 equiv) was added, and the reaction was stirred at 0 °C for 3 h. The reaction was checked by TLC (DCM/MeOH 9:1). The solid suspended in the reaction vessel was filtered and washed with cold MeOH (3 × 10 mL) to afford the desired compound (**66**): 2000 mg, yield = 54%. White solid.^1^H NMR (Varian 400 MHz, DMSO-*d*_6_) δ 8.64 (d, *J* = 4.9 Hz, 1H), 8.27 (d, *J* = 16.3 Hz, 1H), 8.11 (d, *J* = 16.3 Hz, 1H), 7.94 (s, 1H), 7.81 (dd, *J* = 8.3, 1.8 Hz, 1H), 7.51 (d, *J* = 4.8 Hz, 1H), 7.49–7.43 (m, 1H), 7.14 (d, *J* = 8.3 Hz, 1H), 7.04 (t, *J* = 7.5 Hz, 1H), 3.91 (s, 3H), 2.44 (s, 3H).

#### Synthesis of 4-(2-Methoxyphenyl)-6-(4-methylpyridin-2-yl)­pyrimidin-2-amine (**17**)

1-(2-Methoxyphenyl)-3-(4-methylpyridin-2-yl)­prop-2-en-1-one (**66**) (5.85 mmol, 1.0 equiv), guanidine hydrochloride (**69**) (838 mg, 8.78 mmol, 1.5 equiv), and 2.2 mL of aqueous KOH 50% were added in 14 mL of EtOH. The reaction was stirred at reflux for 1 h. After 1 h, 2.2 mL of H_2_O_2_ 30% was added in a period of 5 min. The reaction was stirred at reflux for 2 h. At the end, the reaction was checked by TLC (PetEt/EtOAc 1:1). The mixture was extracted with EtOAc (2 × 5 mL), and the organic layer was washed with H_2_O (2 × 15 mL) and brine (1 × 10 mL). The organic phase was dried over Na_2_SO_4_. Then the solid was filtered off, and the mixture was concentrated under reduced pressure. The resulting residue was purified by direct phase FC on a silica gel by eluting with PetEt and EtOAc, going from 0% to 50% of EtOAc. The pure product was collected, and the solvent was removed under reduced pressure to afford the desired compound (**17**): 102 mg, yield = 9%. Yellow solid. Purity (method C) = 99%. mp = 160 °C. ^1^H NMR (Varian 400 MHz, DMSO-*d*_6_) δ 8.56 (d, *J* = 4.9 Hz, 1H), 8.21–8.17 (m, 1H), 7.98 (s, 1H), 7.79 (dd, *J* = 7.7, 1.8 Hz, 1H), 7.48–7.43 (m, 1H), 7.35 (dd, *J* = 4.9, 0.8 Hz, 1H), 7.17 (d, *J* = 7.7 Hz, 1H), 7.07 (td, *J* = 7.5, 0.8 Hz, 1H), 6.70 (s, 2H), 3.85 (s, 3H), 2.42 (s, 3H). ^13^C NMR (Varian 101 MHz, DMSO-*d*_6_) δ 164.83 (C), 164.06 (C), 163.07 (C), 157.69 (C), 154.45 (C), 149.49 (CH), 148.12 (C), 131.38 (CH), 130.49 (CH), 127.14 (CH), 126.17 (CH), 121.89 (C), 120.68 (CH), 112.28 (CH), 107.08 (CH), 55.99 (CH_3_), 20.94 (CH_3_). Mass calculated for C_17_H_16_N_4_O = 292.1324 g/mol. HRMS (ESI-TOF, Exploris 240): experimental *m*/*z* 293.1399 [M + H]^+^, theoretical *m*/*z* 293.1397 [M + H]^+^. Δ = 0.0002.

#### Synthesis of Ethyl 2-Amino-4,6-di­(thiophen-2-yl)­nicotinate (**70**)

Thiophene-2-carbaldehyde (**71**) (2.65 mL, 24.6 mmol, 1.0 equiv), 1-(thiophen-2-yl)­ethan-1-one (**72**) (2.50 mL, 24.6 mmol, 1.0 equiv), ethylcyano acetate (**73**) (2.62 mL, 24.6 mmol, 1.0 equiv), and NH_4_OAc (5688 mg, 73.8 mmol, 3.0 equiv) were solubilized in 10 mL of toluene. The mixture was stirred to reflux overnight. The reaction was checked with TLC (PetEt/EtOAc 7:3). The mixture was extracted with EtOAc (2 × 10 mL), and the organic phase was washed with H_2_O (3 × 30 mL) and brine (1 × 20 mL). The organic layer was dried over Na_2_SO_4_. The solid was filtered off, and the solvent was removed under reduced pressure. The crude was purified by direct FC on a silica gel by eluting with PetEt and EtOAc, going from 0% to 70% of EtOAc. The solvent was removed under reduced pressure to afford the desired compound **70** (2800 mg, yield = 34%) as a white solid. ^1^H NMR (Varian 400 MHz, DMSO-*d*_6_) δ 7.86 (dd, *J* = 3.7, 1.1 Hz, 1H), 7.68 (ddd, *J* = 8.9, 3.7, 1.1 Hz, 2H), 7.22 (dd, *J* = 3.6, 1.2 Hz, 1H), 7.19 (s, 1H), 7.15 (ddd, *J* = 5.1, 3.6, 1.2 Hz, 2H), 6.57 (s, 2H), 4.06 (q, *J* = 7.1 Hz, 2H), 0.96 (t, *J* = 7.1 Hz, 3H).

#### Synthesis of Ethyl 2-Amino-4,6-di­(thiophen-2-yl)­nicotinic acid (**74**)

Ethyl 2-amino-4,6-di­(thiophen-2-yl)­nicotinate (**70**) (1000 mg, 3.03 mmol, 1.0 equiv) was solubilized in 10 mL of EtOH. Then, 15 mL of an aqueous solution of NaOH 4 M was added to the mixture. The reaction was refluxed overnight. The reaction was checked with TLC (PetEt/EtOAc 7:3) and revealed with KMnO_4_. The mixture was extracted with EtOAc (1 × 10 mL), and the organic phase was washed with H_2_O (3 × 30 mL). Then, HCl 37% was added on the aqueous phase until pH ∼3, at which point the product started to precipitate. The solid was filtered and dried to afford the desired compound **74** (653 mg, yield = 71%) as a white solid. Mass calculated for C_14_H_10_N_2_O_2_S_2_ = 302.0184 g/mol. ESI-MS (LTQ-XL, MeOH): *m*/*z* 303.00 [M + H]^+^.

#### Synthesis of 2-Methoxyethyl 2-amino-4,6-di­(thiophen-2-yl)­nicotinate (**18**)

Ethyl 2-amino-4,6-di­(thiophen-2-yl)­nicotinic acid (**74**) (300 mg, 0.99 mmol, 1.0 equiv), 2-bromomethoxy ethane (**75**) (0.10 mL, 1.09 mmol, 1.1 equiv), and K_2_CO_3_ (410 mg, 2.97 mmol, 3.0 equiv) were solubilized in 5.0 mL of DMF. The mixture was stirred overnight at rt. The reaction was checked with TLC (PetEt/EtOAc 7:3). Then, 20 mL of H_2_O was added to the mixture, and the product was extracted with EtOAc (3 × 5 mL). The organic phase was washed with H_2_O (1 × 30 mL) and brine (1 × 15 mL). The organic phase was dried over Na_2_SO_4_. Then the solid was filtered off, and the solvent was removed under reduced pressure. The crude product was purified by direct FC on a silica gel by eluting with PetEt and EtOAc, going from 0% to 30% of EtOAc. The solvent was removed under reduced pressure to afford the desired compound **18** (155 mg, yield = 32%) as a colorless oil. Purity (method C) = 99%. mp = 160 °C. ^1^H NMR (Varian 500 MHz, DMSO-*d*_6_) δ 7.86 (dd, *J* = 3.7, 0.9 Hz, 1H), 7.70 (dd, *J* = 5.1, 1.1 Hz, 1H), 7.67 (dd, *J* = 5.0, 0.9 Hz, 1H), 7.26 (dd, *J* = 3.6, 1.1 Hz, 1H), 7.19 (s, 1H), 7.17–7.13 (m, 2H), 6.53 (s, 2H), 4.28–4.17 (m, 2H), 3.40–3.36 (m, 2H), 3.17 (s, 3H). ^13^C NMR (Varian 126 MHz, DMSO-*d*_6_) δ 167.34 (C), 157.16 (C), 152.25 (C), 143.73 (C), 143.42 (C), 140.08 (C), 129.21 (CH), 128.40 (CH), 127.78 (CH), 127.70 (CH), 127.21 (CH), 126.51 (CH), 108.15 (CH), 106.82 (C), 69.25 (CH_2_), 63.78 (CH_2_), 57.93 (CH_3_). Mass calculated for C_17_H_16_N_2_O_3_S_2_ = 360.0602 g/mol. HRMS (ESI-TOF, Exploris 240): experimental *m*/*z* 361.0674 [M + H]^+^, theoretical *m*/*z* 361.0675 [M + H]^+^. Δ = 0.0001.

### Biological Assays at Human Adenosine Receptors

#### Cell Culture

Chinese hamster ovary (CHO) cells stably expressing human ARs were grown adherently and maintained in Dulbecco’s modified Eagle’s medium with nutrient mixture F12 (DMEM/F12), supplemented with 10% fetal bovine serum (FBS), 100 U/mL penicillin, 100 μg/mL streptomycin, 2.5 μg/mL amphotericin, 1 mM sodium pyruvate, and 0.1 mg/mL Geneticin (G418) at 37 °C, and aerated with 5% CO_2_/95% O_2_.

#### Membrane Preparation

All of the pharmacological methods followed the procedures as described earlier.^98^ In brief, the membranes for radioligand binding were prepared from CHO cells stably transfected with human ARs through two centrifugations at different speeds. The first low-speed (1000*g*) centrifugation allowed for the removal of cell fragments and nuclei, while the second, performed at high speed (100,000*g*), allowed for the precipitation of the crude membrane fractions. The resulting membrane pellet was resuspended in the buffer used for the respective binding experiments, frozen in liquid nitrogen, and stored in aliquots at −80 °C.

#### Binding Assay

The binding affinity of the novel compounds was evaluated using radioligand competition experiments in CHO cells stably expressing hA_1_ AR, hA_2A_ AR, and hA_3_ AR subtypes, as early described.^89^ The radioligands used were 1.0 nM [^3^H]­CCPA (*K*_d_ = 1.1 nM) for hA_1_, 10 nM [^3^H]­NECA (*K*_d_ = 20 nM) for hA_2A_, and 1.0 nM [^3^H]­HEMADO (*K*_d_ = 1.5 nM) for hA_3_ receptors. Results were expressed as *K*_i_ values (dissociation constants), which were calculated with the program GraphPad (GraphPad Software, San Diego, CA, USA). Each concentration was tested three to five times in duplicate, and the values were given as the mean ± standard deviation (SD).

#### Functional Study at Human A_2B_ AR

Functional activity was determined as described earlier.^99^ Briefly, cells stably expressing hA_2B_ AR and the plasmid pGloSensor-22F coding for the biosensor were cultured. This biosensor encodes for a genetically modified form of firefly luciferase into which a cAMP-binding protein moiety was inserted. The desiderate cell number was harvested and incubated for 2 h at rt, with 3% v/v GloSensor cAMP reagent stock solution, 10% FBS, and 87% CO_2_ independent medium. Cells were dispensed in the wells of a 384-well plate, and the reference agonist NECA or the understudy compounds were added at different concentrations. Since the compounds were unable to stimulate cAMP production, they were studied as antagonists. The antagonist profile was evaluated by assessing the ability to counteract a NECA-induced increase in cAMP accumulation. Responses were expressed as a percentage of the maximal relative luminescence units (RLU). Concentration–response curves were fitted by a nonlinear regression using the Prism software. The antagonist profile of the compounds was expressed as the IC_50_, which is the concentration of the antagonist that produces a 50% inhibition of the agonist effect. Each concentration was tested three to five times in duplicate, and the values were given as the mean ± standard deviation (SD).

#### Evaluation of Apparent Passive Permeation of BBB by the PAMPA-BBB Assay

Ten commercial drugs with known permeabilities (atenolol, caffeine, desipramine, enoxacin, hydrocortisone, ofloxacin, piroxicam, promazine, testosterone, and verapamil) were included in the experiment to obtain cutoff values. One to two milligrams of each compound was weighed and dissolved in 1 mL of ethanol and then diluted to 5 mL with PBS pH 7.4/ethanol buffer solution (70:30). The solution was filtered through PVDF membrane filters (30 mm diameter, 0.45 mm pore size), and the absorbance spectra were recorded using a microplate reader (Tecan Infinite M1000, wavelength step size 2 nm; wavelength start: 230 nm; wavelength end: 360 nm; settle time 0 ms. scan number 66). If the concentration of the solutions was too high, they were diluted using the same PBS/ethanol solution (70:30). The ranges of optimum absorbance values, for which the dilution was taken as a starting point for the subsequent steps, were set, and the maximus and minimus with relative absorbances were taken for each compound. Then, 180 μL of buffer solution was placed in the wells of the acceptor plate (MultiScreen 96-well Culture Tray clear, Merck Millipore), and subsequently, 4 μL/well of a pig brain lipid solution (Merck Millipore) in dodecane (20 mg/mL) was gently laid on the membrane of each well of the donor plate (Multiscreen IP Sterile Plate, PVDF membrane, pore size 0.45 mM, Merck Millipore). The membranes with the lipid layer were dried in air for 10 min at room temperature. Once the preparation was complete, the donor plate was carefully placed over the acceptor plate to form a “sandwich”, and after 2.5 h of incubation at 25 °C, the donor plate was removed. The solution in the wells of the acceptor plate was transferred to a transparent UV plate (Microplate UV 96 wells, flat bottom, transparent, Greiner) for absorbance reading. The absorbance values of these solutions were evaluated at the same wavelengths identified for the substances in the initial reading. The PAMPA assay was performed in duplicate (two plates) and in technical triplicate (three wells per plate). Data were elaborated as reported in SI Tables S7 and S8 and Figure S8.

## Supplementary Material







## Notes

Data set A_2A_ (CSV)
Molecular formula strings and associated biochemical data (CSV)
Supporting figures, tables, protocols, and spectra data (PDF)
