# Supplementary material for: Applying Deep-Learning-Driven De Novo Design to Hit Identification: A Case Study on A2A Adenosine Receptor Antagonists
Source: J Med Chem. 2026 Jun 19;69(13):15403–22. doi: 10.1021/acs.jmedchem.6c00231 (PMC13370865; doi:10.1021/acs.jmedchem.6c00231)

Supporting Information

Applying Deep Learning–Driven De Novo Design to Hit Identification: A Case Study on A_{2A} Adenosine Receptor Antagonists

Margherita Persico,[¥] Alessandra Micoli,[§] Veronica Salmaso,[†] Agostino Cianciulli,[§] Stefano Moro,[†] Giampiero Spalluto,[¥] Michela Buccioni,[‖] Gabriella Marucci,[‖] Rosaria Volpini,[‖] Alfonso Pozzan,[§] Fabrizio Micheli,[§] Stephanie Federico[¥*]*

[¥]Department of Chemical and Pharmaceutical Sciences, Via Licio Giorgieri 1, 34127 Trieste;

[§]Aptuit, an Evotec Company, Via Alessandro Fleming 4, Verona, 37135, Italy;

[†]Molecular Modeling Section (MMS), Department of Pharmaceutical and Pharmacological Sciences, University of Padova, via Marzolo 5, Padova, 35131, Italy;

[‖]Medicinal Chemistry Unit, School of Pharmacy, University of Camerino, Camerino, 62032, Italy.

**Corresponding Authors:* Alessandra Micoli, alessandra.micoli@evotec.com; Stephanie Federico, sfederico@units.it

Table of contents

CHEMBL dataset of A_{2A} AR antagonists, partial and inverse agonists…………………………...S3

SciFinder-n dataset of A_{2A} AR antagonists, partial and inverse agonists………………………………S4

Final dataset of A_{2A} AR antagonists, partial and inverse agonists……………………………….....S5

Datasets of A_1, A_{2B}, A_3 ARs antagonists…………………………………………………………...S5

Table S1: Validation set……………………………………………………………………………..S6

Table S2: Classification machine learning models developed for the different targets…………………….S10

Table S3: Regression machine learning models developed for A_{2A} AR Antagonist………………………..S11

Table S4: Classification machine learning models developed for the different targets…………………….S11

Table S5: Regression machine learning models developed for A_{2A} AR antagonists……………………….S12

Figure S1: Structures of three known antagonists used as inception in REINVENT generative tool……...S12

Figure S2: Graphs generated with TensorBoard illustrating the average score obtained by REINVENT…S13

Figure S3: Pharmacophores……………………………………………………………………...S14

Figure S4: 34 Molecules filtered by using the three pharmacophores……………………………………..S15

Table S6: 100 best ranked molecules coming from the application of Bayesian Optimization……………S16

Figure S5: 13 molecules selected for visual inspection of ranking value for BO analysis………………...S19

PAMPA: Data elaboration……………………………………………………………………………..S20

Table S7: Values used for the generation of the calibration line……………………………………….....S21

Figure S6: PAMPA calibration line…………………………………………………………………..S21

Table S8: Apparent Permeability of synthesised compounds **(13,14,16-18)**………………………………S21

Table S9: The maximum Tanimoto similarity coefficient…………………………………………………S22

Table S10: Details on generative setup S22

Scheme S1: Synthesis of 1,2,4-Triazole derivatives…………………………………………………….S263

Scheme S2: Synthesis of pyrazole-pyrimidine derivatives……………………………………………...S274

Scheme S3: Synthesis of 2-amino pyrimidine derivatives through Suzuki-Miyaura reaction…………….S274

Scheme S4: Synthesis of 2-amino pyrimidine derivatives through cyclization reaction………………….S284

Scheme S5: Synthesis of 2-amino pyridine derivatives………………………………………………...S25

References……………………………………………………………………………………….S26

Compounds' spectra……………………………………………………………………………S28

CHEMBL dataset of A_{2A} AR antagonists, partial and inverse agonists

Dataset containing literature data was derived from CHEMBL (https://www.ebi.ac.uk/chembl/), which is an open-source platform of EML-EBI property, first released in 2009. It is a manually cured database containing bioactive molecules with drug-like properties, published in scientific literature and coming from different sources, e.g. articles, Patents, Binding DB and books. It covers different fields of chemistry, biology and medicine, by public data about compounds, assays, tissues, documents, targets, cells, data warnings, drugs, indications and mechanisms.[1] Particularly the research on CHEMBL was made in different steps: 1) Search on CHEMBL for Targets using three key words: "Adenosine A_{2A} receptor", "A_{2A}" and "ADORA2A"; 2) Selection of the appropriate targets (target organism), including the three different synonyms explicated in the previous point, looking to different species, i.e. mus musculus (CHEMBL2115), rattus norvegicus (CHEMBL302), cavia porcellus (CHEMBL2605) and Homo Sapiens (CHEMBL251); 3) Visually inspection of molecules published for the different species and identification of "Homo Sapiens" (CHEMBL251) which contained most of the results; 4) Filter for Target organism Homo Sapiens (CHEMBL251); 5) Filter for type "small molecule".

These molecules, displayed as SMILES strings, were automatically downloaded from CHEMBL in a .csv file, with a total of 14,175 entries published from 1989 to mid-2021. Molecules included in this file, were characterized by three fundamental features that allowed us to easily and quickly recognize them, i.e. molecule CHEMBL ID, canonical SMILE and isomeric SMILE. The molecule ID is a unique code referred to a molecule, aimed at recognizing it on the site. Canonical and isomeric

SMILE, define the structure of each molecule, respectively hiding or explicating the stereochemistry. From this file, different filters were applied by directly working on the .csv file using Excel: 1) Removal of duplicates. When affinity or activity data of duplicates were not convergent, only the data with most records, or the best one in terms of reliability of the assay was kept. In this case, the chosen value was the one for which the assayed organism was "Homo Sapiens"; 2) Manual division of molecules in Agonist, Antagonists and Partial / Inverse Agonists. Since the mechanism of action was missing in ChEMBL for most compounds, this information was retrieved from the original publications, which also allowed verification of data accuracy (e.g., values, units, species) exported from ChEMBL. Finally, only partial, inverse agonists and antagonists were kept for the next steps; 3) Join of data towards A_1, A_{2B} and A_3 ARs, basing on CHEMBL ID of the molecule and of the targets

After the application of all these filters, the final number of molecules included in CHEMBL dataset was 4,777 including antagonists, partial and inverse agonists.

SciFinder-n dataset of A_{2A} AR antagonists, partial and inverse agonists

Dataset containing molecules published in patents, was derived from SciFinder, and in particular SciFinder-n (https://scifinder-n.cas.org).[2] The research was made using an advanced search in references, using as key words the concepts "Antagonist" and "Adenosine A2A Receptor". Data were filtered for "patents". Then, all the results were deeply analysed, by visually looking at the molecules published in each, particularly by identifying patents which include deep SAR analysis. The interesting bibliographic results were selected and then molecules related to each of them were visually inspected. On the latter, other additional filters were applied: 1) Reaction role: include product, exclude reactants, reagents and catalysts; 2) Reference role: biological study, therapeutic use; 3) Target indicator: membrane proteins, receptor antagonists. The resulting molecules were

automatically downloaded from SciFinder-n as an Excel file .xlsx, including substance identifiers, i.e. CAS number and SMILES string.

This file contained a total of 13,149 molecules which were further analysed, to collect affinity or activity data at the four ARs, as well as interesting molecules included in the patent but not in the 13,149 molecules automatically downloaded. Some of the data were expressed as pIC_{50} or pKi and, therefore, data were converted in IC_{50} or Ki. Moreover, for each molecule, additional parameters were added, i.e. type of assay (binding or functional) and assay description. Thus, to allow a rational analysis of the dataset. Molecules included in this dataset, were identified through CAS number and SMILES string, except for the molecules that were added by reading each patent which were numbered from 1 to 99. Particularly, referring to the SMILES, both canonical not explicating the stereochemistry and isomeric SMILES were included. The final number of molecules included in this dataset were 13,479.

Final dataset of A_{2A} AR antagonists, partial and inverse agonists

The final dataset was built by merging the previous described ones, i.e. CHEMBL and Scifinder-n Datasets. At this stage, the total average of molecules was 18,256. This dataset has been further refined by different steps: 1) Removal of molecules with no activity data on A_{2A} AR; 2) Removal of duplicates. After these steps, the final number of molecules was 9,237. A list of all the molecules included in the dataset is available as a xls file in supplementary material.

Datasets of A_1, A_{2B}, A_3 ARs antagonists

The datasets for A_1, A_{2B}, A_3 ARs were built by collecting data published in CHEMBL (https://www.ebi.ac.uk/chembl/) for each receptor. In this case, the research included was less detailed if compared to the one used for A_{2A} AR, as it was used only to have an idea of the selectivity

profile. Therefore, for all the three receptors common steps have been used to build the datasets: 1) Research on CHEMBL for target, i.e A_1 receptor or A_{2B} receptor or A_3 receptor; 2) Selection of activity types, i.e. Ki, IC_{50} and % inhibition; 3) Selection of assay type, i.e. Binding and Functional; 4) Download of .csv files. In total, the datasets contained 5,313 molecules for A_1 AR, 4,037 molecules for A_{2B} AR, and 5,136 molecules for A_3 AR.

***Table S1**: Validation set*

Molecule CHEMBL ID	Smiles	Standard Type	Standard Relation	Standard Value	Standard Units	Document ChEMBL ID	Document Journal	Document Year
CHEMBL3137331	CNC(=O)c1ccc(Nc2ncc(C(F)(F)F)c(NCc3nccnc3N(C)S(C)(=O)=O)n2)cc1	Ki	'='	370	nM	CHEMBL4507299		2021
CHEMBL4794145	CCOC(=O)CC[C@H](Nc1nc2nn(C)cc2c2nc(-c3ccco3)nn12)C(=O)OCC	Ki	'='	2670	nM	CHEMBL4673199	RSC Med Chem	2021
CHEMBL4797620	CCOC(=O)C(Nc1nc2nn(C)cc2c2nc(-c3ccco3)nn12)C(C)C	Ki	'='	1272	nM	CHEMBL4673199	RSC Med Chem	2021
CHEMBL4785862	CCOC(=O)[C@H](Cc1ccccc1)Nc1nc2nn(C)cc2c2nc(-c3ccco3)nn12	Ki	'='	1300	nM	CHEMBL4673199	RSC Med Chem	2021
CHEMBL4758353	CCCCC(Nc1nc2nn(C)cc2c2nc(-c3ccco3)nn12)C(=O)OCC	Ki	'='	551	nM	CHEMBL4673199	RSC Med Chem	2021
CHEMBL4740116	CCOC(=O)C(Nc1nc2nn(C)cc2c2nc(-c3ccco3)nn12)c1ccccc1	Ki	'='	860	nM	CHEMBL4673199	RSC Med Chem	2021
CHEMBL2180931	C[C@H](Nc1nc2nn(C)cc2c2nc(-c3ccco3)nn12)c1ccccc1	Ki	'='	55.2	nM	CHEMBL4673199	RSC Med Chem	2021
CHEMBL4744151	CCOC(=O)CCCNc1nc2nn(C)cc2c2nc(-c3ccco3)nn12	Ki	'='	231	nM	CHEMBL4673199	RSC Med Chem	2021
CHEMBL4763391	CCOC(=O)C(CC(C)C)Nc1nc2nn(C)cc2c2nc(-c3ccco3)nn12	Ki	'='	398	nM	CHEMBL4673199	RSC Med Chem	2021
CHEMBL4782033	CCOC(=O)[C@H](Cc1ccc(O)cc1)Nc1nc2nn(C)cc2c2nc(-c3ccco3)nn12	Ki	'='	1302	nM	CHEMBL4673199	RSC Med Chem	2021
CHEMBL4757966	CCOC(=O)C(CO)Nc1nc2nn(C)cc2c2nc(-c3ccco3)nn12	Ki	'='	348	nM	CHEMBL4673199	RSC Med Chem	2021

CHEMBL4755552	CCOC(=O)C(C)Nc1nc2nn(C)cc2c2nc(-c3ccco3)nn12	Ki	'='	820	nM	CHEMBL4673199	RSC Med Chem	2021
CHEMBL4754809	CCOC(=O)C[C@H](Nc1nc2nn(C)cc2c2nc(-c3ccco3)nn12)C(=O)OCC	Ki	'='	445	nM	CHEMBL4673199	RSC Med Chem	2021
CHEMBL4759275	CCOC(=O)[C@@H](Cc1cc2ccccc2[nH]1)Nc1nc2nn(C)cc2c2nc(-c3ccco3)nn12	Ki	'='	889	nM	CHEMBL4673199	RSC Med Chem	2021
CHEMBL4792994	COC(=O)[C@@H]1CCCN1c1nc2nn(C)cc2c2nc(-c3ccco3)nn12	Ki	'='	428	nM	CHEMBL4673199	RSC Med Chem	2021
CHEMBL4758037	CCOC(=O)C(Nc1nc2nn(C)cc2c2nc(-c3ccco3)nn12)C(O)c1ccccc1	Ki	'='	376	nM	CHEMBL4673199	RSC Med Chem	2021
CHEMBL4763600	COc1ccc(N2CCOCC2)c2sc(NC(=O)N3CCC(OC)CC3)nc12	Ki	'='	33	nM	CHEMBL4732184	Eur J Med Chem	2021
CHEMBL4746896	COc1ccc(N2CCOCC2)c2sc(NC(=O)N3CCC(C(O)CF)CC3)nc12	Ki	'='	6.4	nM	CHEMBL4732184	Eur J Med Chem	2021
CHEMBL4760845	CC1(O)CCN(C(=O)Nc2nc3c(OCCF)ccc(N4CCOCC4)c3s2)CC1	Ki	'='	361	nM	CHEMBL4732184	Eur J Med Chem	2021
CHEMBL4748372	COc1ccc(N2CCOCC2)c2sc(NC(=O)N3CCC(OCCF)CC3)nc12	Ki	'='	23.9	nM	CHEMBL4732184	Eur J Med Chem	2021
CHEMBL4750049	COc1ccc(N2CCOCC2)c2sc(NC(=O)N3CCCCC3)nc12	Ki	'='	6	nM	CHEMBL4732184	Eur J Med Chem	2021
CHEMBL4742132	COc1ccc(N2CCOCC2)c2sc(NC(=O)N3CC=CCC3)nc12	Ki	'='	2.4	nM	CHEMBL4732184	Eur J Med Chem	2021
CHEMBL4783617	COc1ccc(N2CCOCC2)c2sc(NC(=O)N3CCC(O)CC3)nc12	Ki	'='	3.8	nM	CHEMBL4732184	Eur J Med Chem	2021
CHEMBL4745912	COc1ccc(N2CCOCC2)c2sc(NC(=O)N3CCC(O)(CCF)CC3)nc12	Ki	'='	9.4	nM	CHEMBL4732184	Eur J Med Chem	2021
CHEMBL4785362	COc1ccc(N2CCOCC2)c2sc(NC(=O)N3CCCC(F)C3)nc12	Ki	'='	6.4	nM	CHEMBL4732184	Eur J Med Chem	2021
CHEMBL4783723	COc1ccc(N2CCOCC2)c2sc(NC(=O)N3CCC(F)CC3)nc12	Ki	'='	4.9	nM	CHEMBL4732184	Eur J Med Chem	2021
CHEMBL4753657	COc1ccc(N2CCOCC2)c2sc(NC(=O)N3CCC(CF)CC3)nc12	Ki	'='	3.6	nM	CHEMBL4732184	Eur J Med Chem	2021
CHEMBL2105747	COc1ccc(N2CCOCC2)c2sc(NC(=O)N3CCC(C)(O)CC3)nc12	Ki	'='	3.9	nM	CHEMBL4732184	Eur J Med Chem	2021
CHEMBL4784681	COc1ccc(N2CCOCC2)c2sc(NC(=O)N3CCC(F)(CO)CC3)nc12	Ki	'='	3.6	nM	CHEMBL4732184	Eur J Med Chem	2021
CHEMBL4788835	COc1ccc(N2CCOCC2)c2sc(NC(=O)N3CCC(C(C)(O)CF)CC3)nc12	Ki	'='	37.9	nM	CHEMBL4732184	Eur J Med Chem	2021
CHEMBL4746388	COc1ccc(N2CCOCC2)c2sc(NC(=O)N3CCC(O)(CF)CC3)nc12	Ki	'='	2.8	nM	CHEMBL4732184	Eur J Med Chem	2021
CHEMBL4795658	COc1ccc(N2CCOCC2)c2sc(NC(=O)N3CCC(COCCF)CC3)nc12	Ki	'='	35.1	nM	CHEMBL4732184	Eur J Med Chem	2021
CHEMBL4787582	CC1=C(C(=O)OCc2ccccc2)C(c2cccs2)NC(=S)N1	IC50	'='	1000	nM	CHEMBL4765483	J Med Chem	2021
CHEMBL3091707	CCCOC(=O)C1=C(C)NC(=O)NC1c1ccco1	IC50	'='	1000	nM	CHEMBL4765483	J Med Chem	2021

CHEMBL183	CCCn1c(=O)c2[nH]c(C3CCCC3)nc2n(CCC)c1=O	Ki	'='	157	nM	CHEMBL4765483	J Med Chem	2021
CHEMBL113142	Nc1nc(NCCc2ccc(O)cc2)nc2nc(-c3ccco3)nn12	Ki	'='	1.9	nM	CHEMBL4765483	J Med Chem	2021
CHEMBL4849795	Nc1nc(Cc2ccccc2)cn2c1nc1ccccc12	EC50	'='	9.2	nM	CHEMBL4813923	Eur J Med Chem	2021
CHEMBL4855390	Nc1nc(-c2cccc(Br)c2)cn2c1nc1ccccc12	EC50	'='	10.1	nM	CHEMBL4813923	Eur J Med Chem	2021
CHEMBL4873408	CC(=O)Nc1ccc(-c2cn3c(nc4ccccc43)c(N)n2)cc1	IC50	'='	311.8	nM	CHEMBL4813923	Eur J Med Chem	2021
CHEMBL4862573	COc1cccc(-c2cn3c(nc4cc(F)c(F)cc43)c(N)n2)c1	IC50	'='	27	nM	CHEMBL4813923	Eur J Med Chem	2021
CHEMBL4853667	Nc1nc(-c2ccc([N+](=O)[O-])cc2)cn2c1nc1ccccc12	IC50	'='	71.9	nM	CHEMBL4813923	Eur J Med Chem	2021
CHEMBL4862534	Nc1nc(-c2ccc(Br)cc2)cn2c1nc1ccccc12	IC50	'='	38.4	nM	CHEMBL4813923	Eur J Med Chem	2021
CHEMBL4876739	Nc1nc(-c2ccc(F)cc2F)cn2c1nc1ccccc12	IC50	'='	33.7	nM	CHEMBL4813923	Eur J Med Chem	2021
CHEMBL4860395	Nc1nc(-c2ccc(Cl)cc2)cn2c1nc1ccccc12	IC50	'='	35.4	nM	CHEMBL4813923	Eur J Med Chem	2021
CHEMBL4872721	Nc1nc(-c2ccco2)cn2c1nc1ccccc12	IC50	'='	32.6	nM	CHEMBL4813923	Eur J Med Chem	2021
CHEMBL4857425	Nc1nc(CO)cn2c1nc1ccccc12	IC50	'='	149	nM	CHEMBL4813923	Eur J Med Chem	2021
CHEMBL4846299	Cc1ccc(-c2cn3c(nc4ccccc43)c(N)n2)cc1	IC50	'='	14.4	nM	CHEMBL4813923	Eur J Med Chem	2021
CHEMBL4858421	CC(=O)Nc1cccc(-c2cn3c(nc4ccccc43)c(N)n2)c1	IC50	'='	48.6	nM	CHEMBL4813923	Eur J Med Chem	2021
CHEMBL4846301	COc1cccc(-c2cn3c(nc4ccccc43)c(N)n2)c1	IC50	'='	10.1	nM	CHEMBL4813923	Eur J Med Chem	2021
CHEMBL4857237	Nc1nc(-c2ccc(C(F)(F)F)cc2)cn2c1nc1ccccc12	IC50	'='	26.5	nM	CHEMBL4813923	Eur J Med Chem	2021
CHEMBL4846381	N#Cc1ccc(-c2cn3c(nc4ccccc43)c(N)n2)cc1	IC50	'='	74.4	nM	CHEMBL4813923	Eur J Med Chem	2021
CHEMBL4849821	COc1ccccc1-c1cn2c(nc3ccccc32)c(N)n1	IC50	'='	83.1	nM	CHEMBL4813923	Eur J Med Chem	2021
CHEMBL4849986	Cc1c(-c2ccccc2)nc(N)c2nc3ccccc3n12	IC50	'='	113.7	nM	CHEMBL4813923	Eur J Med Chem	2021
CHEMBL4876950	COc1ccc(-c2cn3c(nc4ccccc43)c(N)n2)cc1	IC50	'='	51.2	nM	CHEMBL4813923	Eur J Med Chem	2021
CHEMBL4867327	Nc1nc(-c2ccc(OC(F)(F)F)cc2)cn2c1nc1ccccc12	IC50	'='	130.8	nM	CHEMBL4813923	Eur J Med Chem	2021
CHEMBL4850192	Nc1nc(-c2ccccc2)cn2c1nc1ccccc12	IC50	'='	46.5	nM	CHEMBL4813923	Eur J Med Chem	2021
CHEMBL4875468	CC(C)(C)c1ccc(-c2cn3c(nc4ccccc43)c(N)n2)cc1	IC50	'='	33	nM	CHEMBL4813923	Eur J Med Chem	2021
CHEMBL4877586	Nc1nc(-c2ccc(O)cc2)cn2c1nc1ccccc12	IC50	'='	81.3	nM	CHEMBL4813923	Eur J Med Chem	2021

CHEMBL4862394	N#Cc1cccc(-c2nc(N)n3nc(Cc4cccnc4)nc3c2-c2ccncn2)c1	Ki	'<='	10	nM	CHEMBL4825746	ACS Med Chem Lett	2021
CHEMBL4853681	N#Cc1cccc(-c2nc(N)n3nc(C(O)c4c(F)cccc4F)nc3c2C#N)c1F	Ki	'<='	10	nM	CHEMBL4825746	ACS Med Chem Lett	2021
CHEMBL4875506	N#Cc1cccc(-c2nc(N)c3nn(Cc4ncccc4F)nc3c2-c2ccncc2)c1	Ki	'<='	10	nM	CHEMBL4825746	ACS Med Chem Lett	2021
CHEMBL4872027	Cn1nccc1-c1c(-c2cccc(C#N)c2F)nc(N)c2nn(Cc3ccccn3)nc12	Ki	'<='	10	nM	CHEMBL4825746	ACS Med Chem Lett	2021
CHEMBL4851026	Cc1ncoc1-c1c(-c2cccc(C#N)c2)nc(N)c2nc(C(N)c3c(F)cccc3F)nn12	Ki	'<='	10	nM	CHEMBL4825746	ACS Med Chem Lett	2021
CHEMBL4865725	N#Cc1cccc(-c2cc3nc(C(O)c4c(F)cccc4F)nn3c(N)n2)c1F	Ki	'<='	10	nM	CHEMBL4825746	ACS Med Chem Lett	2021
CHEMBL4858144	Nc1nc(-c2ccc(F)cc2)c(-c2ccc(=O)[nH]c2)c2nc(Cc3ccc(F)cn3)nn12	IC50	'='	0.3	nM	CHEMBL4840384	ACS Med Chem Lett	2021
CHEMBL4867286	COc1ccc(Cc2nc3c(-c4ccc(=O)n(C)c4)c(-c4ccccc4)nc(N)n3n2)nc1	IC50	'='	7.72	nM	CHEMBL4840384	ACS Med Chem Lett	2021
CHEMBL4865474	Nc1nc(-c2ccccc2)c(-c2ccnnc2)c2nc(CC3CCCO3)nn12	IC50	'='	83.87	nM	CHEMBL4840384	ACS Med Chem Lett	2021
CHEMBL4870159	Cn1cc(-c2c(-c3ccccc3)nc(N)n3nc(Cc4ncccc4F)nc23)ccc1=O	IC50	'='	0.57	nM	CHEMBL4840384	ACS Med Chem Lett	2021
CHEMBL4873266	Cn1cc(-c2c(-c3ccccc3)nc(N)n3nc([C@H](O)c4ncccc4F)nc23)ccc1=O	IC50	'='	0.52	nM	CHEMBL4840384	ACS Med Chem Lett	2021
CHEMBL4876080	Cn1cc(-c2c(-c3ccc(F)cc3)nc(N)n3nc([C@H](O)c4ncccc4F)nc23)ccc1=O	IC50	'='	8.7	nM	CHEMBL4840384	ACS Med Chem Lett	2021
CHEMBL4862010	Nc1nc(-c2ccccc2)c(-c2ccnnc2)c2nc(Cc3ncccc3F)nn12	IC50	'='	9.07	nM	CHEMBL4840384	ACS Med Chem Lett	2021
CHEMBL4855908	Nc1nc(-c2ccc(F)cc2)c(-c2ccc(=O)[nH]c2)c2nc(CCC(F)(F)F)nn12	IC50	'='	0.74	nM	CHEMBL4840384	ACS Med Chem Lett	2021
CHEMBL4864129	Nc1nc(-c2ccccc2)c(-c2ccc(=O)[nH]c2)c2nc(Cc3ncc(F)cc3Cl)nn12	IC50	'='	0.29	nM	CHEMBL4840384	ACS Med Chem Lett	2021
CHEMBL4876056	Cc1cc(-c2c(-c3ccc(F)cc3)nc(N)n3nc(CN4CCCCC4)nc23)c[nH]c1=O	IC50	'='	5.72	nM	CHEMBL4840384	ACS Med Chem Lett	2021

Table S2: *Classification machine learning models developed for the different targets. RFC = Random Forest Classification; SVC = Support Vector Classification.*

Target	Bioassays	Low / High Threshold	Low / High in the initial dataset	ML algorithm	Accuracy	Cohen's Kappa	Precision (Low / High)	Recall (Low / High)
A_{2A}AR Antagonist	Binding (pKi values)	pKi ≥ 7	2991 / 2985	RFC	0.87	0.73	0.87 / 0.86	0.86 / 0.87
A_1AR Antagonist	Binding (pKi values)	pKi ≥ 6	1784 / 1050	SVC	0.80	0.59	0.71 / 0.87	0.79 / 0.81
A_3AR Antagonist	Binding (pKi values)	pKi ≥ 6	865 / 2001	SVC	0.84	0.62	0.71 / 0.90	0.79 / 0.86
A_{2B}AR Antagonist	Functional (pIC_{50} values)	pKi ≥ 6	272 / 196	RFC	0.91	0.82	0.92 / 0.90	0.94 / 0.88

***Table S3**: Regression machine learning models developed for A_{2A} AR Antagonist. RFR = Random Forest Regression; MAE = mean absolute error; RMSE = root mean squared error.*

Target	Bioassays	Number of data in the initial dataset	ML algorithm	Q^2	MAE	RMSE
**A_{2A}AR Antagonist**	Binding (pKi values)	4699	RFR	0.65	0.53	0.51
**A_{2A}AR Antagonist**	Binding (pIC_{50} values)	547	RFR	0.64	0.54	0.52

***Table S4**: Classification machine learning models developed for the different targets.*

Target	Bioassays	Low / High Threshold	Low / High in the initial dataset	ML algorithm	Accuracy	Cohen's Kappa	Precision	Recall
**A_{2A}AR Antagonist**	Binding (pKi values)	pKi ≥ 7	3002 / 2985	CatBoost Classifier	0.87	0.74	0.86	0.89
**A_1AR Antagonist**	Binding (pKi values)	pKi ≥ 6	1795 / 1050	Random forest Classifier	0.83	0.64	0.84	0.90
**A_3AR Antagonist**	Binding (pKi values)	pKi ≥ 6	876 / 2001	Random forest Classifier	0.86	0.66	0.88	0.93
**A_{2B}AR Antagonist**	Functional	pKi ≥ 6	272 / 196	Random forest	0.93	0.85	0.90	0.93

(pIC_{50} values)	Classifier

***Table S5**: Regression machine learning models developed for A_{2A} AR antagonists. MAE = mean absolute error; RMSE = root mean squared error.*

Target	Bioassays	Number of data in the initial dataset	ML algorithm	R^2	MAE	RMSE
A_{2A}AR Antagonist	Binding (pKi values)	4699	CatBoost Regressor	0.71	0.49	0.65
A_{2A}AR Antagonist	Binding (pIC_{50} values)	547	CatBoost Regressor	0.71	0.37	0.53

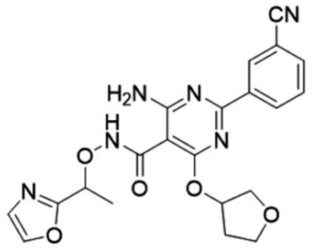

Ki (hA_{2A}) = 0.1 nM [3]

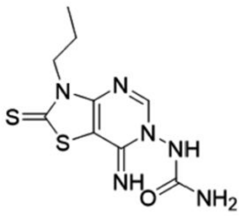

Ki (hA_{2A}) = 3.8 pM [4]

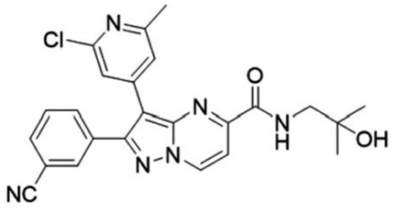

Ki (hA_{2A}) = 0.2 nM [5]

***Figure S1**: Structures of three known antagonists used as inception in REINVENT generative tool.*

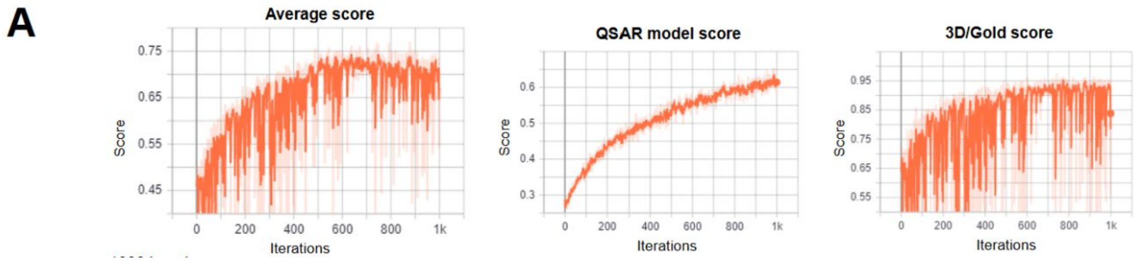

B

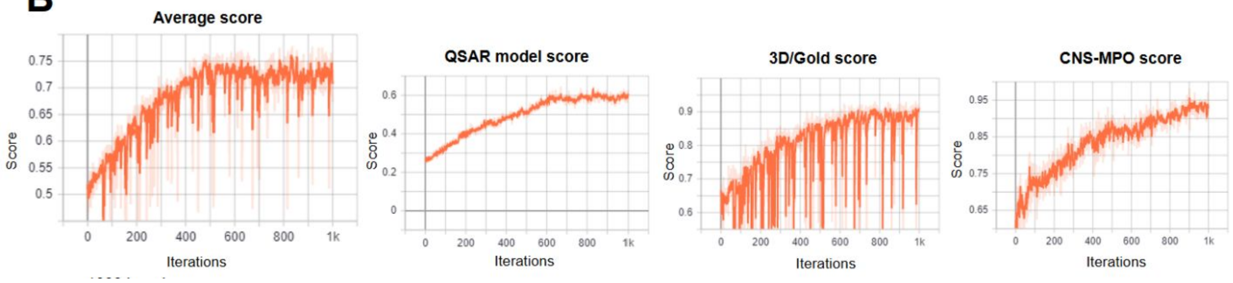

Figure S2: ***A***. *Graphs generated with TensorBoard illustrating the average score obtained by REINVENT at each iteration, using both the activity model and the 3D Gold component, and the individual contributions of each component.* ***B.*** *Graphs generated with TensorBoard illustrating the average score obtained by REINVENT at each iteration, using the activity model, the 3D Gold and the CNS-MPO component, and the individual contributions of each component.*

A

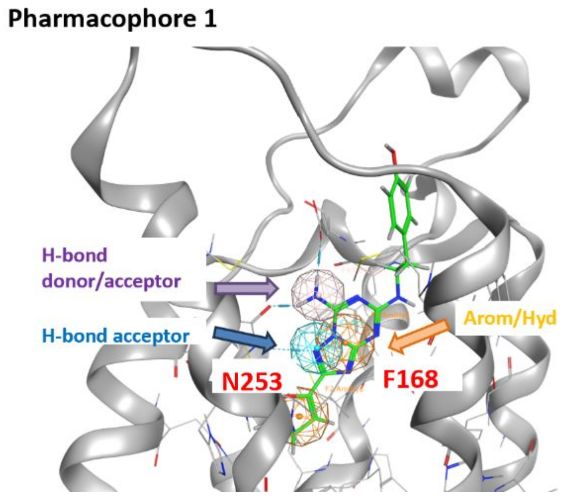

B

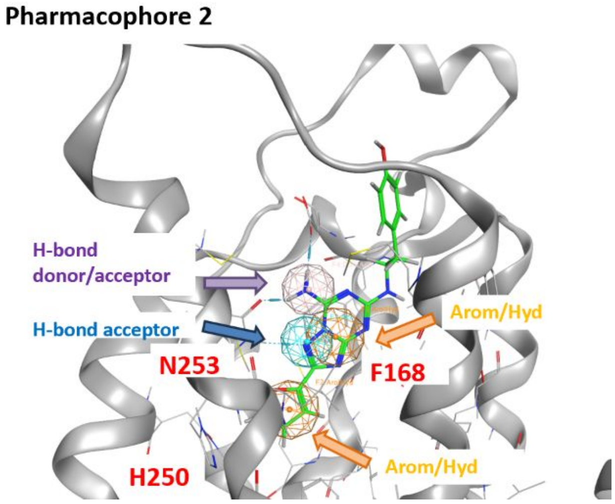

C

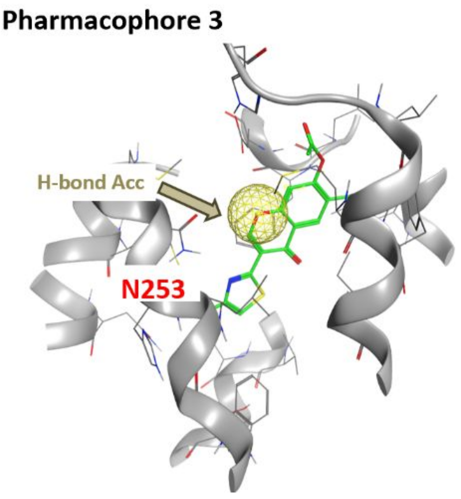

Figure S3: *Pharmacophores.* ***A.*** *First pharmacophoric filter, represented by binding mode of ZM-241385 (PDB ID: 4EIY);* ***B.*** *Second pharmacophoric filter, represented by binding mode of ZM-241385 (PDB ID: 4EIY);* ***C.*** *Third pharmacophoric filter, represented by binding mode of chromone ligand 4d (PBD ID: 6ZDR).*

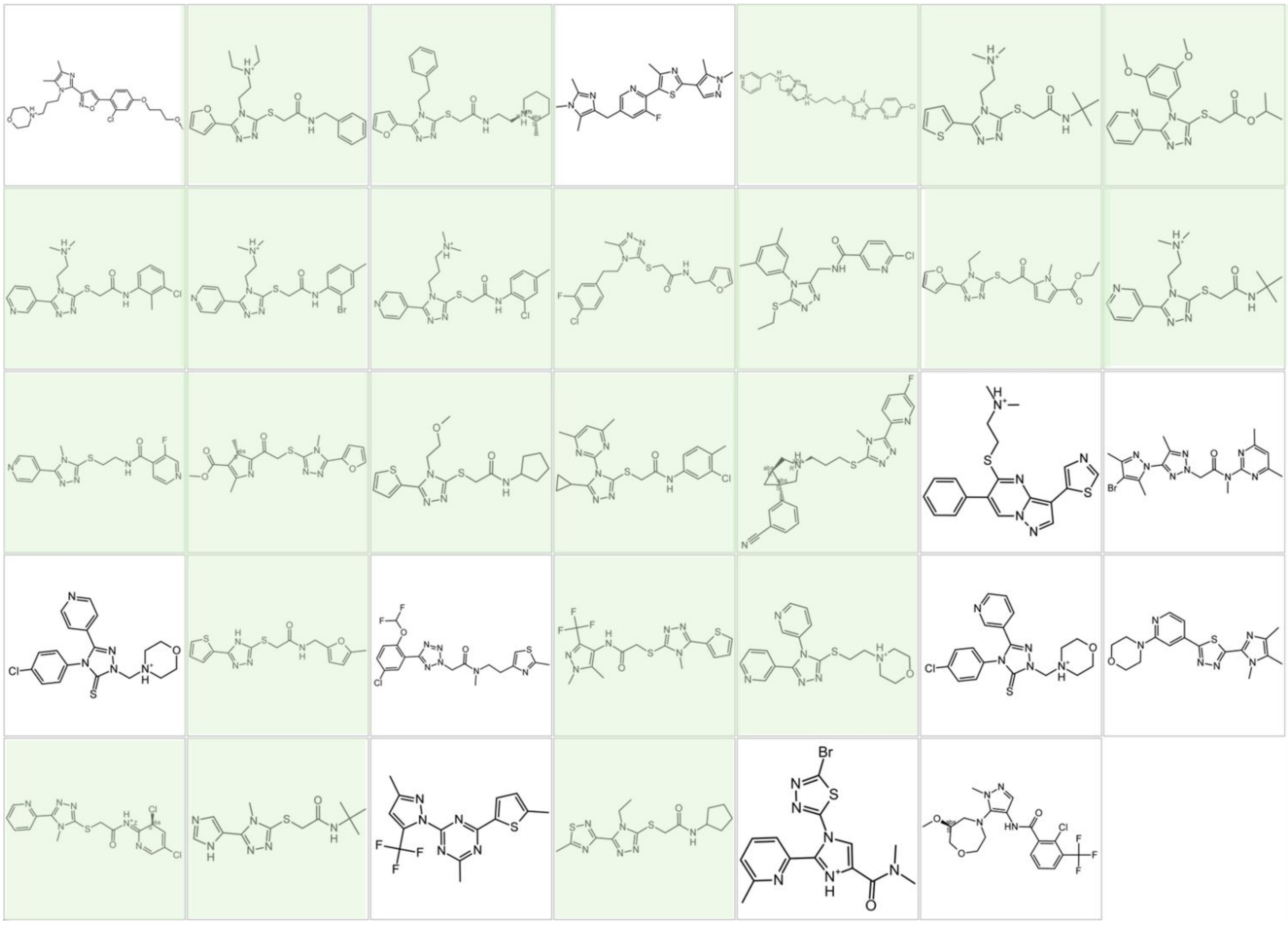

***Figure S4**: 34 molecules filtered by using the three pharmacophores listed in Figure S2. 23 molecules (squared in green) share the 1,2,4-triazolic core.*

Table S6: 100 best ranked molecules coming from the application of Bayesian Optimization by following the alternative filter pipeline to the first run of REINVENT.

SMILES	RANK	Source
Cc1cccc(-c2nn(CCC3)c3c2-c2cc(CF)nc(C)c2)n1	1	Model_Gold_CNS
CCc1cc(-c2nc3c(C)nc(C4CCC4)n3cc2)cc(C(N)=O)n1	2	Model_Gold_CNS
CCN(C(C)=C(C)N1N=Cc2cscc2)C1=S	3	Model_Gold
CC(C)N(CC1)CCN1c1cc(-c2c(-c3cc(C)nc(C)c3)n(CC#N)nc2)cc(F)n1	4	Model_Gold_CNS
Cc1nc(-c2c(-c3cc(C)nc(C)c3)nc(-c3nn(CCN(C)C)cc3)s2)cs1	5	Model_Gold_CNS
Cc1csc(-c2c(N3CCOCC3)ncc(-n3nccc3CC#N)c2)n1	6	Model_Gold_CNS
NC(c(cc1)c(N2CC(CC3)OC3C2)nc1-c1cc(F)cc(C#N)c1)=O	7	Model
CC(C)(C1)CN1c1nccc(-c2c(-c3cc(CF)nc(C)c3)n(C)nc2)n1	8	Model
Cc1nn(C)c(-c2c3scnn3c(-c3c(C)nc(C)s3)n2)c1	9	Model_Gold_CNS
Cc1cc(-c2cc(-c3cn(C4CCN(C)CC4)nc3)nn2-c(cc2)ccc2F)cc(C)n1	10	Model_Gold
Cc1nc(-c(c(C(N2CCCC2)=O)ccc2)c2F)nc(N)n1	11	Model_Gold_CNS
N#Cc1cc(-c2nc(C3SCC=N3)ns2)cnc1N(C1)CC1F	12	Model_Gold
CC(C)NC(Nc1c(-n2ncc(C)c2)nc(C2CC2)cc1C)=O	13	Model
CC(CC#N)N(C(C)=NN1c(nc(cc2)-n3c(C)nc(C)c3)c2Br)C1=O	14	Model_Gold
CCC(COc(nc(-n1nccc1C(F)F)nc1N(CC2)CC2(F)F)c1F)N	15	Model
FC(Oc1cccc(CNCc2nc(-c3cccs3)no2)n1)F	16	Model_Gold_CNS
CCN(CC)C(c1c(N)n(-c2ncc(C)c(-c3cccs3)n2)nc1)=O	17	Model_Gold_CNS
Cc1cccc(-c2c(-c3ccncc3)n(C(CC3)CCN3c3cc(C)ncc3)nc2)n1	18	Model_Gold_CNS
Cc(nc1C)c(-c2nc(C)cnc2N(C)C2CCN(C)CC2)n1-c(c(F)cc(C#N)c1)c1F	19	Model
Cc1ccc(C(NNC(Cc2coc3c2ccc(OC)c3)=O)=O)o1	20	Model_Gold_CNS
Cc1cccc(-c2c(-c3cc(OC)cc(OC)c3)nns2)n1	21	Model_Gold_CNS
CCc1nc(C)cc(-c2nc(-c3cc(C)nc(C)c3)ns2)c1	22	Model_Gold_CNS
Cc(c(-c1nc(N2C(C3)CN(C)C3C2)cc(C(N)=O)n1)c(cc1)OC)c1C#C	23	Model_Gold
COC(c(oc(-c1nc(C2CCOCC2)ccc1)c1)c1-c1nccs1)O	24	Model_Gold_CNS
Cc1cc(C)nn1CCN(C)Cc(cc1-c2cn(C)nc2)c(C)n1-c1cc(F)ncc1	25	Model_Gold_CNS
S=C(N(CN1CCOCC1)N=C1c2ncccc2)N1c1ccncc1	26	Model_Gold_CNS
NS(c(cc1)ccc1C(N1c(cc2)ccc2F)=NNC1=S)(=O)=O	27	Model_Gold
Cc(cc1C(F)F)nn1-c(nc1C)nc(N(CC2)CCC2(CC#N)N)c1Br	28	Model
Cc(cc1)ncc1-c(nc(N1CCC1)nc1)c1-c1ccns1	29	Model_Gold
Cc(n(C)nc1C)c1-c1cccc(N2N=C(C)N(CCS(C)(=O)=O)C2=O)c1	30	Model_Gold_CNS
Cc1c(-c2nc(C)nc(-c(ccc(OC)c3)c3OC)c2)sc(C)n1	31	Model_Gold_CNS
CC(C)C(C(N)=O)NC(c(nc1C)c(C)n1-c(cccc1)c1OC)=O	32	Model_Gold
CC(CCC1)(CN1c(c(-c1cn(C)nc1C)c1)ncc1C(NC1(CO)CC1)=O)F	33	Model

Cc1cc(C(N)=O)cnc1-c1cccc(-c2nc(CCN(C)C3)c3c(CF)n2)c1	34	Model_Gold_CNS
NC(N(CCCc1c2)c1ncc2-c1c(-c(cc2)ccc2F)n(CC#N)nc1)=O	35	Model_Gold
CCCN1CCN(CN(C2=S)N=C(c3nccnc3)N2c2ccccc2)CC1	36	Model_Gold_CNS
Cc(cc(cc1)-c2c(-c3cnc(N(CCC4)CC4N)s3)nc(C#N)n2C)c1F	37	Model_Gold
Cc1nc(CN(CC2)c3nccc(-c4nn(CCN5CCOCC5)cc4)c3)c2s1	38	Model_Gold_CNS
Cc1csc(NC(c2c(COCC3)n3c(-c3cc(F)ccc3)n2)=O)n1	39	Model_Gold
Cc(n(-c1nn(CC(N(C)c2nc(C)cc(C)n2)=O)nc1C)nc1C)c1Br	40	Model
CCN(C(c1cccc(S(NCc2ccco2)(=O)=O)c1)=NN1)C1=S	41	Model_Gold_CNS
Cc(cc1)ncc1-c1c(-c2ncccc2)c(-c2ccco2)ncn1	42	Model_Gold_CNS
Cc(nc1C)cc(-c2cccc(-c(cc3)ncc3OC)n2)c1O	43	Model_Gold_CNS
CCc1nccc(-c(c(-c2cc(C)nc(C)c2)c2)nc(C)c2C#N)c1	44	Model_Gold_CNS
Cc1nc(-c2nc(-c3cc(CF)nc(C)c3)nc(C)n2)cs1	45	Model_Gold_CNS
CCCN(CCC)CC(Nc1cc(C)nn1-c1nc(C)cc(C)n1)=O	46	Model_Gold_CNS
Cc1cccc(-c2cc(-c3ccnn3C)c(-c3nc(C)ccc3)n2CCN(C)C)n1	47	Model_Gold_CNS
Cc1cc(-c2n[nH]cc2-c2nc(C)nc(C)c2)cc(C)n1	48	Model_Gold_CNS
CC(CC1)(CN1c1c(-n2cnc(C)c2)nc(C2CCN(Cc3cnc(C)n3C)CC2)cc1)F	49	Model_Gold
Cc1cccc(-c2nc(C#N)c(C#N)nc2-c(cc2)ccc2F)n1	50	Model_Gold_CNS
CCNCc1cc(-c2nc(-c3nc(C)ccc3)cs2)c(SC)s1	51	Model_Gold_CNS
CC(C)(C1)OCCN1c(cc1)ncc1-c1c(C)ncc(-c2cn(C3CCN(C)CC3)nc2)c1	52	Model_Gold_CNS
COc(cc1)ccc1N(C(c1ncncc1)=NN1CN2CCOCC2)C1=S	53	Model_Gold_CNS
Cc(cc(c1c2)N3CCC3)nn1nc2-c1c(-c(cc2)nc(C)c2F)n(C)nc1	54	Model
CC(COC(CN(C)C)C1)N1c1cc(-c2cc(C3=CCOS3)c(C)nc2C)ncn1	55	Model_Gold
CC(C)N(C)C(CC1)CN1c(nc(-c1c(C)ncc(-<br>c2c(C)n(C)nc2)c1)c(C#N)c1)c1F	56	Model
CNCc(cc(cc1)F)c1-c1ncncc1-c(cc1)ccc1C#N	57	Model_Gold_CNS
Cc(cc1)nc(CC#N)c1-c1nc(C)c(CN=C2C=CN(CCCOC)C=C2)cc1	58	Model_Gold
CC(C)C(CO)NCc(nc(cc1)-c(ccc(F)c2)c2OC)c1F	59	Model_Gold_CNS
Cc1c(CN2CCOCC2)sc(-c(cc(-c2cc(C)nc(C)c2)nc2)c2F)n1	60	Model_Gold_CNS
Cc1c(C)nc(CNC(N(C)c(cc(cc2)-c3nc(C)ncc3)c2Cl)=O)c(C)n1	61	Model_Gold
Cc1csc(-c2nnc(CSc3nc(C)nc(C)c3)cc2)n1	62	Model_Gold_CNS
CC(C)NC(Cn1c(-c(cc2)cc(-c3cc(C)nc(C)c3)c2OC)nc(C)c1C)=O	63	Model_Gold_CNS
CC(C1)N(C)C(C)CN1c(nc1)nc(-c2cc(C)ncc2)c1-c1nc(C)ccc1	64	Model_Gold_CNS
CN(C)CCn(cc1-c2cc(-c3cc(F)cnc3)ncc2)nc1-c1ccncc1	65	Model_Gold
CCOC(c1c(C)oc(C(N2C)=C(N)N(Cc3ccccc3)C2=O)c1)=O	66	Model_Gold
CC(C)C(CO)NCc(nc(cc1)-c(cc(cc2)C#N)c2OC)c1F	67	Model_Gold
Cc1cc(C)nn1-c(c1c(N)nc2)csc1c2NC(N(C)C)=O	68	Model_Gold
CCCCOCCCN(C(C=Cc1ncccc1)=NN1)C1=S	69	Gold
CC(C)(C(N)=O)N(C)Cc(cc(cc1)-c2cnc3n2cccc3C(F)(F)F)c1OC	70	Model_Gold
CC(C)n1nc(C)c(-c2nnc(-n3nc(C)cc3C)s2)c1C	71	Model_Gold_CNS

Cc(ccc(C(OC)=O)c1)c1NC(NC(c1ccco1)=O)=S	72	Gold
Cc(cc1)nc(C)c1C#Cc1c(C)nc(C)nc1-c1cc(C)nc(C)c1	73	Model_Gold_CNS
Cc1coc(-c2nc(CN(C)c3ncnc(C)c3)c(C)o2)n1	74	Model_Gold_CNS
CCN(Cc1ncc(-c2nc(C)ccc2)c(-c2nc(C)ccc2)n1)C(C)CN1CCCC1	75	Model_Gold_CNS
NC(Cc1ccccc1)(CC1)CCN1c(ncnc1-c(ccc(F)c2)c2F)c1C#N	76	Model
Cc1ncc(COC)n1CCCN(CC1)CCN1c1cc(C#N)cc(C(F)(F)F)c1C#N	77	Model_Gold
CC(Cn1nc(C)nc1C)NC(c1csc(-c2nc(C)ccc2)n1)=O	78	Model_Gold_CNS
Cc1cc(-c2nn(CCN(C)C)c(-c3cc(-n4cnc(F)c4)cnc3)c2)ncc1	79	Model_Gold
Cc(cc1)nn1-c1nccc(-c2nc(CN3CCN(C)CC3)cs2)n1	80	Model_Gold_CNS
NC(c(c1c2cccc1)cn2-c1cc(C#N)cnc1N(CC1)CC1(F)F)=O	81	Model
CC1OC(C)CN(Cc(cc2)c(C)nc2-c2csc(-c3nc(C)c(C)nc3)n2)C1	82	Model_Gold_CNS
Cc(cc(-c1cnc(C#N)n1C)c(F)c1)c1NC(NC(CC1)CS1(=O)=O)=S	83	Model_Gold
Cc(nc1C)c(C)nc1-c1cccc(-c(ccc(COC)c2)c2OC)c1O	84	Model_Gold_CNS
Cc1ccnc(-c2cncc(-c(cc3CCC4)cnc3N4C(N)=O)c2)c1F	85	Model_Gold
CC(C)C(CO)NCc(nc(cc1)-c2cc(C#N)ccc2)c1F	86	Model_Gold_CNS
N#CCc(c(-c1nc(-c(cc2)cnc2C#N)cnc1)cc(F)c1)c1Cl	87	Model_Gold
COc(cc1)cc(S2)c1N(CCCN=C=S)C2=N	88	Model_Gold_CNS
CN(C)C=Cc1c(-c2ccncc2)sc(C(C=CO2)C2=O)n1	89	Model_Gold
Cc1cc(C(N=C(N2C)N3CC(c(cc(cc4)F)c4OC)OCC3)=CC2=O)cc(C)n1	90	Model_Gold_CNS
CCN(C=C(C(C)=C1)c(ccc(C)n2)c2Oc2c(C)nc(C)cc2)C1=O	91	Model_Gold_CNS
Cc1ccc(-c2cn(C)nc2)nc1Cc1nccc(OCCN2CCOCC2)c1	92	Model_Gold_CNS
NC(c1c(-n2c(CF)ncc2)n(-c2ccc(C(F)(F)F)cc2)nc1)=O	93	Model
Cc(cc1)nnc1-c(cc1)nc(-c2nn(CC(F)(F)F)c(C3CCN(C)CC3)c2)c1F	94	Model_Gold_CNS
CC1(CNC(c(cc2)cc(C#N)c2-c2cc(C)cc(C)c2)=O)CN(C)CC1	95	Model_Gold_CNS
O=C(c(cc1-c2cncc(F)c2)cnc1N(CC1)SCC1F)NCc1cnccc1	96	Model_Gold
CC(C)n1nc(C)nc1-c1cc(C(N)=O)c(-c(ccc(C#N)c2)c2F)n1C	97	Model
Cc1c(CCc2nn3c(C)cnc(C)c3n2)ncn1CC#N	98	Model_Gold
Cc1c(CC(NCc2c(C)nc(C)s2)=O)c(C)nn1CCc1nc(C)nc(C)c1	99	Model_Gold_CNS
Cc1nc(CNC(c2c(C)ncc(-c3nc(C)ncc3)c2)=O)cs1	100	Model_Gold_CNS

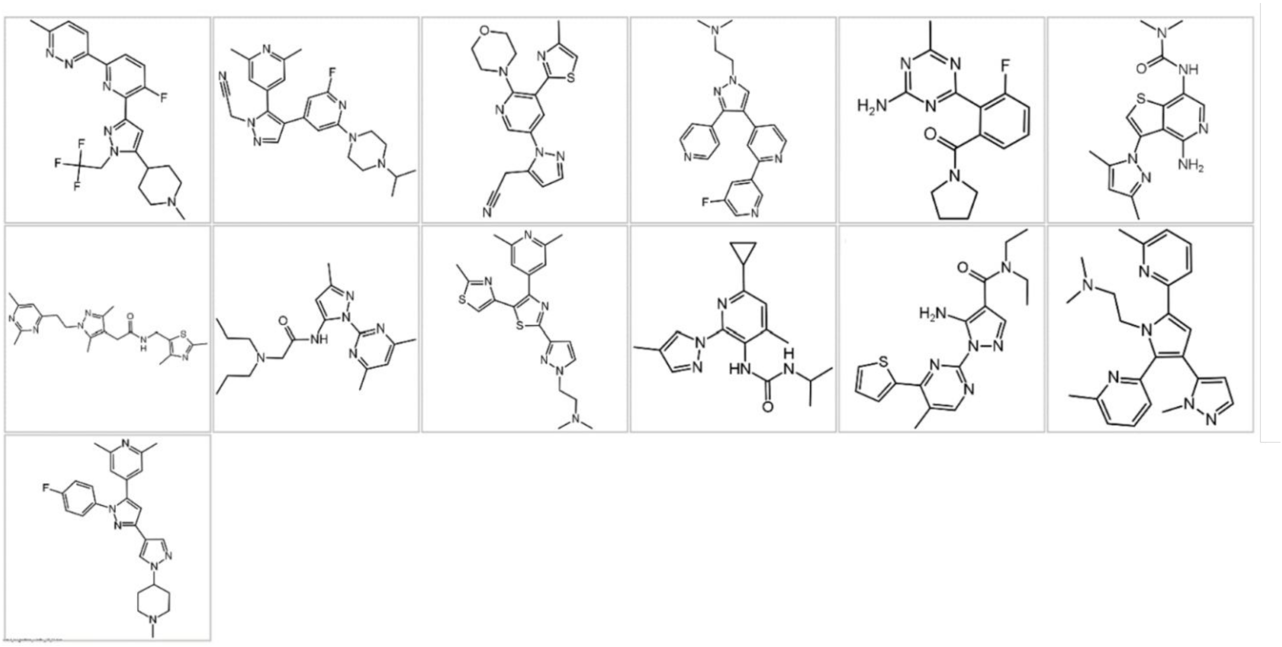

Figure S5: *13 molecules selected for visual inspection of ranking value for BO analysis. Rank values are comprised between 0 and 100 (among the 100 starting molecules), the best the molecule is ranked basing on predicted activity, the nearest the value is to 0.*

PAMPA: Data elaboration

The permeability coefficient (Pe), expressed in cm/s, was calculated with the following formula:

$$Pe = \frac{Vd * Vr}{(Vd + Vr)St} ln \frac{100 * Vd}{100 * Vd - \%T(Vd + Vr)}$$

$$\%T = \frac{Vr * Ar}{Ad0 * Vd} * 100$$

$$\frac{Vd * Vr}{(Vd + Vr)St} = 0{,}0000236$$

Vd and Vr are the volumes of the donor and acceptor solutions (0.18 cm^3), S is the membrane area (0.266 cm^2), t is the incubation time (2.30 h = 9000 s), Ar is the absorbance in the acceptor compartment after incubation and Ad0 is the absorbance in the donor compartment before incubation. Absorbances were the average absorbance value between the three wells for each identified maximum and minimum. The *Pe* of each compound was calculated for each wavelength, together with the average of these values with the corresponding standard deviation (SD). The results are expressed as the mean of the Pe ± SD of the two tests. The experimental data obtained for the known drugs were then correlated with the permeability data reported in the literature using Microsoft® Excel® for Microsoft 365 MSO (Office 2021). The linear correlation between the experimental and literature permeability values of known drugs was used to classify the compounds according to their ability to cross the BBB by passive permeation: CNS+ (permeable), CNS- (non-permeable) and CNS+/- (borderline). The straight line was designed using the values obtained from one of the two experiments performed, in order to ensure the closest value to literature.

Table S7: *values used for the generation of the calibration line.*

Drug	Bib. *Pe* (10^{-6} cm s^{-1})	Exp. *Pe* (10^{-6} cm s^{-1}) ± SD
Caffeine	1.3	1.6 ± 2.3
Desipramine	12	6.0 ± 2.3
Enoxacine	0.8	2.2 ± 0.3
Ofloxacine	0.8	1.9 ± 0.8
Piroxicam	2.5	2.4 ± 1.2
Promazine	8.8	7.5 ± 6.0
Verapamil	16	5.8 ± 3.0

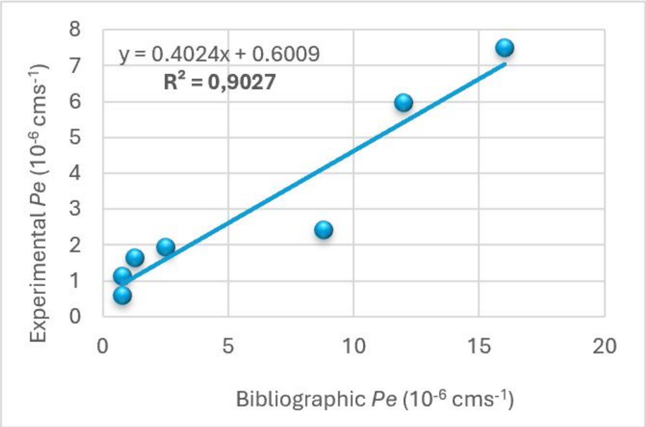

Figure S6: *PAMPA calibration line (exp. Pe = y), (bib. Pe = x) based on known drugs. Pe CNS+ = 2.2105 * 10^{6} cm/s; Pe CNS- = 1.4057 x 10^{6} cm/s.*

Table S8: *Apparent Permeability of synthesised compounds* ***(13,14,16-18)***

Compound	Apparent PE	SD
**13**	4.26146	0.27939
**14**	7.48585	4.01435
**16**	3.0832	0
**17**	9.88601	3
**18**	5.29813	0.83159

Table S9: The maximum Tanimoto similarity coefficient between the compound fingerprints and those in the specified training datasets was computed using a KNIME node. The computations are based on the CDK toolkit.

Compound	Reinvent Run	A2A training<br>Tanimoto similarity	Adenosine (A2A, A2B, A1, A3) training<br>Tanimoto similarity	A2A ligands<br>Tanimoto similarity
4	First	0.53	0.54	0.54
5	First	0.50	0.50	0.46
6	First	0.56	0.58	0.56
7	First	0.57	0.57	0.56
8	First	0.51	0.51	0.50
9	First	0.56	0.56	0.57
10	First	0.56	0.56	0.54
11	First	0.56	0.56	0.55
12	First	0.55	0.55	0.49
13	First run (second selection)	0.61	0.61	0.57
16	Second	0.65	0.69	0.63
17	Second	0.62	0.62	0.46

18	Second	0.74	0.74	0.59

Table S10: *Details on generative setup*

Run	Scoring Function	Component_name	Weight	Transformation_type	Reinforcement_learning parameters				Number of cores*	Run time for 1000 iteration
					Batch_size	Learning_rate	n_ahc_samples	Sigma		
**First**	Model	activity model	1	no_transformation	128	0.0001	Not applicable	128	16	1 hour
**First**	Gold	3D/Gold	1	Sigmoid (high : 65.0, k: 0.25, low : 50)	128	0.0001	Not applicable	128	64	8 days
**First**	Model_Gold	activity model<br>3D/Gold	1<br>1	no_transformation<br>Sigmoid (high : 65.0, k: 0.25, low : 50)	128	0.0001	Not applicable	128	64	5 days
**First**	Model_Gold_CNS	activity model<br>3D/Gold<br>CNS-MPO	1<br>1	no_transformation<br>Sigmoid (high : 65.0, k: 0.25, low : 50)<br>Sigmoid (high : 4, k: 0.2, low : 3)	128	0.0001	Not applicable	128	128	3 days
**Second**	Model_5xGold_CNS	activity model<br>3D/Gold<br>CNS-MPO	1<br>5<br>1	no_transformation<br>Sigmoid (high : 65.0, k: 0.25, low : 55)<br>Sigmoid (high : 4, k: 0.2, low : 3)	128	0.0001	64	128	128	3 days
**Second**	Model_10xGold_CNS	activity model<br>3D/Gold<br>CNS-MPO	1<br>10<br>1	no_transformation<br>Sigmoid (high : 65.0, k: 0.25, low : 55)<br>Sigmoid (high : 4, k: 0.2, low : 3)	128	0.0001	64	128	128	4 days

* Calculation were performed on AMD EPYC 7763 64-Core Processor

Scheme S1: Synthesis of 1,2,4-Triazole derivatives

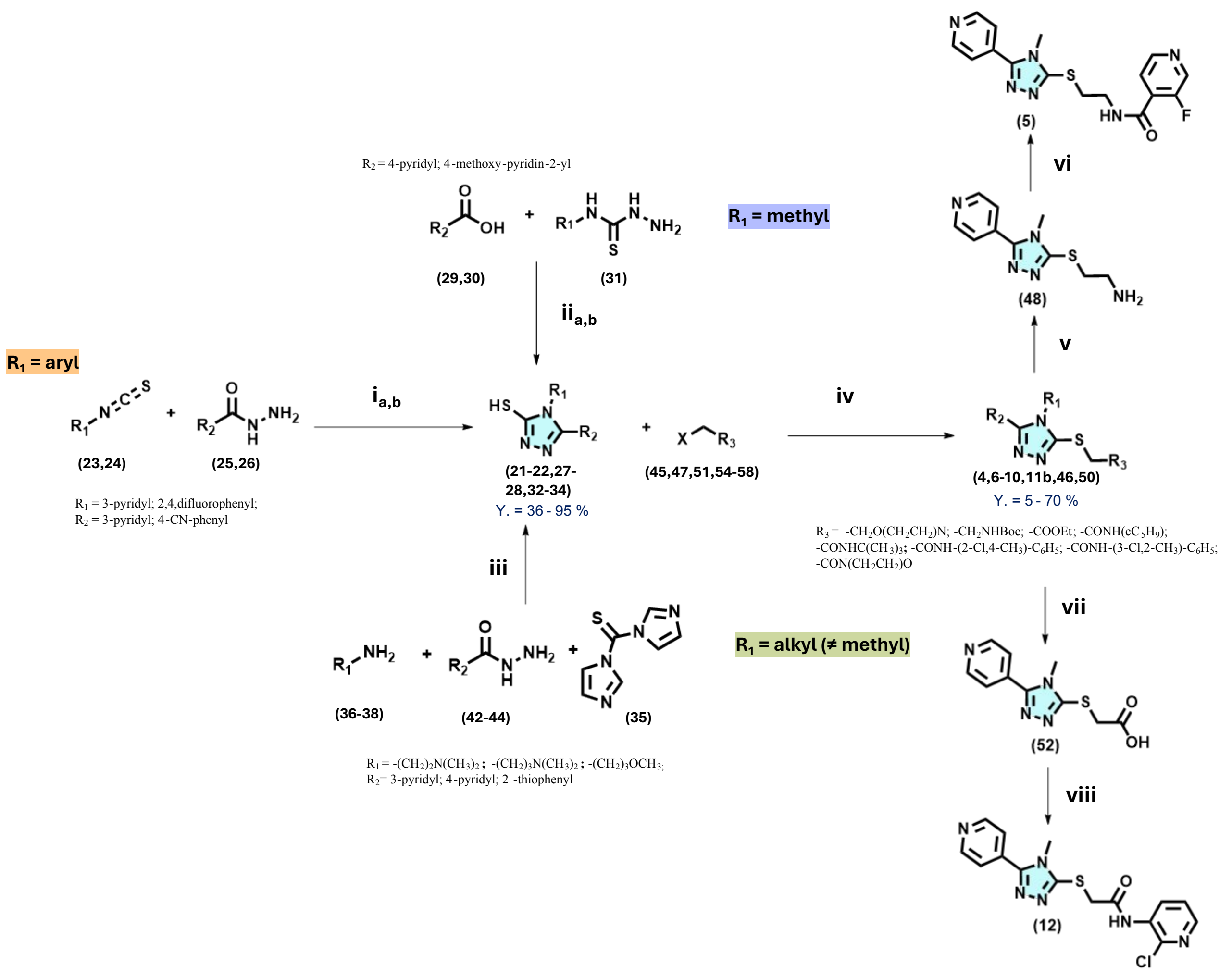

i_a) NaOH 2M, EtOH, reflux, i_b) HCl 37%; ii_a) T_3P, DIPEA, DMF, 0 ˚C, NaOH 4M pH ~ 11, RT -> 80 ˚C, ii_b) HCl 37%; iii) 2-MeTHF, 130 ˚C, MW; iv) K_2CO_3, MeOH / Acetone, 50 ˚C or KOH 4M, EtOH, reflux; v) TFA, DCM, RT; vi) 3-fluoroisonicotinoyl chloride, DIPEA, DMF, from 0˚C -> RT; vii) NaOH 4M, RT; viii) 2-chloropyridine-3-amine, CPME, N-methylimidazole, TCHF, RT.[3-5]

Scheme S2: Synthesis of pyrazole-pyrimidine derivatives

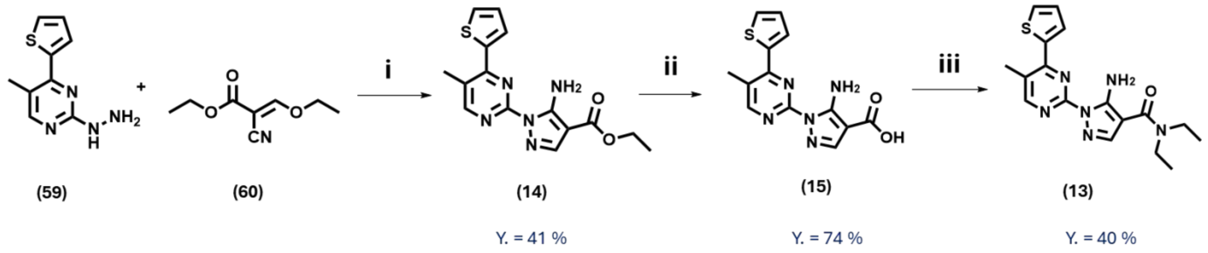

i) EtOH, reflux; ii) LiOH 3 M, RT; iii) $(CH_2CH_3)_2NH$, HBTU, DIPEA, DMF, 50 ˚C.[6-8]

Scheme S3: Synthesis of 2-amino pyrimidine derivatives through Suzuki-Miyaura reaction

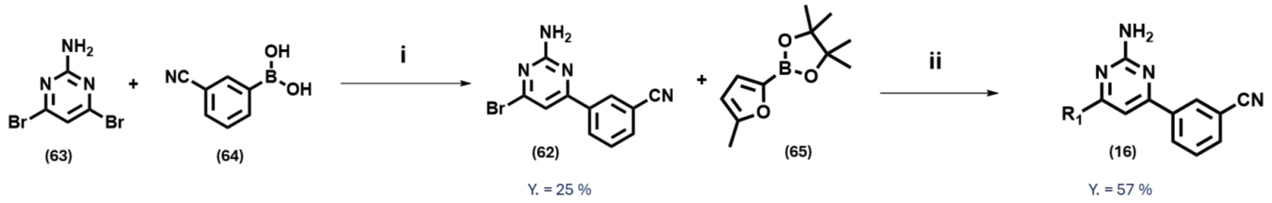

i) $Pd(PPh_3)_4$, Na_2CO_3 2M, EtOH /toluene (3 : 7), reflux; ii) $Pd(PPh_3)_4$, Na_2CO_3, dioxane / H_2O (3 : 1), reflux.[9]

Scheme S4: Synthesis of 2-amino pyrimidine derivatives through cyclization reaction

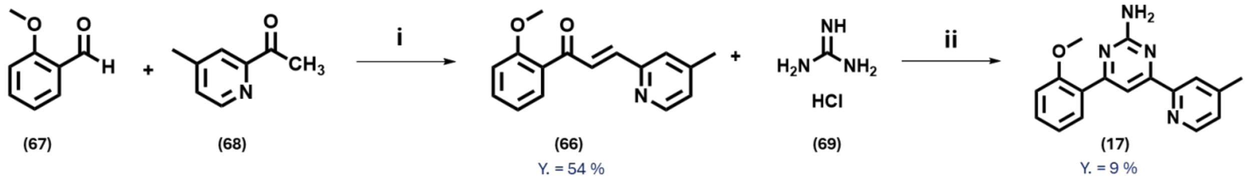

i) KOH 85 % (w/w MeOH / H_2O 6 : 1), 0 ˚C; KOH 50 % (w/w H_2O), EtOH, reflux; ii) H_2O_2 30 % (w/w H_2O), reflux.[10]

Scheme S5: Synthesis of 2-amino pyridine derivatives

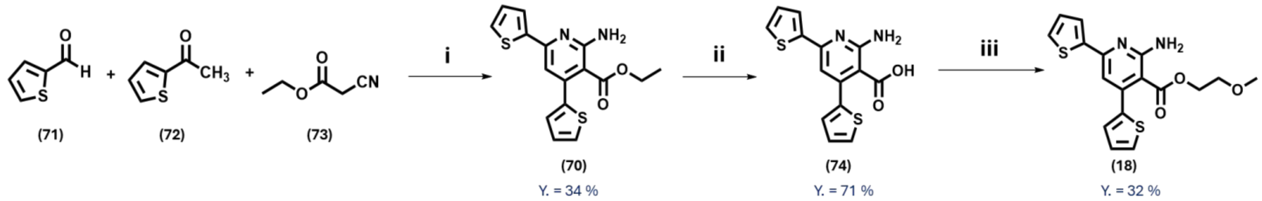

i) NH_4OAc, toluene, reflux; ii) NaOH 4M, EtOH, reflux; iii) 2-bromomethoxy ethane, K_2CO_3, DMF, RT.[11]

References

(1) Zdrazil, B.; Félix, E.; Hunter, F.; Manners, E.; Blackshaw, J.; Corbett, S.; Marleen De Veij; Ioannidis, H.; Méndez, D.; Mosquera, J. F.; María Paula Magariños; Bosc, N.; Arcila, R.; Tevfik Kizilören; Gaulton, A.; A. Patrícia Bento; Adasme, M. F.; Monecke, P.; Landrum, G. A.; Leach, A. R. The ChEMBL Database in 2023: A Drug Discovery Platform Spanning Multiple Bioactivity Data Types and Time Periods. *Nucleic Acids Research* 2023, *52* (D1). https://doi.org/10.1093/nar/gkad1004.

(2) Gabrielson, S. W. SciFinder. *Journal of the Medical Library Association* 2018, *106* (4). https://doi.org/10.5195/jmla.2018.515.

(3) Yuan, C.; Yan, J.; Song, C.; Yang, F.; Li, C.; Wang, C.; Su, H.; Chen, W.; Wang, L.; Wang, Z.; Qian, S.; Yang, L. Discovery of [1,2,4]Triazole Derivatives as New Metallo-β-Lactamase Inhibitors. *Molecules* 2019, *25* (1), 56. https://doi.org/10.3390/molecules25010056.

(4) Cremonesi, S.; Micheli, F.; Semeraro, T.; Tarsi, L. Dopamine D3 Receptor Antagonists Having a Bicyclo Moiety, February 9, 2017. WO2017021920A1.

(5) Kaul, S.; Alkaied, N.; Shanthi, S.; Cianciulli, A.; Micheli, F.; Semeraro, T.; Trist, I. M. L. Antagonists of GPR39 Protein, November 4, 2021. WO2021222858.

(6) Siddesh Mb; Basavaraj, P.; Thriveni, K. S.; Sandeep, C. “Synthesis of Polynuclear Pyrimidine Derivatives and Their Pharmacological Activities.” *Heterocycles* 2014, *4* (4), 503–514.

(7) Li, H.; Yuan, J.; Bakthavatchalam, R.; Hodgetts, K., J.; Mao, J.; Wustrow, D., J.; Guo, Q. 5 Membered Heterocyclic Amides and Related Compounds, January 22, 2009. WO2009012482A3.

(8) Min, J.; Scott, D. C.; Bhasin, D.; Schulman, B. A.; Syngh, B.; Hammill, J. T.; Guy, K. R. Methods and Compositions of Inhibiting DCN1-UBC12 Interaction, March 23, 2017. WO2017049295A1.

(9) Sagong, H. Y.; Bauman, J. D.; Patel, D.; Das, K.; Arnold, E.; LaVoie, E. J. Phenyl Substituted 4-Hydroxypyridazin-3(2*H*)-Ones and 5-Hydroxypyrimidin-4(3*H*)-Ones: Inhibitors of Influenza a Endonuclease. *Journal of Medicinal Chemistry* 2014, *57* (19), 8086–8098. https://doi.org/10.1021/jm500958x.

(10) Varga, L.; Nagy, T.; Kövesdi, I.; Benet-Buchholz, J.; Dormán, G.; Ürge, L.; Darvas, F. Solution-Phase Parallel Synthesis of 4,6-Diaryl-Pyrimidine-2-Ylamines and 2-Amino-5,5-Disubstituted-3,5-Dihydro-Imidazol-4-Ones via a Rearrangement. *Tetrahedron* 2003, *59* (5), 655–662. https://doi.org/10.1016/S0040-4020(02)01560-0.

(11) Abou-Elkhair, R. A. I.; Moustafa, A. H.; Haikal, A. Z.; Ibraheem, A. M. Synthesis and Biological Evaluation of 2-Oxonicotinonitriles and 2-Oxonicotinonitrile Based Nucleoside Analogues. *European Journal of Medicinal Chemistry* 2014, *74*, 388–397. https://doi.org/10.1016/j.ejmech.2013.12.055.

Compounds' spectra

Compound 4

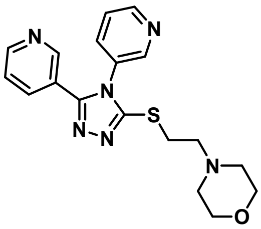

^{1}H NMR (500 MHz, DMSO-d_6) δ 8.75 (dd, J = 4.8, 1.5 Hz, 1H), 8.70 (dd, J = 2.5, 0.6 Hz, 1H), 8.60 (dd, J = 4.9, 1.6 Hz, 1H), 8.57 (dd, J = 2.2, 0.8 Hz, 1H), 8.01 (ddd, J = 8.0, 2.5, 1.5 Hz, 1H), 7.74 – 7.69 (m, 1H), 7.62 (ddd, J = 8.1, 4.8, 0.6 Hz, 1H), 7.42 (ddd, J = 8.0, 4.9, 0.8 Hz, 1H), 3.55 – 3.50 (m, 4H), 3.38 - 3.35 (m, 2H), 2.63 (t, J = 6.9 Hz, 2H), 2.40 – 2.33 (m, 4H).

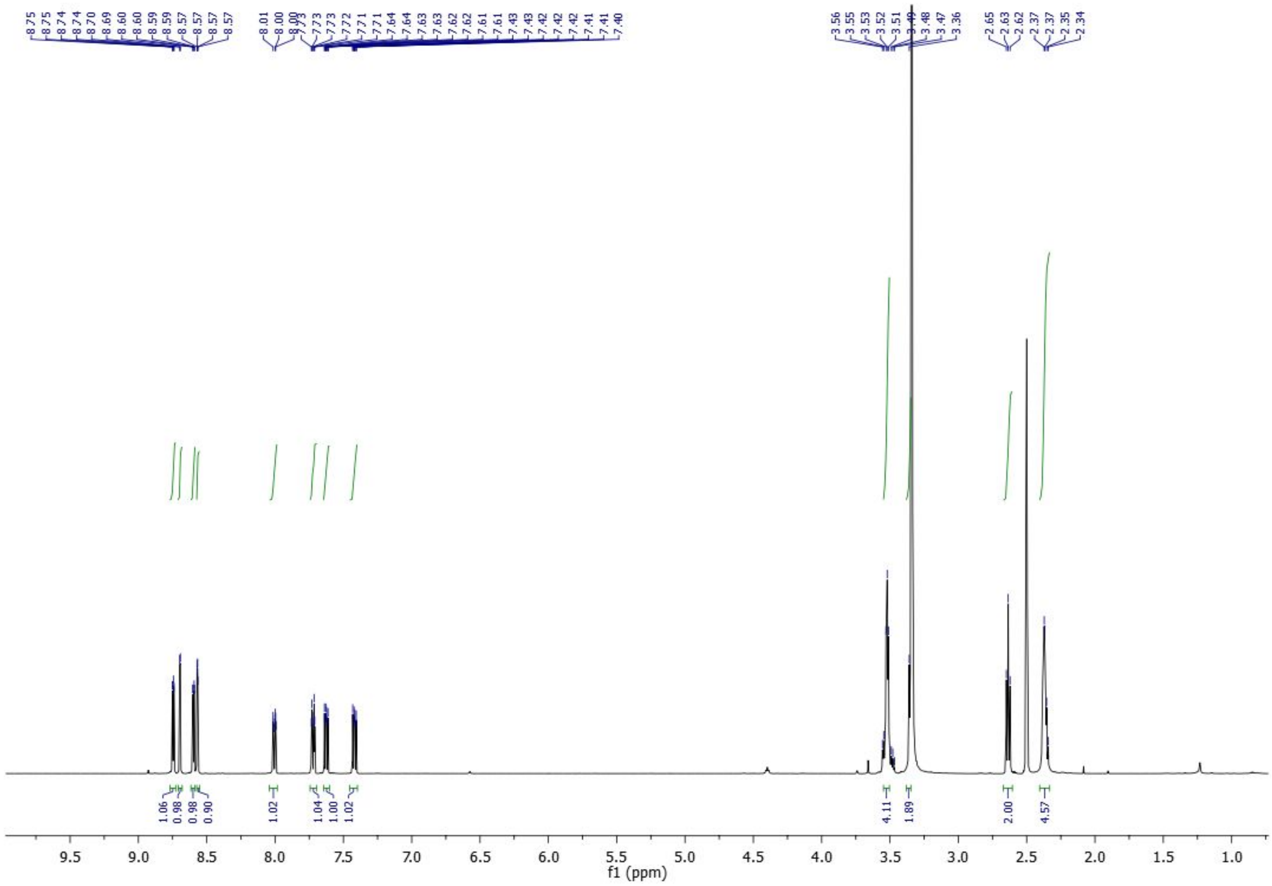

HSQC, HMBC (Bruker 500 MHz, DMSO-d_6) δ 152.87 (C), 152.47 (C), 151.19 (C), 150.93 (CH), 150.74 (C), 150.52 (CH), 148.46 (CH), 148.25 (CH), 135.64 (CH), 135.58 (CH), 124.46 (CH), 123.45 (CH), 65.84 (2 CH_2), 56.79 (CH_2), 52.67 (2 CH_2), 29.64 (CH_2).

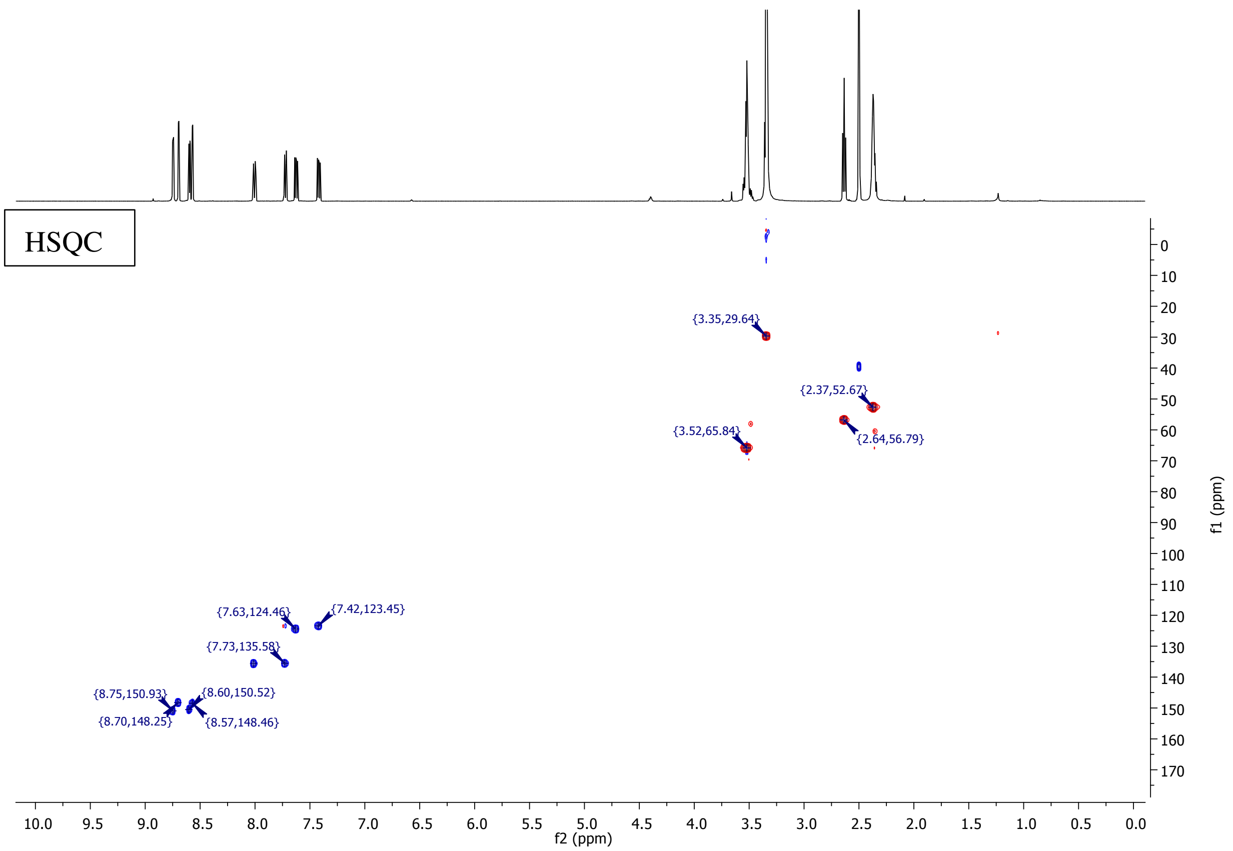
HSQC
{3.35,29.64}
{2.37,52.67}
{3.52,65.84}
{2.64,56.79}
{7.63,124.46}
{7.42,123.45}
{7.73,135.58}
{8.75,150.93}
{8.60,150.52}
{8.70,148.25}
{8.57,148.46}
f2 (ppm)
f1 (ppm)

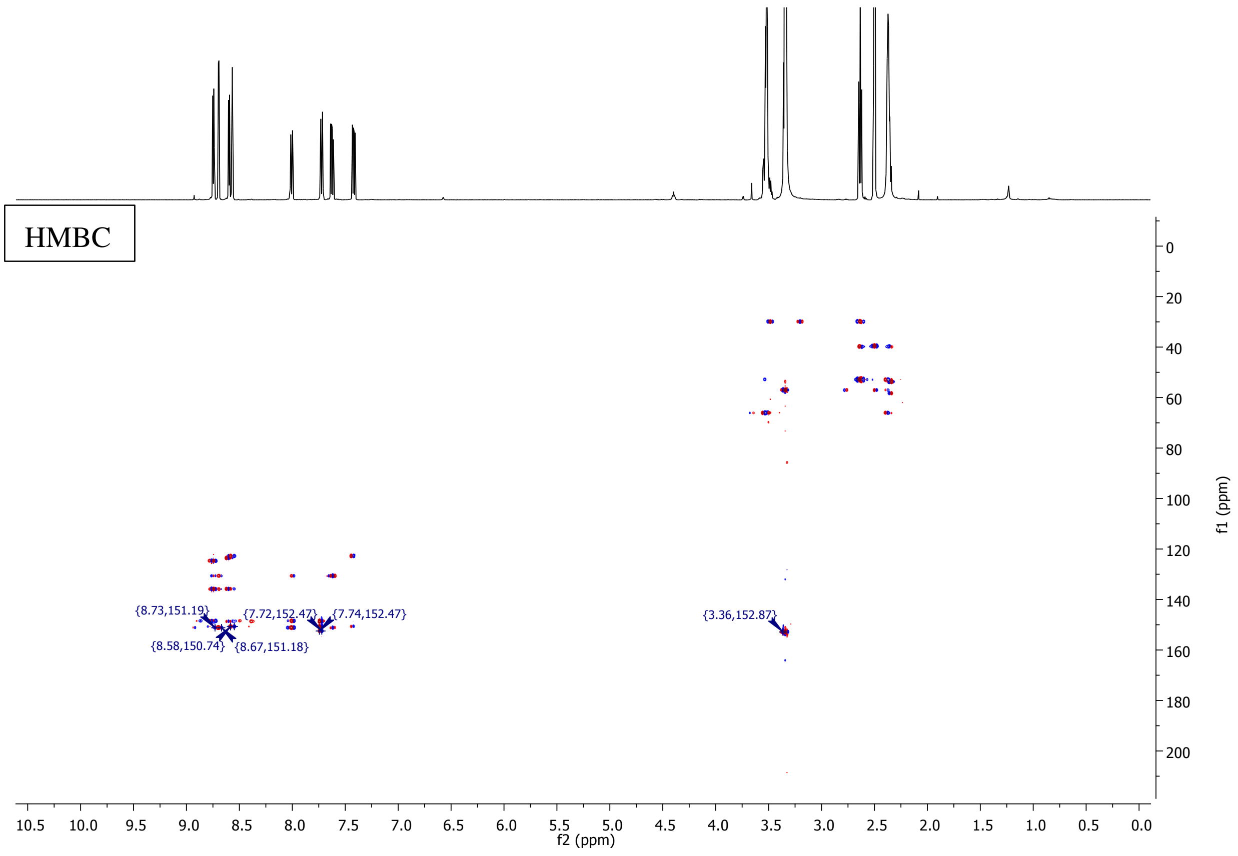
HMBC
{8.73,151.19}
{7.72,152.47}
{7.74,152.47}
{8.58,150.74}
{8.67,151.18}
{3.36,152.87}
f2 (ppm)
f1 (ppm)

UPLC-MS (MeOH): m/z 369.38 $[M+H]^+$. Rt. = 0.56 min. Analysis type: LCMS basic method. Purity 98 %.

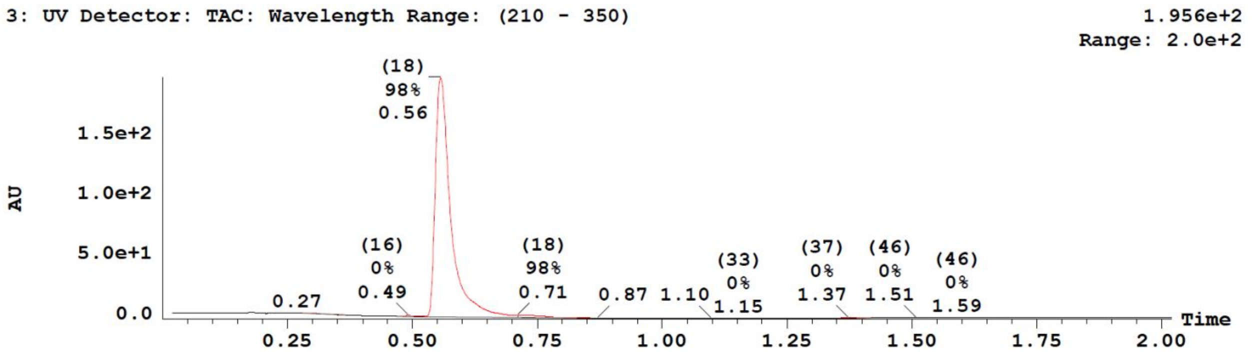

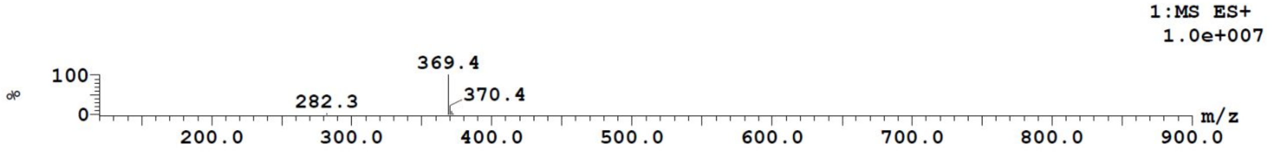

HRMS (ESI-TOF, Exploris 240): experimental m/z 369.1494 $[M+H]^+$, theoretical m/z 369.1492 $[M+H]^+$. Δ = 0.0002.

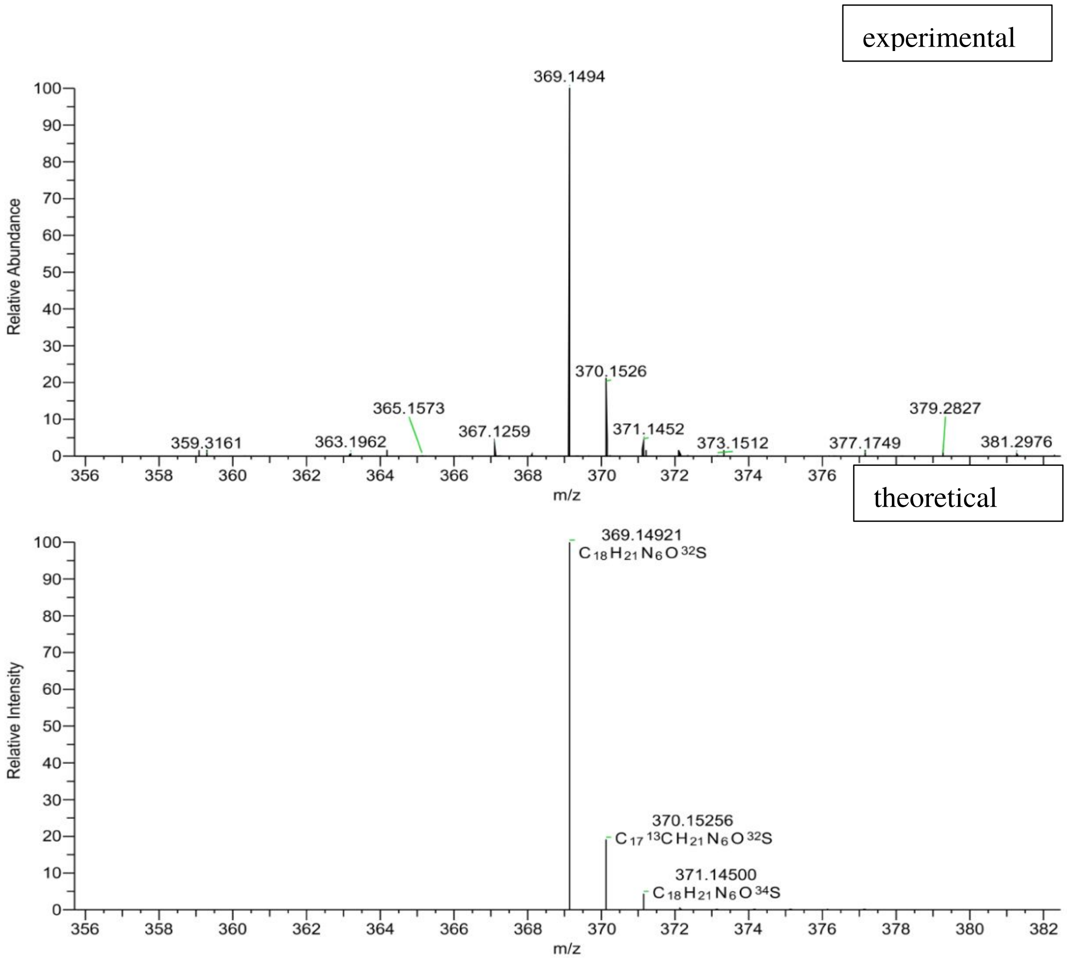

Compound 5

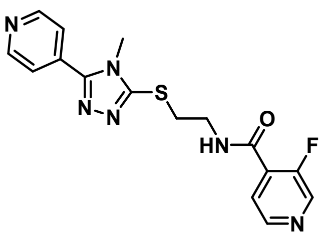

^{1}H-NMR (Bruker 500 MHz, DMSO-d_6) δ 8.91 (t, J = 5.3 Hz, 1H), 8.77 (dd, J = 4.5, 1.6 Hz, 2H), 8.69 (d, J = 1.0 Hz, 1H), 8.53 (dd, J = 4.8, 1.0 Hz, 1H), 7.74 (dd, J = 4.5, 1.6 Hz, 2H), 7.61 - 7.56 (m, 1H), 3.68 (s, 3H), 3.67 - 3.62 (m, 2H), 3.41 (t, J = 6.6 Hz, 2H).

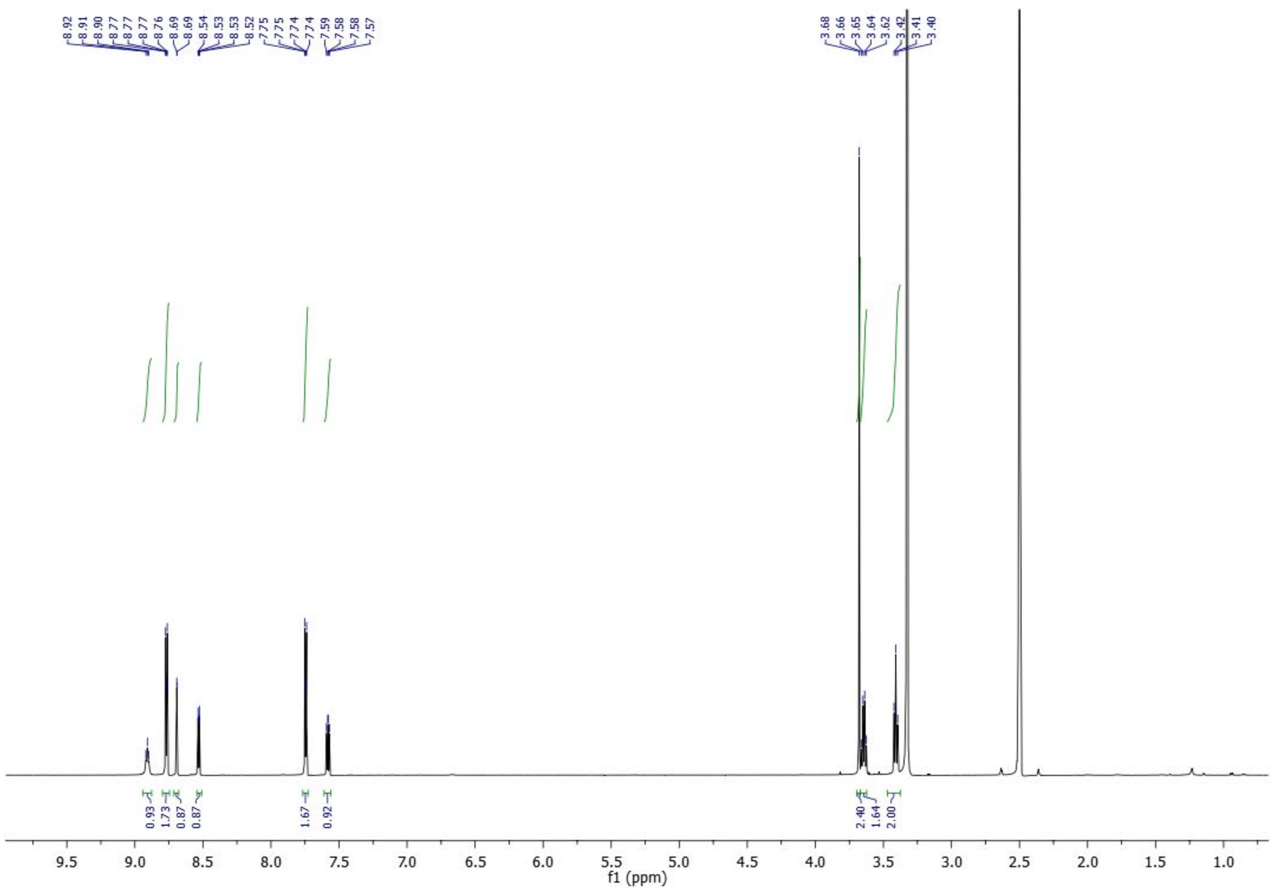

HSQC, HMBC (Bruker 500 MHz, DMSO-d_6) δ 162.20 (C), 153.31 (C), 151.91 (C), 150.20 (2 CH), 146.29 (C), 146.11 (CH), 138.79 (CH), 130.67 (C), 123.11 (CH), 122.21 (C), 122.01 (2 CH), 38.78 (CH_2), 31.77 (CH_2), 31.62 (CH_3).

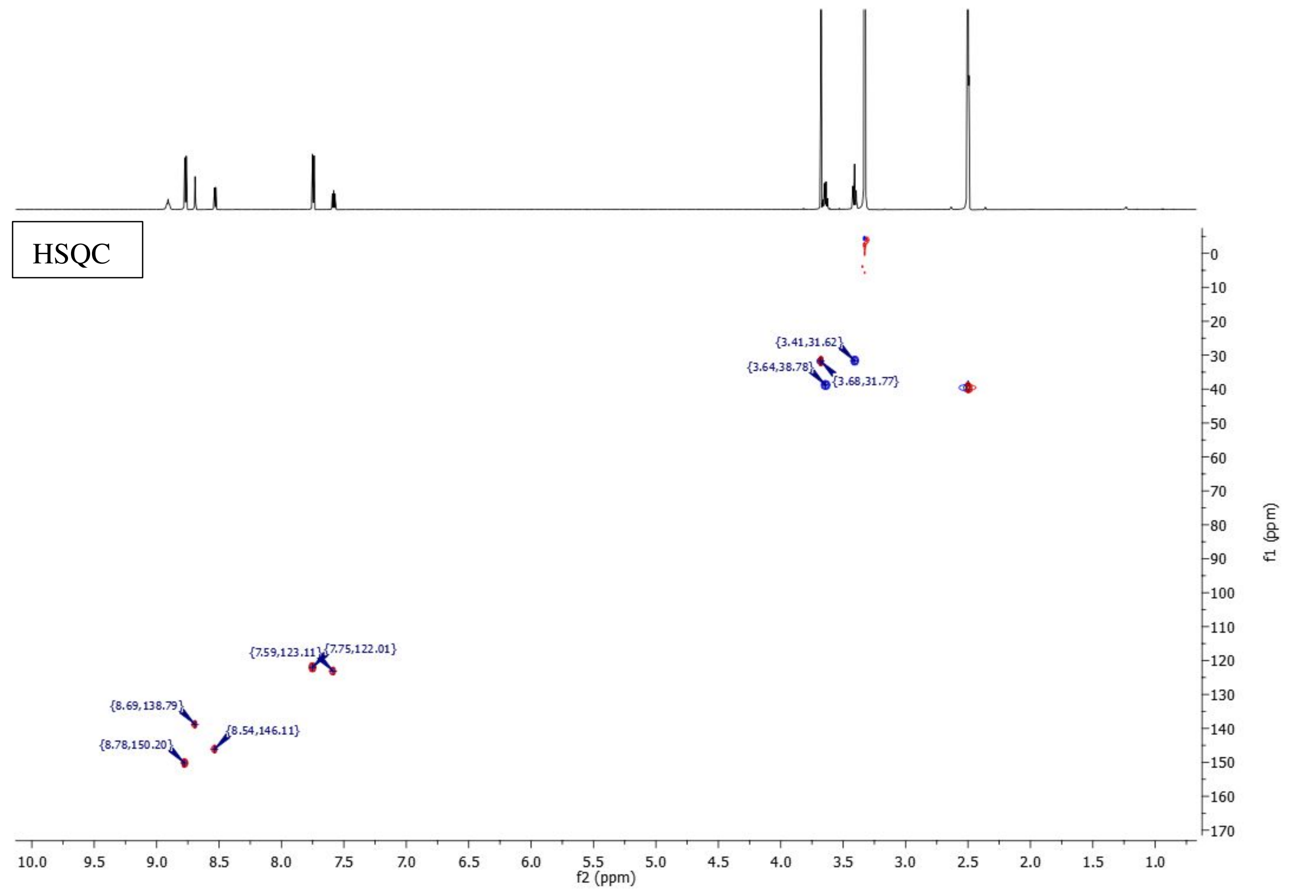
HSQC
{3.41,31.62}
{3.64,38.78}
{3.68,31.77}
{7.59,123.11}
{7.75,122.01}
{8.69,138.79}
{8.54,146.11}
{8.78,150.20}
f1 (ppm)
f2 (ppm)

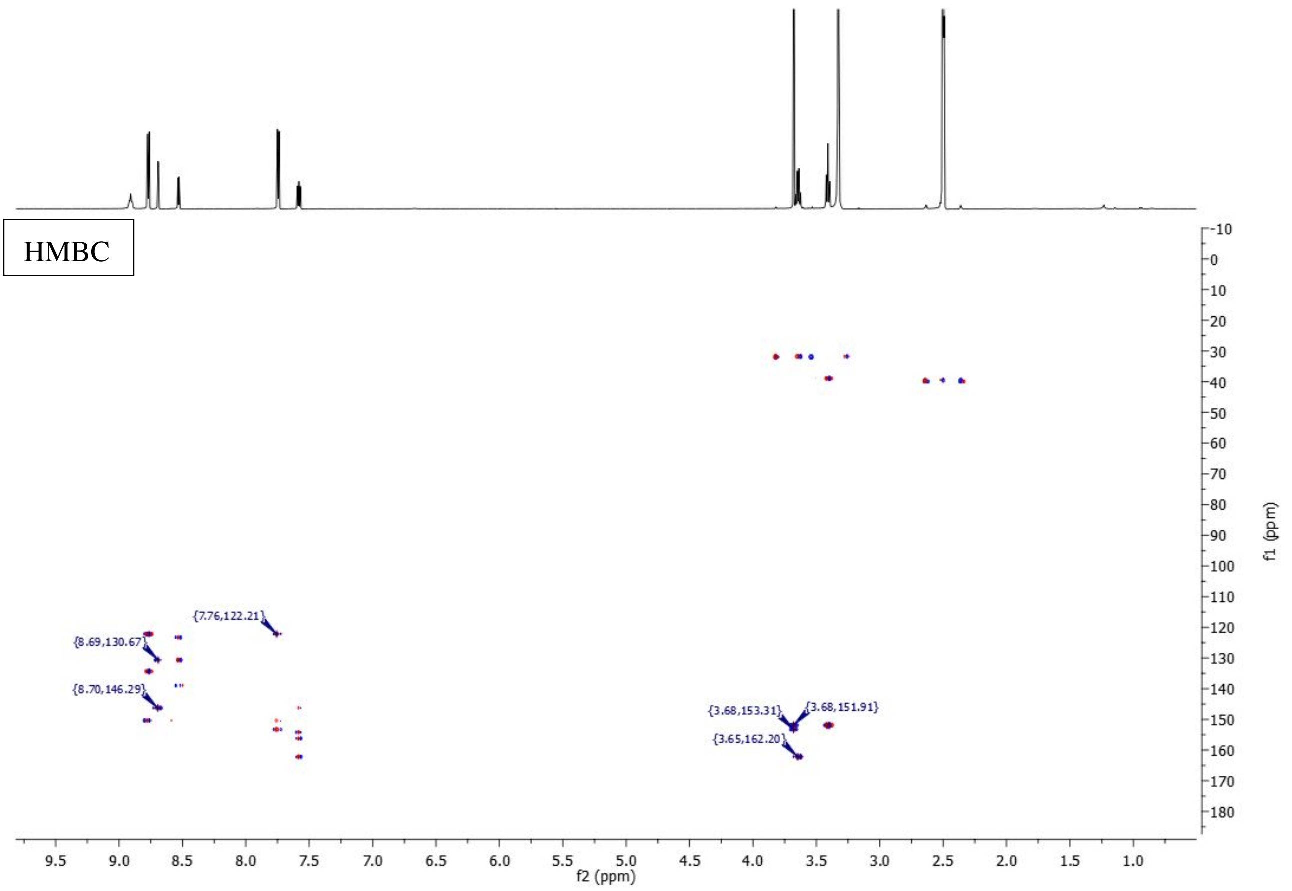
HMBC
{7.76,122.21}
{8.69,130.67}
{8.70,146.29}
{3.68,153.31}
{3.68,151.91}
{3.65,162.20}
f1 (ppm)
f2 (ppm)

UPLC-MS (MeOH): m/z 359.4 [M+H]$^+$. Rt. = 0.49 min. Analysis type: LCMS acid method.

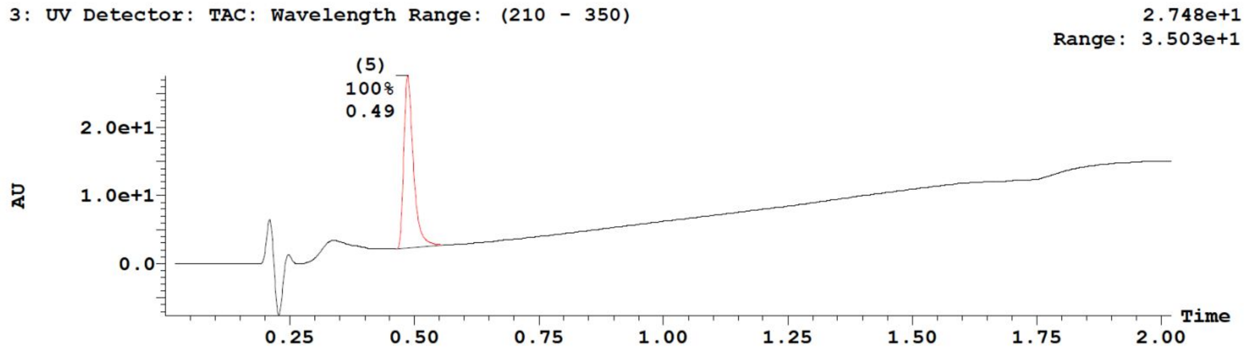

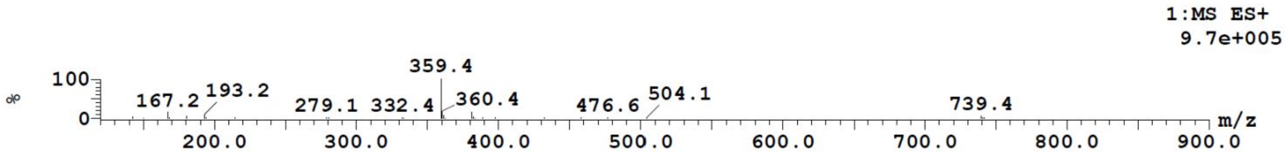

HRMS (ESI-TOF, Exploris 240): experimental m/z 359.1084 [M+H]$^+$, theoretical m/z 359.1085 [M+H]$^+$. Δ = 0.0001.

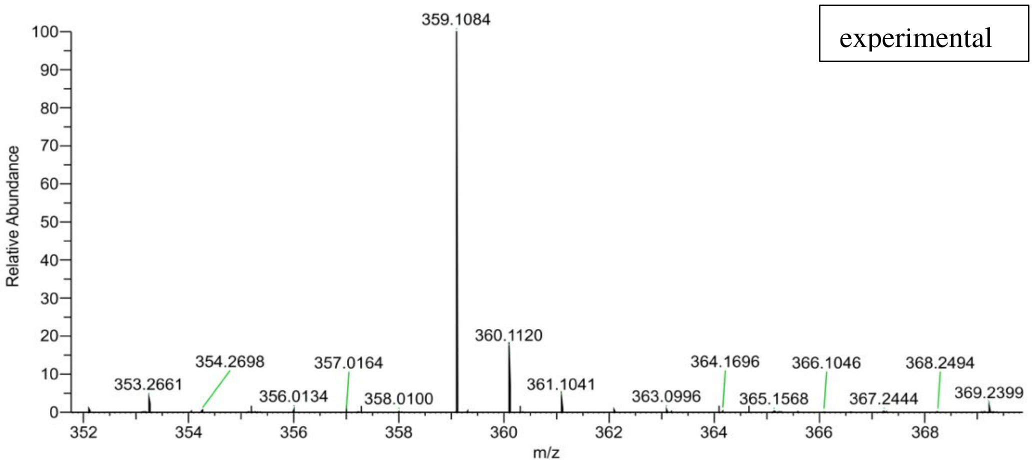

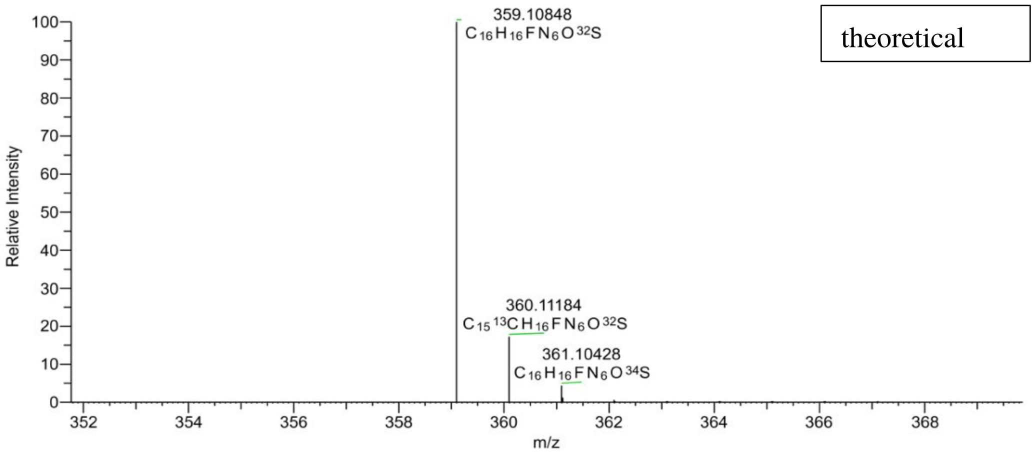

<u>**Compound 6**</u>

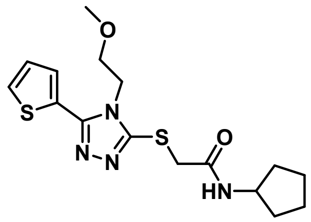

^{1}H NMR (Bruker 500 MHz, DMSO-d_6) δ 8.17 (d, J = 7.1 Hz, 1H), 7.79 (dd, J = 5.1, 1.1 Hz, 1H), 7.64 (dd, J = 3.7, 1.1 Hz, 1H), 7.24 (dd, J = 5.1, 3.7 Hz, 1H), 4.31 (t, J = 5.3 Hz, 2H), 3.95 (dd, J = 13.3, 7.1 Hz, 1H), 3.85 (s, 2H), 3.61 (t, J = 5.3 Hz, 2H), 3.18 (s, 3H), 1.81 – 1.55 (m, 4H), 1.52 – 1.28 (m, 4H).

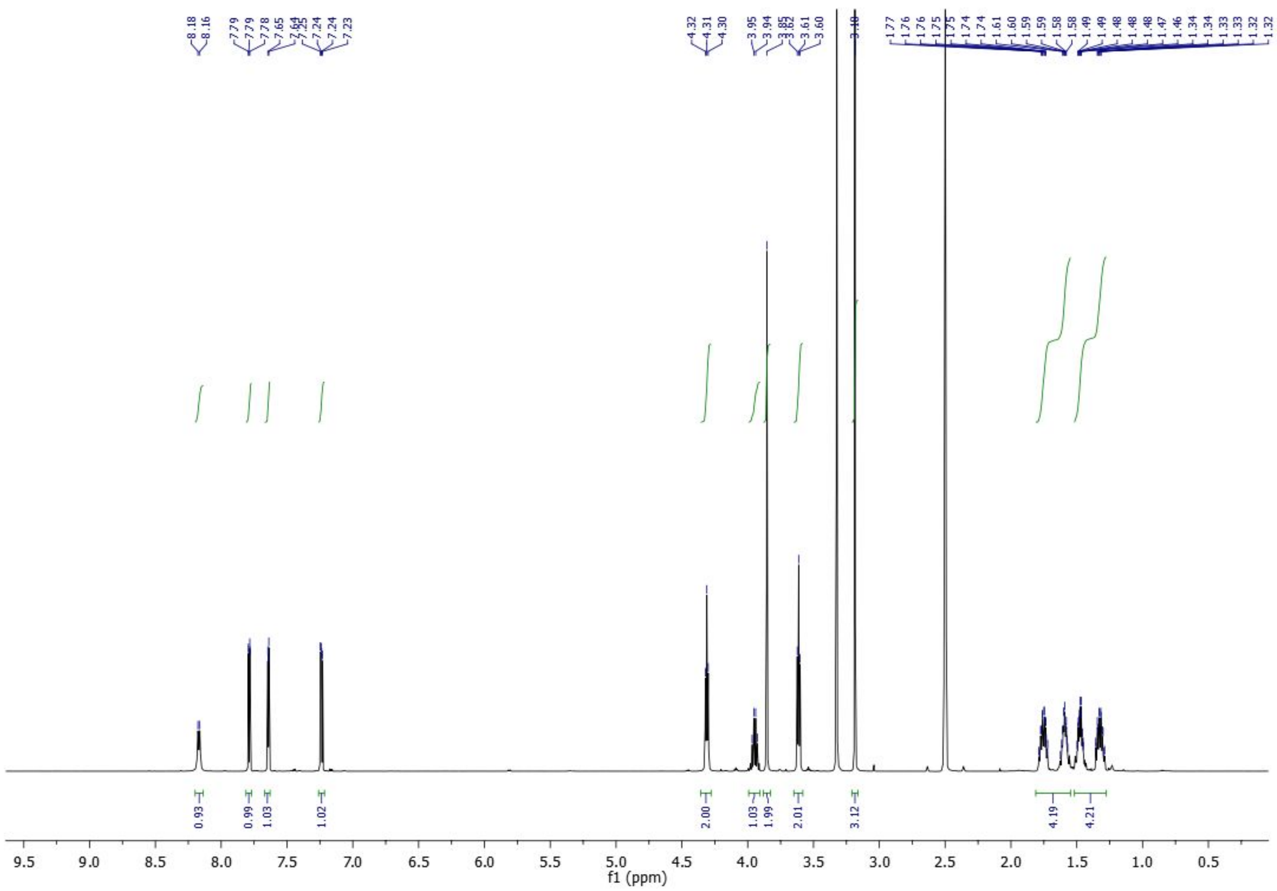

HSQC, HMBC (Bruker 500 MHz, DMSO-d_6) δ 166.14 (C), 151.12 (C), 150.97 (C), 128.80 (CH), 128.05 (C), 127.98 (CH), 127.84 (CH), 69.49 (CH_2), 58.16 (CH_3), 50.44 (CH), 44.23 (CH_2), 36.99 (CH_2), 31.88 (2 CH_2), 23.07 (2 CH_2).

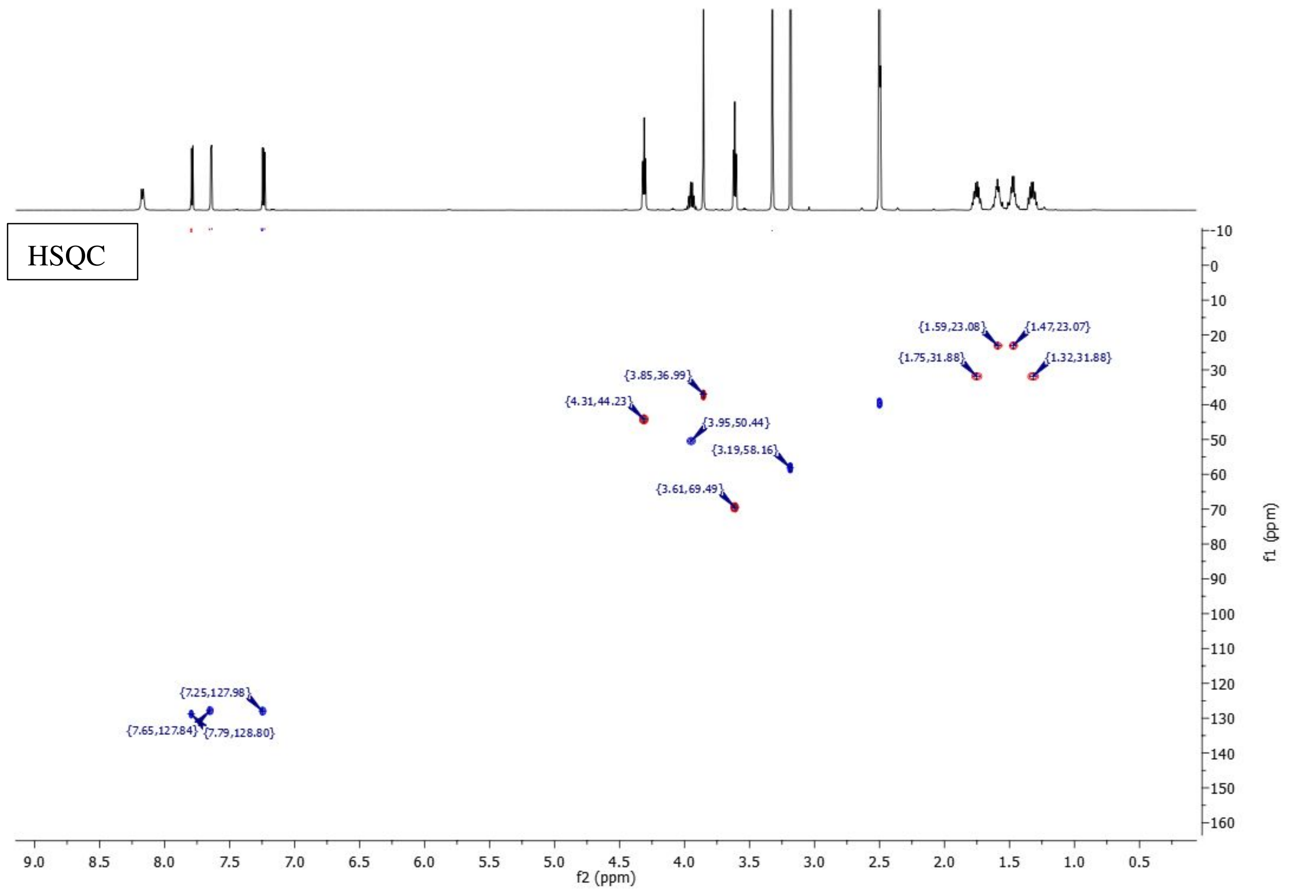
HSQC
{1.59,23.08}
{1.47,23.07}
{1.75,31.88}
{1.32,31.88}
{3.85,36.99}
{4.31,44.23}
{3.95,50.44}
{3.19,58.16}
{3.61,69.49}
{7.25,127.98}
{7.65,127.84}
{7.79,128.80}
f1 (ppm)
f2 (ppm)

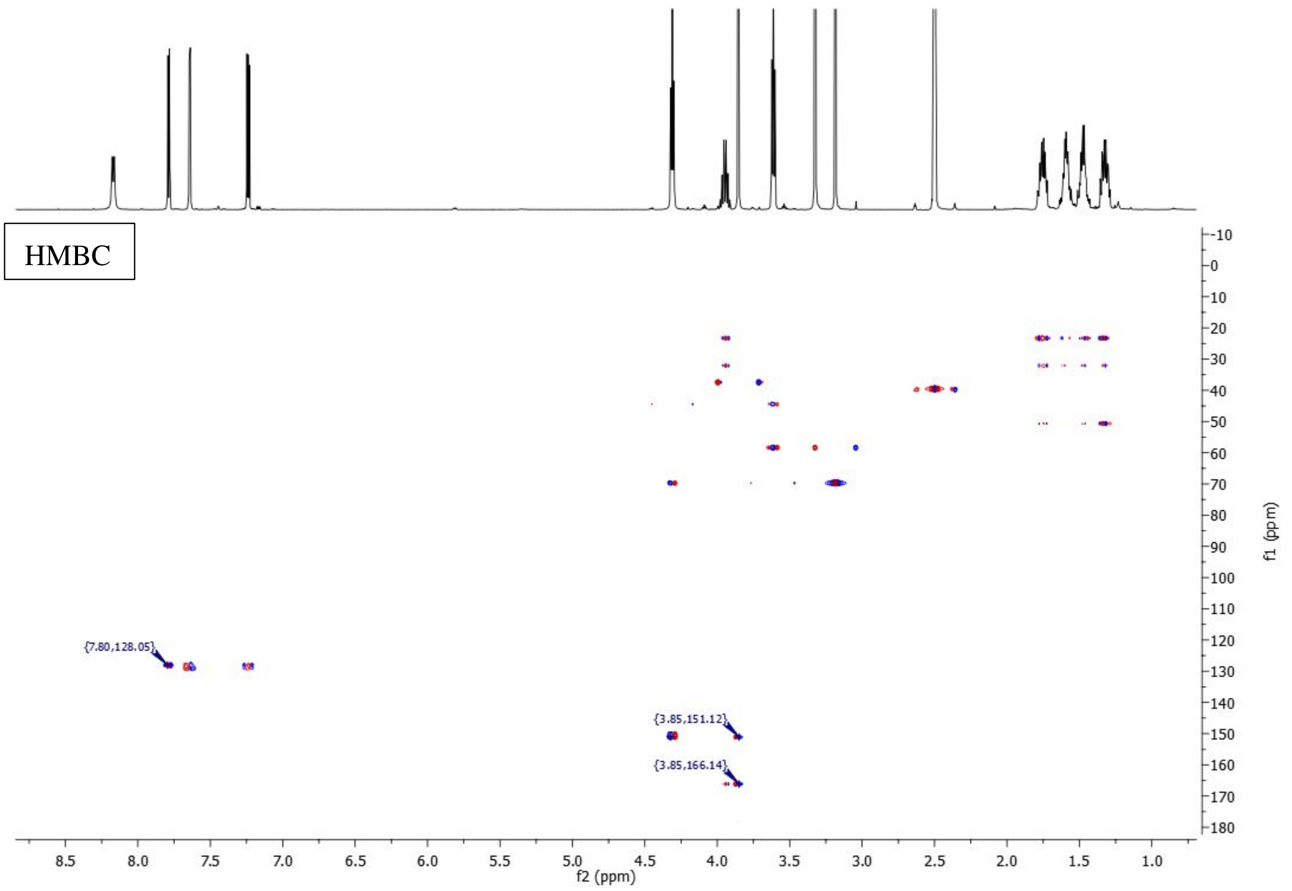
HMBC
{7.80,128.05}
{3.85,151.12}
{3.85,166.14}
f1 (ppm)
f2 (ppm)

UPLC-MS (MeOH): m/z 367.25 $[M+H]^+$. Rt. = 0.82 min. Analysis type: LCMS acid method.

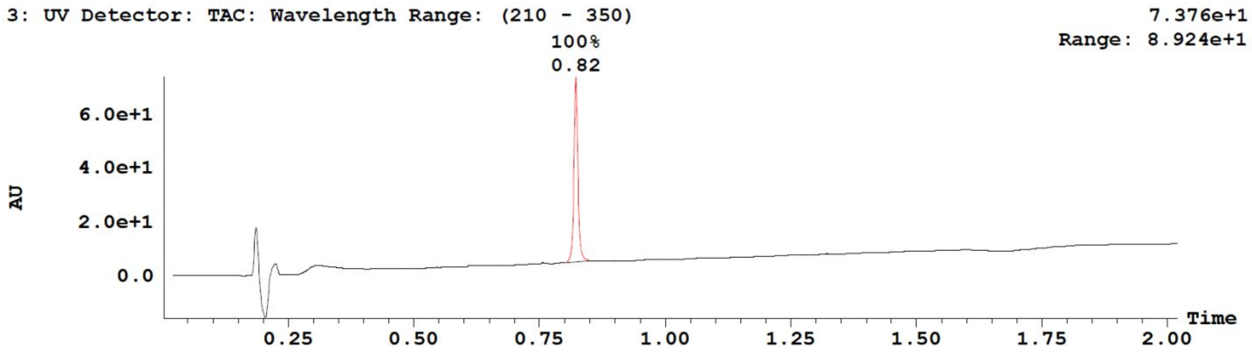

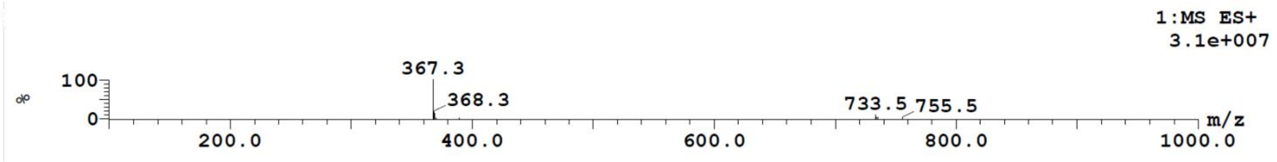

HRMS (ESI-TOF, Exploris 240): experimental m/z 367.1259 [M+H]+, theoretical m/z 367.1257 [M+H]+. Δ = 0.0002.

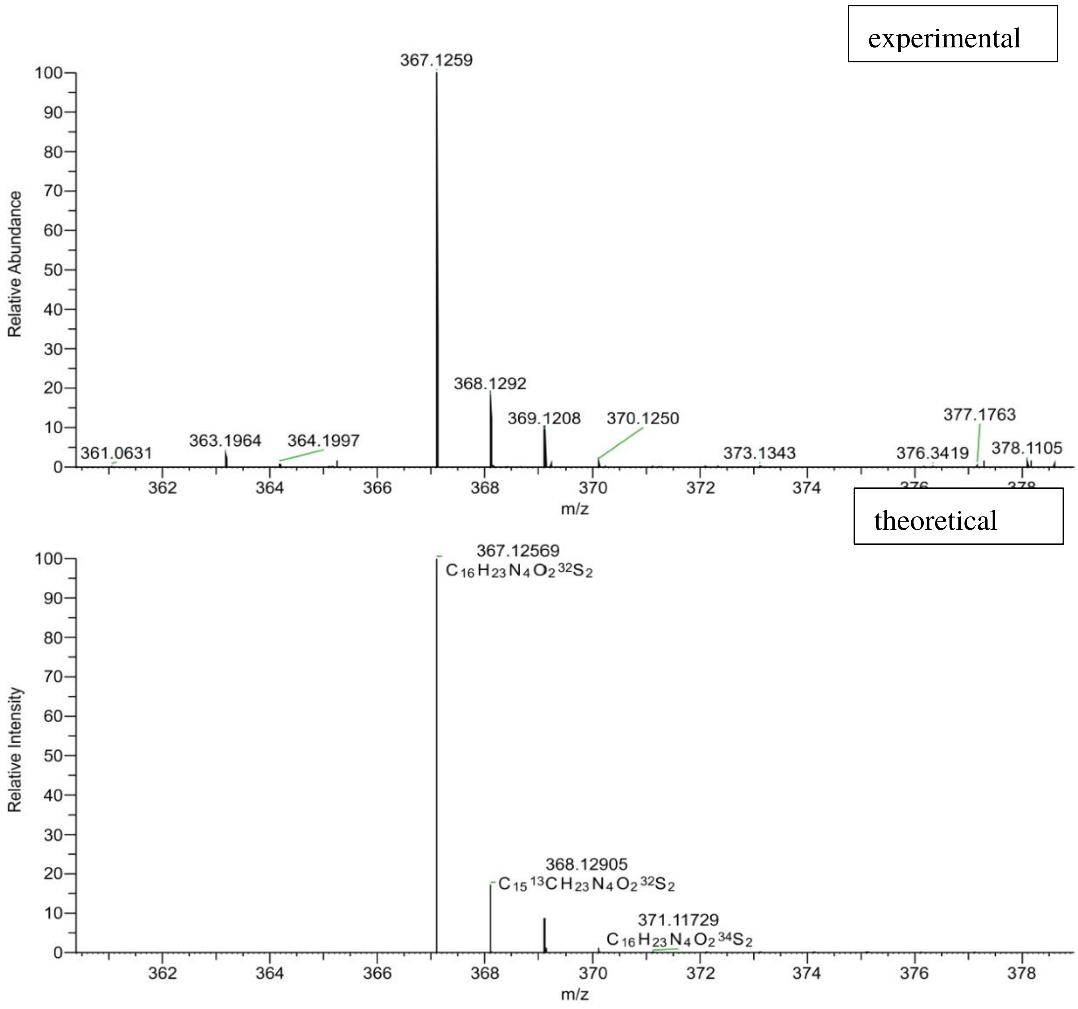

Compound 7

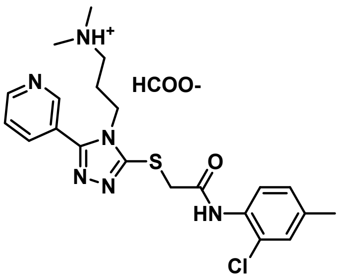

^{1}H NMR (Bruker 500 MHz, DMSO-d_6) δ 9.88 (s, 1H), 8.77 (dd, J = 4.5, 1.6 Hz, 2H), 8.19 (s, 1H), 7.72 (dd, J = 4.5, 1.6 Hz, 2H), 7.61 (d, J = 8.2 Hz, 1H), 7.32 (d, J = 0.9 Hz, 1H), 7.13 (dd, J = 8.2, 0.9

Hz, 1H), 4.25 (s, 2H), 4.14 (t, $J = 6.5$ Hz, 2H), 2.28 (s, 3H), 2.10 (t, $J = 6.5$ Hz, 2H), 1.98 (s, 6H), 1.76 - 1.64 (m, 2H).

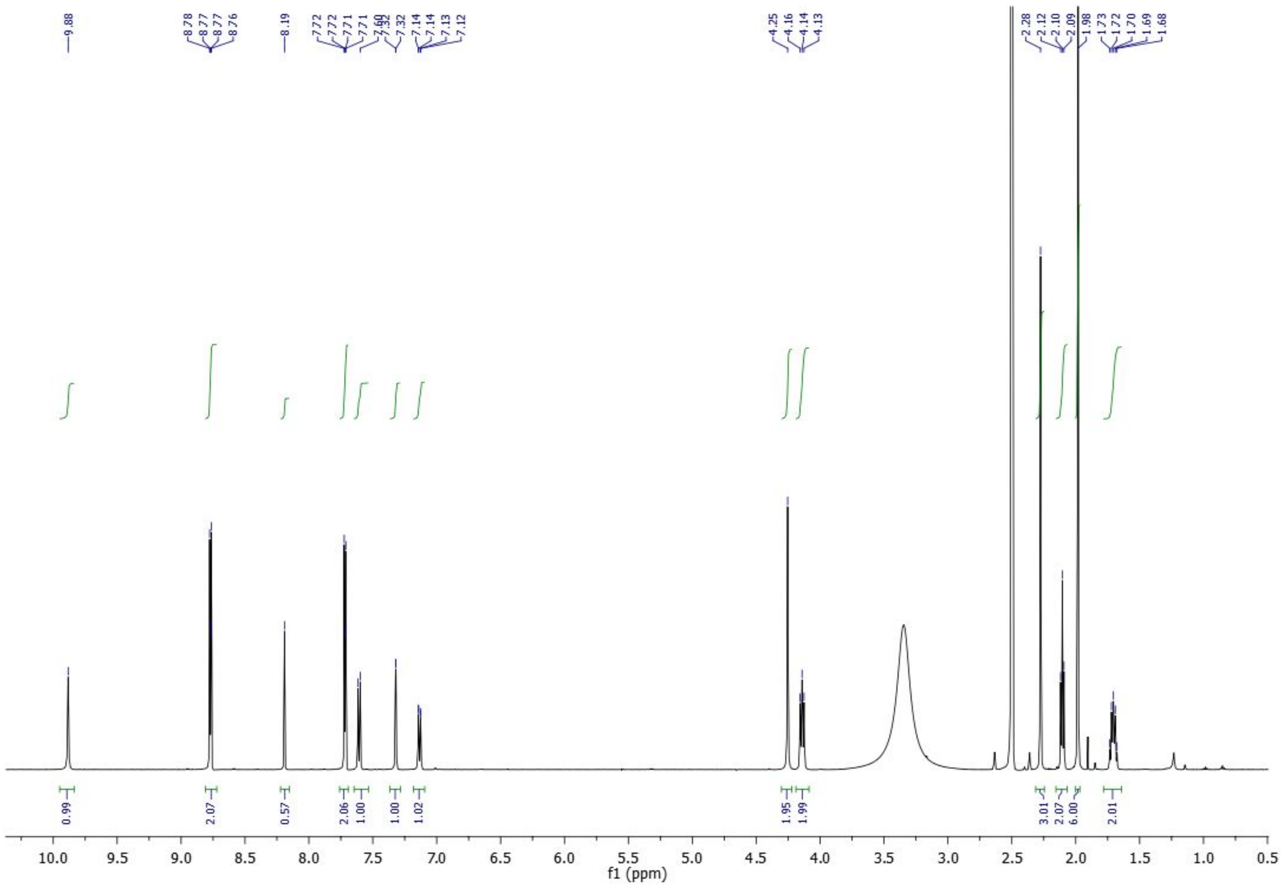

HSQC, HMBC (Bruker 500 MHz, DMSO-d_6) δ 166.25 (C), 163.26 (CH), 153.05 (C), 151.58 (C), 150.29 (2 CH), 136.28 (C), 131.97 (C), 129.42 (CH), 127.85 (CH), 125.94 (C), 125.23 (CH), 122.39 (C), 122.18 (2 CH), 55.12 (CH_2), 44.54 (2 CH_3), 42.66 (CH_2), 36.64 (CH_2), 26.67 (CH_2), 19.89 (CH_3).

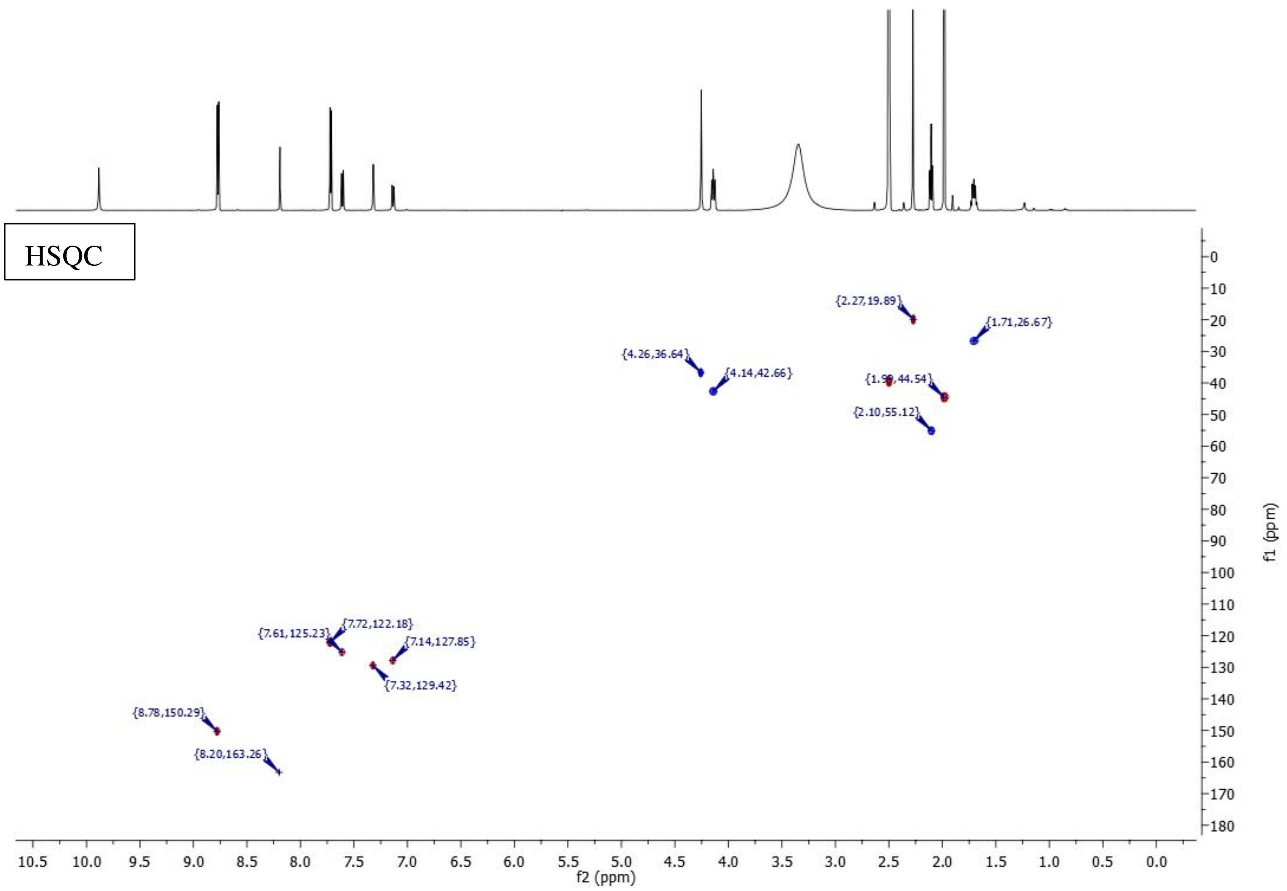
HSQC
{2.27,19.89}
{1.71,26.67}
{4.26,36.64}
{4.14,42.66}
{1.99,44.54}
{2.10,55.12}
{7.72,122.18}
{7.61,125.23}
{7.14,127.85}
{7.32,129.42}
{8.78,150.29}
{8.20,163.26}
f1 (ppm)
f2 (ppm)

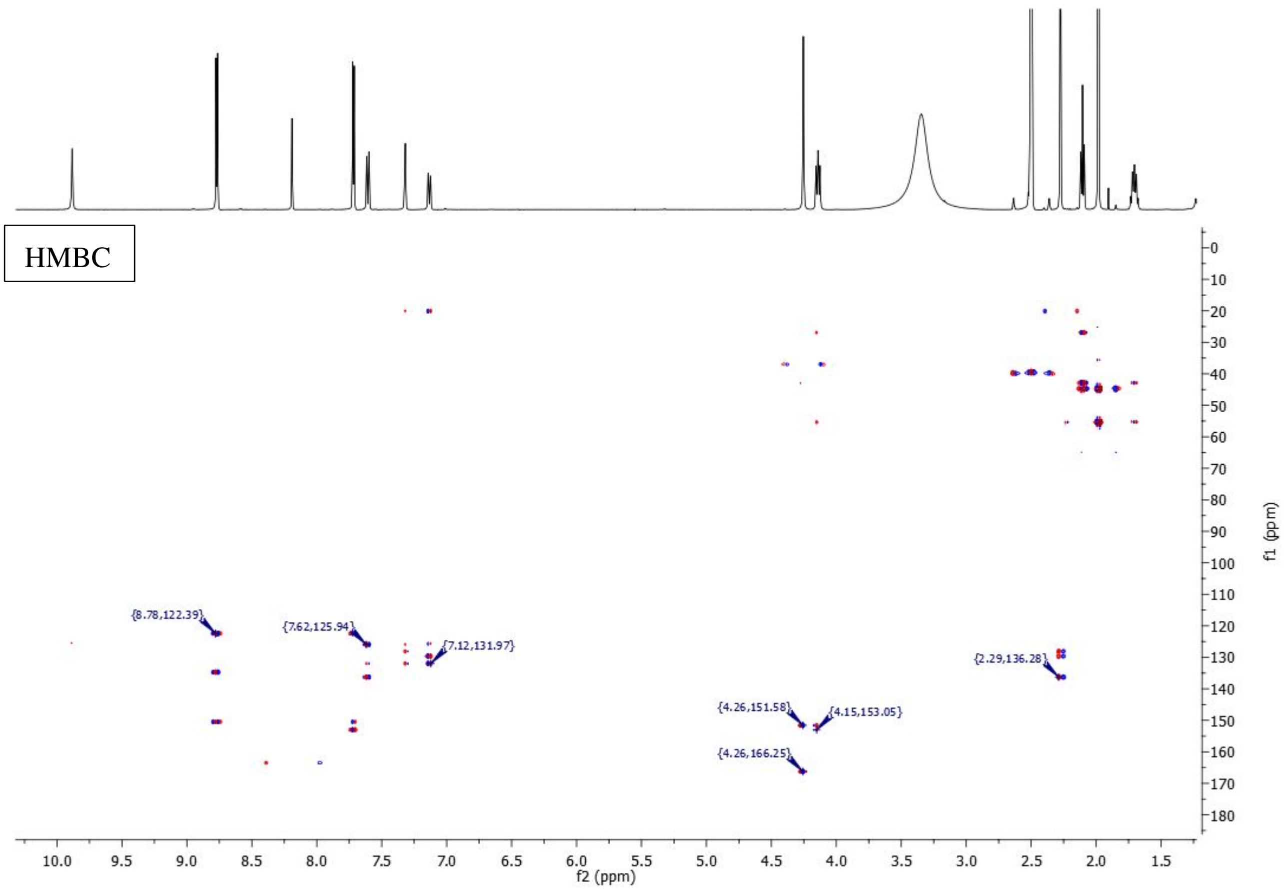
HMBC
{8.78,122.39}
{7.62,125.94}
{7.12,131.97}
{2.29,136.28}
{4.26,151.58}
{4.15,153.05}
{4.26,166.25}
f1 (ppm)
f2 (ppm)

UPLC-MS (MeOH): m/z 445.31 $[M+H]^+$ (not salified). Rt. = 0.52 min. Analysis Type: LCMS acid method.

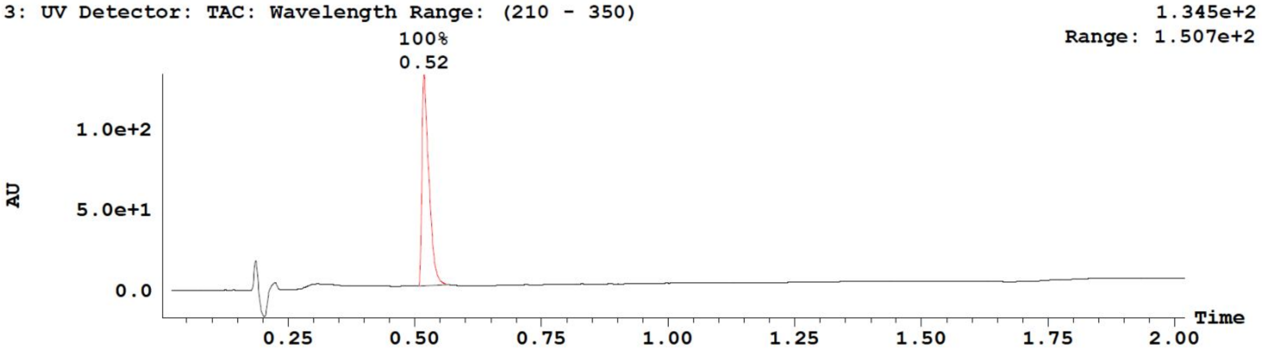

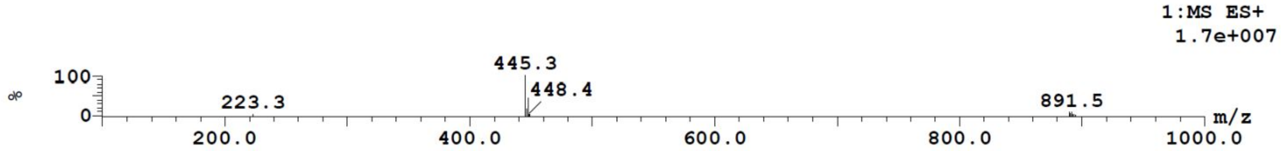

HRMS (ESI-TOF, Exploris 240): experimental m/z 445.1573 $[M+H]^+$, theoretical m/z 445.1572 $[M+H]^+$. Δ = 0.0001.

experimental

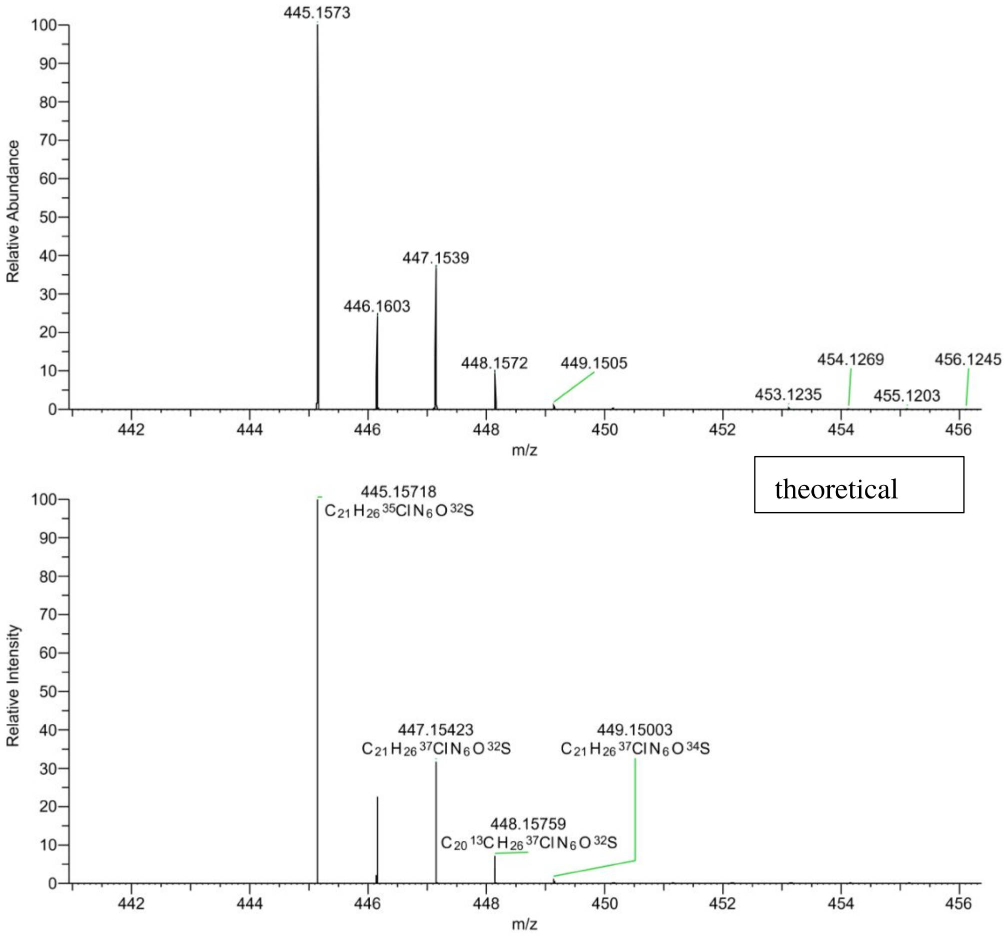

<u>**Compound 8**</u>

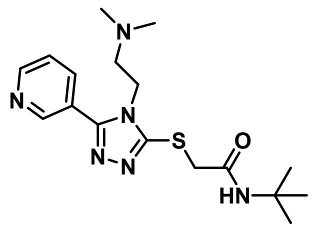

^{1}H NMR (Bruker 500 MHz, DMSO-d_6) δ 8.83 (dd, J = 2.2, 0.8 Hz, 1H), 8.74 (dd, J = 4.9, 1.6 Hz, 1H), 8.14 – 8.05 (m, 1H), 7.90 (s, 1H), 7.60 (ddd, J = 7.9, 4.9, 0.8 Hz, 1H), 4.08 (t, J = 6.4 Hz, 2H), 3.90 (s, 2H), 2.41 (t, J = 6.4 Hz, 2H), 1.97 (s, 6H), 1.24 (s, 9H).

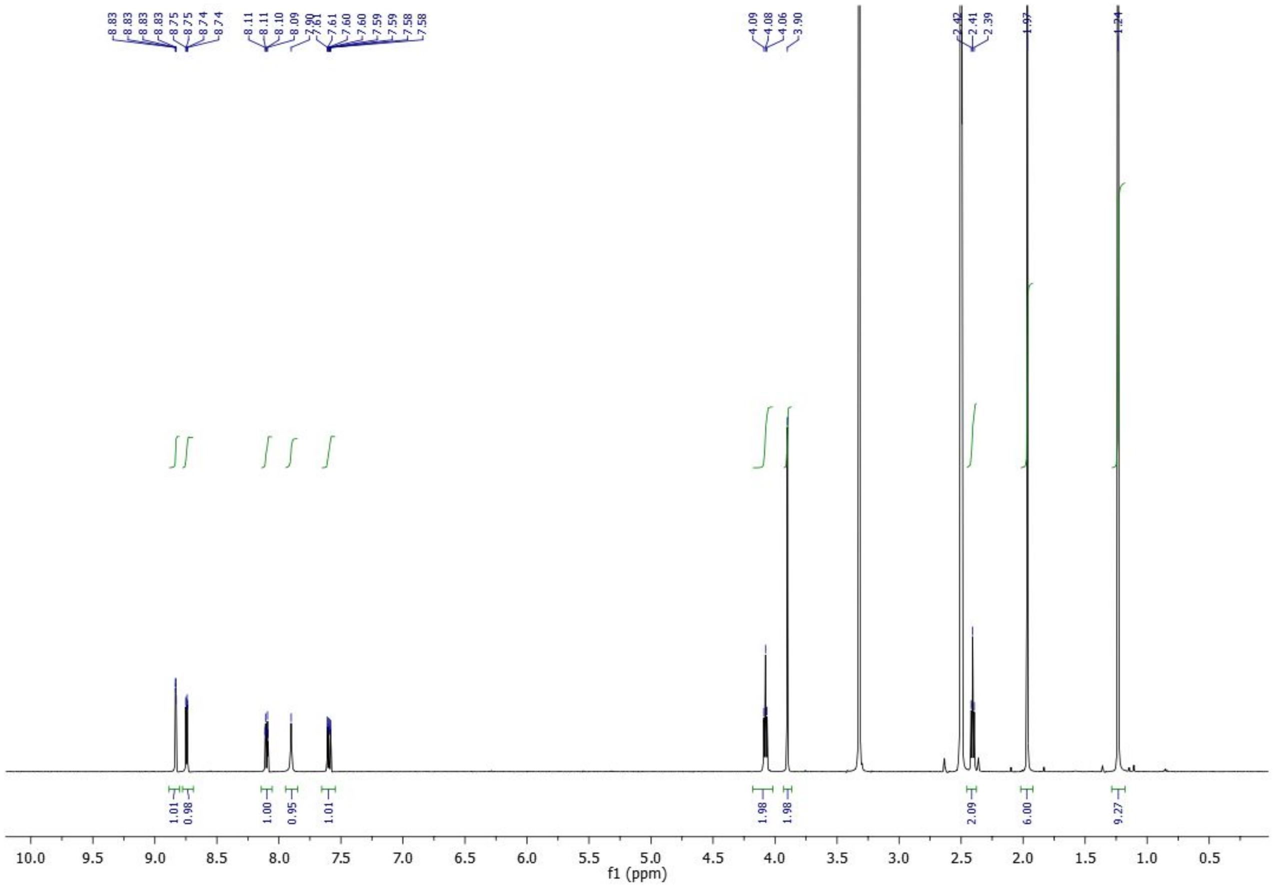

HSQC, HMBC (Bruker 500 MHz, DMSO-d_6) δ 166.13 (C), 153.00 (C), 151.15 (C), 150.86 (C), 150.50 (CH), 148.65 (CH), 135.87 (CH), 123.55 (CH), 57.41 (CH_2), 50.50 (C), 44.79 (2 CH_3), 42.35 (CH_2), 37.78 (CH_2), 28.04 (3 CH_3).

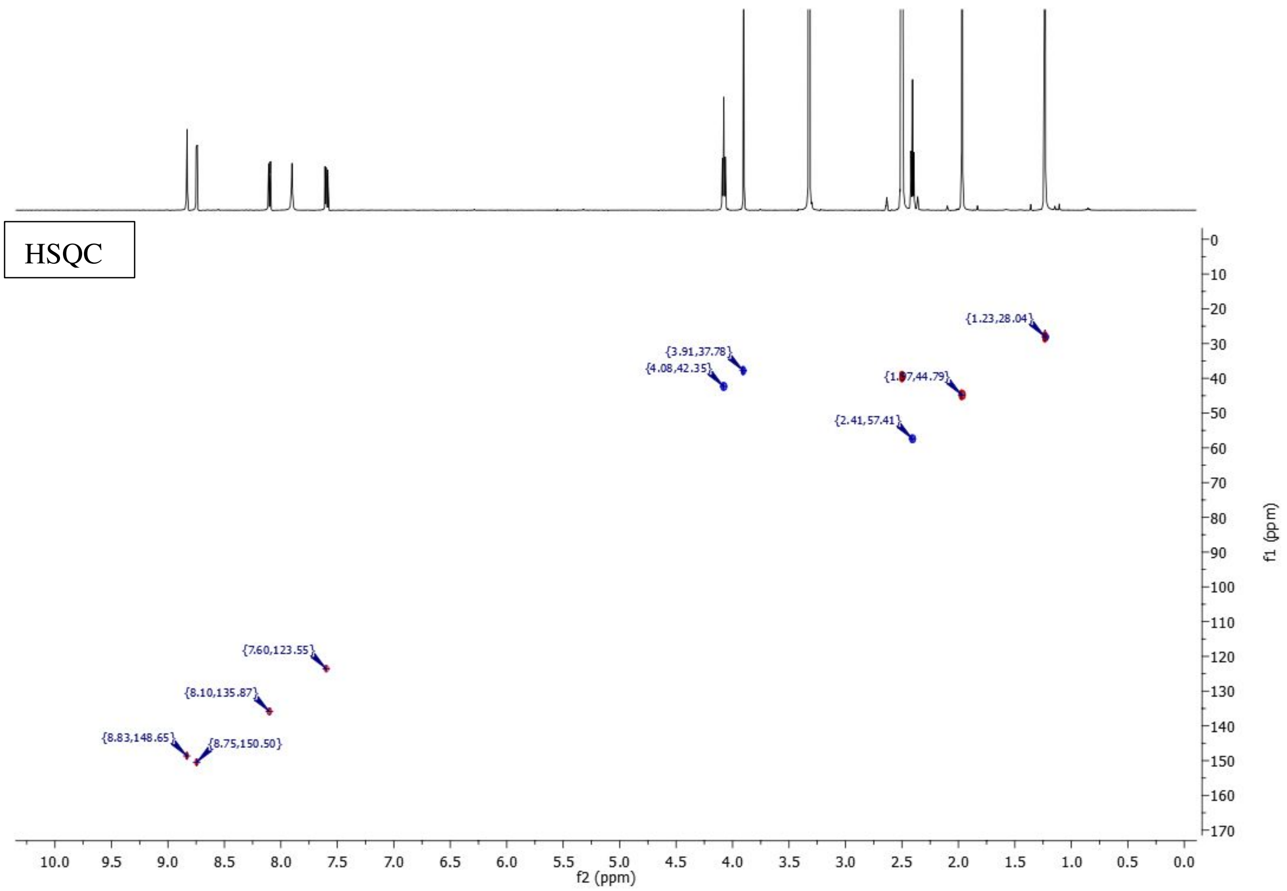
HSQC
{1.23,28.04}
{3.91,37.78}
{4.08,42.35}
{2.41,57.41}
{7.60,123.55}
{8.10,135.87}
{8.83,148.65}
{8.75,150.50}
f1 (ppm)
f2 (ppm)

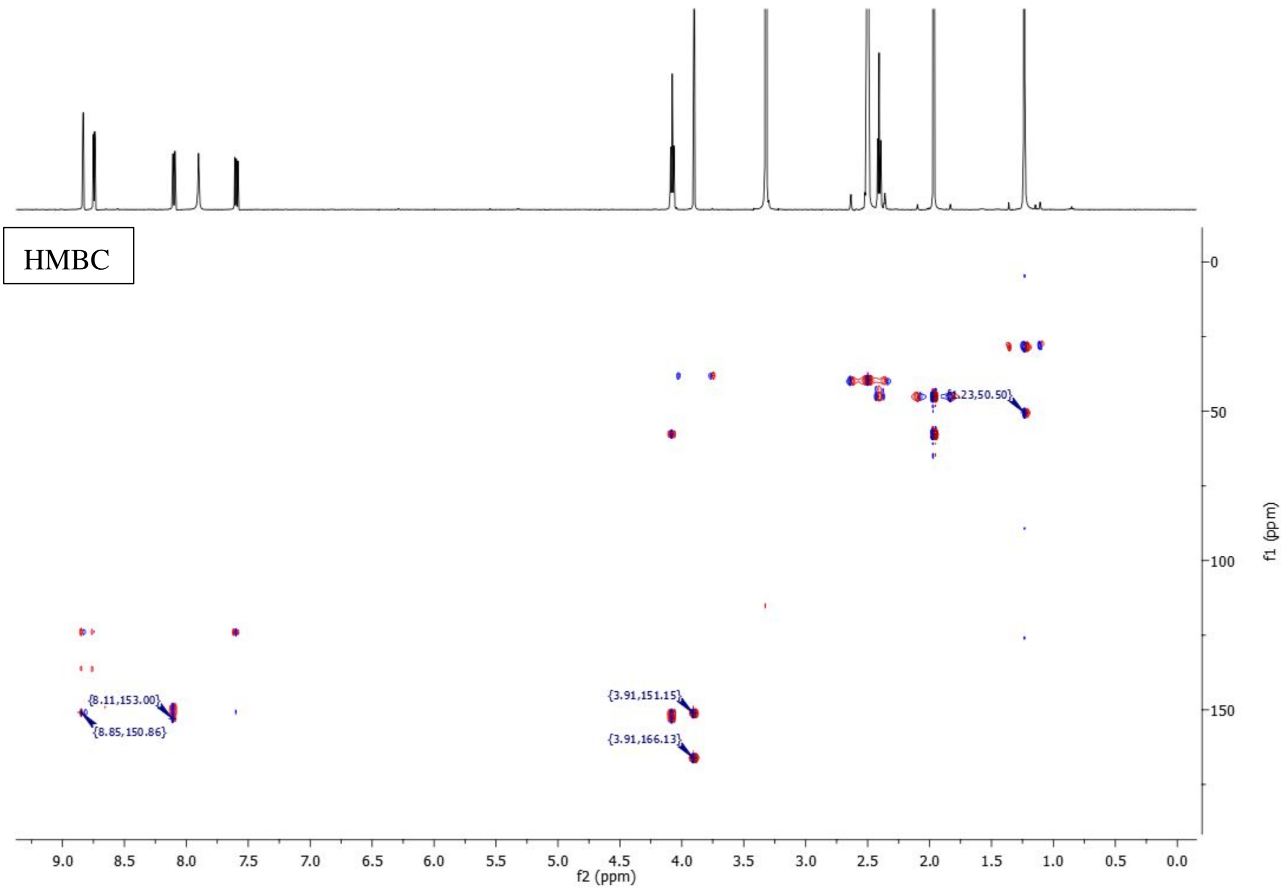
HMBC
{1.23,50.50}
{8.11,153.00}
{8.85,150.86}
{3.91,151.15}
{3.91,166.13}
f1 (ppm)
f2 (ppm)

UPLC-MS (MeOH): m/z 363.5 $[M+H]^+$. Rt. = 0.63 min. Analysis type: LCMS basic method.

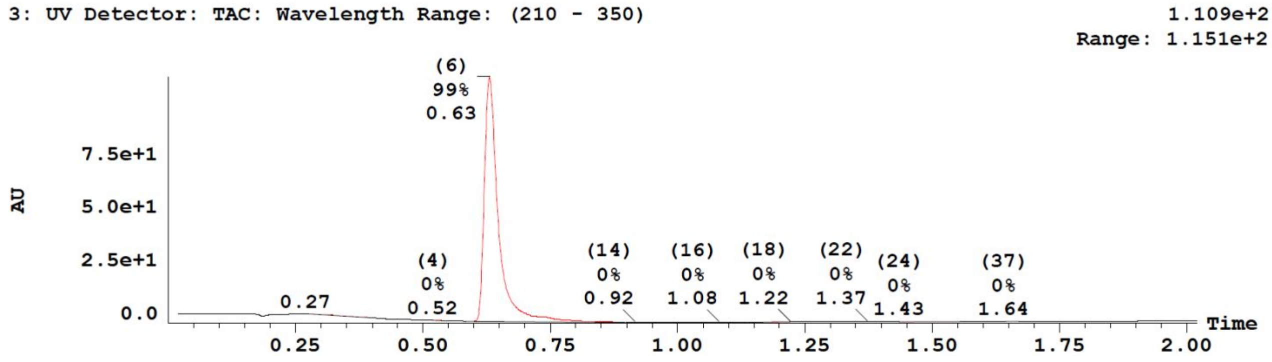

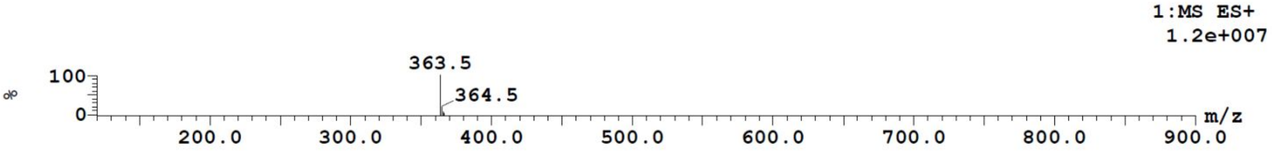

HRMS (ESI-TOF, Exploris 240): experimental m/z 363.1962 $[M+H]^+$, theoretical m/z 363.1962 $[M+H]^+$. Δ =0.0000.

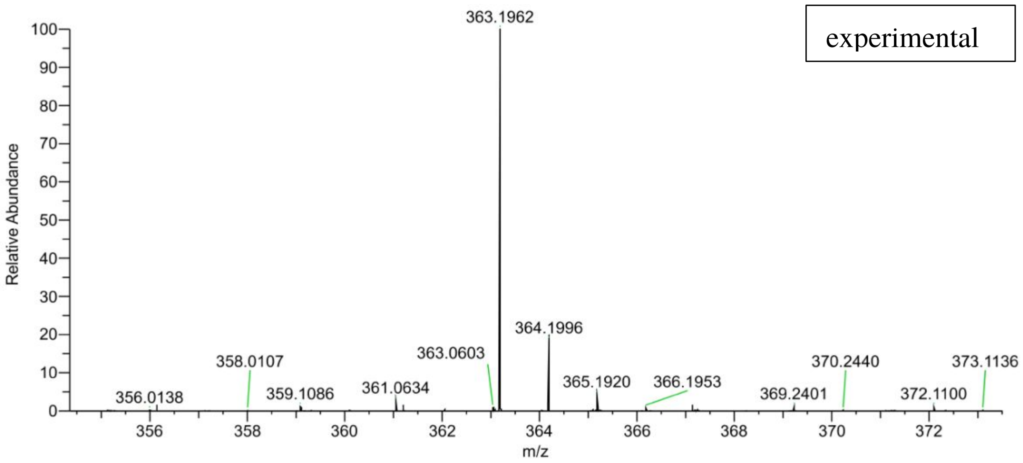

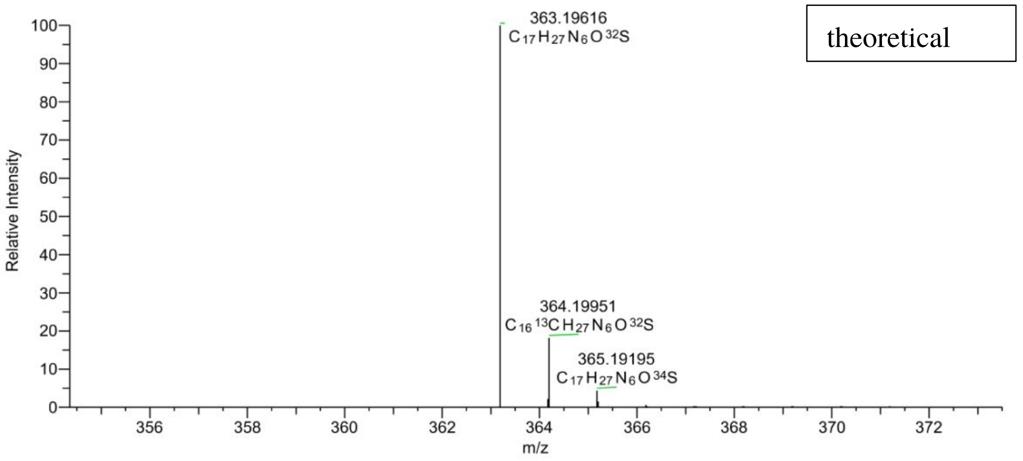

<u>Compound 9</u>

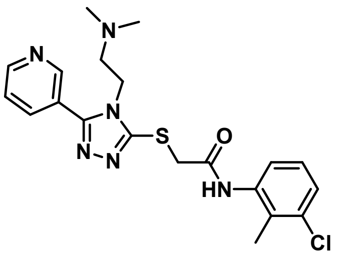

^{1}H NMR (Bruker 400 MHz, DMSO-d_6) δ 10.04 (s, 1H), 8.88 (d, J = 1.8 Hz, 1H), 8.80 (dd, J = 4.8, 1.6 Hz, 1H), 8.14 (dt, J = 7.9, 1.8 Hz, 1H), 7.63 (dd, J = 8.0, 4.8 Hz, 1H), 7.31 (dd, J = 7.9, 1.6 Hz, 2H), 7.20 (t, J = 8.0 Hz, 1H), 4.50 – 4.38 (m, 2H), 4.30 (s, 2H), 3.38 – 3.30 (m, 2H), 2.78 (s, 6H), 2.20 (s, 3H).

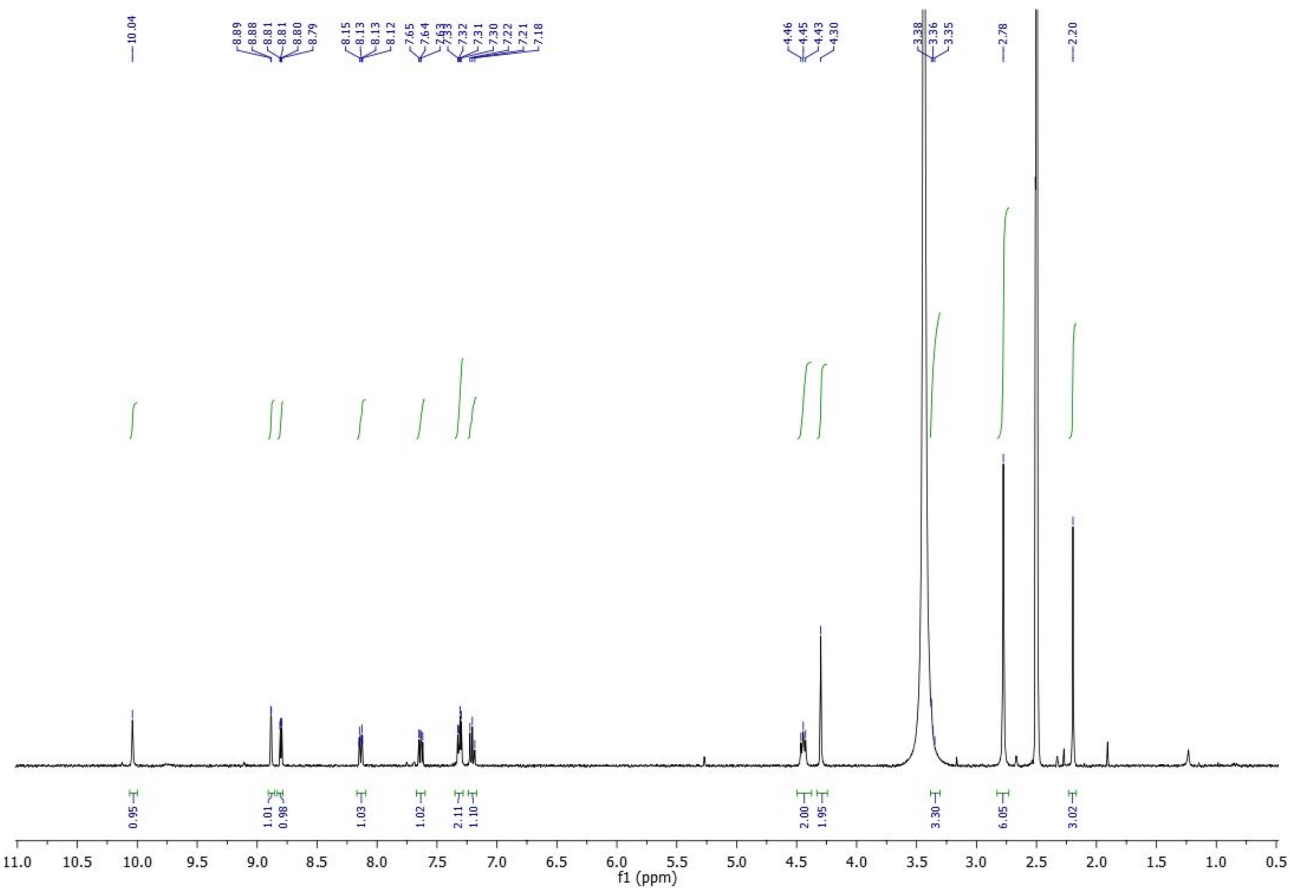

^{13}C NMR (Varian 101 MHz, DMSO-d_6) δ 166.18 (C), 158.83 (C), 158.49 (C), 152.79 (C), 151.02 (CH), 148.79 (CH), 137.45 (C), 136.66 (CH), 133.88 (C), 130.58 (C), 126.95 (CH), 126.42 (CH), 124.41 (CH), 124.17 (CH), 53.99 (CH_2), 42.54 (2 CH_3), 38.89 (CH_2), 37.59 (CH_2), 15.03 (CH_3).

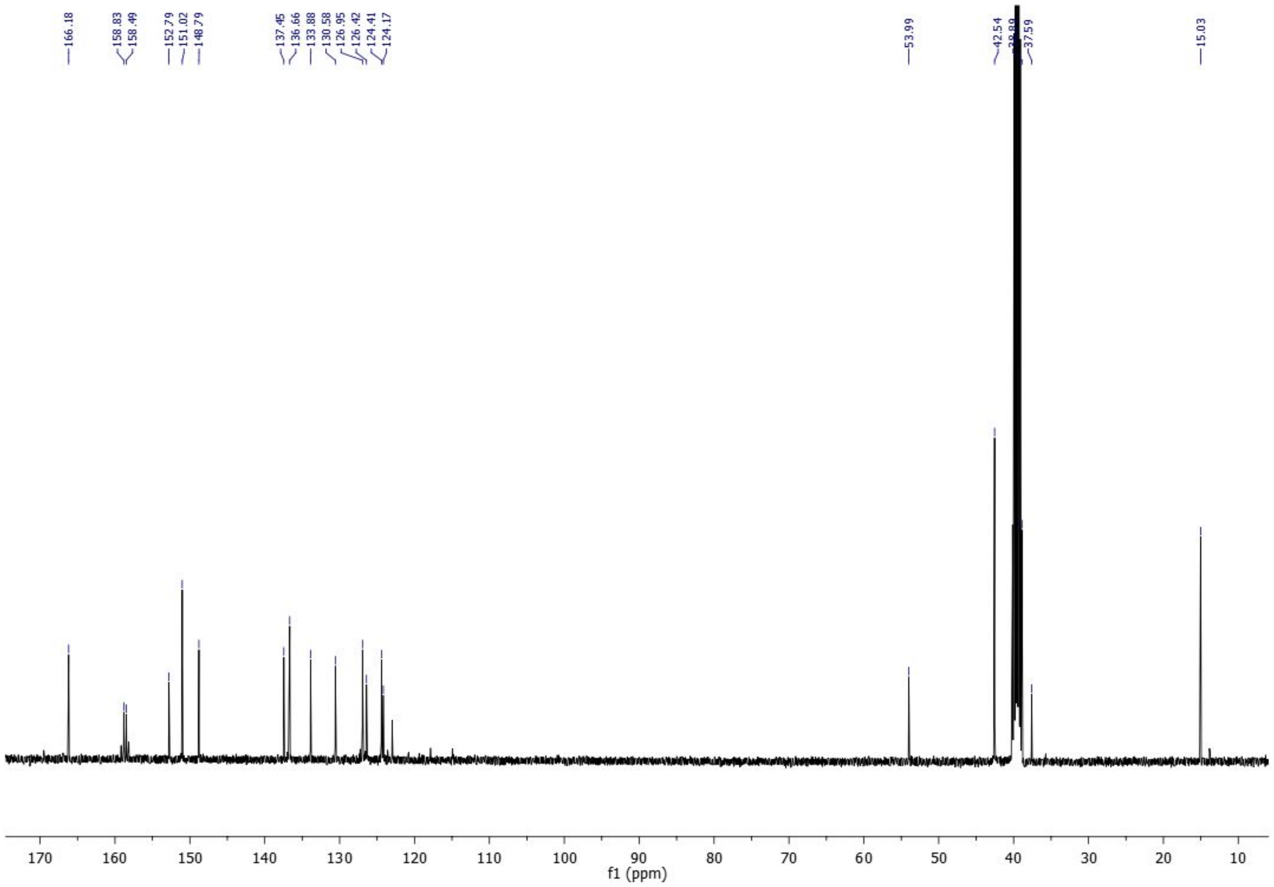

UPLC-MS (MeOH): m/z 431.27 [M+H]$^+$. Rt.= 0.52 min. Analysis type: LCMS acid method.

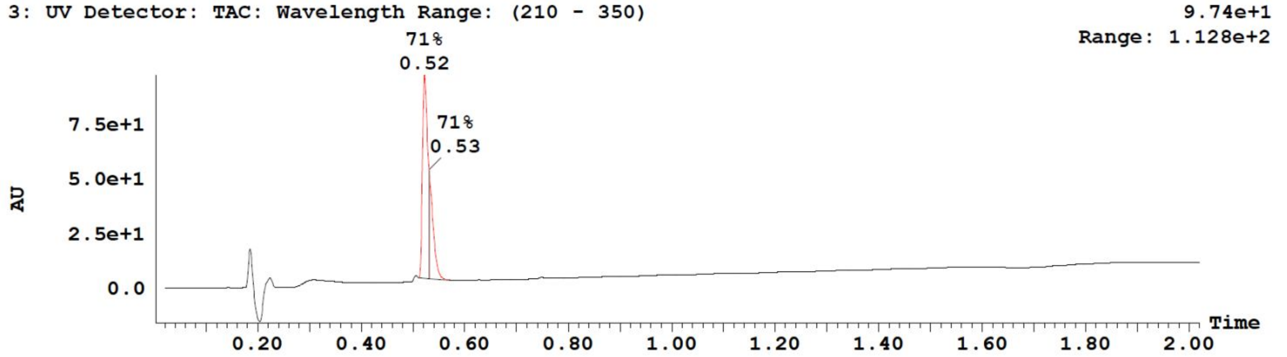

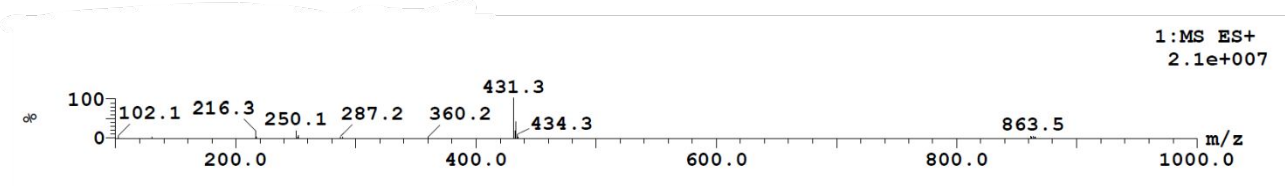

HRMS (ESI-TOF, Exploris 240): experimental m/z 431.1417 $[M+H]^+$, theoretical m/z 431.1415 $[M+H]^+$. $\Delta = 0.0002$.

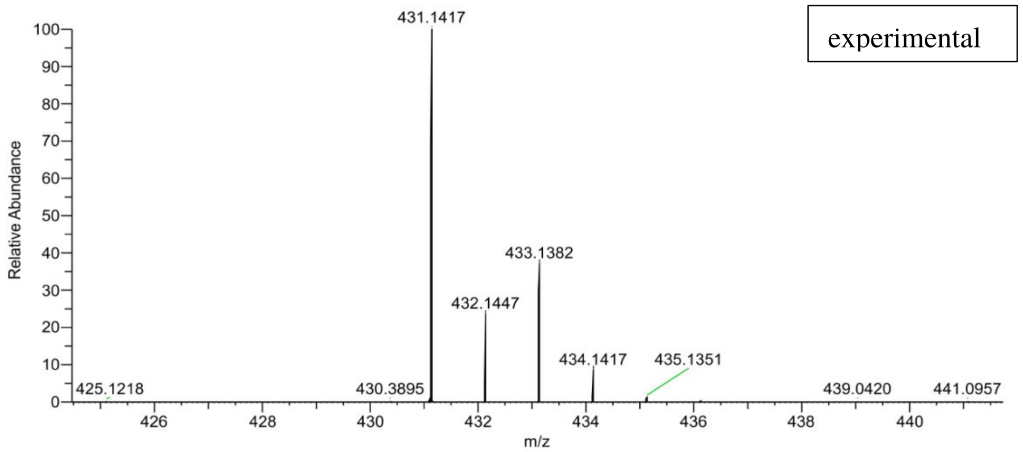

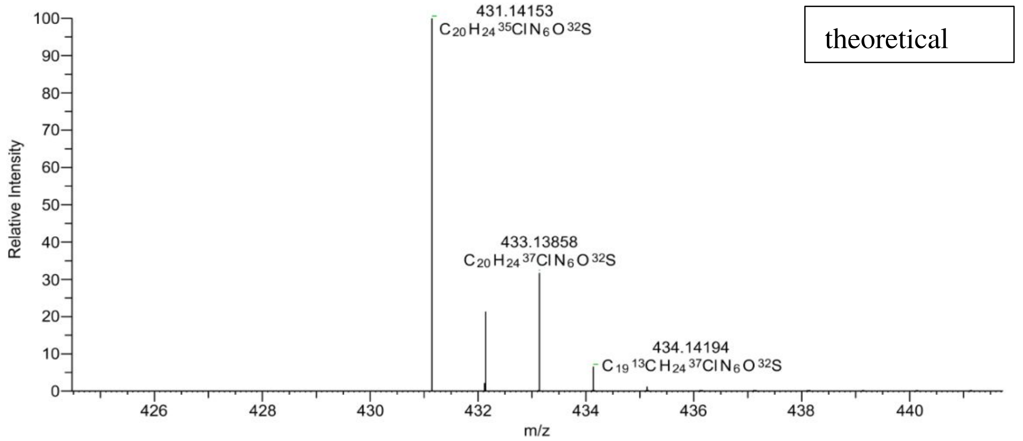

Compound 10

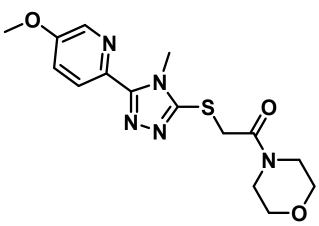

^{1}H NMR (Varian 400 MHz, DMSO-d_6) δ 8.43 (d, J = 3.0 Hz, 1H), 8.06 (d, J = 8.8 Hz, 1H), 7.59 (dd, J = 8.8, 3.0 Hz, 1H), 4.25 (s, 2H), 3.92 (s, 3H), 3.91 (s, 3H), 3.65 – 3.53 (m, 4H), 3.52 – 3.40 (m, 4H).

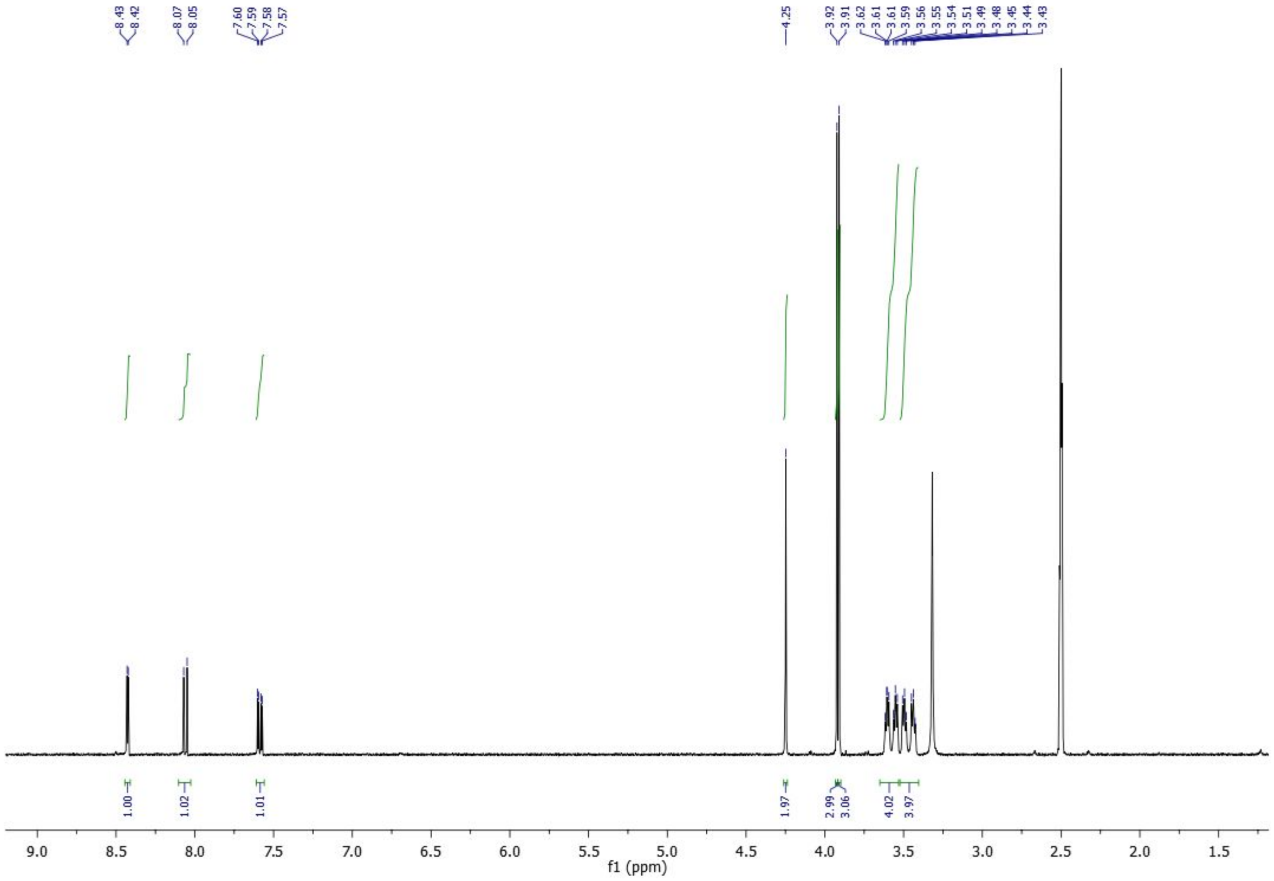

^{13}C NMR (Varian 126 MHz, DMSO-d_6) δ 165.65 (C), 155.87 (C), 152.97 (C), 151.06 (C), 139.65 (C), 136.72 (CH), 124.11 (CH), 121.88 (CH), 65.95 (2 CH_2), 55.90 (CH_3), 45.93 (CH_2), 42.01 (CH_2), 36.43 (CH_2), 32.71 (CH_3).

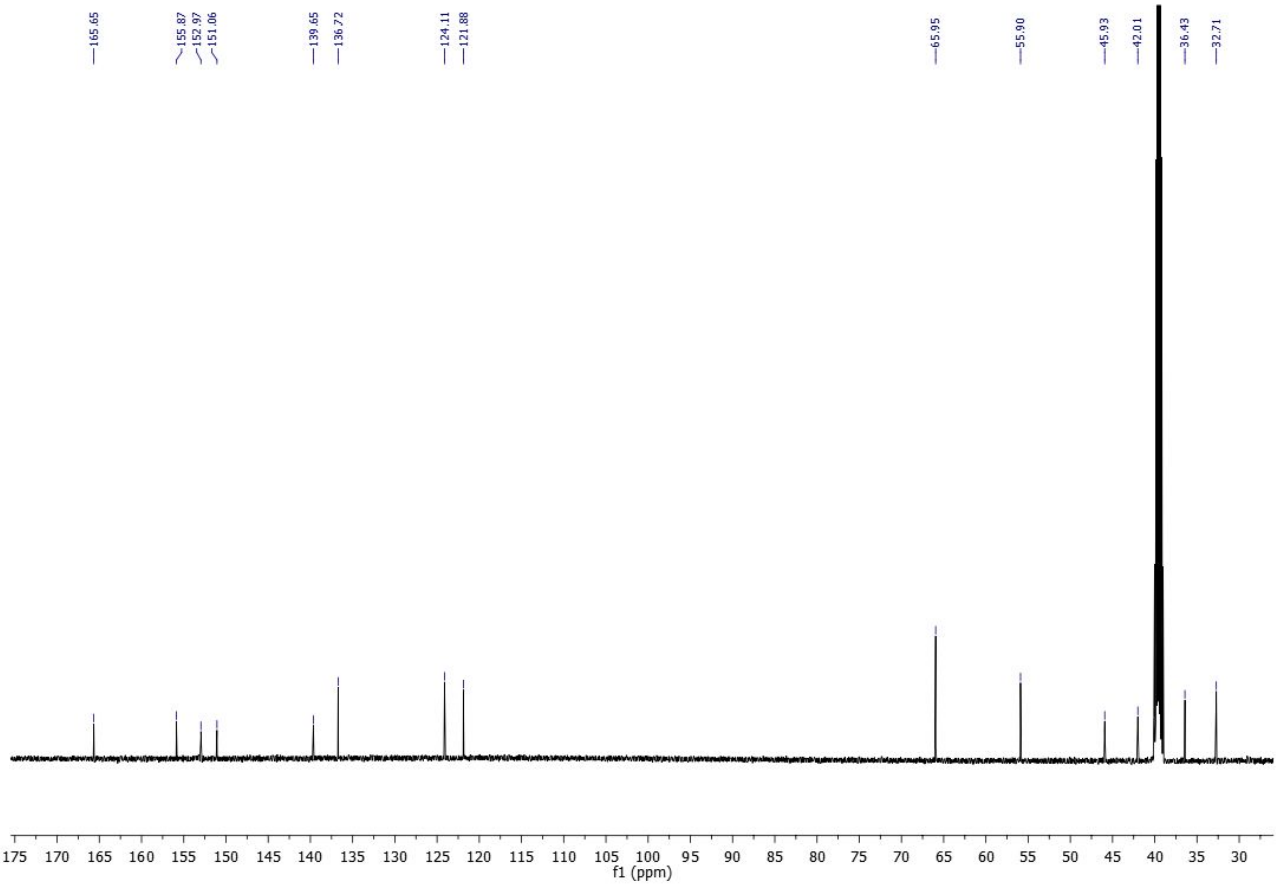

UPLC-MS (MeOH): m/z 350.26 $[M+H]^+$. Rt. = 0.66 min. Analysis type: LCMS acid method.

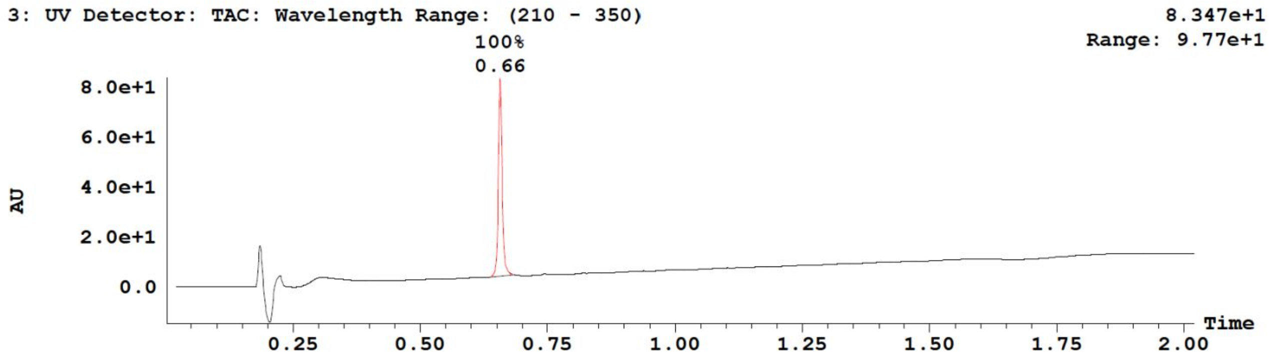

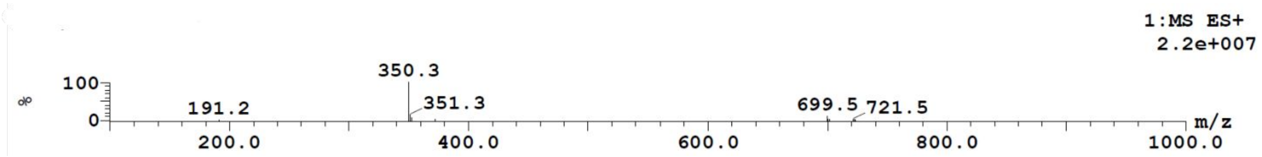

HRMS (ESI-TOF, Exploris 240): experimental m/z 350.1280 $[M+H]^+$, theoretical m/z 350.1281 $[M+H]^+$. Δ = 0.0001.

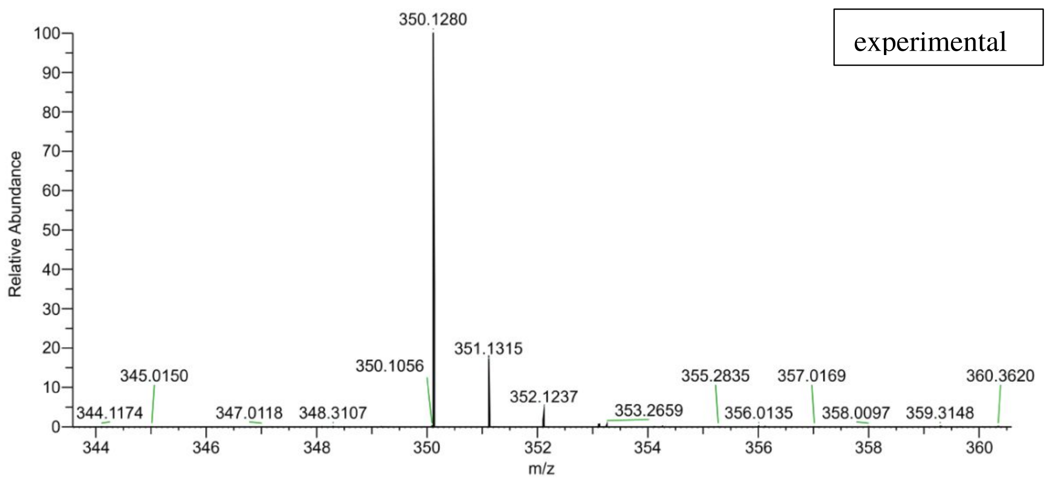

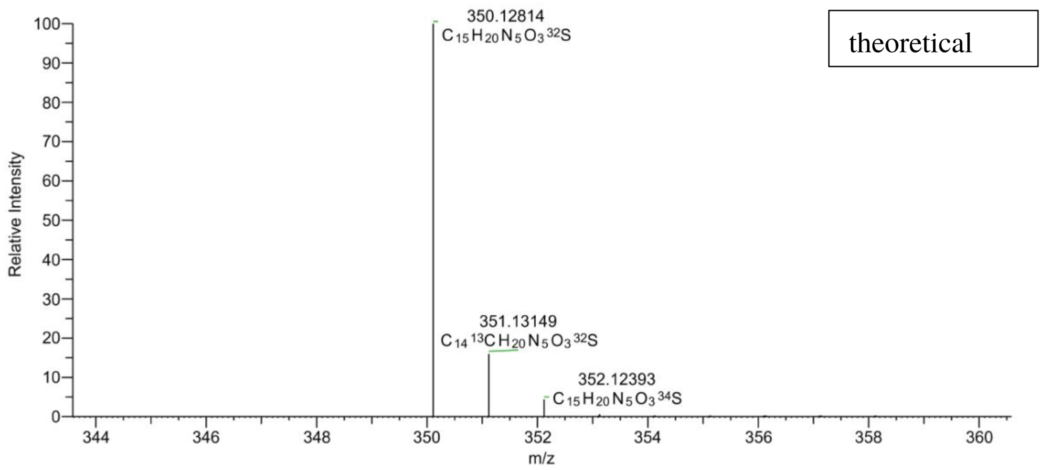

Compound 11b

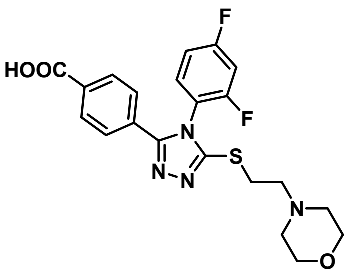

^{1}H NMR (Bruker 500 MHz, CD_3OD) δ 8.08 – 8.00 (m, 2H), 7.73 – 7.60 (m, 1H), 7.59 – 7.49 (m, 2H), 7.36 – 7.27 (m, 1H), 7.27 – 7.20 (m, 1H), 4.09 – 3.86 (m, 4H), 3.72 – 3.60 (m, 4H), 3.30 (dt, *J* = 3.3, 1.6 Hz, 4H).

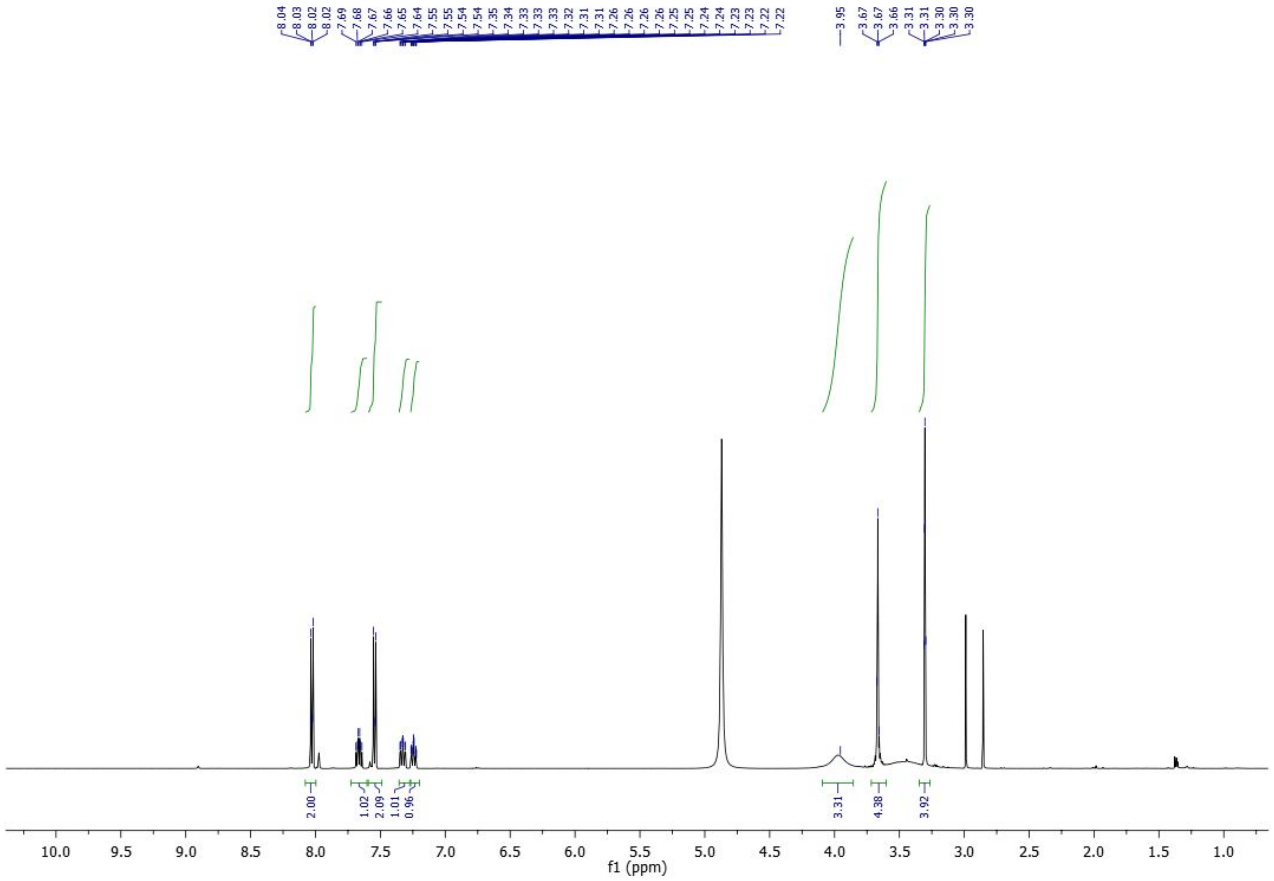

HSQC, HMBC (Bruker 500 MHz, CD_3OD) δ 166.68 (C), 163.39 (C), 155.17 (C), 152.94 (C), 132.50 (C), 131.21 (CH), 129.96 (2 CH), 129.02 (C), 127.82 (2 CH), 116. 88 (C), 113.31 (CH), 105.64 (CH), 104.65 (C), 63.87 (2 CH_2), 56.71 (CH_2), 48.08 (2 CH_2), 25.53 (CH_2).

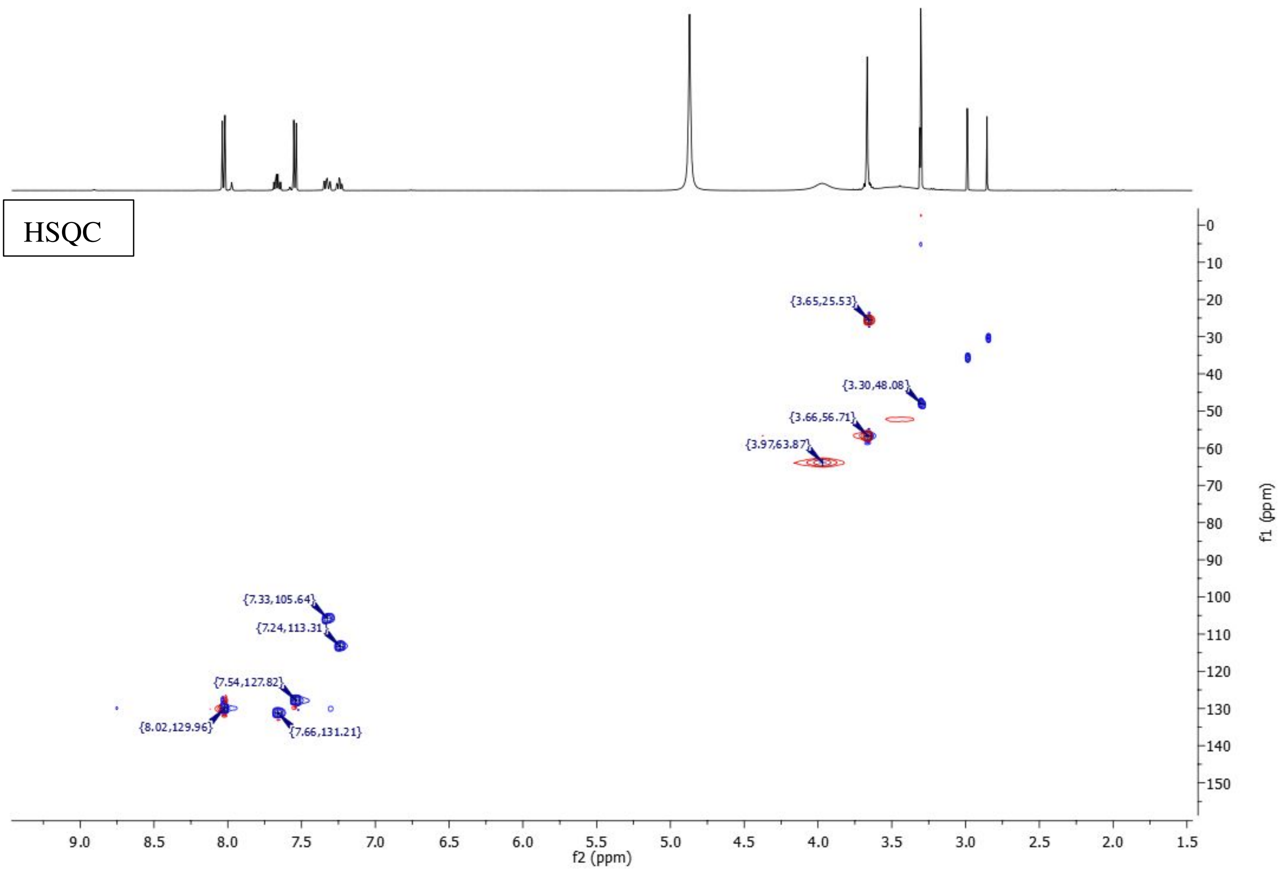
HSQC
{3.65,25.53}
{3.30,48.08}
{3.66,56.71}
{3.97,63.87}
{7.33,105.64}
{7.24,113.31}
{7.54,127.82}
{8.02,129.96}
{7.66,131.21}
f1 (ppm)
f2 (ppm)

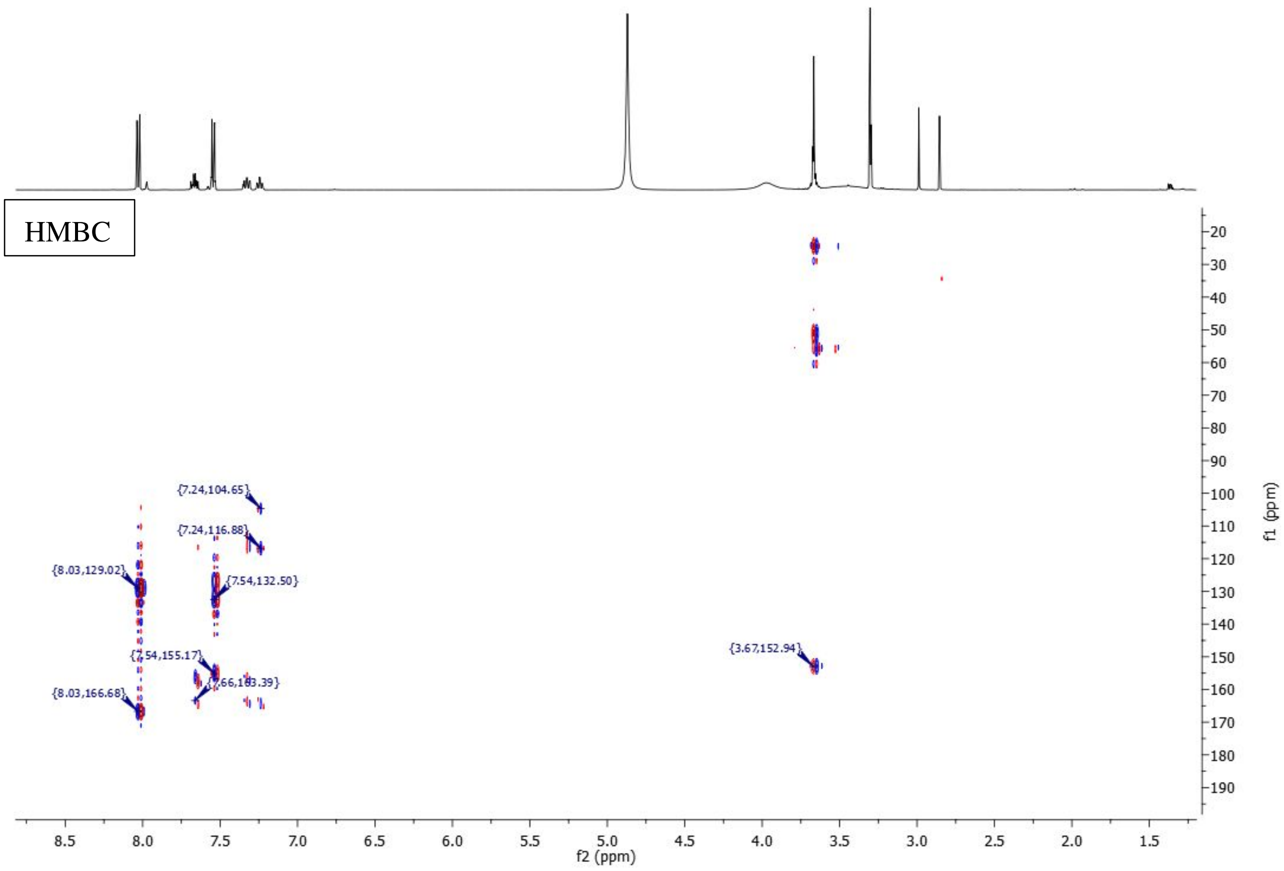
HMBC
{7.24,104.65}
{7.24,116.88}
{8.03,129.02}
{7.54,132.50}
{7.54,155.17}
{7.66,163.39}
{8.03,166.68}
{3.67,152.94}
f1 (ppm)
f2 (ppm)

UPLC-MS (MeOH): 447.28 [M+H]$^+$. Rt. = 0.57 min. Analysis type: LCMS acid method.

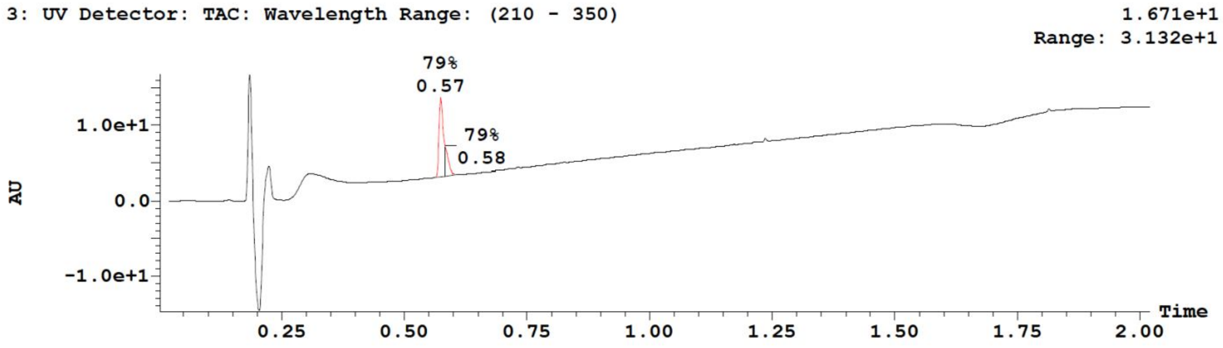

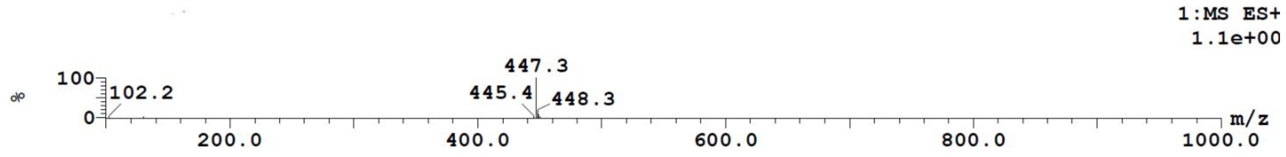

Compound 12

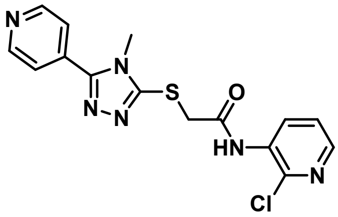

^{1}H NMR (Bruker 500 MHz, DMSO-d_6) δ 10.13 (s, 1H), 8.77 (dd, J = 4.5, 1.6 Hz, 2H), 8.28 – 8.18 (m, 2H), 7.75 (dd, J = 4.5, 1.6 Hz, 2H), 7.44 (dd, J = 8.0, 4.7 Hz, 1H), 4.26 (s, 2H), 3.72 (s, 3H).

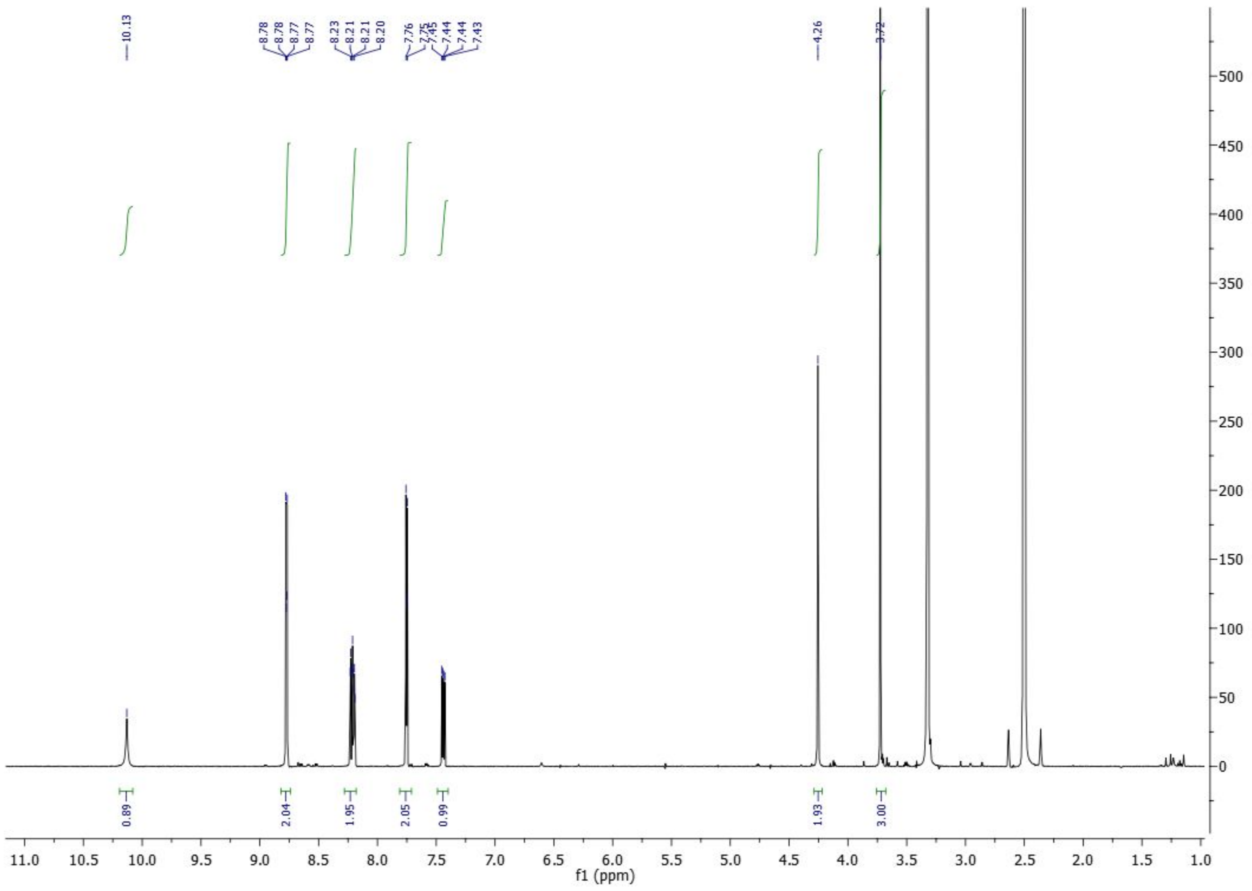

HSQC, HMBC (Bruker 500 MHz, DMSO-d_6) δ 167.02 (C), 153.41 (C), 151.74 (C), 150.15 (2 CH), 145.37 (C), 145. 19 (CH), 142.60 (C), 133.12 (CH), 123.27 (CH), 122.27 (C), 122.00 (2 CH), 36.71 (CH_2), 31.90 (CH_3)

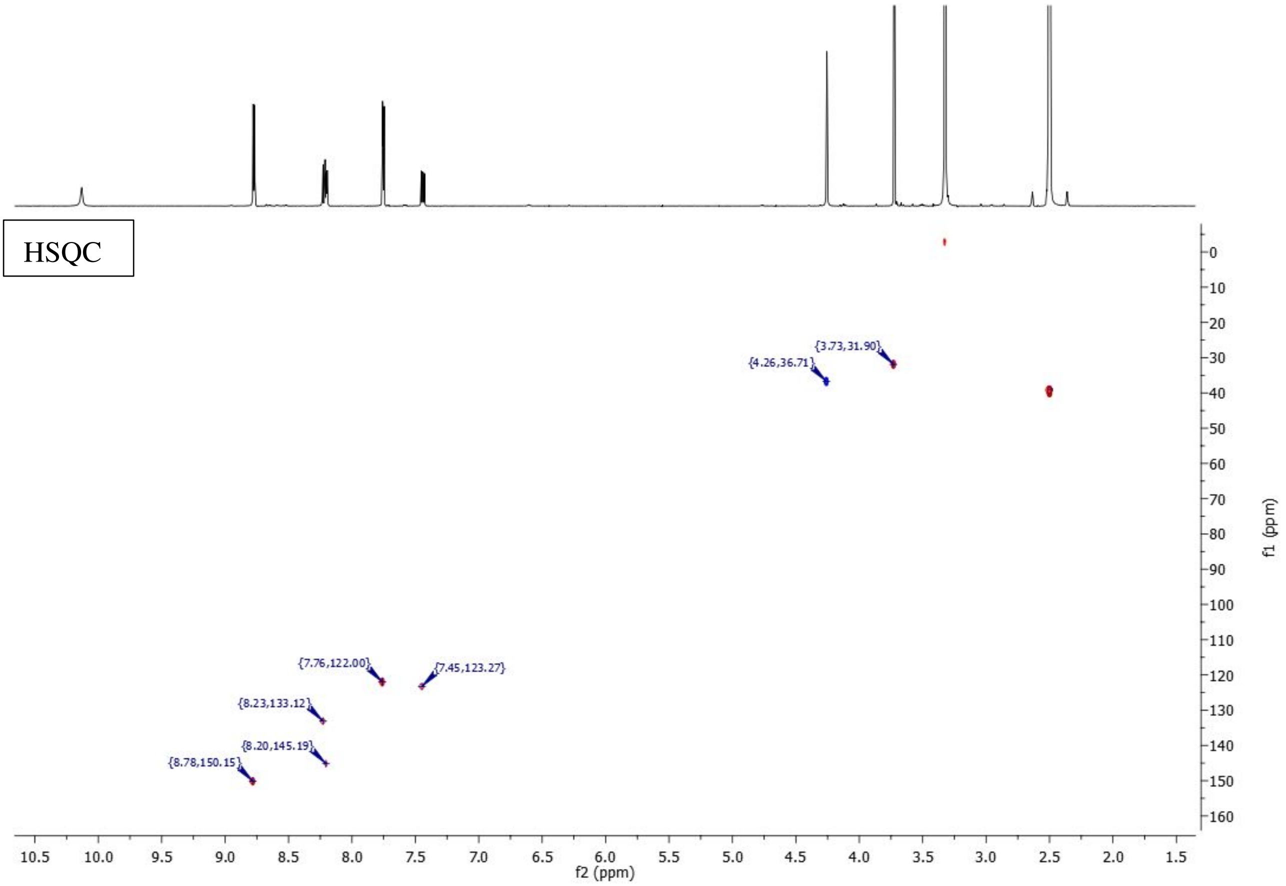

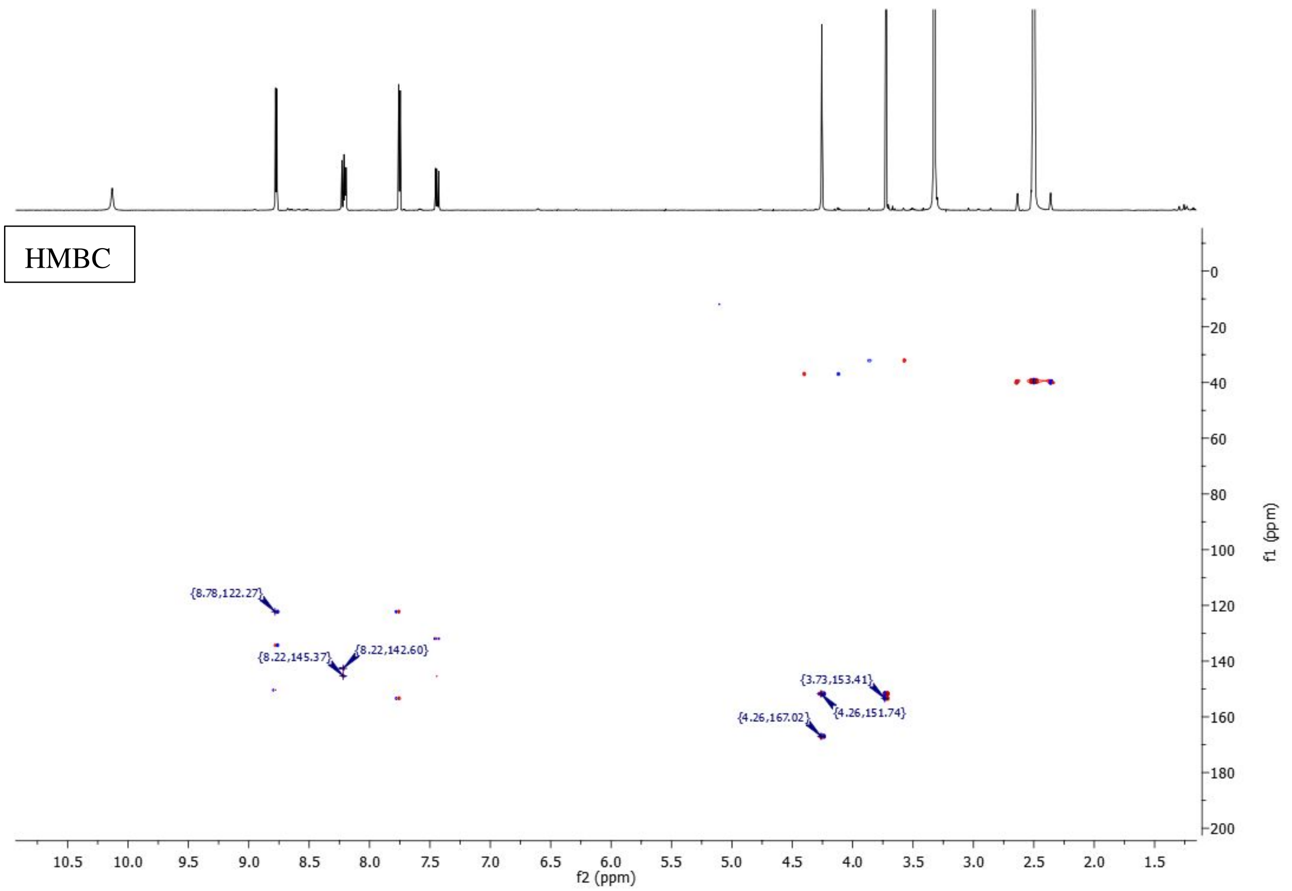

UPLC-MS (MeOH): m/z 361.28 [M+H]$^{+}$; 359.18 [M-H]$^{-}$. Rt. = 0.55 min. Analysis type: LCMS acid method.

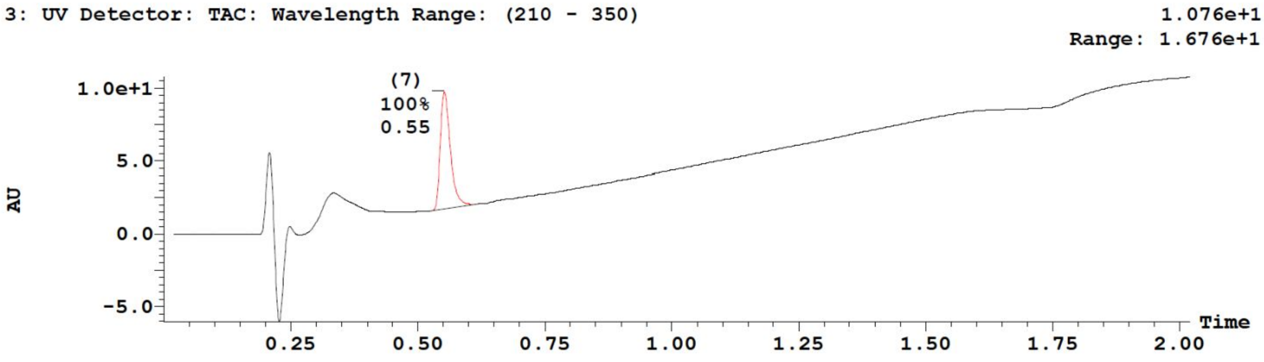

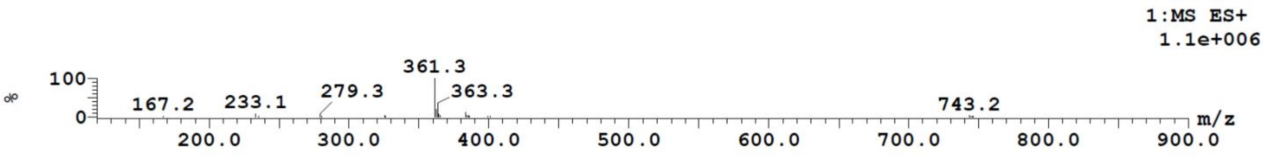

HRMS (ESI-TOF, Exploris 240): experimental m/z 361.0631 $[M+H]^+$, theoretical m/z 361.0633 $[M+H]^+$. Δ = 0.0002.

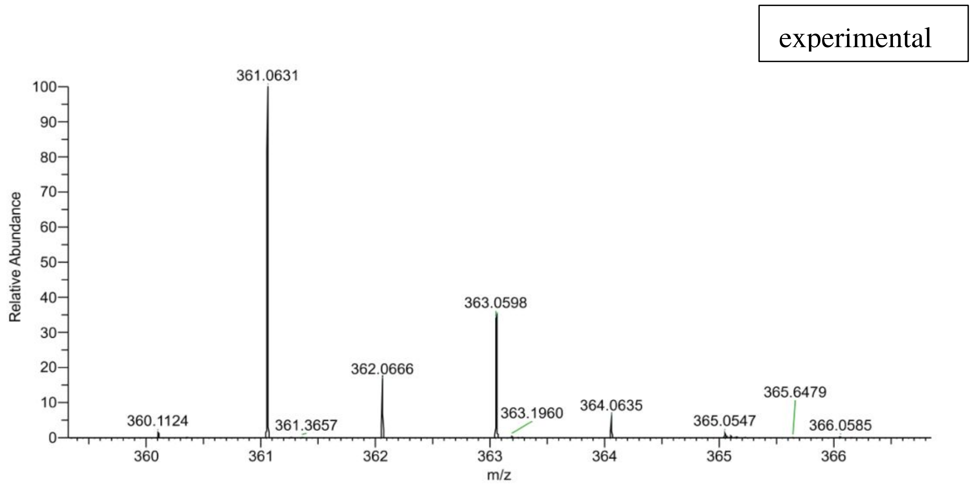

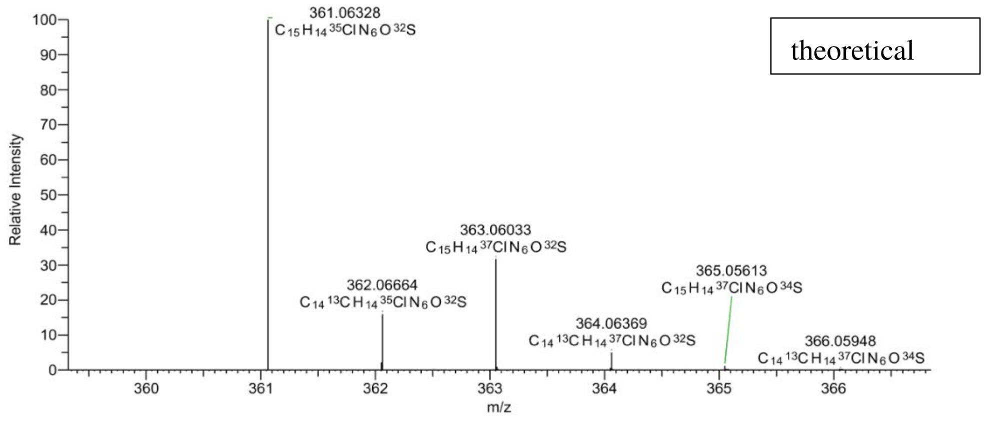

<u>**Compound 13**</u>

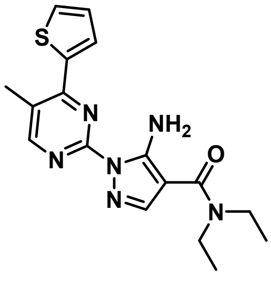

^{1}H NMR (Varian 400 MHz, DMSO-d_6) δ 8.75 (s, 1H), 7.95 – 7.94 (m, 1H), 7.93 (s, 1H), 7.72 (s, 1H), 7.70 (s, 2H), 7.33 (dd, J = 4.9, 4.1 Hz, 1H), 3.47 (q, J = 7.0 Hz, 4H), 2.57 (s, 3H), 1.18 (t, J = 7.0 Hz, 6H).

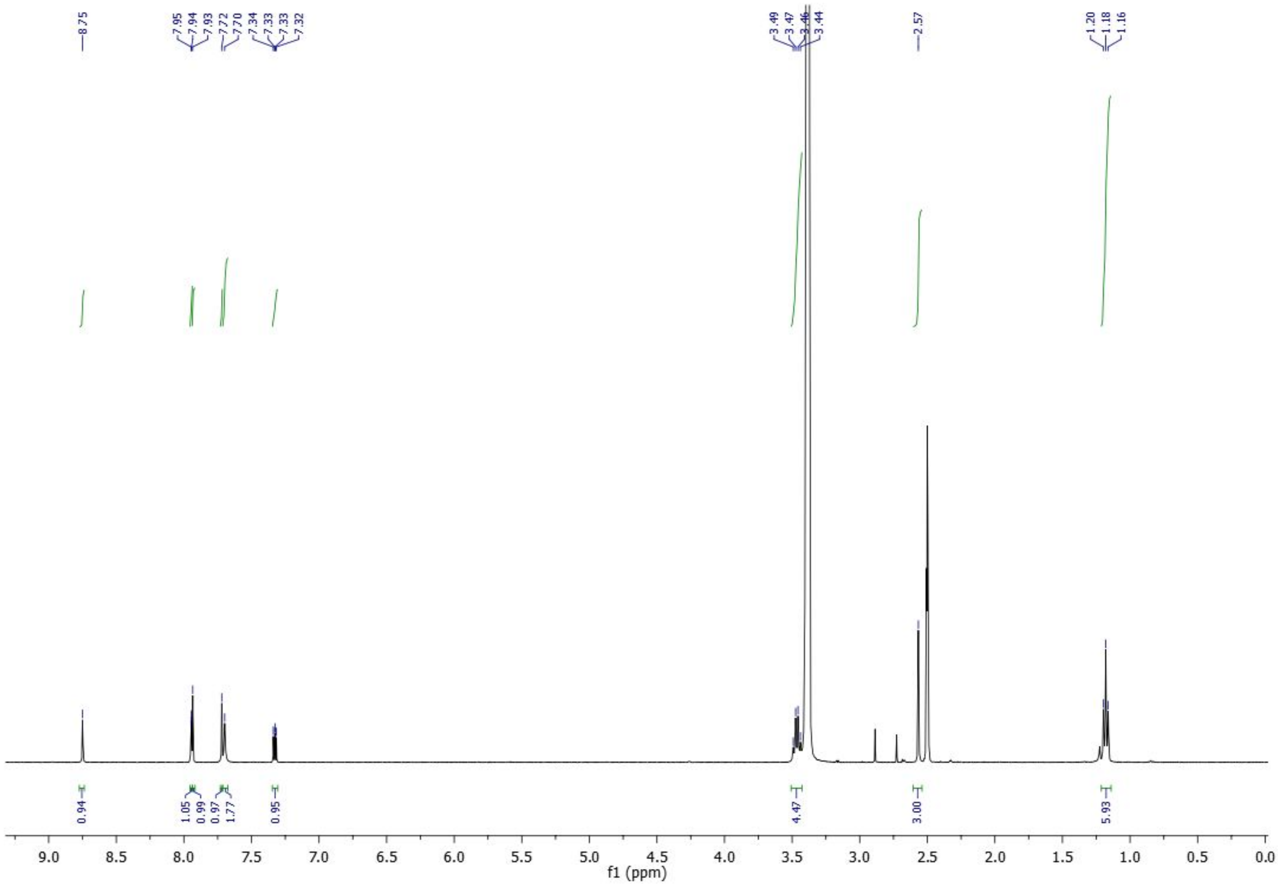

^{13}C NMR (Varian 101 MHz, DMSO-d_6) δ 163.98 (C), 161.06 (CH), 157.48 (C), 155.01 (C), 152.93 (C), 141.60 (C), 140.24 (CH), 131.74 (CH), 131.67 (CH), 129.36 (CH), 122.76 (C), 95.69 (C), 41.12 (2 CH_2), 17.50 (CH_3), 13.80 (2 CH_3).

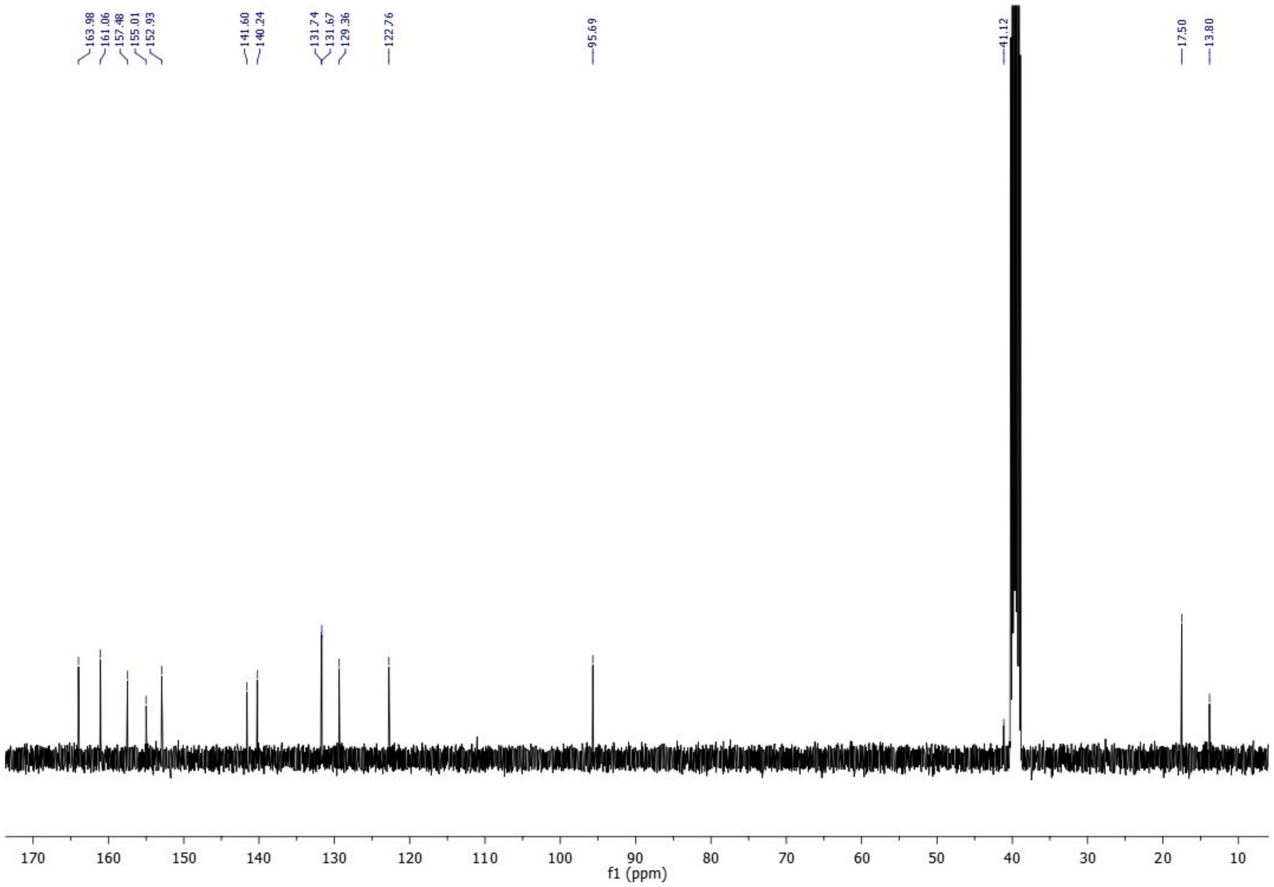

HRMS (ESI-TOF, Exploris 240): experimental m/z 357.1492 [M+H]$^+$, theoretical m/z 357.1491 [M+H]$^+$. Δ = 0.0001.

experimental

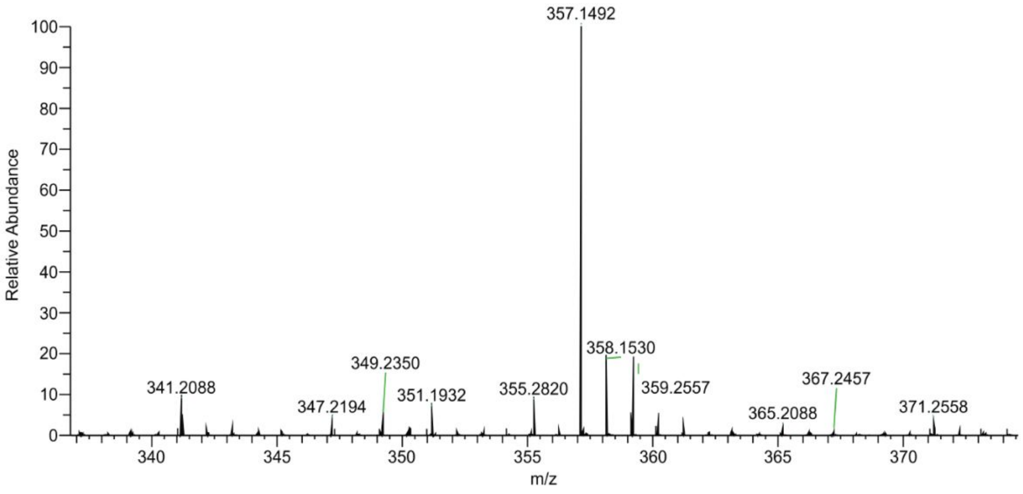
357.1492
358.1530
349.2350
341.2088
351.1932
355.2820
359.2557
367.2457
347.2194
365.2088
371.2558
Relative Abundance
m/z

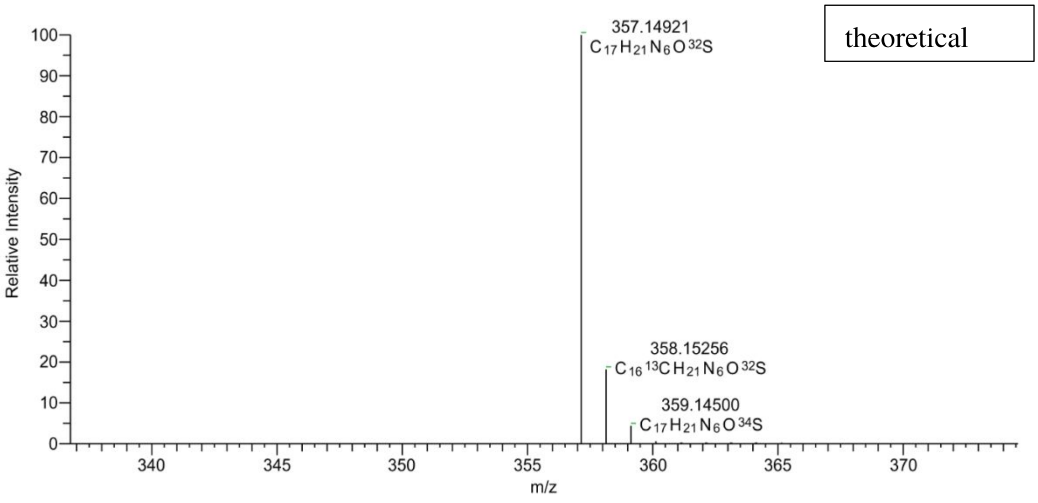
theoretical
357.14921
C17H21N6O32S
358.15256
C16 13CH21N6O32S
359.14500
C17H21N6O34S
Relative Intensity
m/z

Compound 14

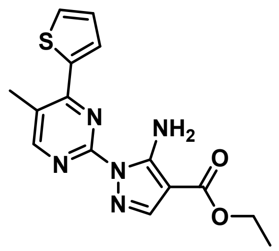

^{1}H NMR (Varian 400 MHz, DMSO-d_6) δ 8.76 (s, 1H), 8.00 – 7.90 (m, 2H), 7.79 (s, 1H), 7.56 (s, 2H), 7.33 (dd, J = 5.1, 3.9 Hz, 1H), 4.23 (q, J = 7.1 Hz, 2H), 2.57 (s, 3H), 1.29 (t, J = 7.1 Hz, 3H).

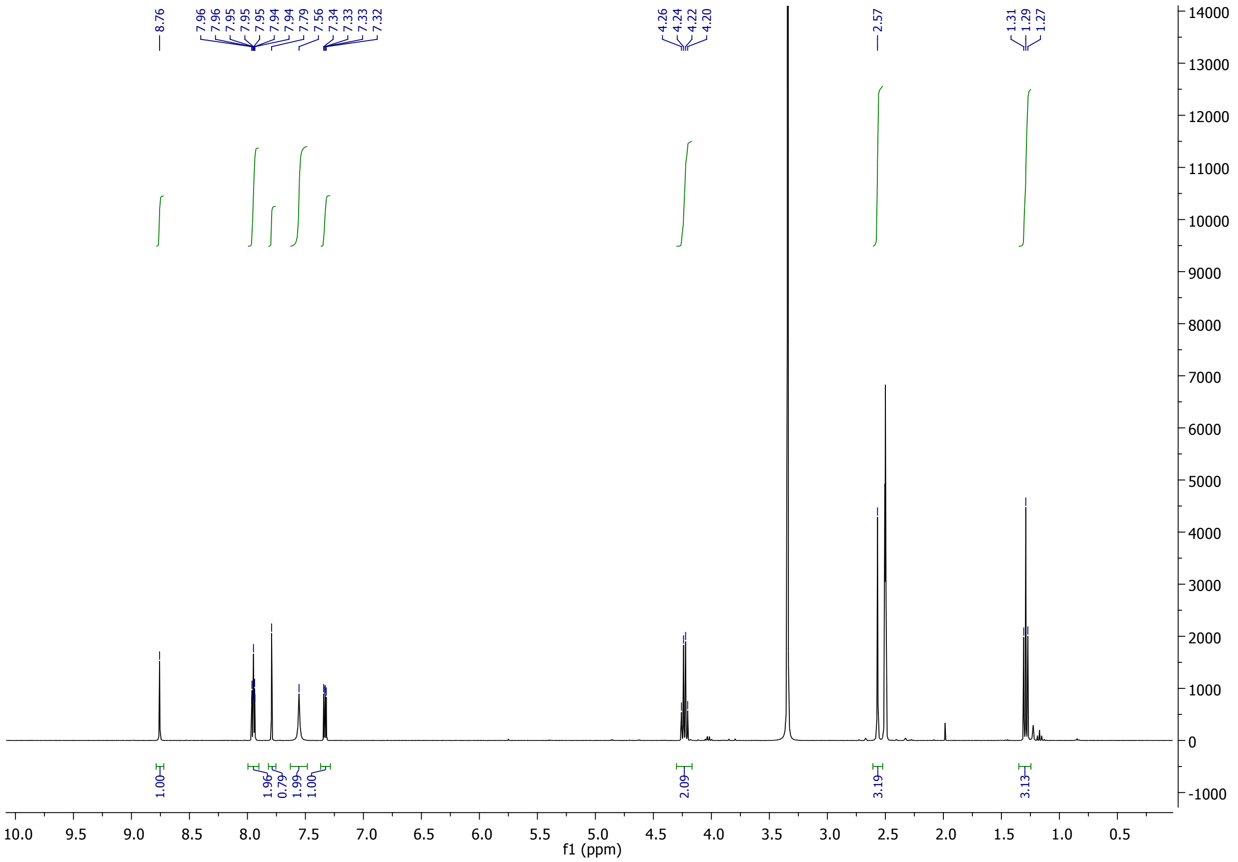

HRMS (ESI-TOF, Exploris 240): experimental m/z 352.0834 $[M+H]^+$.

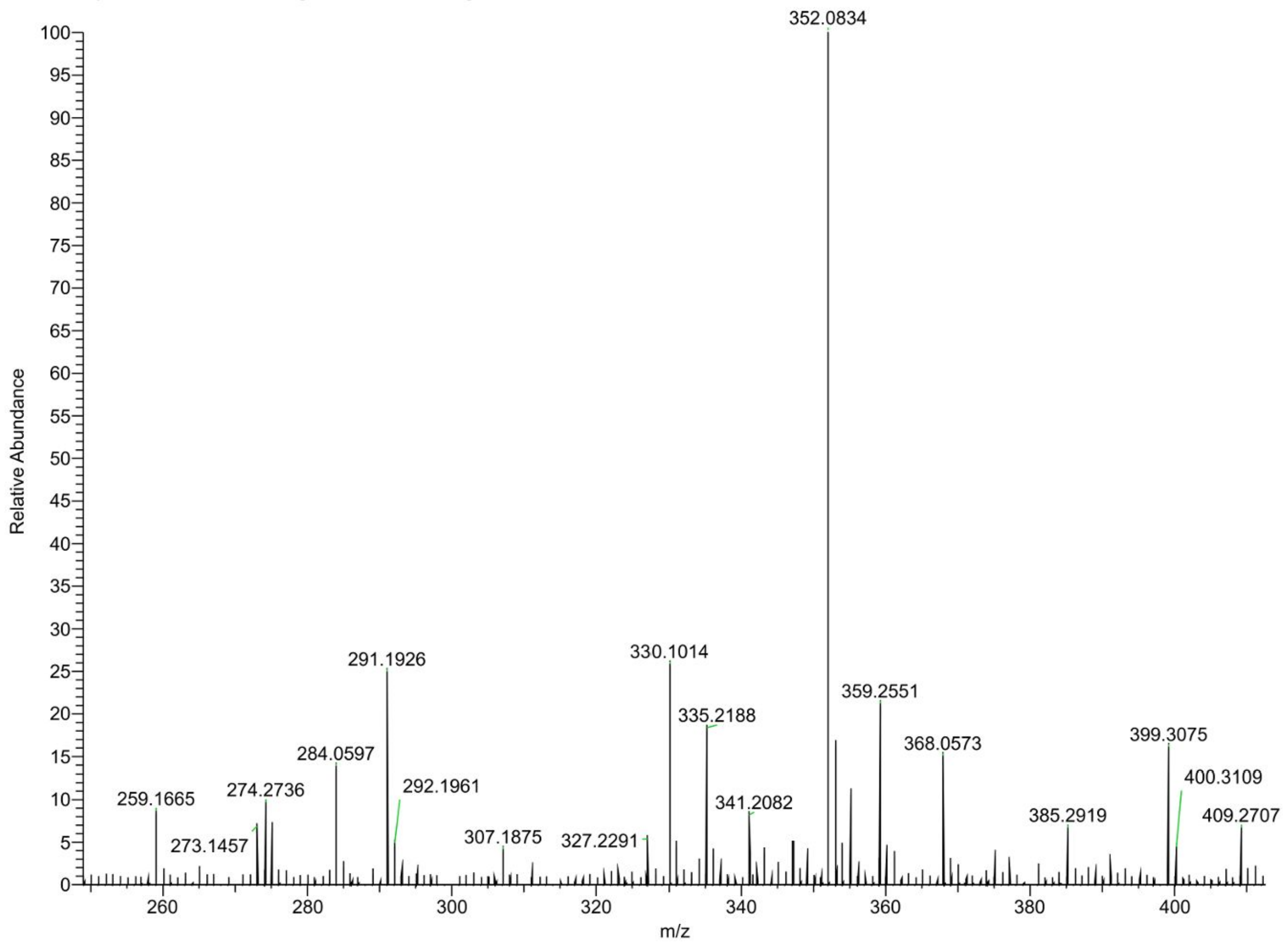

Compound 15

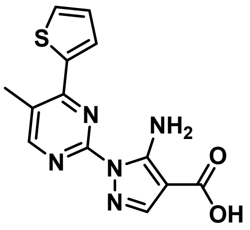

^{1}H NMR (Varian 400 MHz, DMSO-d_6) δ 12.15 (s, 1H), 8.75 (s, 1H), 8.01 – 7.89 (m, 2H), 7.76 (s, 1H), 7.49 (s, 2H), 7.33 (dd, J = 5.0, 3.9 Hz, 1H), 2.57 (s, 3H).

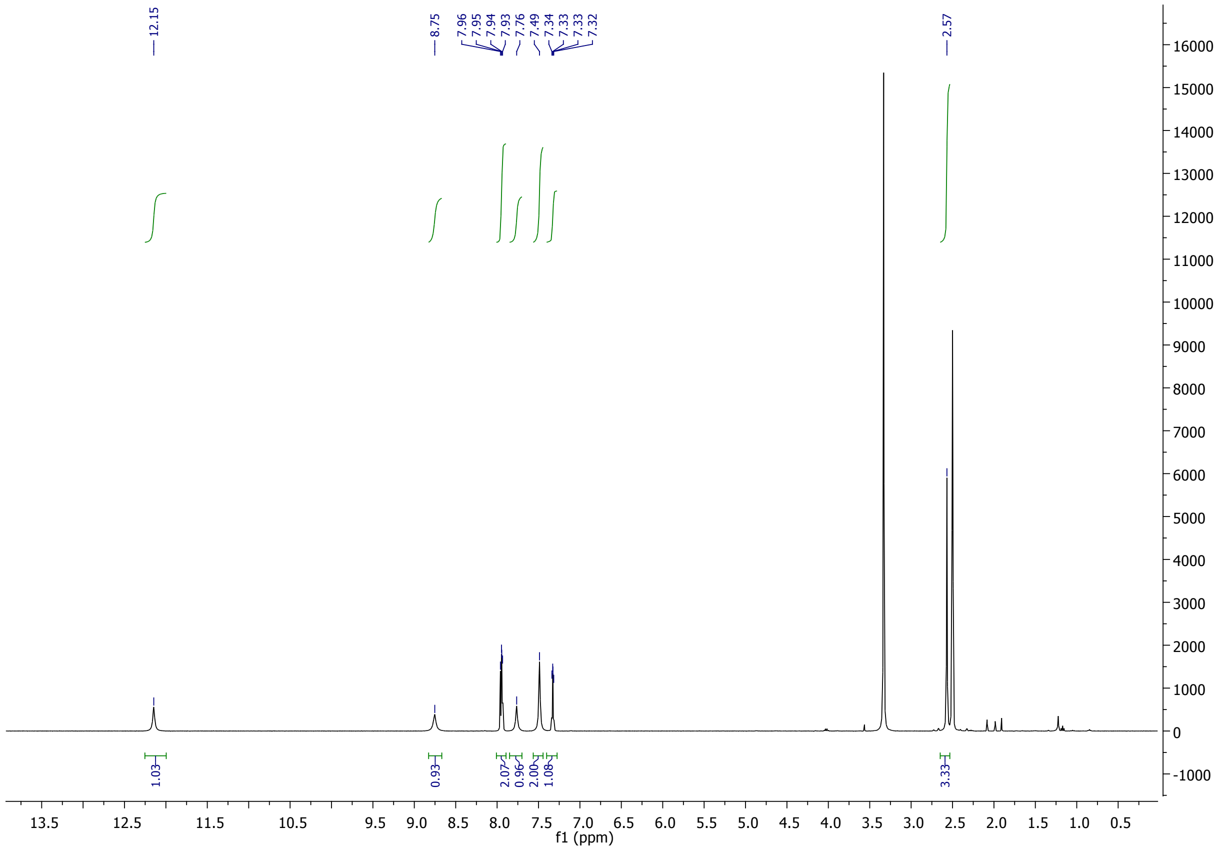

HRMS (ESI-TOF, Exploris 240): experimental m/z 300.0557 [M-H]$^-$.

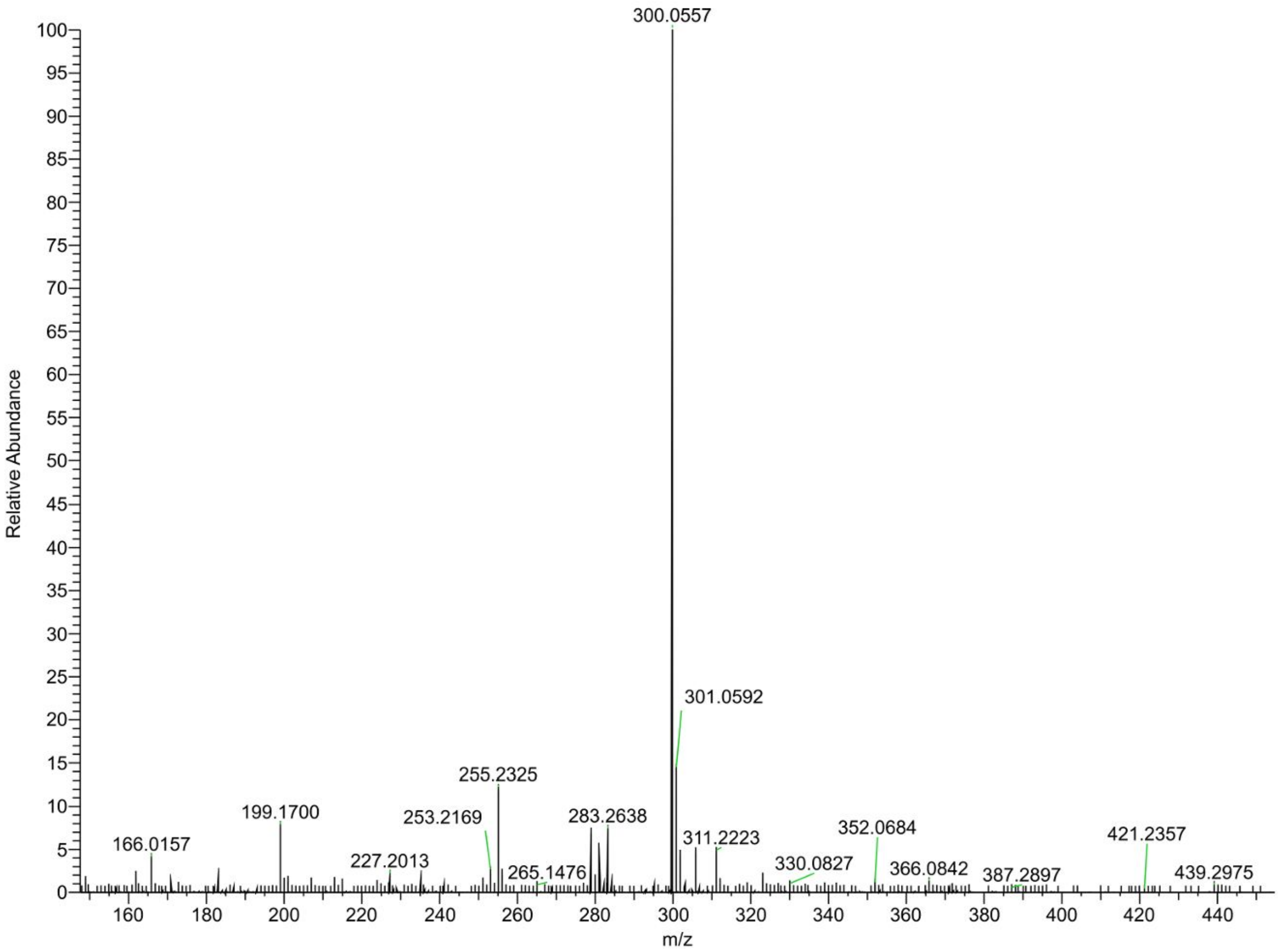

Compound 16

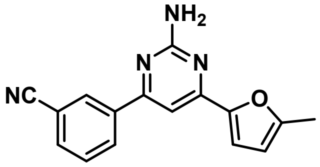

^{1}H NMR (Varian 500 MHz, DMSO-d_6) δ 8.58 (s, 1H), 8.46 (dt, J = 8.0, 1.3 Hz, 1H), 7.99 – 7.96 (m, 1H), 7.73 (t, J = 8.0 Hz, 1H), 7.53 (s, 1H), 7.26 (d, J = 3.3 Hz, 1H), 6.83 (s, 2H), 6.34 (dd, J = 3.3, 1.0 Hz, 1H), 2.39 (s, 3H).

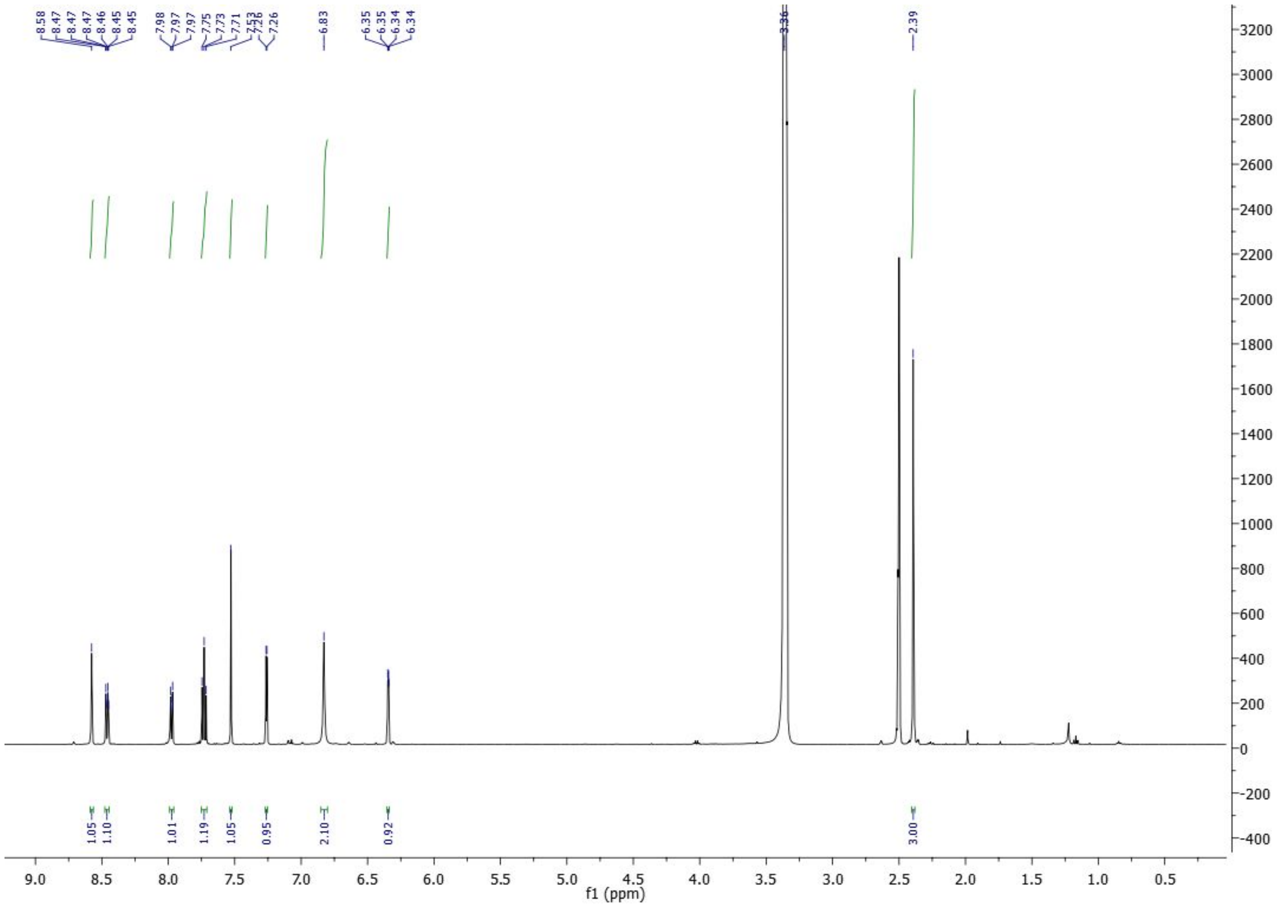

^{13}C NMR (Varian 126 MHz, DMSO-d_6) δ 163.86 (C), 162.06 (C), 156.99 (C), 154.86 (C), 150.25 (C), 138.31 (C), 133.80 (CH), 131.32 (CH), 130.41 (CH), 130.03 (CH), 118.64 (C), 113.66 (CH), 111.92 (C), 108.96 (CH), 99.78 (CH), 13.65 (CH_3).

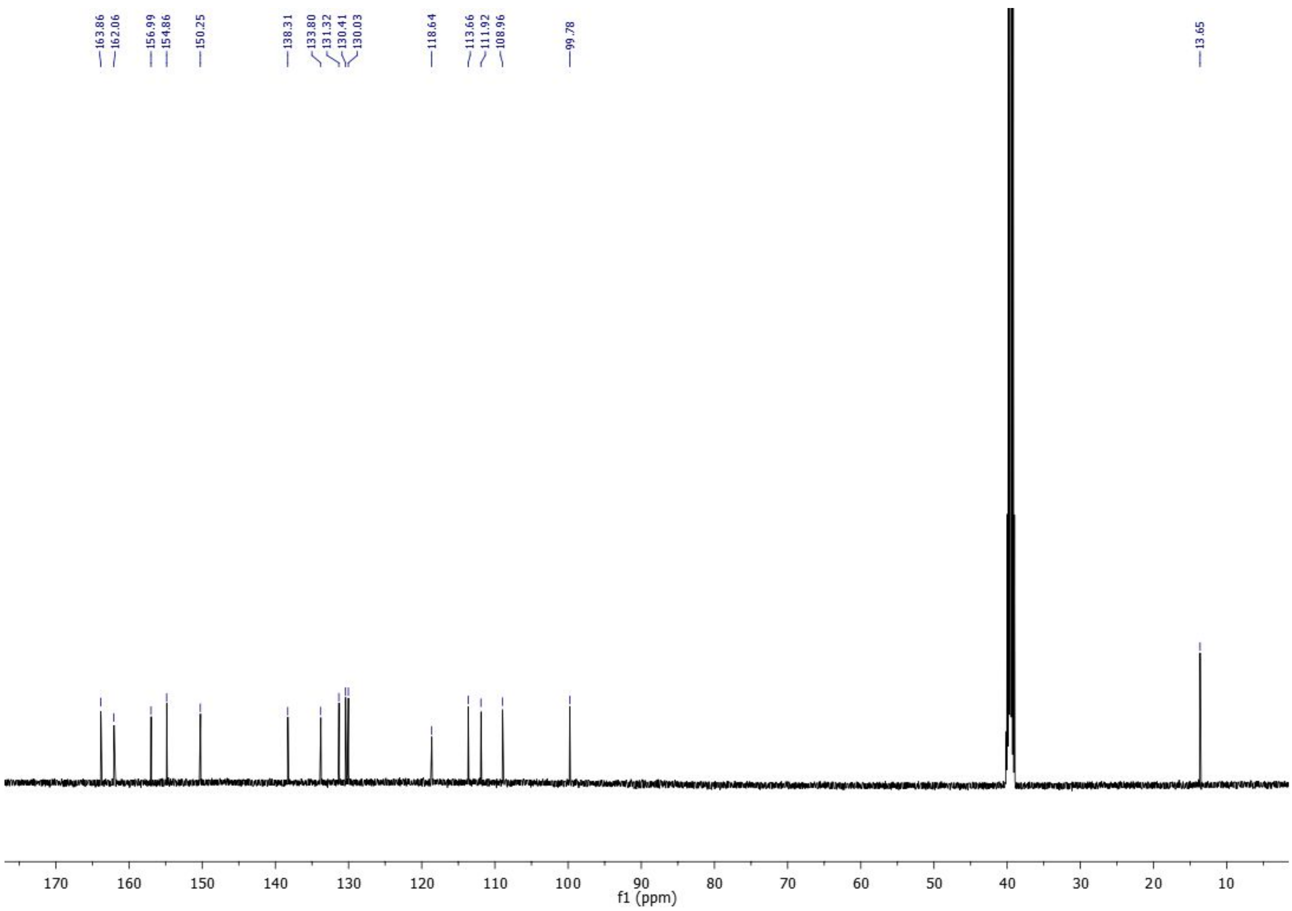

HRMS (ESI-TOF, Exploris 240): experimental m/z 277.1083 $[M+H]^+$, theoretical m/z 277.1084 $[M+H]^+$. Δ = 0.0001.

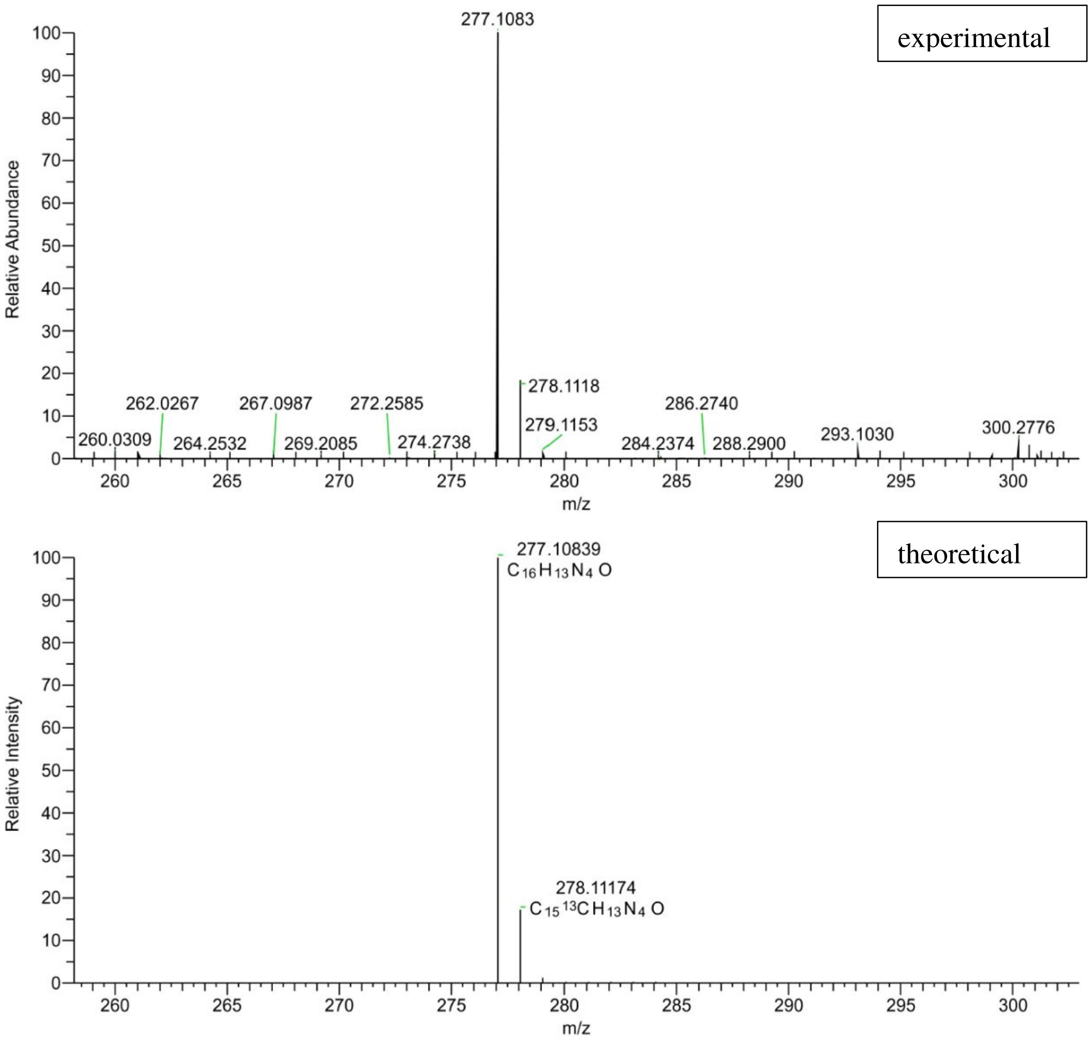

Compound 17

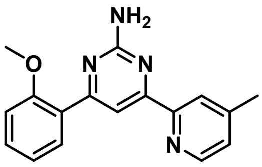

H NMR (Varian 400 MHz, DMSO-d_6) δ 8.56 (d, J = 4.9 Hz, 1H), 8.21 – 8.17 (m, 1H), 7.98 (s, 1H), 7.79 (dd, J = 7.7, 1.8 Hz, 1H), 7.48 – 7.43 (m, 1H), 7.35 (dd, J = 4.9, 0.8 Hz, 1H), 7.17 (d, J = 7.7 Hz, 1H), 7.07 (td, J = 7.5, 0.8 Hz, 1H), 6.70 (s, 2H), 3.85 (s, 3H), 2.42 (s, 3H).

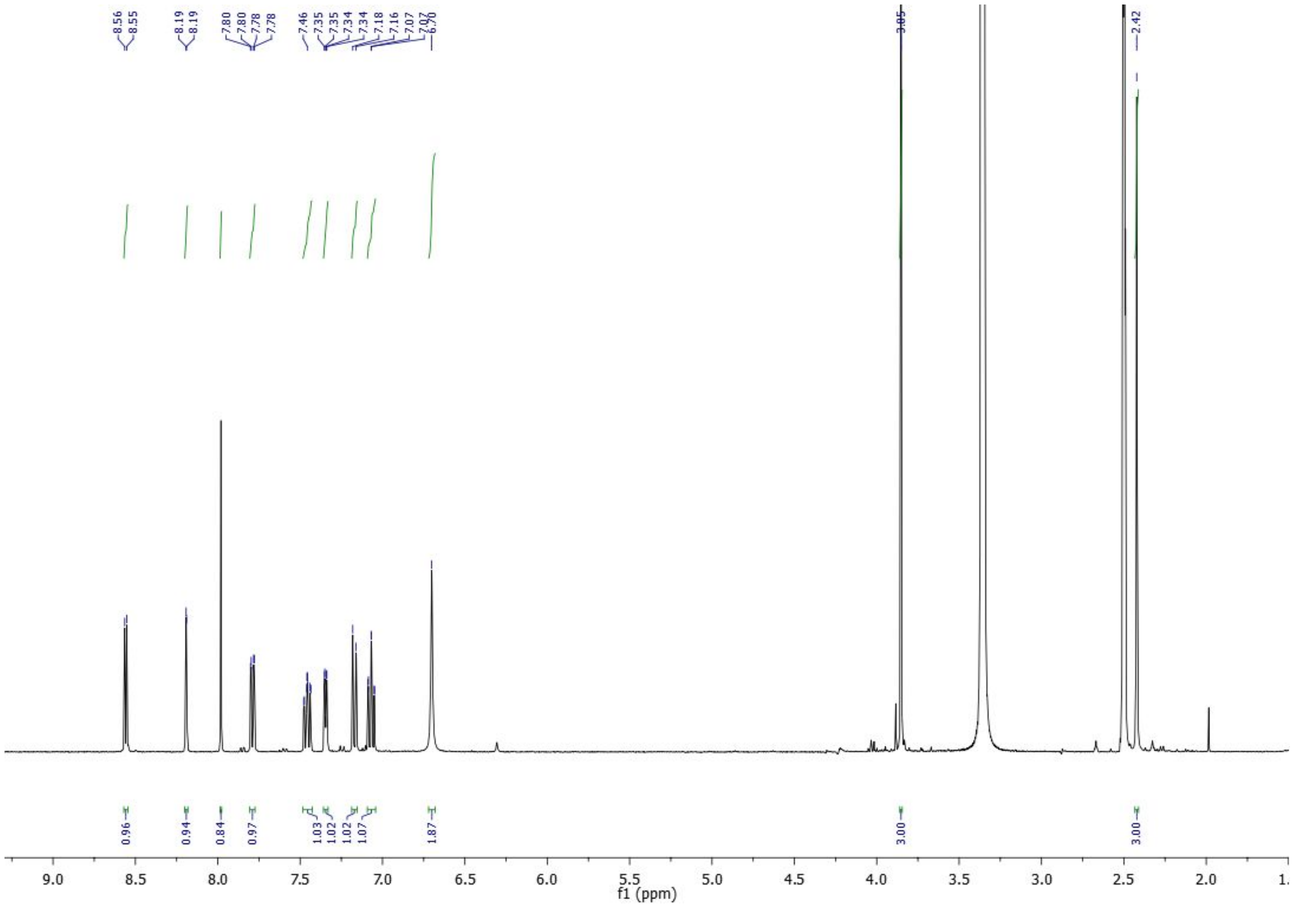

^{13}C NMR (Varian 101 MHz, DMSO-d_6) δ 164.62 (C), 163.85 (C), 162.86 (C), 157.48 (C), 154.24 (C), 149.28 (CH), 147.91 (C), 131.17 (CH), 130.28 (CH), 126.95 (C), 126.95 (CH), 125.96 (CH), 120.47 (CH), 112.07 (CH), 106.87 (CH), 55.78 (CH_3), 20.73 (CH_3).

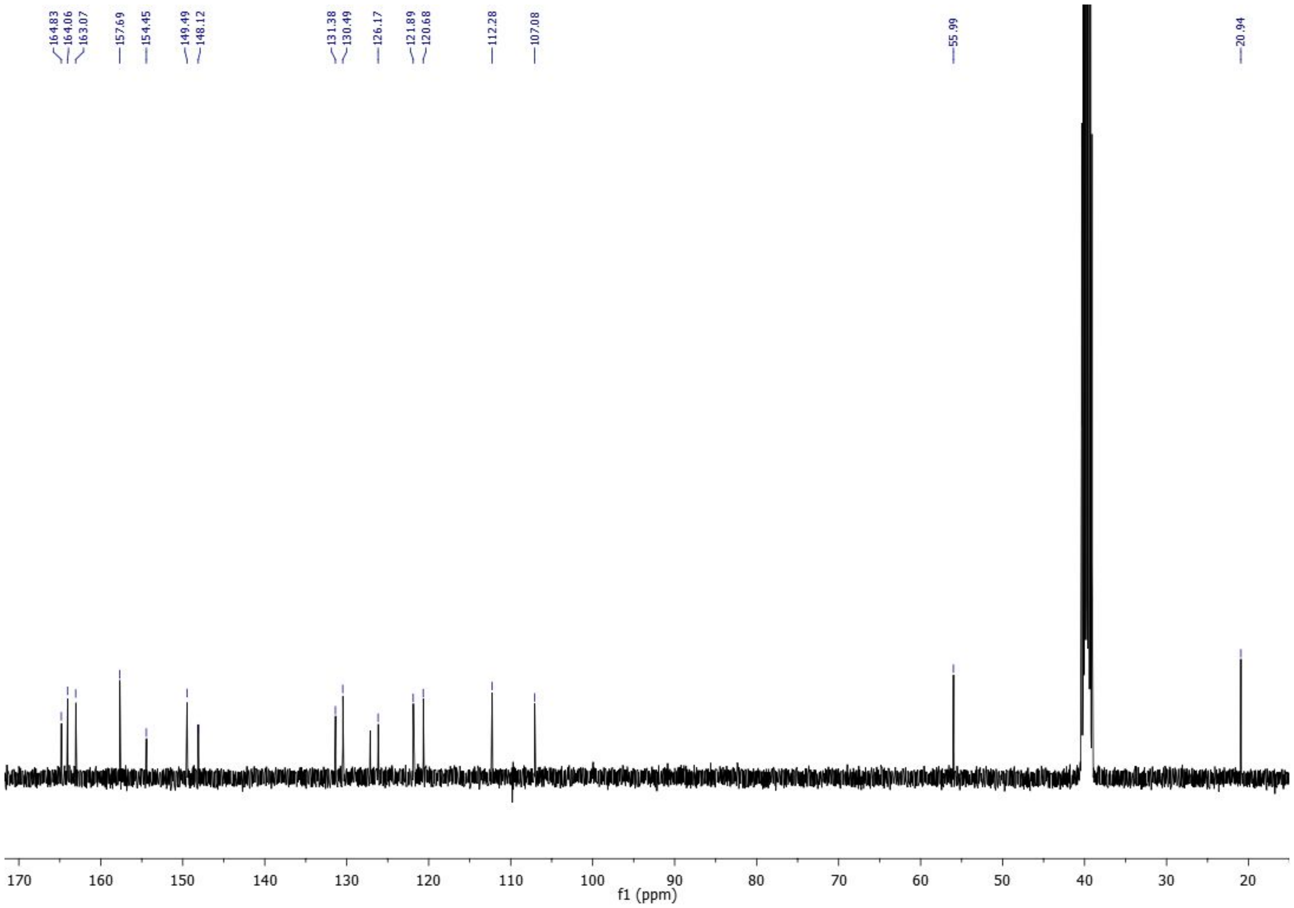

HRMS (ESI-TOF, Exploris 240): experimental m/z 293.1399 [M+H]+, theoretical m/z 293.1397 [M+H]+. Δ = 0.0002.

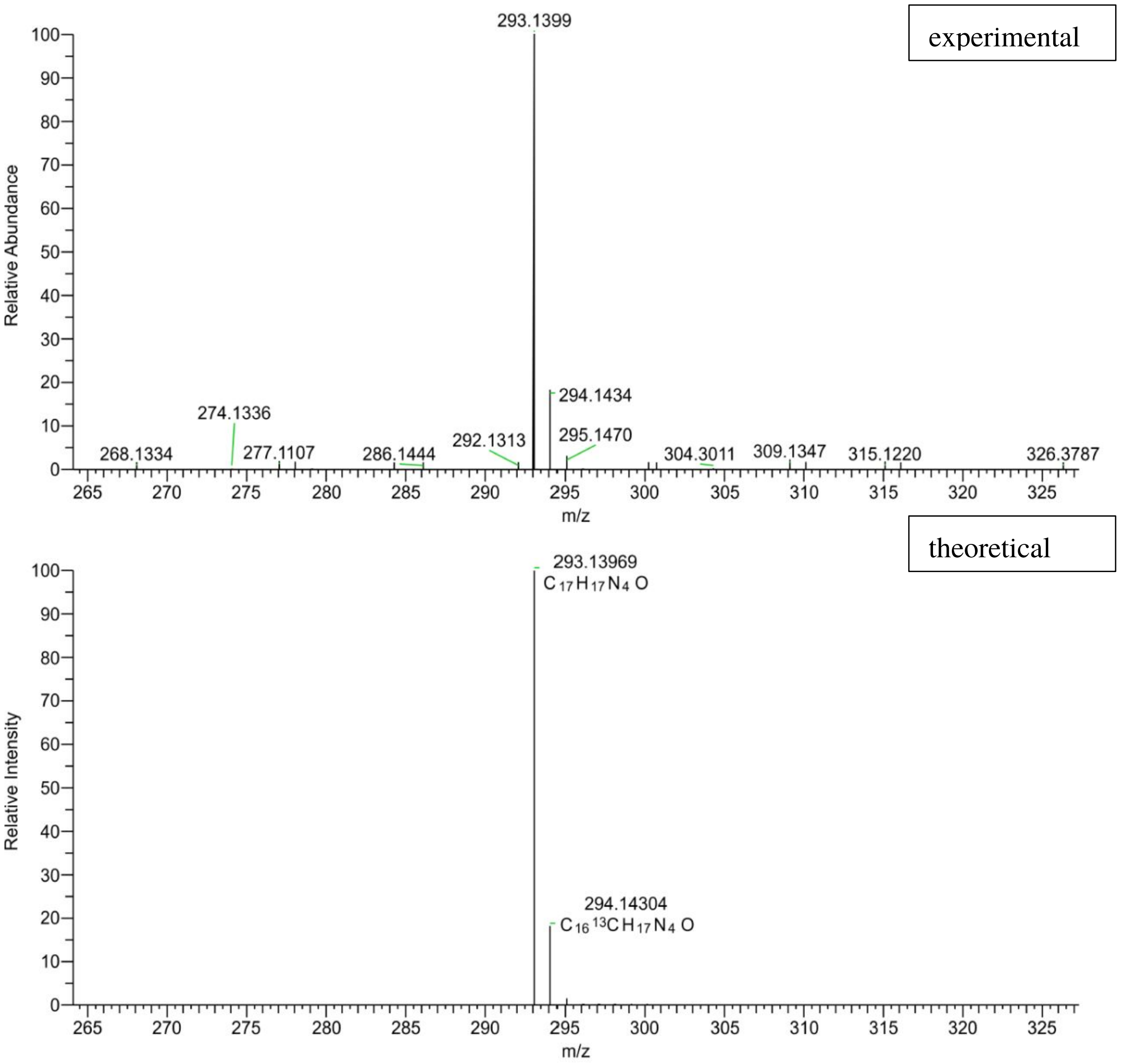

Compound 18

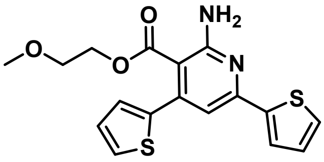

^{1}H NMR (Varian 500 MHz, DMSO-*d*$_6$) δ 7.86 (dd, J = 3.7, 0.9 Hz, 1H), 7.70 (dd, J = 5.1, 1.1 Hz, 1H), 7.67 (dd, J = 5.0, 0.9 Hz, 1H), 7.26 (dd, J = 3.6, 1.1 Hz, 1H), 7.19 (s, 1H), 7.17 - 7.13 (m, 2H), 6.53 (s, 2H), 4.28 – 4.17 (m, 2H), 3.40 – 3.36 (m, 2H), 3.17 (s, 3H).

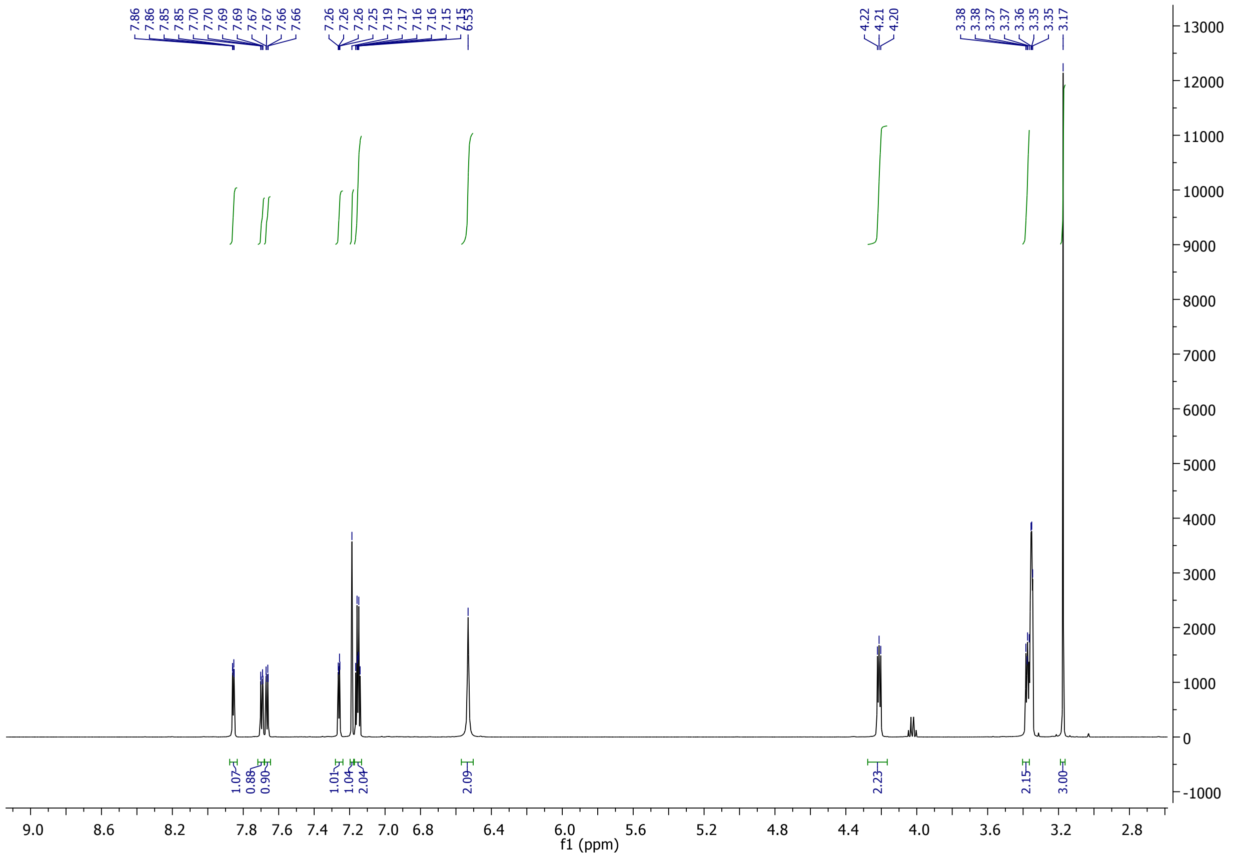

^{13}C NMR (Varian 126 MHz, DMSO-d_6) δ 167.34 (C), 157.16 (C), 152.25 (C), 143.73 (C), 143.42 (C), 140.08 (C), 129.21 (CH), 128.40 (CH), 127.78 (CH), 127.70 (CH), 127.21 (CH), 126.51 (CH), 108.15 (CH), 106.82 (C), 69.25 (CH_2), 63.78 (CH_2), 57.93 (CH_3).

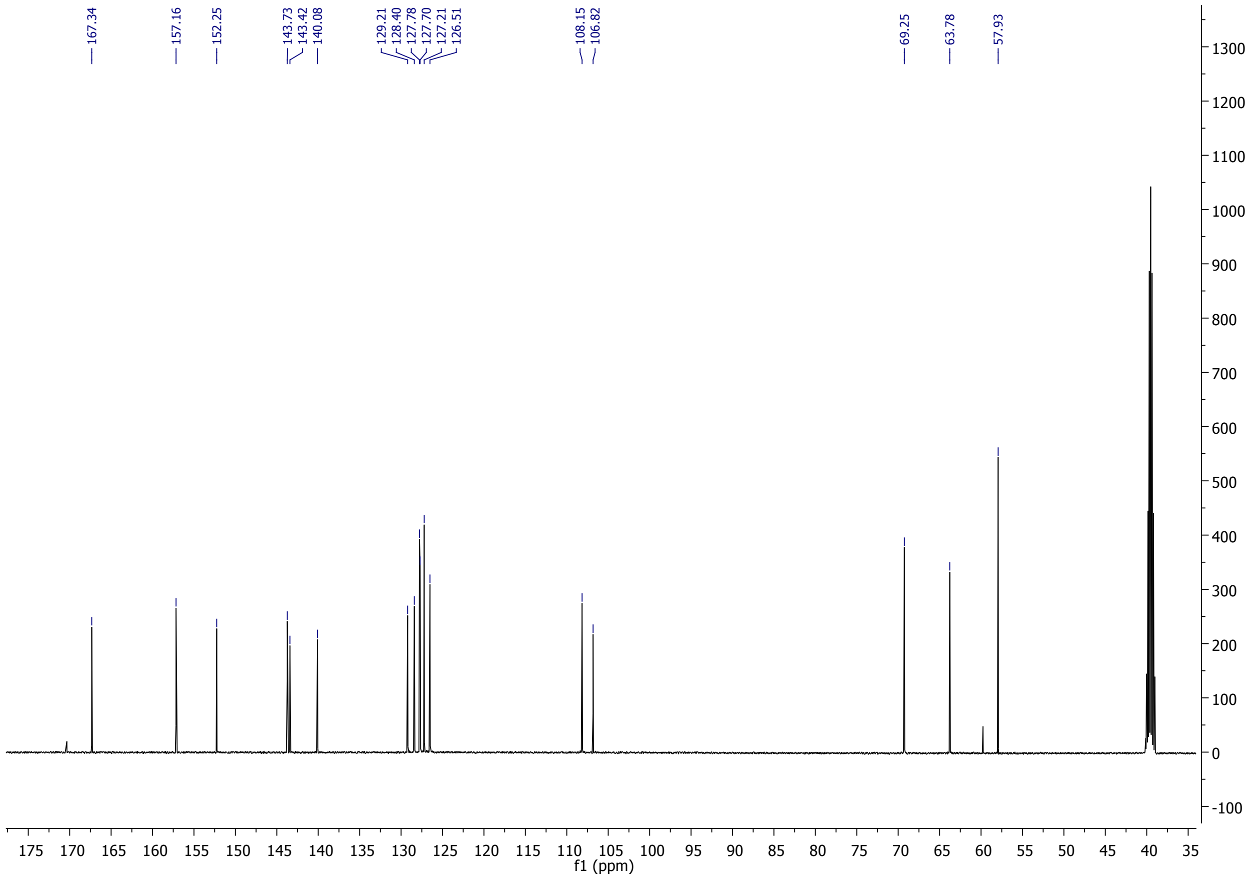

HRMS (ESI-TOF, Exploris 240): experimental m/z 361.0674 $[M+H]^+$, theoretical m/z 361.0675 $[M+H]^+$. $\Delta = 0.0001$.

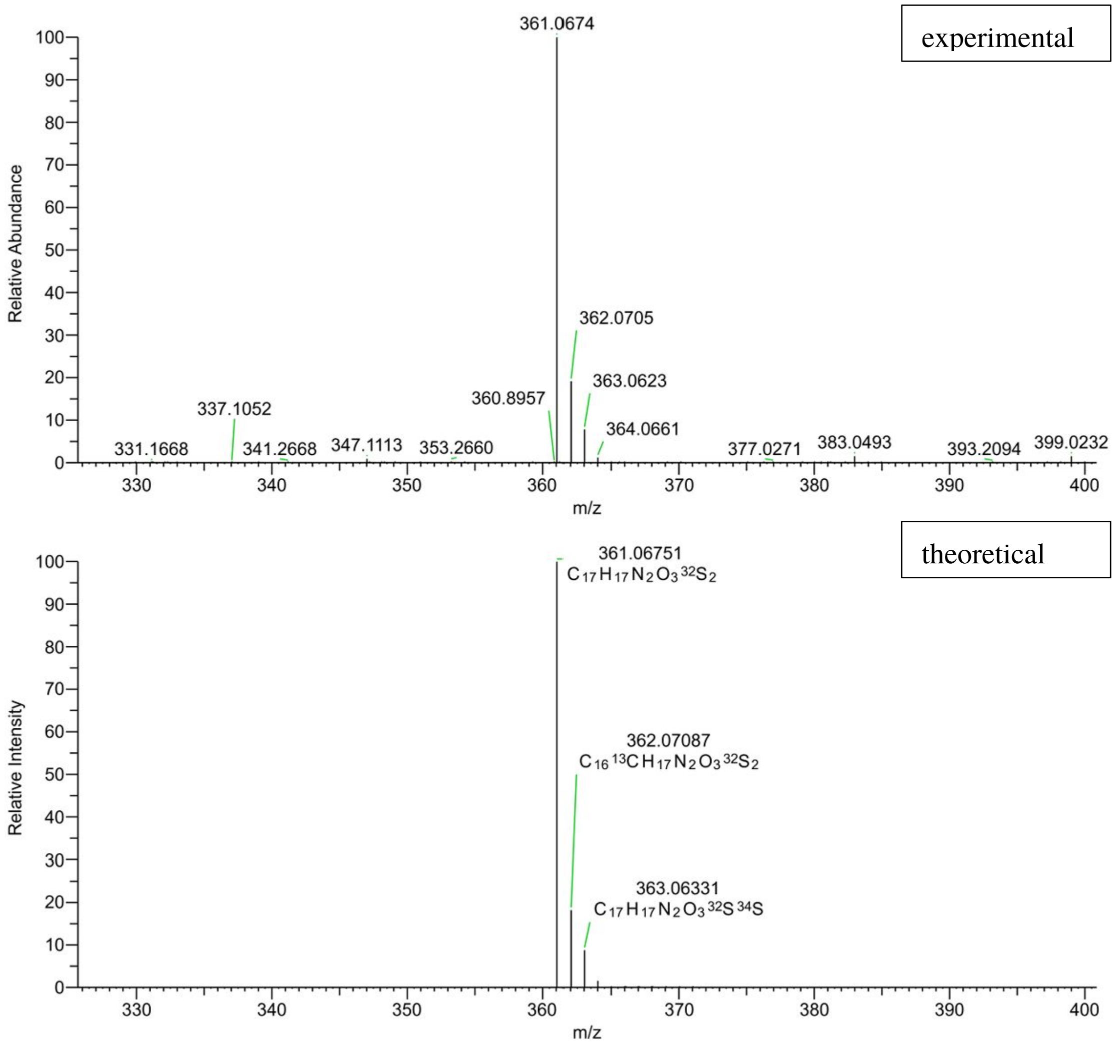

Supplement: Supplementary file 3 [file jm6c00231_si_003.pdf]
